# Hit-to-Lead Optimization of Heterocyclic Carbonyloxycarboximidamides as Selective Antagonists at Human Adenosine A3 Receptor

**DOI:** 10.1021/acs.jmedchem.4c01092

**Published:** 2024-07-29

**Authors:** Xianglin Huang, Anna Chorianopoulou, Panagoula Kalkounou, Maria Georgiou, Athanasios Pousias, Amy Davies, Abigail Pearce, Matthew Harris, George Lambrinidis, Panagiotis Marakos, Nicole Pouli, Antonios Kolocouris, Nikolaos Lougiakis, Graham Ladds

**Affiliations:** † Department of Pharmacology, University of Cambridge, Tennis Court Road, Cambridge CB2 1PD, U.K.; ‡ Laboratory of Medicinal Chemistry, Section of Pharmaceutical Chemistry, Department of Pharmacy, School of Health Sciences, National and Kapodistrian University of Athens, Panepistimiopolis-Zografou 15771, Athens, Greece

## Abstract

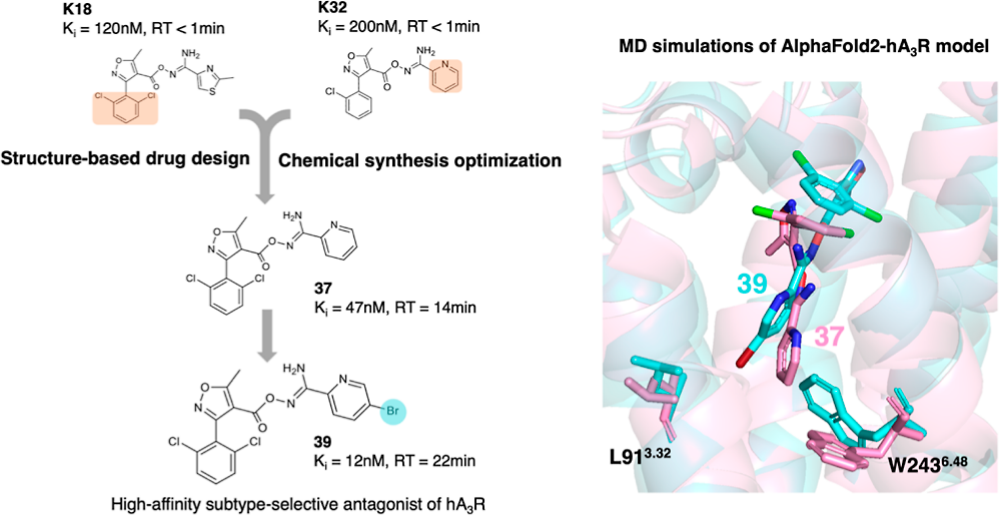

Antagonism of the human adenosine A_3_ receptor (hA_3_R) has potential therapeutic application. Alchemical relative binding free energy calculations of **K18** and **K32** suggested that the combination of a 3-(2,6-dichlorophenyl)-isoxazolyl group with 2-pyridinyl at the ends of a carbonyloxycarboximidamide group should improve hA_3_R affinity. Of the 25 new analogues synthesized, **37** and **74** showed improved hA_3_R affinity compared to **K18** (and **K32**). This was further improved through the addition of a bromine group to the 2-pyridinyl at the 5-position, generating compound **39**. Alchemical relative binding free energy calculations, mutagenesis studies and MD simulations supported the compounds’ binding pattern while suggesting that the bromine of **39** inserts deep into the hA_3_R orthosteric pocket, so highlighting the importance of rigidification of the carbonyloxycarboximidamide moiety. MD simulations highlighted the importance of rigidification of the carbonyloxycarboximidamide, while suggesting that the bromine of **39** inserts deep into the hA_3_R orthosteric pocket, which was supported through mutagenesis studies **39** also selectively antagonized endogenously expressed hA_3_R in nonsmall cell lung carcinoma cells, while pharmacokinetic studies indicated low toxicity enabling in vivo evaluation. We therefore suggest that **39** has potential for further development as a high-affinity hA_3_R antagonist.

## Introduction

Adenosine, a naturally occurring purine nucleoside, is an endogenous agonist of adenosine receptors (ARs).^1^ ARs are G protein-coupled receptors (GPCRs) comprising four subtypes: A_1_, A_2A_, A_2B_, and A_3_. While the A_2A_R and A_2B_R subtypes activate G_s_ to stimulate adenylyl cyclase, increasing 3′,5′-cyclic adenosine monophosphate (cAMP) levels, A_1_R and A_3_R conversely couple to G_i/o_ subunits inhibiting adenylyl cyclase. Beyond inhibiting cAMP accumulation, A_3_R has been suggested to modulate mitogen-activated protein kinase (MAPK) activity which may explain the role of this receptor on cell proliferation and differentiation^2,3^ and in tumor development and progression. Evidence suggests that human A_3_R (hA_3_R) antagonists might become new therapeutic tools for the treatment of both chronic renal disease^4^ and acute renal ischemia and reperfusion injury.^4^ Furthermore, hA_3_R antagonists have demonstrated efficacy in eye pathologies.^2^ Indeed, it has been reported that the potent A_3_R antagonist **MRS1220** (*N*-[9-chloro-2-(2-furanyl)-1,2,4-triazolo[1,5-*c*]quinazolin-5-yl]benzeneacetamide) prevented oligodendrocyte damage and myelin loss triggered by ischemia or by activation of A_3_R in the rat optic nerve.^5^ Hence, blockage of hA_3_R has proven to be useful for the treatment of diverse diseases; however, its role is still to be elucidated in other pathophysiological conditions, such as inflammation, cancer, or pain.^2^ The identification of new potent and selective ligands which can clarify the therapeutic potential arising from blocking or stimulating the hA_3_R remains an attractive objective.^2,6^

Experimental structures have been resolved for all the four subtypes of hARs that have become established drug targets, although hA_3_R only has an active structure reported.^7^ Such experimental structures can help to understand the binding interactions of ARs with ligands and provide templates for structure-based drug design (SBDD) as others^8−14^ and we^15^ have shown. Free energy perturbation coupled with the molecular dynamics simulation (FEP/MD) method^16,17^ has been applied for lead fragment optimization against A_2A_R^18,19^ or for structure–activity relationship (SAR) interpretation, e.g., of 3-deazaadenosine agonists^20^ and thiazolo[5,4-*d*]pyrimidines antagonists^21^ against A_2A_R, or 4-substituted-1,4-dihydrobenzo[4,5]imidazo[1,2-*a*]pyrimidine-3-carboxylate antagonist against A_2B_R.^22^ Furthermore, we have performed the equivalent with FEP/MD thermodynamic integration coupled with the molecular dynamics simulation method (TI/MD)^23^ to describe accurately the structure–affinity relationships of antagonist of human A_1_R (hA_1_R).

**Scheme 1:**
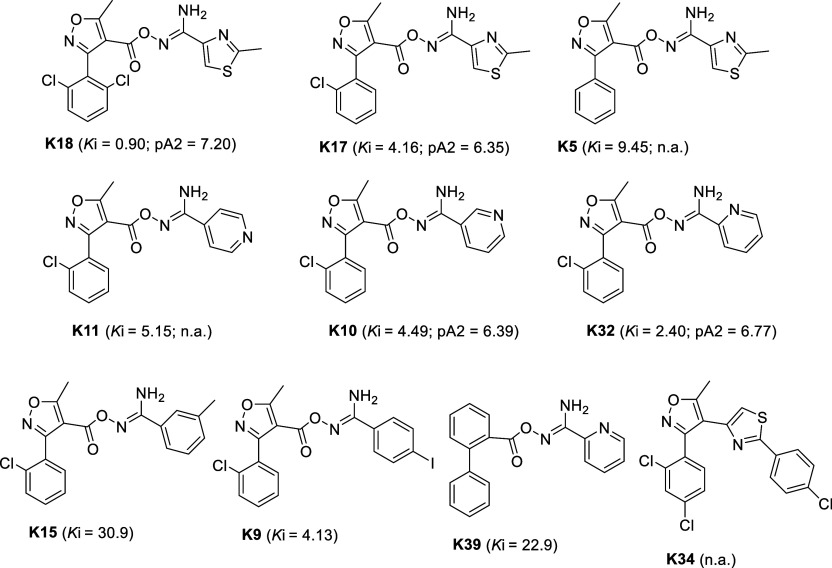
Chemical Structures, Dissociation Constants with Radio-Labeled Assays (*K*_i_ in μΜ), and Antagonistic Potencies (pA_2_) of **K18**/**K32** Analogues Reported in Ref (25); n.a., Not Active

In a previous work, from virtual screening (VS) of an ∼18,000 compound library, we identified hits of different structures as antagonists of ARs with a new structure having low micromolar affinities using radiolabeled assays.^15^ Of particular interest for further development were the hits **K5**, **K9**, **K10**, **K11**, **K15**, **K17**, **K18**, and **K32** (Scheme 1) that are heterocyclic carbonyloxycarboximidamide derivatives.^15,24,25^ These hit compounds (which we purchased from commercial libraries but are synthetically feasible) showed selective low micromolar affinity against hA_3_R using radiolabeled assays, and we showed that the affinities were, in most cases, consistent with antagonistic receptor activities determined using inhibition of cAMP accumulation.^25^

Selecting ligands based on their affinity, an equilibrium parameter, does not necessarily predict in vitro activity, and a ligand’s kinetic properties may provide a better indication of how a ligand will perform in vivo. Kinetic profiling in the drug discovery process allows the resolution of ligand–receptor interactions into both molecular recognition (dependent on association rate constant *K*_on_) and complex stability (dependent on ligand’s dissociation rate constant *K*_off_). Significantly, this enables estimates of the residence time (RT = 1/*K*_off_) of that ligand upon its target.^26^ By testing the compounds shown in Scheme 1, we observed^15,24,25^ that adding chlorine atoms in the phenyl ring of compound **K5** increased affinity and antagonistic potency in **K17** and **K18**. An additional interesting finding was that replacement of the five-membered thiazole ring of compound **K17** with the six-membered pyridine ring maintained the binding affinity in **K10**, **K11**, and **K32**, as well.

Here, we presented a hit-to-lead study through SBDD using TI/MD^27,28^ for the calculation of relative binding free energies and a previous SAR study.^15,24,25^ The accuracy of perturbative binding free energy methods for ligands AR or other class A GPCR systems was previously shown using the FEP/MD^20,22,29−31^ as well as by TI/MD calculations on complexes of A_1_R.^32^ Both of the FEP/MD^33^ and TI/MD^27,28^ methods can provide accurate results for relative binding free energies with a method error of 1 kcal mol^–1^.^28^

The SBDD and synthesis led to 25 new compounds (**37**–**61**) which we tested for their affinity at hA_3_R using nanoluciferease-based bioluminescence resonance energy transfer (NanoBRET) binding assay. Among these, seven compounds **37**, **39**, **40**, **47**, **48**, **59**, and **60** displayed similar or significantly higher affinity than **K18**. Their binding kinetic parameters including *K*_on_, *K*_off_, and RT were determined, as well as the hAR subtype selectivity. Compounds **37** and **39** showed ∼10-fold increased potency against hA_3_R and ∼20-fold higher RT compared to **K18** or **K32**. Based on **37** and **39**, we explored the tolerance of the chlorine atoms in the 2,6-dichlorophenyl group by synthesizing and testing 4 more new analogues (**74**–**77**) with bromine or methyl groups instead and found that **39** still showed the highest affinity and selectivity toward hA_3_R. We then applied the TI/MD method^27^ to confirm quantitatively with an accurate method the observed structure–affinity relationships. We performed 500 ns MD simulations of the representative compounds **37**–**39**, **56**–**57**, and **60** to describe the interactions with residues in the orthosteric binding pocket and conducted an exhaustive analysis with *in vitro* mutagenesis experimentation for our lead compound **39** in comparison with **37** that also informs for SARs^26,41^ Further, since hA_3_R has often been referenced as a promising target for treating cancer,^34^ we explored the ability of **37** and **39** to selectively antagonize hA_3_R in a nonsmall cell lung cancer cell line that endogenously expresses all 4 AR subtypes.^35^ The ability of **39** to inhibit cancer cell proliferation led us to perform a preliminary pharmacokinetic study, which displayed good lipophilicity and permeability across intestinal cells but a relatively low aqueous solubility and metabolic stability. We therefore present the high-affinity hA_3_R antagonist **39** as a new lead compound for future development.

## Results

### Structure-Based Drug Design

**Figure 1:**
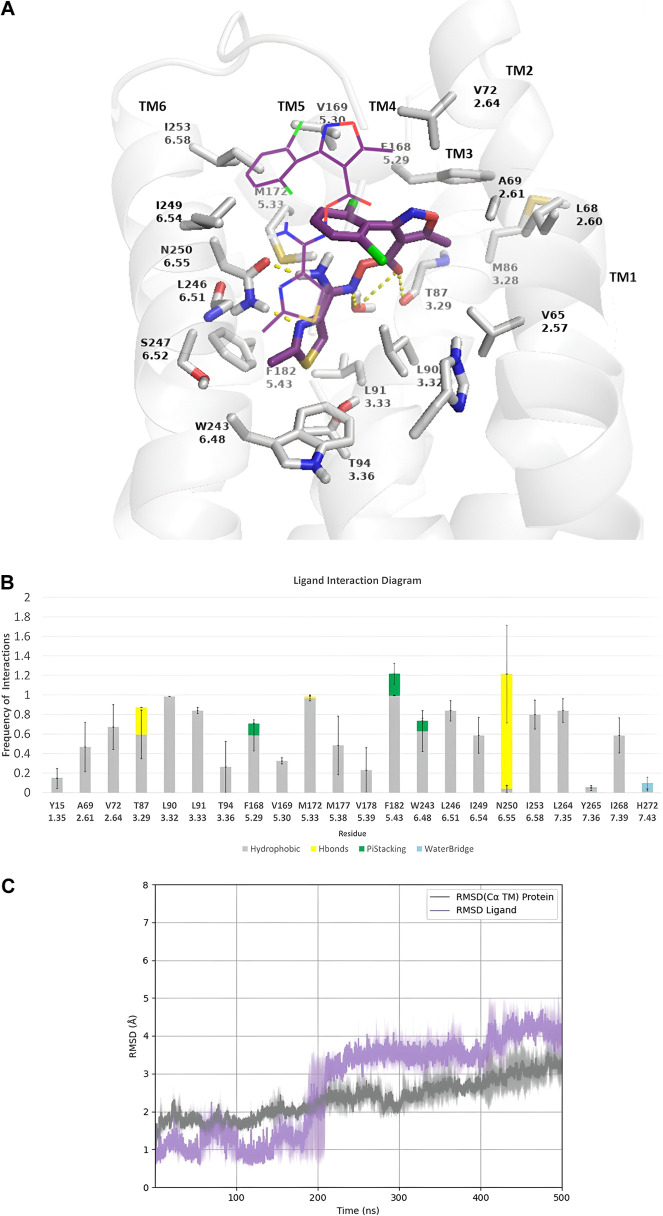
500 ns MD simulations for the complex of compound **K18** with the wild-type (WT) hA_3_R using the amber ff19sb.^39^ (A) Representative frame of **K18** inside the orthosteric binding area; (B) receptor–ligand interaction frequency histograms; bars are plotted only for residues with interaction frequencies ≥0.2. Color figure in frames or bar plots: ligand is shown with pink sticks and ligand’s starting position with a pink wire, receptor is shown with a white cartoon and sticks, hydrogen bonding interactions are shown with yellow dashes or bars, π–π interactions are shown with green dashes or bars, hydrophobic interactions are shown with gray bars, and water bridges are shown with blue bars. (C) Root-mean-square deviation (rmsd) plots of Ca carbons of the protein (gray line) and of heavy atoms of the ligand (magenta line). For MD simulations, we used a revised model of the inactive form of hA_3_R we have recently published,^40^ generated using the multistate Alphafold 2(AF2) method^41,42^ of hA_3_R generated from GPCRdb web-tool;^43^ the complexes of the starting structure (docking pose) and final snapshot from the MD simulations are available as pdb files (see https://github.com/annachor/inactive_A3R_AF2-carbonyloxycarboximidamides_MDs).

Previously using mutagenesis experiments, and MD simulations using the amber14sb force field (ff14sb)^36^ or OPLS2005^37^ with Poisson–Boltzmann or generalized Born and surface area continuum solvation (MM/PBSA^38^ MM/GBSA calculations^38^) binding free energy calculations,^24,25^ we have suggested a preferred binding pose for **K18** or **K17** bearing the 1,3-thiazolyl and 2,6-dichlorophenyl or 2-chlorophenyl groups, respectively, and **K32** (compound **42** in this study) having the 2-pyridinyl and 2-chlorophenyl groups at the two ends of the carbonyloxycarboximidamide linker. Τhe binding pose (after 500 ns MD simulations with ff19sb,^39^) for compound **K18** is shown in Figure 1A.

In this binding pose, the dichlorophenyl group in **K18** is oriented toward transmembrane (TM) 5 and TM6, instead of TM1 and TM2, thus interacting with V169^5.30^ and I249^6.54^. This preference was increased with the number of chlorine atoms (as is reflected by the binding affinity constants of the compounds **K18**, **K17**, and **K5**(15,24,25) (Scheme 1). Compared to 1,3-thiazolyl in **K17**, the more basic 2-pyridinyl group^44^ in **K32** could form a stronger hydrogen bonding interaction with N250^6.55^ leading to a ∼2-fold higher affinity of **K32**.^15,24,25^ Replacement of isoxazole in **K5** with a phenyl group in **K39** reduced the binding affinity and replacement of thiazolyl or pyridinyl in **K17** or **K10**, **K11**, and **K32** by phenyl in **K15** also reduce their binding affinity.^24,25^

**Scheme 2:**
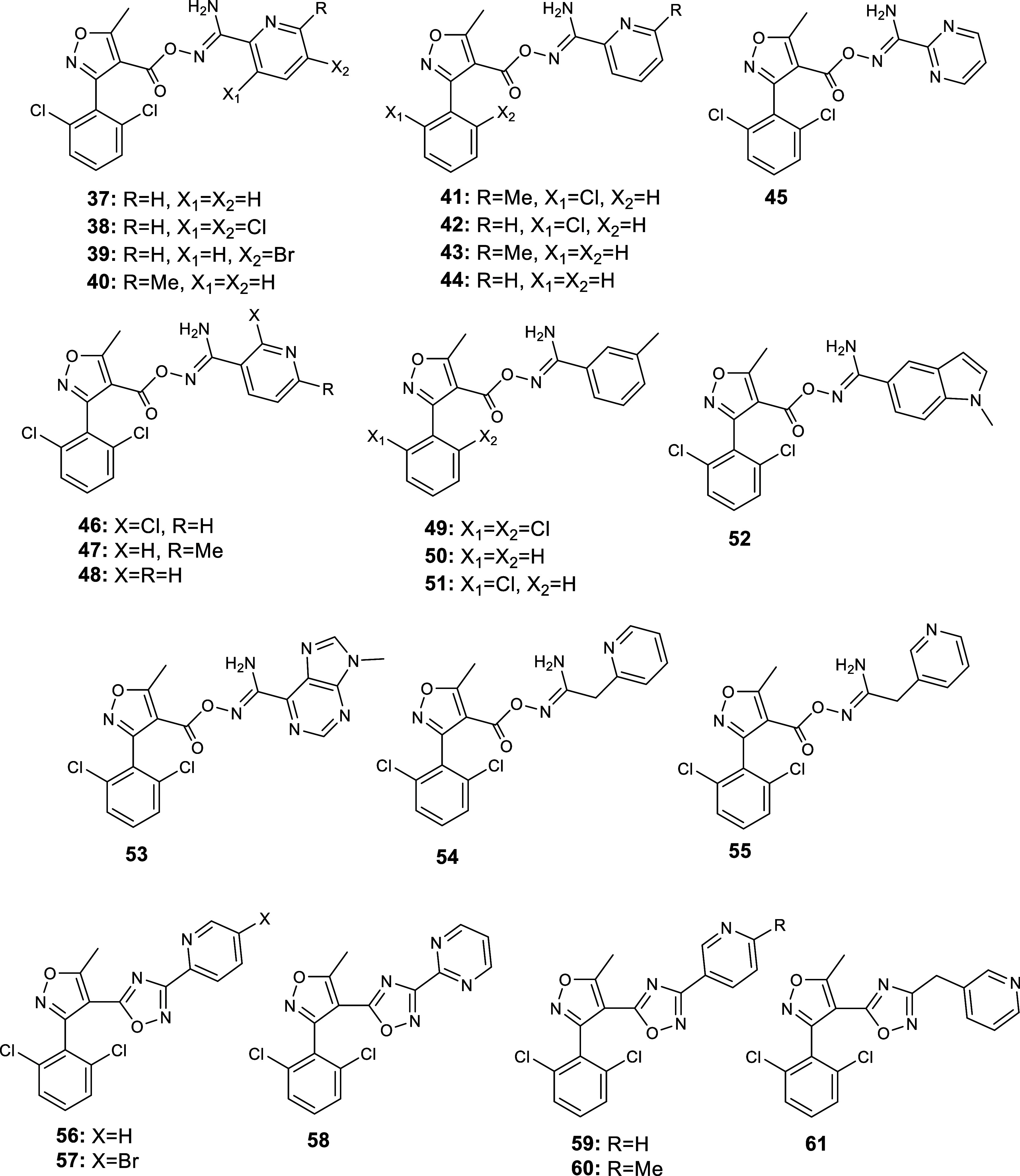
Chemical Structure of the 19 Heterocyclic Carbonyloxycarboximidamides **37–55** and of the 6 Cyclic Derivatives **56–61** Designed and Synthesized in the Hit-to-Lead Optimization as hA_3_R Antagonists Based on the Binding Profile of **K18**/**K32**^15,24,25^

Based on these observations, we assumed that in compound **37** (Scheme 2), the combination of the 2,6-dichlorophenyl and the 2-pyridinyl groups at the ends of the carbonyloxycarboximidamide linker would enhance affinity. To quantify these predictions using SBDD, we performed TI/MD simulations with ff14sb^39^ in heterocyclic carbonyloxycarboximidamide–hA_3_R complexes embedded in 1-palmitoyl-2-oleoyl-*sn*-glycero-3-phosphocholine (POPC) bilayers. We used for the MD simulations a revised model of the inactive hA_3_R generated based on the multistate AF2 method^41,42^ that we recently published.^40^ Interestingly, in the experimental structure of the active hA_3_R (PDB IDs 8X16, 8X17(7)), the orientation of the M172^5.33^, R173,^5.34^ and M174^5.34^ motifs, which we showed in ref (40) as important for the residence time of antagonists, matches the conformation we adopted in our revised^40^ multi-AF2-based model for the inactive hA_3_R (Figure S1), which we used for all the MD simulations in the present work. We performed the calculations using a 1-step protocol which changes the partial charges and the van der Waals interactions in a single simulation by activating both Lennard-Jones and Coulomb softcore potentials simultaneously.

Indeed, our TI/MD calculations suggested that the alchemical transformation **K18** → **37**, where 1,3-thiazolyl is changed to 2-pyridinyl, was favored by a relative binding free energy (ΔΔ*G*_b,TI/MD_) equal to −0.77 ± 0.08 kcal mol^–1^. Also, the alchemical transformation **44** → **37** that changes the 2-chlorophenyl to 2,6-dichlorophenyl was favored by ΔΔ*G*_b,TI/MD_ = −1.83 ± 0.06 kcal mol^–1^. A striking observation from our previous experiments^25^ demonstrated that mutation of L90^3.32^ in the low region of the receptor area, which makes a direct interaction with **K18**, and the remote L264^7.35^ in the middle/upper region (both to alanine) increased the antagonistic affinity of **K18**. This suggests an available empty space in these regions of the orthosteric area that can be filled with a sizable hydrophobic group for increasing the ligand’s affinity. Based on the commercially available synthetic fragments, we tested the addition of a bromine atom at the 5-position of the 2-pyridinyl group in **37** so that it could fit into the bottom area of the receptor. Thus, using TI/MD calculations, we observed that the alchemical transformation **37** → **39** where a bromine group was added was favored by ΔΔ*G*_b,TI/MD_ = −0.67 ± 0.04 kcal mol^–1^. However, the addition of two chlorine atoms at 3,5-positions of the 2-pyridinyl group in **37** was not favored since for the alchemical transformation **37** → **38**, we calculated ΔΔ*G*_b,TI/MD_ = 0.58 ± 0.04 kcal mol^–1^.

We performed additional TI/MD calculations to determine whether replacement of the 2-pyridinyl group in compound **37** by other groups could further improve affinity. For this purpose, we decided to introduce a methyl group at position 6- of the pyridine ring of **37** to obtain **40**, or transfer its nitrogen atom to obtain the 3-pyridinyl analogues **46**–**48**. In addition, we replaced the pyridine ring with the pyrimidin-2-yl moiety to obtain derivative **45**, with the 3-tolyl group to obtain compound **49** or with a bulkier, nitrogen-containing, bicyclic system such as the indole or the purine ring, leading to compounds **52** and **53**, respectively. Finally, we introduced a methylene linker between the carbonyloxycarboximidamide moiety and the pyridine ring to obtain analogues **54** and **55**. In order to establish our previous observations,^15,24,25^ with compounds **K5**, **K17**, and **K18**, which suggested that by increasing the number of chlorine atoms on the phenyl ring of the isoxazole group the binding activity was increased, we decided to study selected analogues bearing one chlorine atom or unsubstituted on this phenyl ring (compounds **41**–**44** and **50**–**51**). Thus, we performed TI/MD binding free energy calculations for most of the above-mentioned alchemical transformations (Table S1). Even when the alchemical calculations did not suggest improved binding to hA_3_R, we still felt that their synthesis was required since it would provide further experimental validation (using in vitro pharmacological techniques) of our model. We therefore synthesized compounds **37**–**55** (Scheme 2).

Furthermore, in compounds **56**–**61** (Scheme 2), we sought to investigate the effect on the antagonistic activity caused by the incorporation of the carbonyloxycarboximidamide pharmacophore between the 2,6-dichlorophenyl isoxazole and the aromatic nitrogen heterocyclic ring, into a rigid 1,2,4-oxadiazole ring. This transformation might be achieved through ring closure of the corresponding carbonyloxycarboximidamide analogues. The 500 ns MD simulations with ff19sb^39^ suggested that compound **56**, the cyclic analogue of compound **37**, is stabilized inside the orthosteric binding area through hydrogen bonding interactions between the amide side chain of N250^6.55^ and the pyridinyl and 4-oxadiazolyl nitrogen atoms.

### Chemical Synthesis

**Scheme 3:**
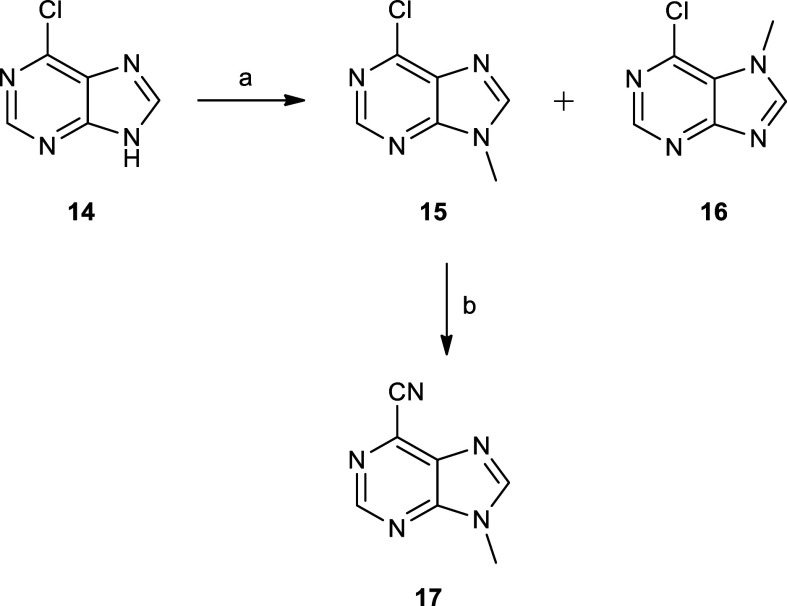
Preparation of Carbonitrile **17** Reagents and conditions: (a) (i) 1.4 equiv of NaH, DMF dry, 0 °C, 1 h, (ii) 1.5 equiv of iodomethane, room temperature (rt), 20 h, 58% for **15** and (b) 0.11 equiv of Zn, 0.6 equiv of Zn(CN)_2_, 0.02 equiv of Pd_2_(dba)_3_, 0.04 equiv of dppf, DMA, reflux, 3 h, 79%.

For the synthesis of the target derivatives, a number of commercially available aryl or aralkyl carbonitriles were used, namely, pyridine-2-carbonitrile (**1**), 3,5-dichloropyridine-2-carbonitrile (**2**), 5-bromopyridine-2-carbonitrile (**3**), 6-methylpyridine-2-carbonitrile (**4**), pyrimidine-2-carbonitrile (**5**), 2-chloropyridine-3-carbonitrile (**6**), 6-methylpyridine-3-carbonitrile (**7**), pyridine-3-carbonitrile (**8**), 3-methylbenzolocarbonitrile (**9**), 2-(pyridin-2-yl)acetonitrile (**10**), 2-(pyridin-3-yl)acetonitrile (**11**), as well as 1-methyl-1*H*-indole-5-carbonitrile (**13**) that was prepared according to a published procedure upon methylation of 1*H*-indole-5-carbonitrile (**12**)^45^ and 9-methyl-9*H*-purine-6-carbonitrile (**17**), which was prepared from 6-chloropurine (**14**) by means of tris(dibenzylideneacetone)dipalladium(0) [Pd_2_(dba)_3_)] and 1,1′-ferrocenediyl-bis(diphenylphosphine) [dppf] in dimethylacetamide (DMA) as depicted in Scheme 3.

**Scheme 4:**
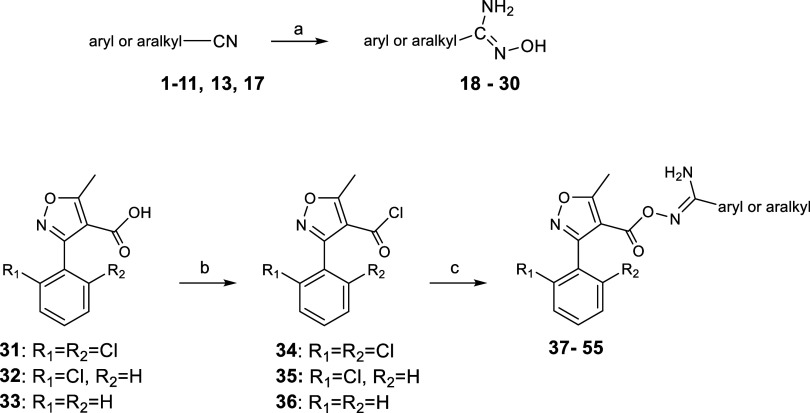
Preparation of Carbonyloxycarboximidamides **37–55** Reagents and conditions: (a) 2.9 equiv of HONH_2_ (50 wt % solution in water), EtOH, reflux, 2 h or 1.5 equiv of HONH_2_·HCl, 1.5 equiv of NaHCO_3_, EtOH, rt 90 min and then reflux 2 h, 67–96%; (b) SOCl_2_, reflux, 3 h; and (c) 1 equiv of amidoxime **18**–**30**, 1.1 equiv of Et_3_N, THF dry, rt, 2–16 h, 70–90% (for 2 steps).

All the above-mentioned aryl or aralkyl carbonitriles were treated with hydroxylamine hydrochloride in the presence of a base or aqueous hydroxylamine and were converted to the aryl amidoximes **18**–**30** (Scheme 4Table S2). The amidoximes reacted with the acyl chlorides **34**–**36**, easily prepared from the commercial carboxylic acids **31**–**33**, respectively, to result in the corresponding target derivatives **37**–**55**.

**Scheme 5:**
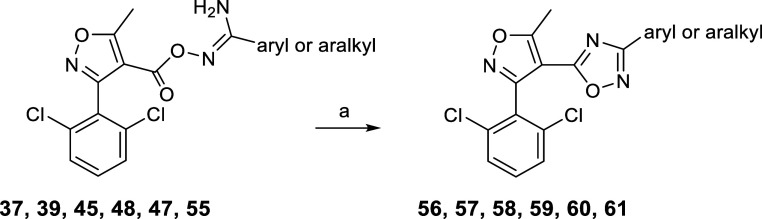
Preparation of 1,2,4-Oxadiazole Ring Closed Analogues **56–61** Reagents and conditions: (a) 1 equiv of KOH, DMSO dry, rt, 30–45 min, 81–93%.

Finally, the target compounds for which the acyloxy imidamide moiety has been incorporated into an 1,2,4-oxadiazole ring (**56**, **57**, **58**, **59**, **60**, and **61**) were prepared upon treatment of the derivatives **37**, **39**, **45**, **48**, **47**, and **55**, respectively, with potassium hydroxide in anhydrous dimethyl sulfoxide (DMSO), through an intramolecular ring closure reaction, followed by dehydration of the resulting intermediate (Scheme 5).

### In Vitro Pharmacological Characterization

#### Quantifying the Binding Affinity and Kinetics of Potential Antagonists at hA_3_R

We have previously reported the characterization of hit compound **K18**, a specific hA_3_R (*K*_i_ < 1 μM) competitive antagonist with a new scaffold as hA_3_R ligand.^15,25^ Based on this scaffold, 25 analogues were synthesized to further investigate the SAR, aiming to develop A_3_R antagonists with high affinity as well as high specificity. The compounds were grouped A–D based on their backbone (Tables 1, S3).

**Table 1:**
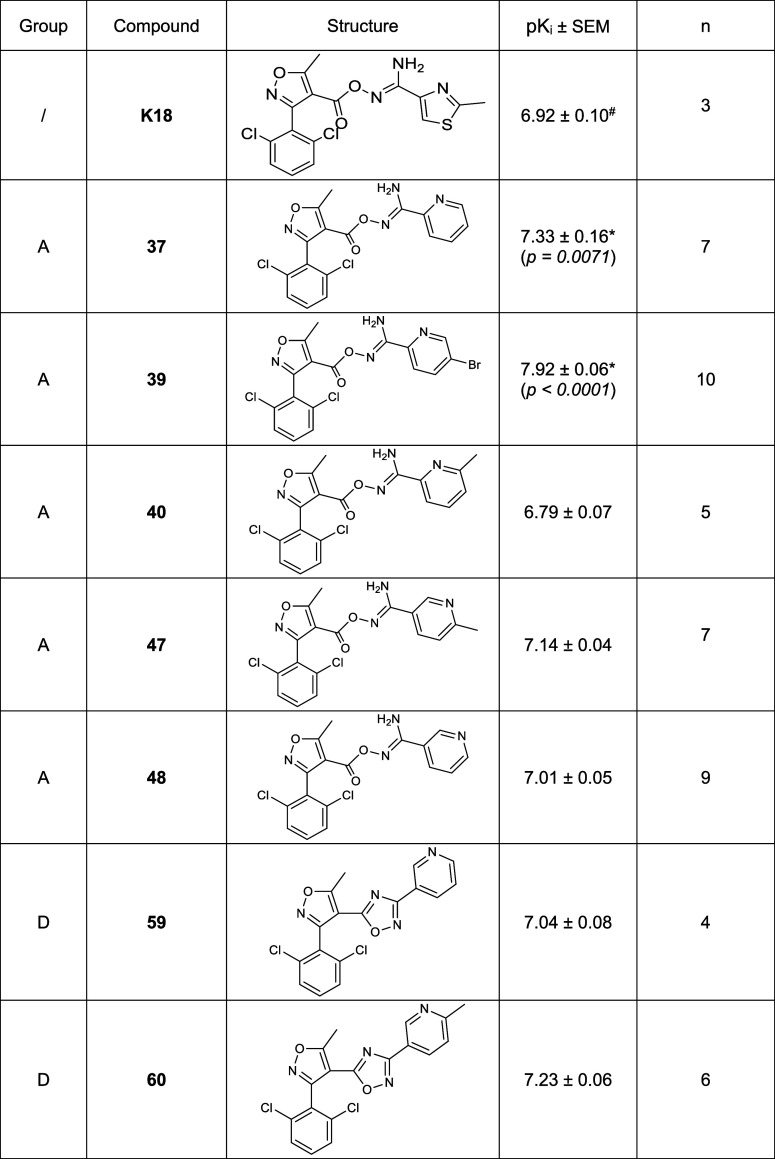
Chemical Structure and Binding Affinity (pK_i_^a^) of the Seven Novel Heterocyclic Compounds, Carbonyloxycarboximidamides (Group A) or 1,2,4-Oxadiazole Derivatives (Group D), which Displayed Equal or Increased Affinity to Their Precursor K18

a All the equilibrium binding affinities (p*K*_i_) were determined with NanoBRET ligand binding assay and represented as mean ± standard error of the mean (SEM) of n independent repeats with experiment conducted in duplicates. Data of **K18** was taken from ref (25). One-way ANOVA with Dunnett’s post-test was used to determine the statistical significance (*p < 0.05) compared to the p*K*_i_ of **K18**.

**Figure 2:**
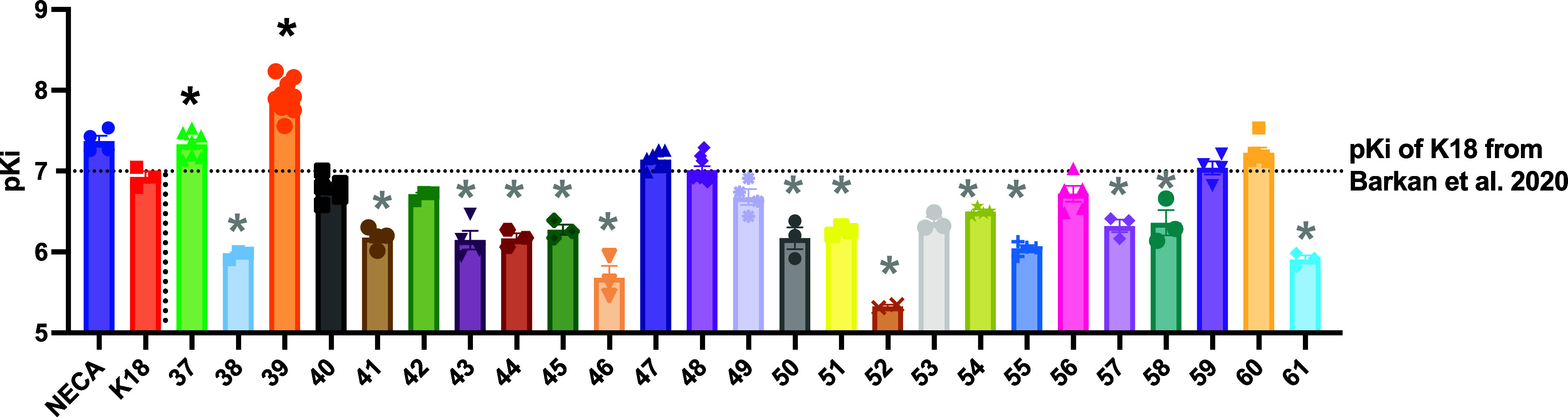
Binding affinity (p*K*_i_) of 25 heterocyclic carbonyloxycarboximidamide analogues or derivatives at hA_3_R determined in NanoBRET binding assay. 5 nM CA200645 was added to HEK293 cells stably expressing Nluc-hA_3_R, interacting with Nluc and produce BRET signal. The BRET ratio values were baseline-corrected with the response induced by high-concentration (1 μM) A_3_R antagonist MRS1220. Each data point represents the mean ± SEM of at least three experiments performed in duplicates. The p*K*_i_ values determined were compared with the p*K*_i_ of K18 previously determined in ref (25). One-way ANOVA with Dunnett’s post-test was used to determine the statistical significance (*p < 0.05) compared to the p*K*_i_ of K18 with black * indicating the affinity significantly higher and the gray * indicating the significantly lower one.

To assess the binding affinities of these compounds at hA_3_R, NanoBRET-based ligand binding assays were used in a human embryonic kidney 293T (HEK293T) cell line stably expressed nanoluciferase (Nluc)-tagged hA_3_R. The saturation binding affinity (p*K*_i_) was determined by using nonfluorescent compounds to displace 5 nM fluorescent A_3_R antagonist **CA200645** at Nluc-hA_3_R. According to the p*K*_i_ values determined (Figure 2 and Tables 1 and S3), there were two out of the 25 compounds that displayed significantly higher affinity, (**37**, p*K*_i_ = 7.33 ± 0.16, *p* = 0.0071; and **39**, p*K*_i_ = 7.92 ± 0.06, *p* < 0.0001) to hA_3_R compared to the reference compound **K18** (p*K*_i_ = 6.92 ± 0.10, K_i_ ∼ 120 nM^25^), while five others showed near-equal affinity, **40** (p*K*_i_ = 6.79 ± 0.11), **47** (p*K*_i_ = 7.19 ± 0.05), **48** (p*K*_i_ = 6.99 ± 0.04), **59** (p*K*_i_ = 7.11 ± 0.05), and **60** (p*K*_i_ = 7.23 ± 0.06) to **K18**. Among these seven compounds, **39** showed the highest affinity (*K*_i_ = 12.0 nM), which is about 10-fold higher than compound **K18**, followed by compound **37** (*K*_i_ = 46.8 nM), and the oxadiazole derivatives **60** (*K*_i_ = 58.9 nM) and **59** (*K*_i_ = 91.2 nM). Since we were aiming to develop hA_3_R-selective antagonists with equivalent or higher affinity to **K18**, only these seven compounds were selected for further investigation.

As we^25^ and others^46^ previously showed, NanoBRET ligand binding assay can also be used to determine the parameters of the ligand binding kinetics. We have previously determined the binding kinetic parameters of fluorescent ligand **CA200645** with *K*_on_(*k*_1_) = 3.25 ± 0.03 × 10^6^ M^–1^ min^–1^ and *K*_off_(*k*_2_) = 0.019 ± 0.003 min^–1^.^25^ Using the “kinetics of competitive binding” model (built in Graphpad prism 9.3.1), we determined the *K*_on_(*k*_3_) and *K*_off_(*k*_4_) of the new seven compounds (Table 2). From these parameters, the RT was determined as 1/*K*_off_ (Table 2). As shown in Table 2, among these seven compounds, **39** showed largest *K*_on_ (5.95 ± 0.42 M^–1^ min^–1^) and the smallest *K*_off_ (0.046 ± 0.002 min^–1^) and therefore longest residence time (22.1 ± 1.0 min), which contributed to its high binding affinities.

**Table 2:** Kinetic Parameters of Binding for the Seven High-Affinity Novel K18 Derivatives at hA_3_R^a^

compound	*K*_on_(*k*_3_) (x10^6^)/M^–1^ min^–1^	*K*_off_(*k*_4_)/min^–1^	RT/min
**37**	1.60 ± 0.34	0.075 ± 0.002	13.5 ± 0.4
**39**	5.95 ± 0.42	0.046 ± 0.002	22.1 ± 1.0
**40**	0.23 ± 0.05	0.057 ± 0.001	17.5 ± 0.2
**47**	1.49 ± 0.44	0.049 ± 0.004	21.3 ± 2.2
**48**	1.01 ± 0.20	0.054 ± 0.003	14.7 ± 3.4
**59**	0.65 ± 0.07	0.050 ± 0.004	20.5 ± 1.9
**60**	0.35 ± 0.06	0.102 ± 0.015	10.7 ± 2.0
**MRS1220**	325 ± 2.8^b^	0.025 ± 0.005^b^	40.32^b^

a *K*_on_(*k*_3_) and *K*_off_(*k*_4_) for each compound determined using NanoBRET binding assay at Nluc-hA_3_R and fitted with the “kinetics of competitive binding model”. RT as determined by 1/K_off_.

b Indicated values from ref (25).

#### Seven High-Affinity Antagonists Displayed High Selectivity for hA_3_R over the Other AR Subtypes

**Figure 3:**
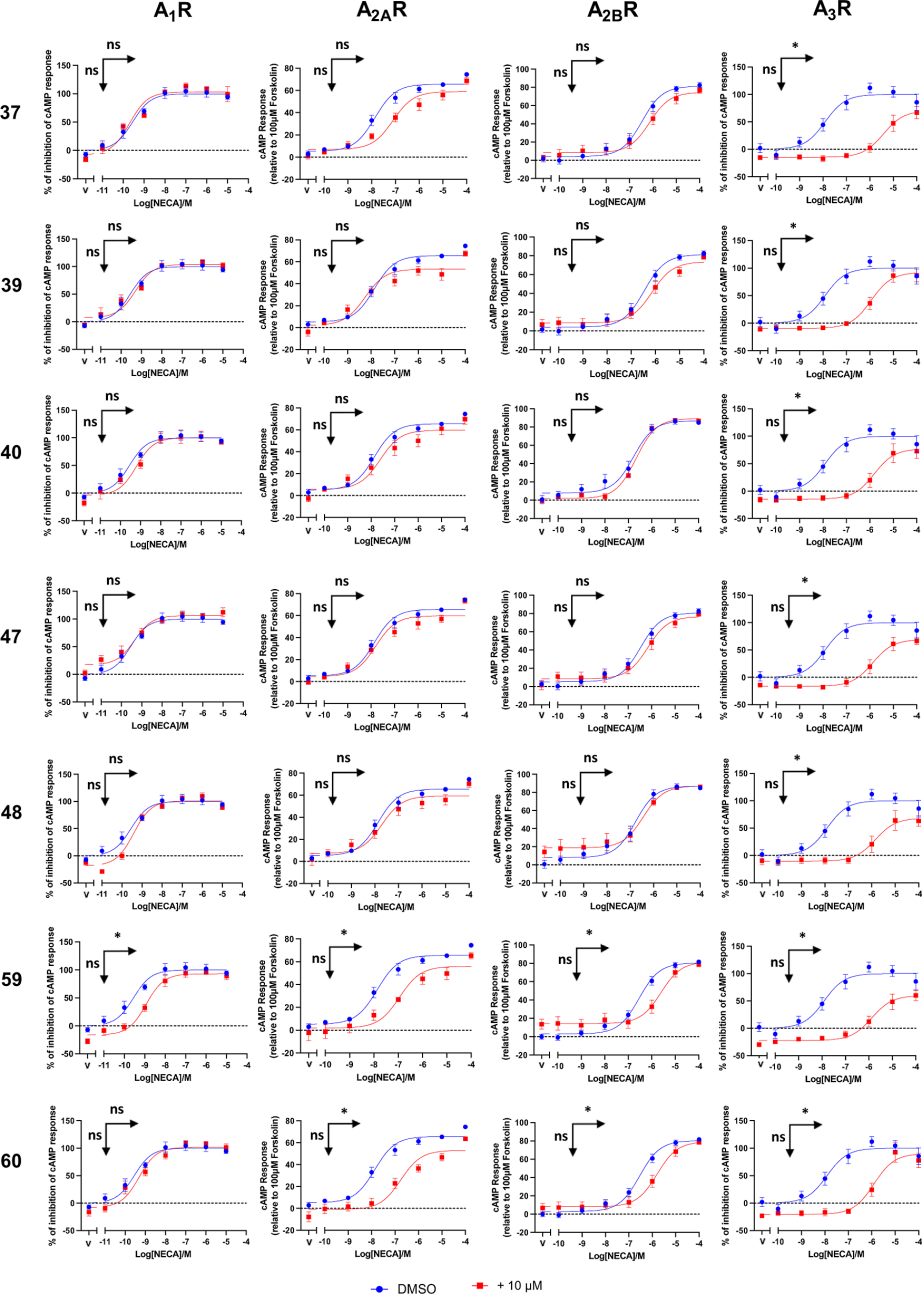
Characterization of the seven selected compounds at all human AR subtypes in cAMP accumulation assay. CHO-K1 cells stably expressing individual AR subtypes were treated with different concentrations of **NECA** or vehicle (V) and 1 μM **forskolin** in the case of G_i/o_-coupled hA_1_R and hA_3_R or DMSO control in the case of G_s_-coupled hA_2A_R and hA_2B_R, as well as 10 μM test compound (red) or DMSO control (blue) for 30 min. In hA_2A_R and hA_2B_R, cAMP response was normalized against the response induced by 100 μM **forskolin**, whereas in hA_1_R and hA_3_R, responses were represented as the percentage of the inhibition of cAMP response generated by 100 μM **forskolin**. Vertical arrow and horizontal arrow denote the significance of the change in efficacy and potency, respectively. One-way ANOVA was performed to compare the changes between DMSO only and the presence of tested compound (*p < 0.05). All values are represented as mean ± standard error of the mean (SEM), obtained in *n* = 3 independent experimental repeats, conducted in duplicates.

To assess the competitive antagonistic action of the seven most potent compounds **37**, **39**, **40**, **47**, **48**, **59**, and **60** at all human AR subtypes, cAMP accumulation assays were performed in CHO-K1 cells stably expressing the individual receptors, hA_1_R, hA_2A_R, hA_2B_R, or hA_3_R. Increasing concentrations of the nonselective AR agonist 5′-*N*-ethylcarboxamidoadenosine (**NECA**) and 10 μM antagonist or DMSO control were coincubated for 30 min. Also, for the G_i/o_-coupled hA_1_R and hA_3_R, 1 μM **forskolin** (a plant toxin that activates adenylyl cyclase independent of the G protein) was added to stimulate cAMP production. For the G_s_-coupled hA_2A_R and hA_2B_R, **forskolin** was not added since the receptors are able to stimulate cAMP accumulation alone. As shown in Figure 3 and Table S4, among these 7 compounds, **37**, **39**, **40**, **47**, and **48** selectively antagonize hA_3_R but not the other AR subtypes. Apart from strong potency at hA_3_R, **60** showed weak antagonism at hA_2A_R and hA_2B_R, while **59** had weak antagonistic effects at hA_1_R, hA_2A_R, and hA_2B_R.

For the most potent compound **39**, different concentrations of the antagonist were used at hA_3_R to perform a full Schild regression analysis (Figure S2). From this analysis, the resulted estimation of antagonist affinity (pA_2_) was 7.95 ± 0.15, and the Schild slope was found to be close to unity, indicating that **39** acts as a competitive antagonist at hA_3_R.

#### Further Exploration of the Role of the 2,6-Dichlorophenyl Group in the Binding Affinity toward A_3_R

**Scheme 6:**
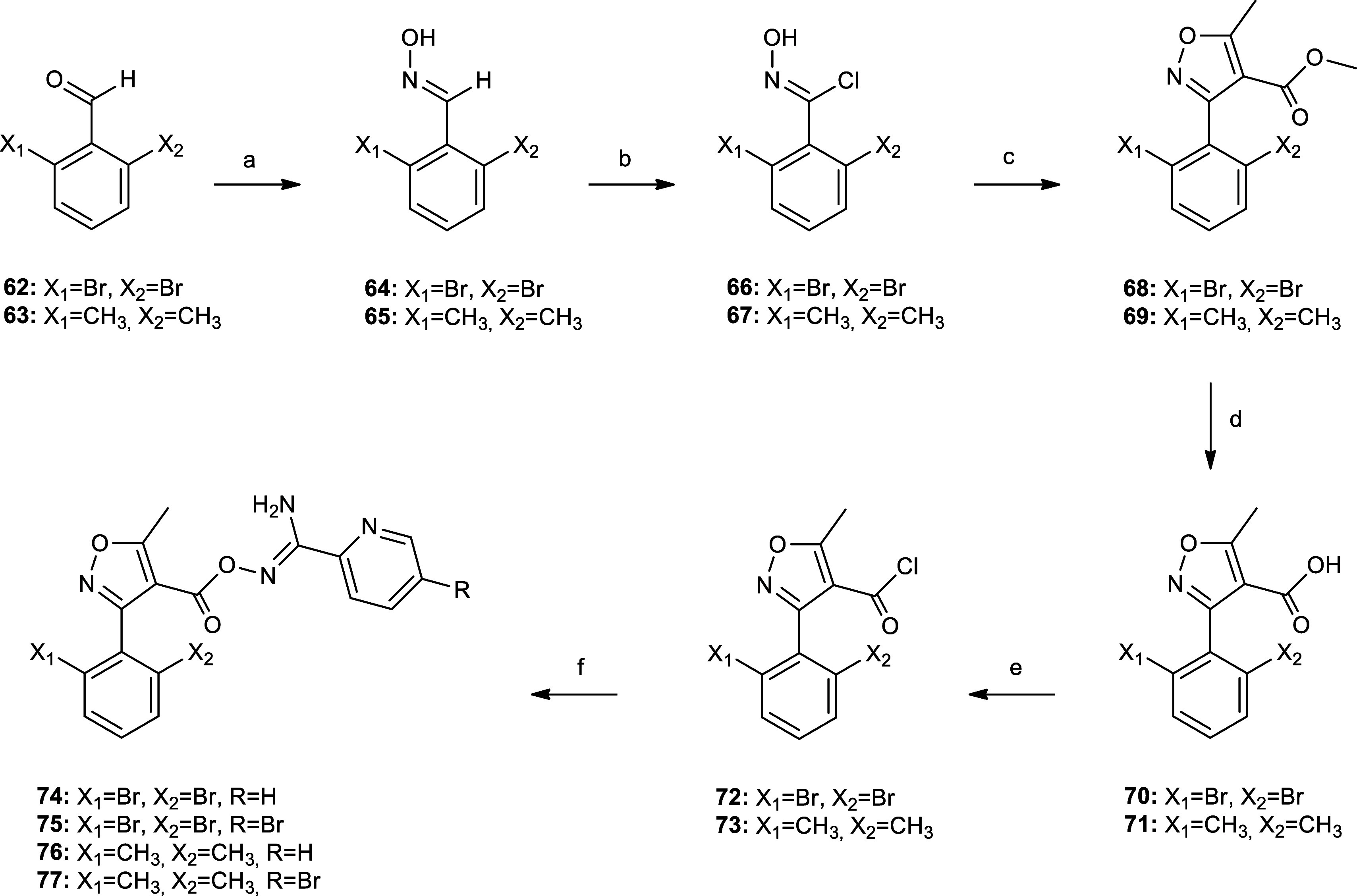
Preparation of 2,6-Dibromo and 2,6-Dimethylphenylisoxazole Analogues **74–77** Reagents and conditions: (a) 1.15 equiv of NH_2_OH·HCl, 1.15 equiv of NaOH, EtOH, reflux, 2 h, 90–95%; (b) 1 equiv of NCS, DMF dry, rt, 2 h, 61–84%; (c) 1 equiv of methyl acetoacetate, 1 equiv of MeONa, MeOH, rt, 16 h, 70–72%; (d) 1.2 equiv of NaOH, MeOH·H_2_O, 65 °C, 3 h, 77–83%; (e) SOCl_2_, reflux, 3 h; and (f) 1 equiv of amidoxime **18** (for **74** and **76**) or 1 equiv of amidoxime **20** (for **75** and **77**), 1.1 equiv of Et_3_N, THF dry, rt, 2 h, 90–97% (for 2 steps).

All seven high-affinity antagonists shared a common structure of the 2,6-dichlorophenyl group at position 3 of the isoxazole ring, and the decrease of the number of chloro-substituents leads to reduction in binding affinity (compounds of groups B and C, Table S3). Therefore, we further explored the importance of these two chloro-groups by synthesizing the structural analogues of our most active lead compounds **37** and **39**, where the two chloro-groups were replaced by either bromo- or methyl-groups, leading to the corresponding derivatives **74**–**77** (Scheme 6). The results from TI/MD calculations for the alchemical transformations **37** → **74** or **37** → **76** that change 2,6-dichlorophenyl to 2,6-dibromorophenyl or 2,6-dimethylphenyl were ΔΔ*G*_b,TI/MD_ = −0.53 ± 0.04 or −0.64 ± 0.05 kcal mol^–1^, respectively. The corresponding alchemical transformations for **39**, **39** → **75**, or **39** → **77** were ΔΔ*G*_b,TI/MD_ = −0.45 ± 0.04 or −0.46 ± 0.04 kcal mol^–1^, respectively. The calculations suggested a possible small improvement in binding affinity, although the error of the method is 1 kcal mol^–1^ which corresponds to an ∼5-fold difference in binding affinity.

Nevertheless, we synthesized compounds **74**–**77** (Scheme 6) and then characterized these 4 new compounds using NanoBRET ligand binding assay at hA_3_R to determine both their affinities and kinetic parameters (Tables 3 and 4). The synthesis of the novel derivatives **74**–**77** was performed starting from commercially available 2,6-disubstituted benzaldehydes **62** and **63** that were converted to the isoxazole methyl esters **68** and **69**, respectively, upon oxime formation, chlorination with *N*-chlorosuccinimide (NCS), and subsequent ring closure of intermediates **66** and **67** with methyl acetoacetate.^47^ The methyl esters **68** and **69** were hydrolyzed under basic conditions to afford the carboxylic acids **70** and **71** that were converted to the corresponding acyl chlorides **72** and **73**. Finally, the latter were coupled with amidoximes **18** and **20** to afford the target derivatives **74**–**77** (Scheme 6). The substitution to bromine from chlorine significantly increased the affinity of **37** but reduced the affinity of **39**, whereas the substitution to the methyl group reduced the affinities of both compounds significantly. However, these compounds still displayed equal or higher affinity when compared with **K18**. Also, we employed cAMP accumulation assay to study their subtype selectivity. These four compounds maintain the hA_3_R specificity observed for their precursors **37** and **39** (Table S5 and Figure S3), all failing to antagonize hA_1_R, hA_2A_R, and hA_2B_R. Finally, interspecies differences of adenosine A_3_R are higher than the other ARs, and this results in the difficulties in developing A_3_R antagonists which have cross-species activity.^48^ For completeness, we examined the affinity of the 11 compounds **37**, **39**, **40**, **47**, **48**, **59**, **60**, **74**, **75**, **76**, and **77** at rat A_3_R (rA_3_R) using the NanoBRET ligand binding assays using Nluc-rA_3_R. None of the compounds show affinity *K*_i_ > 1 μM at Nluc-rA_3_R, suggesting that all are species selective (Figure S4).

**Table 3:**
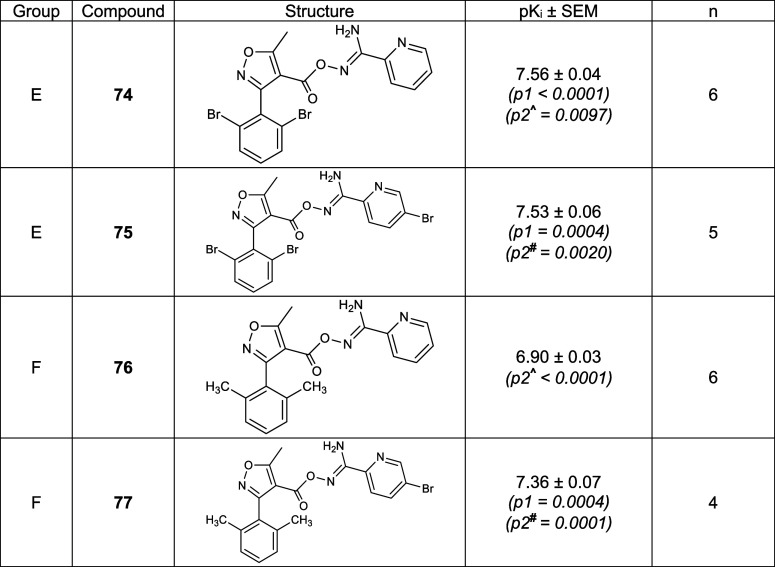
Chemical Structure and Binding Affinity of the Four Analogues Based on **37** and **39**

All the equilibrium binding affinities (p*K*_i_) were determined using NanoBRET binding assay and represented as mean ± SEM of *n* independent repeats with experiment conducted in duplicates. Data of **K18** was taken from ref (25). One-way ANOVA with Dunnett’s post-test was used to determine the statistical significance (*p < 0.05) compared to the p*K*_i_ of **K18** (p1) and **37**^**#**^ or **39**^**∧**^ (p2) as appropriate.

**Table 4:** Kinetics of Binding at Human A_3_R for Four High-Affinity Compounds **74–77**^a^

compound	*K*_on_(*k*_3_) (x10^6^)/M^–1^ min^–1^	*K*_off_ (*k*_4_)/min^–1^	RT/min
**74**	3.83 ± 0.54	0.092 ± 0.006	11.0 ± 0.7
**75**	2.41 ± 0.26	0.057 ± 0.006	18.4 ± 2.1
**76**	1.12 ± 0.23	0.081 ± 0.007	12.6 ± 1.1
**77**	1.51 ± 0.28	0.066 ± 0.002	15.1 ± 0.4

a K_on_(*k*_3_) and *K*_off_(*k*_4_) for each compound determined using NanoBRET binding assay at Nluc-hA_3_R and fitted with the “kinetics of competitive binding model”. RT as determined by 1/*K*_off_.

As summarized in Table 5, the affinities of these 11 compounds determined from (a) the equilibrium binding affinity measured in NanoBRET assay (p*K*_i_), (b) the kinetic dissociation constant (p*K*_d_), and (c) the functional dissociation constant (p*K*_B_/pA_2_) using either the dose ratio eq 1 or for **39** the Schild regression analysis showed excellent agreement, confirming that these 11 compounds are high-affinity hA_3_R-selective antagonist candidates, especially the lead compounds **39**.

**Table 5:** Binding Affinities of 11 Compounds Determined from Different Assays at hA_3_R Showed Good Agreement to Each Other

compound	p*K*_i_ (equilibrium)^a^	p*K*_d_ (kinetics)^b^	p*K*_B_ (cAMP)^c^
**37**	7.33 ± 0.06	7.30 ± 0.10	7.45 ± 0.19
**39**	7.92 ± 0.06	8.11 ± 0.04	#7.95 ± 0.15
**40**	6.79 ± 0.07	6.57 ± 0.09	7.05 ± 0.13
**47**	7.14 ± 0.04	7.43 ± 0.11	6.99 ± 0.18
**48**	7.01 ± 0.05	7.25 ± 0.08	7.09 ± 0.22
**59**	7.04 ± 0.08	7.11 ± 0.09	6.95 ± 0.13
**60**	7.23 ± 0.06	6.54 ± 0.09	6.89 ± 0.07
**74**	7.53 ± 0.06	7.62 ± 0.02	7.32 ± 0.05
**75**	7.56 ± 0.04	7.60 ± 0.12	7.39 ± 0.09
**76**	7.36 ± 0.07	7.34 ± 0.07	7.30 ± 0.14
**77**	6.90 ± 0.03	7.11 ± 0.10	6.55 ± 0.21

a Saturation binding affinity (p*K*_i_) determined through NanoBRET competition binding assay using the Cheng-Prusoff equation. Data taken from Tables 1 and 3.

b Kinetic dissociation constant (p*K*_d_) for each compound determined from *K*_off_/*K*_on_ in Tables 2 and 4.

c Dissociation constant (p*K*_B_) as determined through cAMP response using dose ratio equation for each compound or ^#^ Schild analysis for **39**.

### Binding Profile Investigation

#### MD Simulations

To investigate the binding profile of the best antagonists shown in Scheme 2 at hA_3_R, we performed MD simulations with representative compounds, i.e., **37**, **38**, **39**, and **56** in complex with hA_3_R embedded in POPC bilayers. We used a multistate AF2 method^41,42^ of hA_3_R generated with the GPCRdb^43^ web-tool. We optimized this model of hA_3_R in ref (49) to achieve best agreement with thermodynamic and kinetic data. In the starting docking pose of these compounds, the dichlorophenyl group was oriented toward TM5 and TM6 and is the same as we previously observed for **K18** and **K32** (compound **44** in this study).^15,24,25^

**Figure 4:**
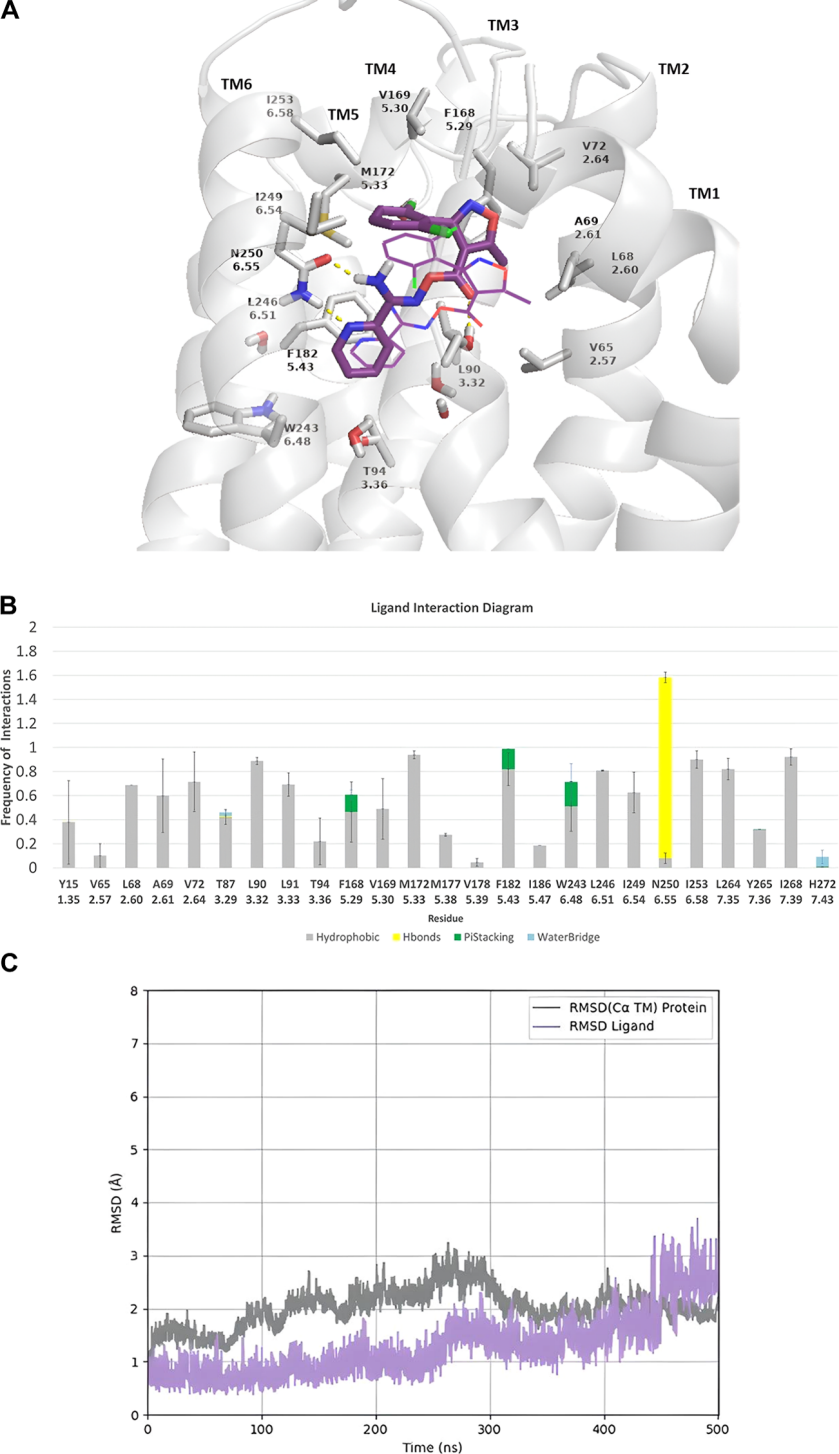

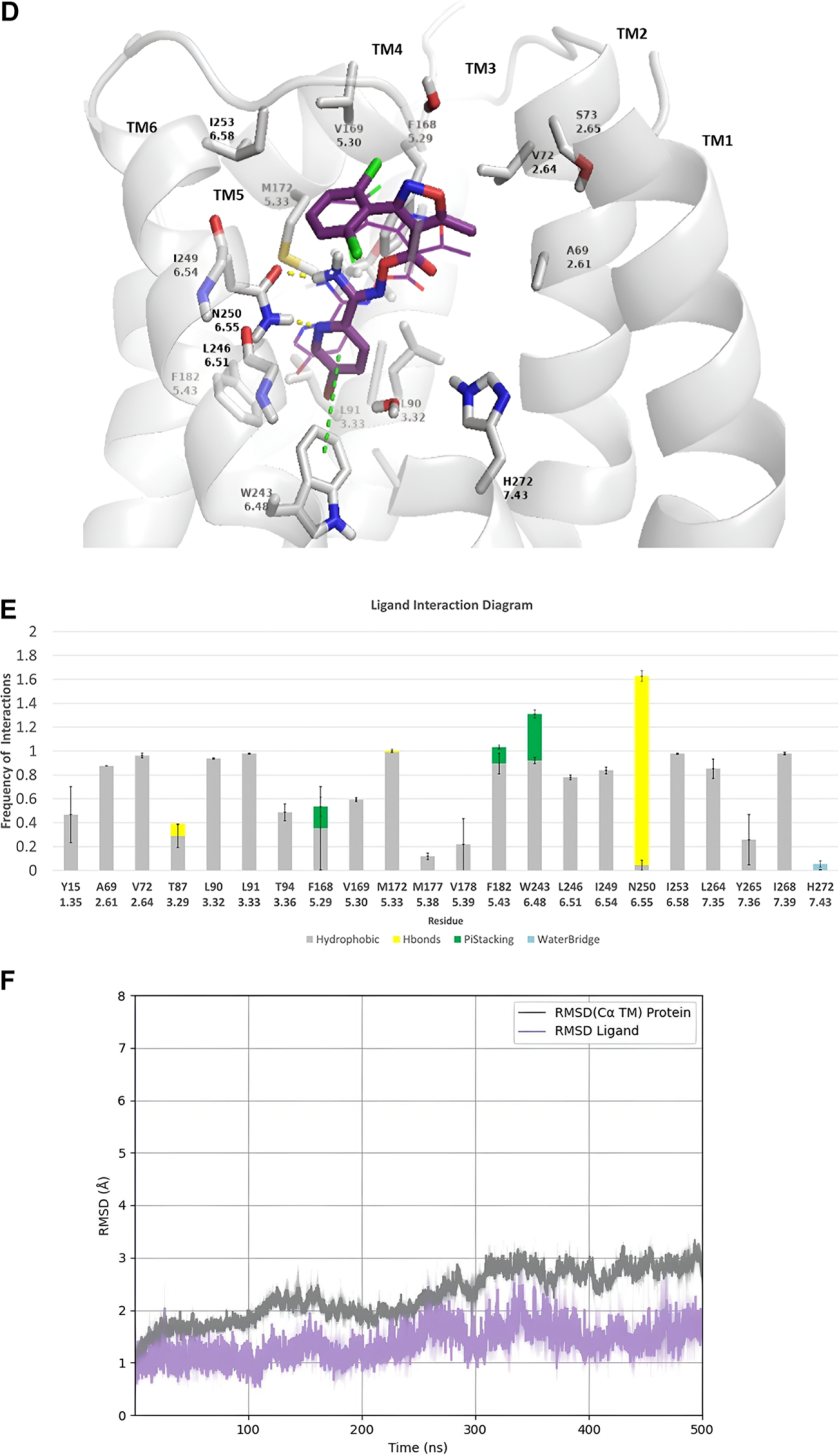
500 ns MD simulations for the complex of compounds of **37** and **39** with the WT hA_3_R using the amber ff19sb.^39^ (Α,D) Representative frame of the ligand inside the orthosteric binding area. (B,E) Receptor–ligand interaction frequency histograms; bars are plotted only for residues with interaction frequencies ≥0.2. Color figure in frames or bar plots: ligand is shown with pink sticks and ligand’s starting position with a pink wire, receptor is shown with a white cartoon and sticks, hydrogen bonding interactions are shown with yellow dashes or bars, π–π interactions are shown with green dashes or bars; hydrophobic interactions are shown with gray bars; and water bridges are shown with blue bars. (C,F) rmsd plots of Ca carbons of the protein (gray line) and of heavy atoms of the ligand (magenta line). For MD simulations, we used a revised model of the inactive form of hA_3_R we have recently published,^40^ generated using the multistate AF2 method^41,42^ of hA_3_R generated from GPCRdb web-tool;^43^ the complexes of the starting structure (docking pose) and final snapshot from the MD simulations are available as pdb files (see the Ancillary Information).

Using 500 ns MD simulations of the antagonists **37** and **39** in complex with hA_3_R, we were able to reveal the important residues in the orthosteric binding area essential for **37** and **39** binding (Figure 4). We observed that with compound **37**, its dichlorophenyl group orientates toward TM5 and TM6 to form dispersion interactions interacting with V169^5.30^, M172^5.33^, and I249^6.54^, F182^5.43^, and I253^6.58^. Moreover, the isoxazole forms aromatic π–π stacking interaction with the phenyl group of F168^5.29^ (Figure 4A,B). The amide side chain of N250^6.55^ is suggested to form a bidentate hydrogen bond with the carboximidamide amino group, while the 2-pyridinyl nitrogen can form water-bridged interactions with N250^6.55^. Nitrogen and oxygen atoms of isoxazole can form hydrogen bonds with the NH groups of F168^5.29^ or V169^5.30^, and the carboximidamide carbonyl group is suggested to form water-bridged interactions with H272^7.43^. There are also other water-bridged interactions between the isoxazol-4-carbonyloxycarboximidamide and example V72^2.64^, F168^5.29^, M172^5.33^, L246^6.51^, and I268^7.39^. The 2-pyridinyl group forms dispersion interactions with L90^3.32^, L91^3.33^, T94^3.36^, M177^5.38^, F182^5.43^, W243^6.48^, L246^6.51^, and I253^6.58^. The methyl group in the isoxazole ring can interact with V72^2.64^, Y15^1.35^, A69^2.61^, V72^2.64^, L264^7.35^, I268^7.39^, and H272^7.43^ through dispersion interactions. By modifying **37** into **39** through a 5-bromo substitution to the 2-pyridinyl group, we observed that **39** is tilted from TM7 and ΤΜ4 to TM5 showing additional dispersion interactions with M177^5.38^, V178^5.39^ and loosing interactions with F168^4.52^, L264,^7.35^ and H272^7.43^ (Figure 4D,E). Significantly, compared to **37**, **39** moves deeper into the bottom of the binding area forming hydrogen bonding interactions with T87^3.29^. In **38**, the 3,5-dichloro substitution in the 2-pyridinyl group diminishes affinity by ∼316-fold, and the 500 ns MD simulations suggested that this ligand escaped from the binding area (Figure S5A–C). We further expanded the MD simulation analysis in the oxadiazole series **56**–**61** by studying compounds **56**, **57**, and **60** (see Discussion in the Supporting Information and Figure S6).

#### TI/MD Calculations

**Figure 5:**
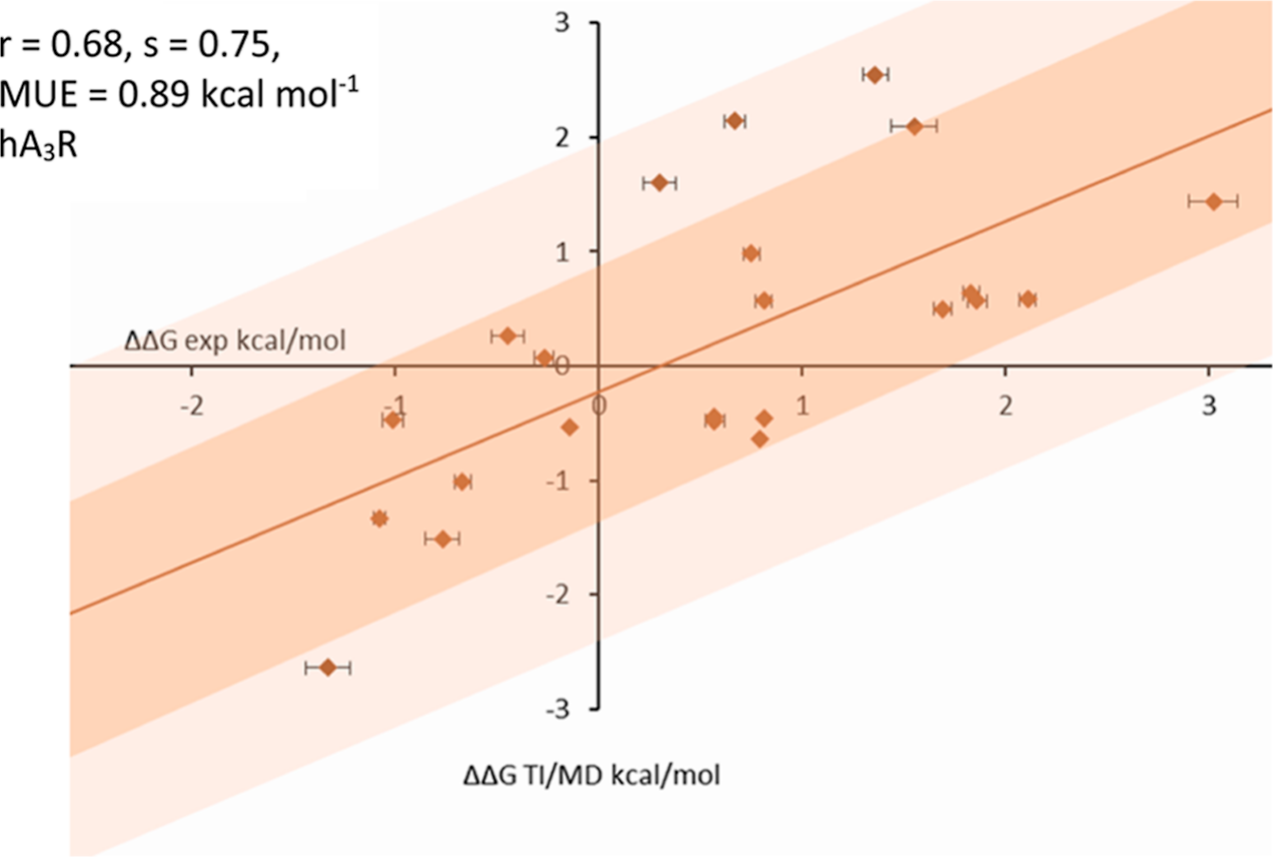
Computed ΔΔ*G*_b_,_TI/MD_ values plotted against ΔΔ_*G*b,exp_ values estimated by the experimental binding affinities p*K*i (Table S1) for hA3R using NanoBRET binding assay; *r*: correlation coefficient, *s*: slope. For TI/MD simulations, we used a revised model we recently published^40^ of the inactive form of hA_3_R generated based on the multistate AF2 method^41,42^ of hA_3_R.

MD simulations can describe qualitatively SARs based on the inspection of MD simulation trajectories and protein–ligand interaction frequency plots. While, the TI/MD simulations can accurately calculate the changes in binding affinity between different substituents. The set of the studied compounds **K18**, **37**–**42**, **44**–**46**, **48**, **52**, **54**, **55**, and **74**–**77** display 3 orders of magnitude differences between their affinity range. The results from the TI/MD alchemical calculations for 23 pairs of ligands in complex with hA_3_R using our optimized model of hA_3_R are shown in Table S1. As is shown in Figure 5, the correlation coefficient between the TI/MD calculated relative binding free energies and experimental values was *r* = 0.68 (*p* = 0.026) with mean unsigned error (MUE) = 0.89 kcal mol^–1^ (Table S1).

#### Mutagenesis Studies of Compound **39** at hA_3_R in Comparison with **37**

Based on the interaction frequency of the amino acid residues in the orthosteric binding area in contact with antagonist, (Figure 4B,D) predicted from the 500 ns MD simulations, we next employed mutagenesis (alanine substitution except where alanine was present, then glycine was used) with NanoBRET binding assay to experimentally investigate residues that were suggested to be important for the binding of **37** and **39**. First, since mutation of residues in GPCRs can have a detrimental effect on receptor trafficking, we initially determined the cell–surface expression of WT and mutant Nluc-hA_3_R using fluorescence-activated cell sorting (FACS) in flow cytometry^50,51^ and presented as % WT (Table S6). 4 out of 24 of the hA_3_R mutants showed significantly reduced cell surface expression (compared with the WT). Conversely, the hA_3_R mutants V72^2.64^A, F168^5.29^A, and F182^5.43^A displayed increased cell surface expression. Despite these changes in the cell surface expression levels, all the mutants expressed sufficiently to enable NanoBRET ligand binding experiments to be formed. The equilibrium dissociation constant (*K*_d_) of the fluorescent ligand **CA200645** was determined for each mutant and the WT hA_3_R—all expressed individually in HEK293T cells (Table S6). While the affinity of **CA200645** remained unchanged for most of the hA_3_R mutants, Y15^1.35^A, L91^3.33^A, M172^5.33^A, M177^5.38^A, L246^6.51^A, and I268^7.39^A did display significantly lower affinity for **CA200645** with the worst L246^6.51^A (*K*_i_ ∼ 258 nM) being about 10-fold lower when compared with the WT (∼24 nM). For hA_3_R, F168^5.29^A, N250^6.55^A, and H272^7.43^A, **CA200645** was unable to bind, so these three could not be further investigated in competition binding assays. Importantly, the reductions in the binding affinity of **CA200645** did not correlate with the changes in the cell surface expression, indicating the sufficient expression of all mutants.

**Figure 6:**
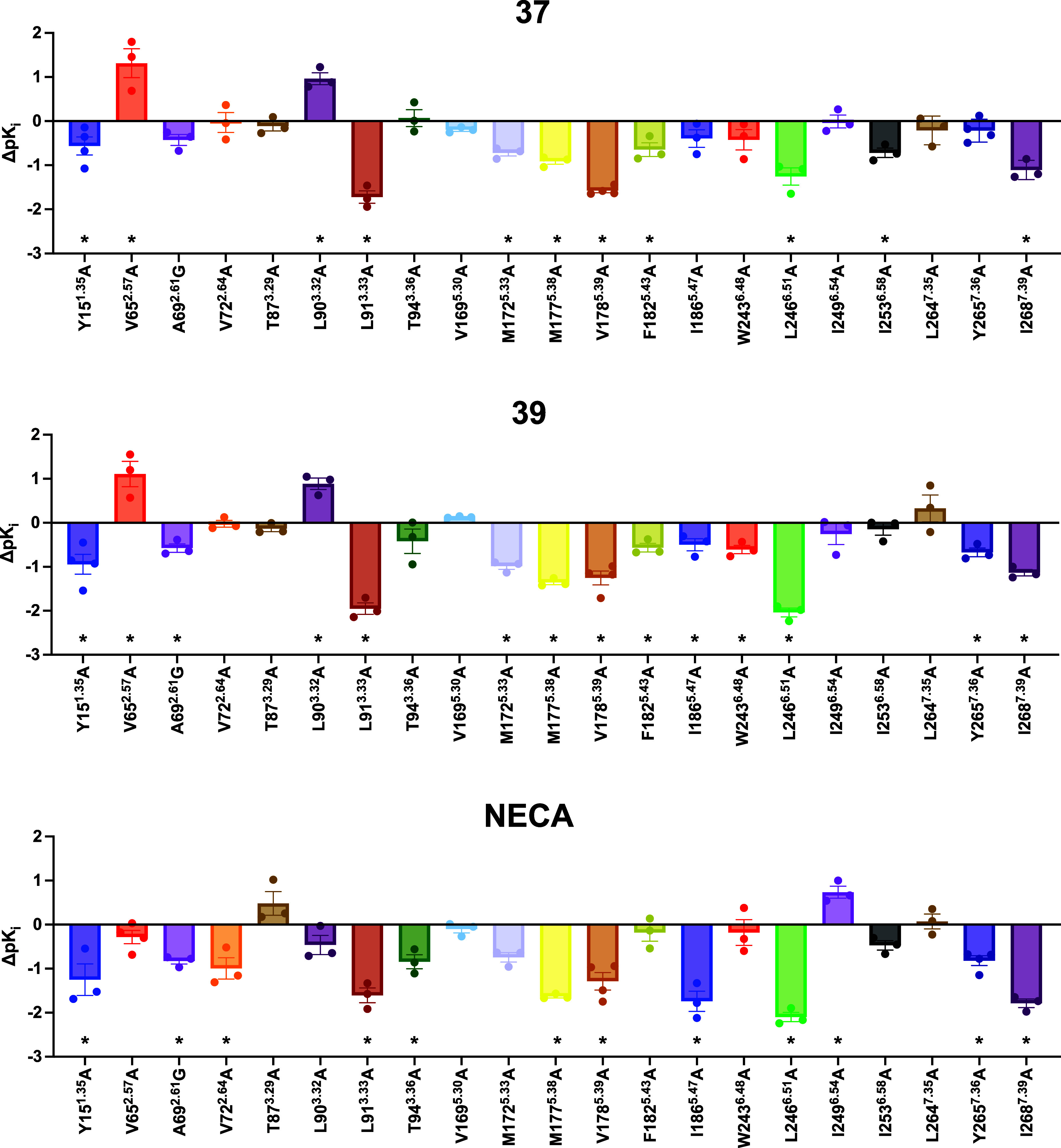
Changes in the binding affinities for compounds **37** and **39** and NECA measured using NanoBRET binding against WT and mutants hA_3_R. The binding affinity of **37** and **39** and NECA at hA_3_R WT and mutants were determined using NanoBRET binding assay performed in HEK293T transiently transfected with each construct. The change in affinity (Δp*K*_i_) is calculated as the difference of p*K*_i_ between the mutant and WT. Data is represented as mean ± SEM of *n* = 3 independent repeats conducted in duplicates. Statistical significance (*p < 0.05) compared with WT was determined using one-way ANOVA with Dunnett’s post-test.

In the NanoBRET competition binding assays, **37**, **39**, and agonist **NECA** were tested at WT and the 21 mutant hA_3_R, and the mutational effect was represented as the change in affinity compared with the WT (Δp*K*_i_) (Table S6 and Figure 6). There were 8 mutants (Y15^1.35^A, L91^3.33^A, M172^5.33^A, M177^5.38^A, V178^5.39^A, F182^5.43^A, L246^6.51^A, and I268^7.39^A) which showed significantly reduced affinity for both **37** and **39**, while V65^2.57^A and L90^3.32^A displayed increases in affinity for both antagonists. This indicated the importance of these residues in composing the orthosteric binding pocket of **37** and **39**. Noteworthy, while the effects of mutating the residues Y15^1.35^, L91^3.33^, M172^5.33^, M177^5.38^, L246^6.51^, and I268^7.39^ to alanine was the same between **37**, **39**, and **CA200645**, the residues V178^5.39^ and F182^5.43^ appear to be unique for **37** and **39** forming dispersion interactions with the 2-pyridinyl group of these antagonists.

Also, **37** and **39** displayed different extents of changes in the affinities at several mutants which might explain the increased affinity of **39** when compared to **37**. Compound **39** showed greater reductions in the affinity at L91^3.33^A and L246^6.51^A (∼97-fold and ∼115-fold, respectively) when compared with **37** (∼58-fold and ∼22-fold, respectively). This correlates well with the simulation results (the additional Br going deeper into the binding pocket composite of L91^3.33^A and L246^6.51^A and therefore increasing the affinity). Additionally, W243^6.48^A showed significant reduction (*p* = 0.0006) in the affinity of **39** but not of **37** (*p* = 0.2192) when compared with the WT, indicating that there is a stronger π–π interaction of **39** with the extra bromine. On the other hand, I253^6.58^A at the top of the binding pocket had significant reduced affinity for **37** (*p* = 0.0030) but not for **39** (*p* = 0.7289), suggesting that **37** interacts more with the top of the binding pocket than **39**.

For the completeness of the study, we have also assessed the binding affinity of agonist **NECA** at all the hA_3_R mutants and compared with WT. Among the 21 mutants tested, there were 12 mutants previously studied with **NECA** using cAMP accumulation assay.^52^ The mutational effects of these mutants in the potency of cAMP responses induced by **NECA** showed a good agreement with their effects at the change of binding affinity of **NECA** in this study. For example, T94^3.36^A, L246^6.51^A, I268^7.39^A, and M177^5.38^A showed significant reduction in the binding affinity of **NECA**, while M177^5.38^A also showed reduction in the cAMP response potency, and the other three mutants showed no cAMP response. Also, the agonist **NECA** and the antagonists **37** and **39** showed differences in the change of affinity at several mutants. **37** and **39** showed increased affinity at both V65^2.57^A and L90^3.32^A while **NECA** displayed no changes. Also, **NECA** had higher affinity at I249^6.54^A and lower affinity at V72^2.64^A and T94^3.36^A, while **37** and **39** had no change at these mutants. The agreement between two studies with different types of pharmacological assays and the ability to reveal the differences between the binding pockets of agonist and antagonisshowed the robustness of the mutagenesis study.

### Disease Model Validation and In Vitro Pharmacokinetic Profiling

#### Validation of Compound Selectivity at Endogenously Expressed Receptors in a Disease Model of Lung Cancer

**Figure 7:**
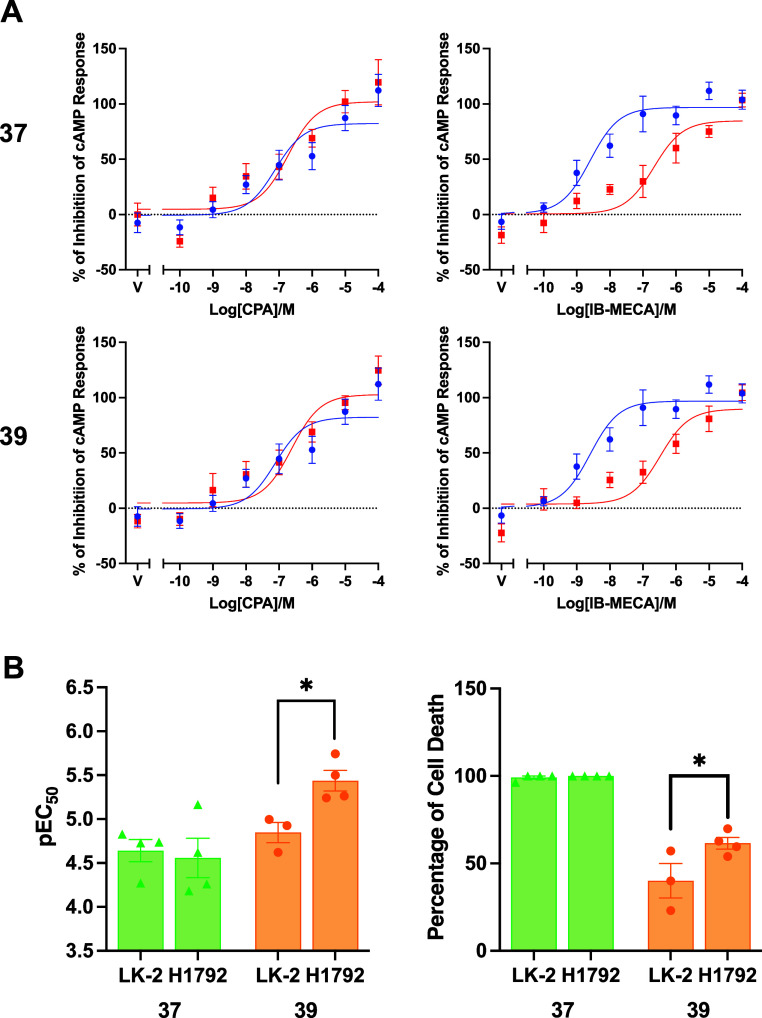
Selective inhibition of hA_3_R in nonsmall cell lung carcinoma cells inhibits proliferation. (A) Inhibition of forskolin-mediated cAMP accumulation in NCI-H1792 cells in response to **CPA** or **IB-MECA**, costimulated with **DMSO** (blue circles) or 10 μM **37** or **39** (red squares). (B) pEC_50_ and *E*_max_ values for the inhibition of LK-2 and NCI-H1792 cell proliferation for **37** and **39**. Statistical significance determined using an unpaired Student’s *t*-test.

We next sought to confirm that the lead compounds, **37** and **39**, were able to selectively antagonize hA_3_R when endogenously expressed. We utilized LK-2 and NCI-H1792 cells, which are both nonsmall cell lung carcinoma cells. LK-2 cells display low hA_1_R and hA_2B_R expression, whereas NCI-H1792 cells express all the 4 adenosine receptor subtypes.^35^ Neither *N*^6^-cyclopentyl-adenosine (**CPA**; A_1_R selective agonist) nor 1-deoxy-1-[6-[[(3-iodophenyl)methyl]amino]-9*H*-purin-9-yl]-*N*-methyl-β-D-ribofuranuronamide (**IB-MECA**, A_3_R selective agonist) was able to inhibit forskolin-mediated cAMP accumulation in LK-2 cells, consistent with their low adenosine receptor expression (Figure S7). **CPA** inhibited forskolin-mediated cAMP production in NCI-H1792 cells (pEC_50_ 6.51 ± 0.63), but neither **37** nor **39** was able to antagonize the response (pEC_50_ values of 6.83 ± 0.52 and 7.13 ± 0.60, respectively) (Figure 7 and Table S7). The response to **IB-MECA** was more potent (pEC_50_ of 8.50 ± 0.32), with both **37** and **39** significantly reducing the potency of the response (6.69 ± 0.57 and 6.24 ± 0.42, respectively). Calculated p*K*_B_ values were similar to those measured in the heterologous expression system (7.17 ± 0.13 and 7.46 ± 0.20). This confirmed the ability of both compounds to selectively antagonize hA_3_R when endogenously expressed alongside other adenosine receptors.

hA_3_R has often been referenced as a promising target for treating cancer.^34^ We therefore aimed to see the effect of selectivity antagonizing the receptor on cell proliferation. Compound **37** significantly impaired proliferation, displaying toxicity even in the absence of A_3_R (Figure S7 and Table S8). While **39** still reduced proliferation in LK-2 cells, showing some nonspecific toxicity, the effect was increased in NCI-H1792 cells, with an increase in potency and an increase in the percentage of cell death (Figure 7).

#### Pharmacokinetic Assessment of Lead Compound **39**

The highest affinity compound **39** was evaluated in the in vitro pharmacokinetic study including solubility, absorption, and metabolism (Table 6). For the solution properties, **39** showed low aqueous solubility with a mean solubility of 0.10 μM in phosphate buffer solution (PBS), 0.16 μM in simulated gastric fluid, and 43.13 μM in simulated intestinal fluid. It displayed a comparable partition coefficient (Log *D* = 2.82) with the reference compounds **haloperidol** (Log *D* = 2.49) and **phenytoin** (Log *D* = 2.28), showing adequate lipophilicity. Compound **39** also showed high protein binding in human plasma, with 99% of the proteins bound, which is similar to that of the reference compounds **sertraline** (99%) and **warfarin** (95%).

**Table 6:** *In Vitro* Pharmacokinetic Profile of Compound **39** and Reference Compounds

solution properties				
aqueous solubility^a^				
		**39**	**diethyl stilbestrol**	**disulfiram**
	In PBS, pH 7.4 (μM)	0.10	5.22	45.64
	in simulated gastric fluid (μM)	0.16	N.D.	N.D.
	in simulated intestinal fluid (μM)	43.13	N.D.	N.D.
partition coefficient^b^ (*n*-octanol/PBS, pH 7.4)				
		**39**	**haloperidol**	**phenytoin**
	Log *D*	2.82	2.49	2.28
protein binding^c^				
		**39**	**sertraline**	**warfarin**
	% protein bound	99	99	95
	% recovery	105	94	112
In Vitro **Absorption**				
permeability in Caco-2 cell^d^				
		**39**	**propranolol**	**labetalol**
	*P*_app_A-B (10^–6^ cm/s)	7.2	32.2	8.3
	*P*_app_B-A (10^–6^ cm/s)	0.6	32.6	38.2
	% recovery (A-B)	26	74	99
	% recovery (B-A)	19	96	103
	uptake ratio	12	0.99	0.22
In Vitro **Metabolism**				
intrinsic clearance (human liver microsomes)^e^				
		**39**	**terfenadine**	**verapamil**
	*t*_1/2_ (min)	9.5	16.9	26.6
	CL_int_ (μL/min/mg of microsomes)	729	410.1	261.1

a Aqueous solubility (μM) in PBS at pH 7.4/simulated gastric fluid/simulated intestinal fluid determined with high-performance liquid chromatography–ultraviolet spectroscopy.

b The partition efficient of the compound between *n*-octanol and PBS at pH7.4, measuring the lipophilicity of the tested compound. Log *D* was calculated as Log10(the amount of compound in *n*-octanol/the amount of compound in PBS).

c Measure of percentage of protein binding and percentage of compound recovery during the assay determined in equilibrium dialysis using human plasma.

d The permeability of compounds assessed in bidirectional Caco-2 cell permeability assay with pH = 6.5 for donor chamber and pH = 7.4 for receiver chamber. The extent of permeability is measured as apparent permeability coefficient (*P*_app_) from apical (A) to basolateral (B) or in reverse direction. The percentage recovery of the compound is calculated as the total amount of compound in the donor and the receiver at the end of experiment/the amount of initial compound present. The uptake ratio of the compound is calculated as *P*_app_A-B/*P*_app_B-A.

e The metabolic stability of compounds was determined in 0.1 mg/mL human liver microsomes, measured as the half-life (*t*_1/2_) and apparent intrinsic clearance (CL_int_).

The in vitro absorption of **39** was assessed using the bidirectional permeability assay (*P*_app_) in Caco-2 cells. Compound **39** had a *P*_app_ of 7.2 × 10^–6^ cm/s from the apical side (A) to the basolateral side (B) and a *P*_app_ of 0.6 × 10^–6^ cm/s from B to A, resulted in an uptake ratio (*P*_app_A-B/*P*_app_B-A) of 12, suggesting no drug efflux across the membrane occurred. However, the percent recovery was lower (26% A to B and 19% B to A) compared to the reference compound in both directions (74% A-B and 96% B-A for **propranolol**), indicating potential problems like poor solubility or nonspecific binding during the assay.

The in vitro metabolism of **39** was assessed as its intrinsic clearance in human liver microsomes. Compound **39** had a shorter half-life (9.5 min) than the precursor compound **K18** (24 min^25^) as well as the reference compounds (**terfenadine**, 16.9 min and **verapamil** 26.6 min). The resulted intrinsic clearance (CL_int_) was 729 μL/min/mg of microsome, which was higher than **K18** (287.2 μL/min/mg of microsome^25^) **terfenadine** (410.1 μL/min/mg of microsome) and **verapamil** (261.1 μL/min/mg of microsome). These indicated that **39** has a relatively rapid metabolism in hepatic microsomes and therefore will cause less accumulation and potential toxicity.

## Discussion

Here, we have reported a hit-to-lead study by computational design, synthesis, and screening using NanoBRET binding assay of 25 novel derivatives targeting selectively hA_3_R, as analogues or derivatives of the selective A_3_R carbonyloxycarboximidamide heterocyclic hits **K17**, **K18**,^15^ or **K32**(24,25) from a previous structure-based VS.^15,24,25^ In these previously reported hits, the carbonyloxycarboximidamide moiety was connected with a 3-(2-chlorophenyl)-isoxazolyl, 3-(2,6-dichlorophenyl)-isoxazolyl or 3-(2-chlorophenyl)-isoxazolyl group, respectively, and the carboximidamide carbon was connected with the 1,3-thiazolyl or 2-pyridinyl group.^15,24,25^ Their NanoBRET-based determined dissociation constants for **K17**, **K32**, and **K18** were *K*_i_ = 600, 250, and 120 nM, respectively, showing the importance of attaching two ortho-chlorine substituents in the phenyl group attached to isoxazole ring and that as regards affinity and antagonistic potency 2-pyridinyl (**K32**) > 4-(1,3-thiazolyl) (**K17**) > 3-pyridinyl (**K10**) > 4-pyridinyl (**K11**).^24,25^ However, the residence time of these hits was low and unmeasurable (<1 min).^25^

We used, for the SBDD of the novel carbonyloxycarboximidamide derivatives, the same binding pose of **K18** (or **K32**) in the orthosteric binding area of hA_3_R. This binding pose was previously suggested^16^ after refinement of the docking pose of **K18** from VS^15^ using 100 ns MD simulations with the OPLS2005^37^ force field. The binding pose in **K18**(14) was further confirmed with a combination of 500 ns MD simulations using the ff14sb and alanine mutagenesis supported by affinity experiments.^24,25^ In **K18** (or **K32**), the phenyl group that is attached at the 3-position of the isoxazole ring is oriented to the upper region of the receptor, while the 1,3-thiazolyl or 2-pyridinyl group was oriented deep in the receptor. There is an increasing propensity of the phenyl group to turn toward TM5 and TM6 by increasing the chlorine substituents attached to the available ortho positions of the phenyl group junction. Based on these observations, we used alchemical binding free energy calculations applied with the TI/MD and ff19sb simulations in complexes of ligands with a revised model for hA_3_R we recently published^40^ generated from the multi-AF2-based model for inactive hA_3_R (Table S1) to design and synthesize more potent compounds than **K18** (or **K32**). At the time we submitted this paper, the structure of the active state of hA_3_R in complex with agonists and Gi heterotrimer was reported.^7^ Interestingly, in the experimental structure, the orientation of R173^5.34^ matches the conformation we used in our revised multi-AF2-based model for the inactive hA_3_R that we use for all the MD simulations performed here.^40^ The purchase of compounds like **K18** (or **K32**) for testing^15,24,25^ from companies was costly, and the synthesis of these series with sufficient diversity in structure for exploring SARs was accomplished simply and in good yields using commercially available carbonitriles (Schemes 3 and 4). The heterocyclic carbonyloxycarboximidamides **37**–**55** and **74**–**77**, and their structurally related 1,2,4-oxadiazole derivatives **56**–**61**, provide novel and highly selective hA_3_R antagonists, which are synthetically feasible and easily accessed drug molecules amenable for further development.

Among the 29 synthesized compounds (belonging in groups A–F; Tables 1, 3, and S3), there are three groups of compounds (A–C; Tables 1 and S3) which only differ by the number of chloro-substituents in the phenyl ring of 3-phenyl-isoxazole, (a) **37**, **42**, and **44**; (b) **40**, **41**, and **43**; and (c) **49**, **51**, and **50**. By comparing their binding affinities at hA_3_R, we showed that the affinities of antagonists increased as the number of chloro-substituents increased, which is in line with our findings.^15,24,25^ In agreement with our previous studies,^25^ the addition of chlorine atoms at the ortho positions of the phenyl group in the 3-phenyl-isoxazole moiety enhanced affinity since the dichlorophenyl group enhances the hydrophobic interactions toward TM5 and TM6 with residues V169^5.30^ and I249^6.54^. This was shown in the relative binding free energy values which are ΔΔ*G*_b,exp_ = 1.83 ± 0.06 kcal mol^–1^ and ΔΔ*G*_b,TI/MD_ = 0.64 ± 0.04 kcal mol^–1^ with a deviation of 1.19 kcal mol^–1^ for **37** → **44**, ΔΔ*G*_b,exp_ = 0.75 ± 0.05 kcal mol^–1^ and ΔΔ*G*_b,TI/MD_ = 0.98 ± 0.04 kcal mol^–1^ with a deviation of 0.23 kcal mol^–1^ for **42** → **44**, ΔΔ*G*_b,exp_ = −1.08 ± 0.04 kcal mol^–1^ and ΔΔ*G*_b,TI/MD_ = −1.33 ± 0.03 kcal mol^–1^ with a deviation of 0.25 kcal mol^–1^ for **42** → **37**, and ΔΔ*G*_b,exp_ = −1.01 ± 0.06 kcal mol^–1^ and ΔΔ*G*_b,TI/MD_ = −0.47 ± 0.05 kcal mol^–1^ with a deviation of 0.54 kcal mol^–1^ for **41** → **40**.

Replacement of 2,6-dichlorophenyl group attached to the isoxazole ring with 2,6-dibromophenyl or 2,6-dimethylphenyl in **37** or **39**, respectively, resulted in compounds **74, 76** or **75, 77**, respectively (groups E and F; Table 3). Compared to **37**, the binding affinity of **74** was improved by 1.7-fold; conversely, it was reduced by 2.7-fold for **76**. Interestingly, the modifications on both **75** and **77** resulted in reduced affinities at hA_3_R compared to **39**. This was shown in the relative binding free energy values which are for **37** → **74** ΔΔ*G*_b,exp_ = −0.14 ± 0.05 kcal mol^–1^ and ΔΔ*G*_b,TI/MD_ = _–_ 0.53 ± 0.04 kcal mol^–1^ with a deviation of 0.39 kcal mol^–1^, for **37** → **76** ΔΔ*G*_b,exp_ = 0.79 ± 0.05 kcal mol^–1^ and ΔΔ*G*_b,TI/MD_ = _–_ 0.64 ± 0.05 kcal mol^–1^ with a deviation of 1.43 kcal mol^–1^; for **39** → **75**, we calculated ΔΔ*G*_b,exp_ = 0.57 ± 0.07 kcal mol^–1^ and ΔΔ*G*_b,TI/MD_ = −0.45 ± 0.04 kcal mol^–1^ with a deviation of 1.02 kcal mol^–1^ and for **39** → **77** and ΔΔ*G*_b,exp_ = 0.81 ± 0.08 kcal mol^–1^ and ΔΔ*G*_b,TI/MD_ = −0.46 ± 0.05 with a deviation of 1.27 kcal mol^–1^.

Within group A, all compounds having a dichlorophenyl group attached at the 3-isoxazole position only differ by the aryl group attached to the carbonyloxycarboximidamide moiety, when compared to **K18**. This aryl group showed crucial effects on the binding affinity. Replacement of 2-pyridinyl group in **37** (*K*i = 34.7 nM) with the less basic by ∼10^3^-fold 1,3-thiazolyl group in **K18** (*K*i ∼ 120 nM) or the less basic by ∼10^4^-fold 2-pyrimidinyl group in **45** (*K*_i_ ∼ 537 nM) led to a reduced affinity by 3.5-fold or ∼15.5-fold due to the stronger hydrogen bond that can be formed between the N250^6.55^ amido side chain and 2-pyridinyl group.^44^ The corresponding relative binding free energy values for **K18** → **37** are ΔΔ*G*_b,exp_ = −0.77 ± 0.08 kcal mol^–1^ and ΔΔ*G*_b,TI/MD_ = −1.51 ± 0.08 kcal mol^–1^ with deviation |ΔΔ*G*_b,TI/MD_ - ΔΔ*G*_b,exp_| = 0.74 kcal mol^–1^ and for **37** → **45** are ΔΔ*G*_b,exp_ = 1.69 ± 0.05 kcal mol^–1^ and ΔΔ*G*_b,TI/MD_ = 0.50 ± 0.04 kcal mol^–1^ with a deviation of 1.19 kcal mol^–1^.

It is worth noting that the five compounds with high affinities in this group all have a nitrogen atom at a similar position as the nitrogen in the thiazole ring of **K18**, see compound **37**, which is in line with our findings.^15,24,25^ Changes in this nitrogen position reduce the ability of aryl nitrogen to form a hydrogen bond with N250^6.55^ amido side chain. Thus, compared to the 2-pyridinyl analogue **37** (*K*i = 34.7 nM), the 3-pyridinyl analogue **48** (*K*_i_ ∼ 100 nM) had ∼3-fold lower affinity similar to previous observations for the monochloro derivatives **K32** and **K10** (Scheme 1). The corresponding relative binding free energy values for **37** → **48** were ΔΔ*G*_b,exp_ = 0.67 ± 0.05 kcal mol^–1^ and ΔΔ*G*_b,TI/MD_ = 2.14 ± 0.08 kcal mol^–1^ with a deviation 1.47 kcal mol^–1^. Other changes that reduce the ability of pyridinyl nitrogen to form a hydrogen bond with N250^6.55^ amido side chain are discussed below. Replacement of 2-pyridinyl in **37** with 2-pyridinylmethylene in **54** (*K*_i_ ∼ 316 nM) drives nitrogen away from N250^6.55^ and causes reduction in binding affinity, correspondingly, by ∼9-fold. The corresponding relative binding free energy values for **37** → **54** were ΔΔ*G*_b,exp_ = 1.36 ± 0.04 kcal mol^–1^ and ΔΔ*G*_b,TI/MD_ = 2.55 ± 0.06 kcal mol^–1^ with a deviation of 1.19 kcal mol^–1^. For the same reason is observed reduction in binding affinity by ∼8.7-fold when the 3-pyridinyl group in **48** (*K*_i_ ∼ 102 nM) is changed to the 3-pyridinyl methylene group in **55** (*K*_i_∼ 891 nM). Compared to **48** (*K*_i_ ∼ 102 nM) bearing the 3-pyridinyl group, compound **46** (*K*_i_ ∼ 2089 nM) with the 2-chloro-3-pyridinyl group has 20.5-fold lower binding affinity since the 2-chloro-substituent hampers the hydrogen bonding interaction of 3-pyridinyl nitrogen. This was shown in alchemical perturbation **48** → **46** ΔΔ*G*_b,exp_ = −1.33 ± 0.05 kcal mol^–1^ and ΔΔ*G*_b,TI/MD_ = −2.64 ± 0.11 kcal mol^–1^. The 3-tolyl group in **48** (*K*i ∼ 209 nM) does not form a hydrogen bond, and compared to 3-methyl-2-pyridinyl in **40** (*K*i ∼ 100 nM), its binding affinity was reduced by 2.1-fold; the corresponding alchemical transformations **55** → **48** showed ΔΔ*G*_b,exp_ = −1.33 ± 0.05 kcal mol^–1^ and ΔΔ*G*_b,TI/MD_ = −2.64 ± 0.11 kcal mol^–1^ with a deviation of 1.31 kcal mol^–1^ and **40** → **49** showed ΔΔ*G*_b,exp_ = 0.30 ± 0.08 kcal mol^–1^ and ΔΔ*G*_b,TI/MD_ = 1.60 ± 0.09 kcal mol^–1^ with a deviation of 1.30 kcal mol^–1^. Interestingly, while the addition of methyl group at α-position to nitrogen increases pyridine basicity by 5.4-fold,^53^ the 6-methyl-2-pyridinyl derivative **40** (*K*_i_∼ 128 nM) has a 3.7-fold lower affinity compared to **37**. Therefore, we assumed that the methyl group points toward an area of hA_3_R where the water density is increased. Previously, we suggested^60^ that such an area may reside between a polar substituent of the ligand and TM2 and TM3. The corresponding relative binding free energy values for **37** → **40** were ΔΔ*G*_b,exp_ = 0.81 ± 0.06 kcal mol^–1^ and ΔΔ*G*_b,TI/MD_ = 0.57 ± 0.04 kcal mol^–1^, with a deviation of 0.24 kcal mol^–1^.

Compound **39** (*K*_i_ ∼ 11.7 nM) displayed about a 10- or 3-fold increase in affinity compared to **K18** (*K*_i_ ∼ 120 nM) or **37** (*K*_i_ = 34.7 nM), respectively, whereas **38** (*K*_i_ ∼ 1072 nM) was significantly (*p* < 0.0001) lower in affinity. Among these high-affinity candidates, **K18**, **K38**, and **K39**, compound **39** (the most potent) uniquely has a bromine group in the 2-pyridinyl group at position 5. This brings a 3.9-fold increase in affinity when compared with **37** which only has the pyridinyl group instead since the bromine group at 5-position of the 2-pyridinyl group forms dispersion interactions with W243^6.48^ and F182^5.43^. For the alchemical transformation **37** → **39** in which a bromine group is added at 5-position of the 2-pyridinyl group in **37**, ΔΔ*G*_b,exp_ = −1.33 ± 0.05 kcal mol^–1^ and ΔΔ*G*_b,TI/MD_ = −0.67 ± 0.04 kcal mol^–1^ with a deviation of 0.34 kcal mol^–1^. Compared to **37**, the 3,5-dichloro-2-pyridinyl group in **38** (*K*_i_ ∼ 1072 nM) reduces the affinity by ∼31-fold since the 3-chlorine atom is not favored lying in an area where the water density is increased. Indeed, for alchemical perturbations, **37** → **39** ΔΔ*G*_b,exp_ = −0.67 ± 0.07 kcal mol^–1^, ΔΔ*G*_b,TI/MD_ = −1.01 ± 0.04 kcal mol^–1^, and **37** → **38** ΔΔ*G*_b,exp_ = 2.11 ± 0.04 kcal mol^–1^, ΔΔ*G*_b,TI/MD_ = 0.58 ± 0.04 kcal mol^–1^. Additionally, both **37** and **39** had increased RT (14 and 22 min, respectively) inside the orthosteric binding pocket when compared to **K18** or **K32** (<1 min).

Moreover, a rigidification of the carbonyloxycarboximidamide moiety led to the oxadiazole derivatives with **59** and **60** having slightly higher affinities compared to their precursors **48** and **47**. The 500 ns MD simulations showed that rigidification of the carbonyloxycarboximidamide moiety to oxadiazole derivatives **56**–**61** (class D, Tables 1 and S3) causes reorientation of the dichlorophenyl group through rotation around the oxazole-oxadiazole C–C bond as we showed for compounds **56**, **57**, and **60** (see Figure S6). Thus, the dichlorophenyl group faces TM2 in compounds **56** and **57** and TM7 in compound **60**. Between compounds **56** and **61** (class D, Tables 1 and S3), the 3-pyridinyl derivative **59** is a stronger binder by 2-fold compared to 2-pyridinyl derivative **56**, while for the acyloxyimidamide 2-pyridinyl derivative, **37** has 2-fold higher affinity compared to **48**. Compared to **59** (*K*_i_ = 91.2 nM and RT = 20.5 min), the methyl substituent at 4-position of the 3-pyridinyl group increased the affinity in **60** (*K*_i_ = 58.9 nM and RT = 10.7 min) since is oriented toward M243^6.48^ favoring hydrophobic interactions (see Figure S6,G–I), providing an additional lead for further improvement; the TI/MD results for this series are discussed in the Supporting Information. However, in functional cAMP experiments, we observed that they lost their hAR subtype selectivity. This contrasted with compounds **37**, **39, 40**, **47**, **48**, **and 74–77** which were shown to display high hA_3_R selectivity. Moreover, none of the compounds tested showed high (*K*_i_ > 1 μM) affinity toward rA_3_R.

We previously determined the effects of receptor mutation on antagonist potency (pA_2_) for A_3_R L90A^3.32^, V169^5.30^A/E, M177^5.40^ A, I249^6.54^A, and L264^7.34^A by functional assays.^25^ We reported^24,25^ that M177^5.38^A caused the most significant reduction on the **K18** antagonist effect. Interestingly, we found that L90A^3.32^ in the low region and L264A^7.34^ in the middle/upper region increased **K18** potency, while I249^6.54^A had little effect (compared to WT hA_3_R).^25^ We suggested that V169^5.30^ was not a selectivity filter for hA_3_R agonists or antagonists. Here, we also showed that^32,44,45^ V169^5.30^A did not cause a significant change in affinity for both **37** and **39** (Table S6). Moreover, we did observe significant reductions in affinity for both **37** and **39** with 8 of our mutants hA_3_R (Y15^1.35^A, L91^3.33^A, M172^5.33^A, M177^5.38^A, V178^5.39^A, F182^5.43^A, L246^6.51^A, and I268^7.39^A), while 2 other mutations (V65^2.57^A and L90^3.32^A) produced an increase in affinity for both antagonists **37** and **39**, suggesting that these 10 residues comprise the orthosteric binding pocket (Table S6).

However, there are also differences in binding profiles between compounds **37** and **39.** Indeed, **39** showed a greater reduction in the affinity at L91^3.33^A (∼97-fold) and L246^6.51^A (∼115-fold) when compared with **37** (∼58-fold and ∼22-fold, respectively). The effects of the Y15^1.35^A, Y265^7.36^, or W243^6.48^A mutations were also more significant for **39** than for **37**, which showed excellent agreement with our simulation results. It appears that the sizable hydrophobic bromine can orientate in the orthosteric binding area through the increase in hydrophobic interaction with W243^6.48^ and L91^3.33^. This was also validated through analysis of I253^6.58^A which is located on top of the binding pocket. The binding affinity of **37** showed a greater reduction in affinity compared to **39**, suggesting that **37** occupies a position near the top of the orthosteric pocket.

As is shown in Figure 5 using an AF2-generated model that we recently published,^40^ the correlation between the 23 TI/MD calculated relative binding free energies and experimental values measured with the NanoBRET binding assay was very good with r = 0.68 (*p* = 0.026) and MUE = 0.89 kcal mol^–1^ (Table S1). This shows that we can use this procedure for further optimization of this series of compounds which is ongoing research.

Finally, for future optimization and in vivo studies, lead compound **39** was assessed for its affinity for endogenously expressed receptors and in vitro pharmacokinetic properties. Compound **39** selectively antagonized **IB-MECA**, but not **CPA**, when looking at the inhibition of cAMP production, demonstrating its hA_3_R selectivity in cells expressing both inhibitory hARs. Furthermore, despite cell toxicity in both cell lines, **39** exerted a greater potency and efficacy for the inhibition of cellular proliferation in lung cancer cells expressing the A_3_R, reinforcing the receptor as a potential target for the treatment of cancer. When looking at the pharmacokinetic suitability of **39**, it showed 400-fold higher solubility in simulated intestinal fluid than in PBS or simulated gastric fluid. The partition coefficient and percentage of protein binding and permeability in Caco-2 cells were all comparable to reference compounds **haloperidol**, **sertraline**, or **labetalol**. In terms of in vitro metabolism, compound **39** showed a much larger intrinsic clearance in liver microsomes which resulted in a short half-life (∼9.5 min). Future optimization based on this compound should focus on improving its aqueous solubility and metabolic stability to increase the in vivo efficacy.

## Experimental Section

### Biological Methods

#### Compounds

**NECA, CPA, IB-MECA**, and **MRS1220** were purchased from Tocris Bioscience (Wiltshire, UK). Rolipram was obtained from Cayman Chemicals (Michigan, USA). All the ligands above-mentioned were dissolved in DMSO as 10 mM stock and stored at −20 °C until use. **Forskolin** was purchased from Tocris Bioscience (Wiltshire, UK), made up as 10 mM stock in DMSO, and stored at room temperature. **CA200645** and **CA200623** were purchased from HelloBio (Bristol, UK), dissolved in DMSO as 100 μM stock, and stored at −20 °C. The purity of the tested compounds was >95% (see Chemistry methods, General Information).

#### Constructs

The FLAG tag (DYKDDDDK) and Nluc-hA_3_R (gifted by Stephen Briddon) were cloned into vector pcDNA3.1(−). Site-directed mutagenesis was performed to make A_3_R mutants using the QuikChange Lightning kit (Agilent Technologies, US) according to the manufacturer’s protocol. All the construct sequences were confirmed by the DNA sequencing performed by the DNA sequencing facility at the Department of Biochemistry, University of Cambridge (Cambridge, UK).

#### Cell Culture and Transfection

The source of cells was as described previously.^25,35^ These cells were maintained in Dulbecco’s modified Eagle’s medium (DMEM)/Hams F-12 nutrient mix (F12) GlutaMAX media (ThermoFisher, UK), supplemented with 10% heat-inactivated fetal bovine serum (FBS) (Sigma-Aldrich, Poole, Dorset, UK) and 1% antibiotic-antimycotic (AA) (Sigma, UK). In the cAMP accumulation assay, the Chinese hamster ovary (CHO) K1 cell line was used because of its lack of endogenous ARs expression.^61^ Stable cell lines of CHO-K1-A_1_R, CHO-K1-A_2A_R, CHO-K1-A_2B_R, and CHO-K1-A_3_R were cultured with the Hams F-12 nutrient mix (ThermoFisher, UK) supplemented with 10% FBS and 1% AA. 600 μg/mL Geneticin (ThermoFisher, UK) was added to the stable HEK293T and CHO-K1 cell lines for selection, and the medium was changed every 2 days. In the mutagenesis study, HEK293T cells were plated to a 24-well plate and grown overnight. The seeded cells were then transfected with 250 ng of FLAG-Nluc-A_3_R WT or mutant receptor using polyethylenimine 25 kDa (PEI, Polysciences Inc., Germany) in 6:1 ratio, diluted in 150 mM NaCl. LK-2 and NCI-H1792 cells were cultured in RPMI 1640 medium supplemented with 10% FBS and 1% AA. All cells were maintained at 37 °C with 5% CO_2_ in a humidified atmosphere.

#### NanoBRET Binding Assay

NanoBRET ligand competition binding assays were performed to identify the saturation binding affinity (p*K*_i_) and the binding kinetic parameters of the potential antagonists. HEK293 cells stably expressing Nluc-hA_3_R/rA_3_R or HEK293T cells transfected with FLAG-Nluc-A_3_R WT or mutants for 24 h were seeded onto white 96-well plates (Greiner, UK) at a density of 10,000 cells/well and grown overnight. On the assay day, the medium was discarded and replaced with 100 μL of phosphate-buffered saline (PBS) buffer (ThermoFisher, UK) containing 0.1% bovine serum albumin (BSA, Sigma-Aldrich, Poole, Dorset, UK) and 0.1 μM Nano-Glo Luciferase substrate (Promega, UK). After 5 min of incubation in the dark, tested compounds in different concentrations together with 5 nM **CA200645** for Nluc-hA_3_R or 100 nM **CA200623** for Nluc-rA_3_R or **CA200645** at its *K*i concentration for individual FLAG-Nluc-hA_3_R mutants were added. The BRET signal was measured with Mithras LB940, recording the light emission at 460 nM (Nluc) and 610 nM (fluorescent ligands) for 30 min. The raw BRET ratio was calculated by dividing the emission at 610 nM with the 450 nM emission. Nonspecific binding was determined with the addition of high concentration of unlabeled antagonist, 10 nM **MRS1220**, and the corresponding BRET ratio was used for baseline correction. The baseline-corrected BRET ratio at 10 min poststimulation was used for calculating the affinity constant.

#### cAMP Competition Assay

In the cAMP inhibition (A_1_R or A_3_R) or accumulation (A_2A_R and A_2B_R) experiments, CHO-K1 cells stably expressing the corresponding receptor were performed as described previously.^25^ Briefly, cells expressing the receptor of choice were harvested and resuspended in stimulation buffer (PBS containing 0.1% BSA and 25 μM rolipram) and seeded at a density of 2000 cells/well in the white 384-well Optiplate. Potential antagonists or DMSO control were added with different concentrations of AR agonist **NECA**. In the experiment with A_1_R/A_3_R, cells were costimulated with 1 μM **forskolin** (an adenylyl cyclase activator) to detect the inhibition of cAMP response. LK-2 and NCI-H1792 cells were stimulated as above, but using 1000 cells/well, and 0.1 μΜ forskolin, with different concentrations of **CPA** or **IB-MECA**. After 30 min of stimulation, the cAMP levels were determined with a LANCE cAMP kit (PerkinElmers, MA, US).

#### Flow Cytometry

To assess the cell surface expression level of FLAG-Nluc-A_3_R WT and mutants, HEK293T cells transiently transfected with FLAG-Nluc-A_3_R WT or mutants were analyzed with FACS in flow cytometry. After 48 h of transfection, 300,000 cells were harvested from each sample and washed twice with FACS buffer (PBS containing 1% BSA and 0.03% sodium azide). The cells were then incubated with 50 μL of FACS buffer containing 1:120 phycoerythrin (PE) anti-DYKDDDDK(FLAG) tag antibody (BioLegend, San Diego, US) in the dark for 1 h. After incubation, the cells were washed twice again and resuspended in 50 μL of FACS buffer. Analysis was performed using a BD AccuriTM C6 flow cytometer with an excitation at 488 nm and an emission wavelength at 585 nm. The resulting median intensity of cells was normalized against cell transfection with pcDNA3.1(−) as 0% and FLAG-Nluc-hA_3_R WT as 100%.

#### Proliferation Assay

Proliferation assays were performed using the cell counting kit-8 (CCK-8) as described previously.^35^ Briefly, LK-2 or NCI-H1792 cells were plated at 2500 cells/well of a clear 96-well plates in complete RPMI media and cultured for 24 h. The cells were then treated with compounds over a concentration range for 72 h. CCK-8 reagent was added, and after 3 h, plates were read using a Mithras LB 940 multimode microplate reader.

#### In Vitro Pharmacokinetic Assessment

The pharmacokinetic assessment of **39** was outsourced and performed by Eurofins Panlabs (MO, USA). The assessments included the determination of aqueous solubility, protein binding ability, partition coefficient, permeability across Caco-2 cells, and the metabolic stability in human liver microsomes. The aqueous solubility was determined by comparing the peak area of standard (200 μM calibration standard dissolved in a solvent made up of 60% methanol and 40% water) with the peak area of the corresponding peak in an individual buffer (PBS at pH = 7.4, simulated gastric buffer, and simulated intestinal fluid) as the method shown in ref (54). A chromatogram of 200 μM test compound along with a UV/vis spectrum with labeled absorbance maxima was generated. For protein binding assays, equilibrium dialysis was performed according to ref (55). The partition coefficient was determined from the amount of compound in the organic phase and in the aqueous phase. The amount of compound in buffer was determined as the combined, volume-corrected, and weighted areas of the corresponding peaks in the aqueous phases of three organic-aqueous samples of different compositions following ref (55). In the Caco-2 bidirectional permeability assay, the compounds were tested according to ref (56) in the human colon carcinoma cell line Caco-2. For the intrinsic clearance, the remaining tested compounds in the human liver microsomes (0.1 mg/mL) were quantified using HPLC-MS after 0, 15, 30, 45, and 60 min based in ref (57).

#### Data Analysis

All the assay data was analyzed with Prism 9.3.1 (Graphpad, San Diego, CA). The affinity (p*K*_i_) of the potential antagonists in NanoBRET ligand binding assay was determined by fitting the baseline-corrected BRET ratio response curve with the “one-site *K*_i_ model” based on the Cheng and Prusoff equation^58^ with both the concentration (HotNM) and *K*_d_ (HotKDNM) values of the “Hot ligand” **CA200645** set to 5 nM for hA_3_R and HotNM/HotKDNM as 70 nM/100 nM or 100 nM/300 nM for **CA200623** at hA_3_R or rA_3_R. For the A_3_R mutants, radioligandNM/HotKDNM was input as the corresponding *K*_d_ values shown in Table 4. Receptor binding kinetics was determined as described previously^25,32^ using the Motulsky and Mahan method^59^ (built into Prism 9.3.1) to determine the test compound association rate constant and dissociation rate constant using rate constants previously described.^25^ In the cAMP competition experiments, the responses were normalized to the response of 100 μM **forskolin** as maximum and fitted with a three-parameter logistics equation. p*K*_B_ values for the potential antagonists were determined based on eq 1
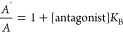
1where *A*′ and *A* are the EC_50_ of the response induced by **NECA** with the presence of the antagonist or DMSO control and *K*_B_ = the affinity of the antagonist used.^58^

All the statistical significance (*p < 0.05) was calculated by nonparametric Kruskal–Wallis test or one-way ANOVA with a Dunnett’s multiple comparison test and determined as described in ref (60).

The data analysis of in vitro pharmacokinetic assessment was performed by Eurofins Panlabs (MO, USA). In the protein binding assay, % protein binding and % recovery were determined using eqs 2 and 3
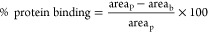
2
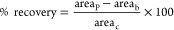
3where area = peak area of the analyte in the protein matrix(p), buffer(b), and control sample(c).

The apparent permeability coefficient (*P*_app_) and % recovery of the test compounds were calculated using eqs 4 and (5)
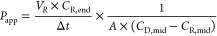
4
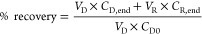
5where *V*_R/D_ is the volume of the receiver/donor chamber. *C*_R/D,end_ is the concentration of the test compound in the receiver/donor chamber at the end time point, Δ*t* is the incubation time, and *A* is the surface area of the cell monolayer. *C*_R/D,mid_ is the calculated midpoint concentration of the test compound in the receiver/donor side. *C*_D0_ is the concentration of the test compound in the donor sample at time zero.

For the intrinsic clearance, the half-life (*t*_1/2_) was determined from the slope of the initial linear range of the logarithmic curve of compound remaining (%) against time, assuming the first-order kinetics. Also, the apparent intrinsic clearance (CLint) was calculated using eq 6
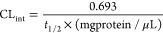
6

### Computational Medicinal Chemistry

#### Preparation of Model of the Unresolved Inactive hA_3_R

Residues are described by their amino acid identity (single letter code) and position (amino acid number) within the specific GPCR with the Ballesteros and Weinstein numbering,^61^ a scheme for class A GPCRs, whereby X.50 represents the defined centrally conserved residue on helix X, in superscript. Αll His residues were protonated on the Nε.^62^

We used for the inactive hA_3_R a ML-based model derived from GPCRdb web-tool^43^ that contains predictions for GPCRs in active and inactive forms via the advanced multistate AF2 method^41,42^ of hA_3_R. We revised this model of the inactive state of hA_3_R by changing the orientation of R173^5.34^, M172,^5.33^ and M174^5.35^ as we previously described.^40^

We superimposed the experimental crystal structure **ZM241385**—A_2A_R complex (PDB ID 3EML)^63^ to our revised^40^ ΑF2 model of WT hA_3_R model N(1.32)–H(7.75). (Residue numbers in parentheses refer to the Ballesteros–Weinstein numbering.^64^) Then, the A_2A_R protein (PDB ID 3EML)^63^ and the crystal waters were removed resulting in the AF2 model of WT hA_3_R in complex with **ZM241385** which was used as a template for the docking calculations. The model of hA_3_R in complex with **ZM241385** was optimized using the Protein Preparation Wizard in Schrödinger suite 2021 (Protein Preparation Wizard; Epik, Schrödinger, LLC, New York, NY, 2021)^65^ as we previously described.^32^

#### Molecular Docking Calculations

Ligands **37-61** and **74-77** preparation was achieved as described in ref (32). Molecular docking calculations were performed using the induced-fit docking protocol of Schrödinger suite 2021 (Induced-fit Docking, Schrödinger, LLC, New York, NY, 2021) in a standard protocol (standard precision) which allows flexibility of both the ligand and the entire binding site. The AF2 model of the WT hA_3_R model in complex with **ZM241385** was used as a template structure. Thus, the grid boxes for the binding site were built considering the coordinates of **ZM241385**. Docking of compounds **37-61** and **74-77** was performed using a softened potential in which the van der Waals scaling factor was set at 0.5 for both receptor and ligand. The Prime refinement step was set on side chain prediction of amino acid residues within 5 Å of the ligand. Subsequently, a minimization of the same set of residues and the ligand for each protein/ligand complex pose was performed. After this stage, any receptor structure in each pose reflects an induced fit to the ligand structure and conformation. For each ligand docked, a maximum of 20 poses was retained. The binding conformations of compounds **37-61** and **74-77** were analyzed, and the top-scoring docking poses were used for the MD simulations to investigate the binding profile of the tested compounds to the inactive hA_3_R.

#### MD Simulations

Each complex of ligands **K18**, **37**–**39**, **56**, **57**, and **60** with hA_3_R from docking calculations was inserted in a pre-equilibrated hydrated POPC membrane bilayer according to the Orientation of Proteins in Membranes (OPM) database.^66^ The orthorhombic periodic box was set 12 Å away from the protein, and the 10 × 10 × 18 Å box consisted ca. 130 lipids and 13,000 TIP3P water molecules,^67^ using the System Builder utility of Desmond v4.9 (Schrödinger Release 2021-1: Desmond Molecular Dynamics System, D. E. Shaw Research, New York, NY, 2021. Maestro-Desmond Interoperability Tools, Schrödinger, New York, NY, 2021). Sodium and chloride ions were added randomly in the water phase to neutralize the systems and reach the experimental salt concentration of 0.150 M NaCl. The total number of atoms of the complex was approximately 75,000, and the simulation box dimensions was 71 × 81 × 105 Å^3^. We used the LEaP, the main program for preparing simulations in Amber Software, antechamber, and Parmchk2 of AmberTools22 to assign the amberff19sb force field parameters^39^ for the calculation of the protein and intermolecular interactions, lipids21 parameters for lipids,^68^ the Generalized Amber Force Field parameters for the ligands,^69^ and the TIP3P model for waters and ions.^70−72^ Partial charges for ligands were obtained using RESP^73^ fitting of the electrostatic potentials calculated with Gaussian03^74^ at the Hartree–Fock/6-31G*^75^ level of theory and the antechamber of AmberTools22.^76^

MD simulation protocol starts with energy minimization by applying 2500 steps of steepest descent to remove bad contacts and 7500 steps of conjugated gradient minimization in the presence of a harmonic restraint with a force constant of 5 kcal mol^–1^ Å^–2^ on all atoms of protein and ligand and a nonbonded cutoff of 12.0 Å. The next stage in the MD simulation protocol is to allow the system to heat up from 0 to 310 K using the Langevin thermostat (dynamics)^77^ for temperature control, as implemented in the Amber22 program,^78^ employing a Langevin collision frequency of 2.0 ps. Heating was accomplished in two consecutive steps in the presence of a harmonic restraint with a force constant of 10 kcal mol^–1^ Å^–2^ on all membrane, protein, and ligand atoms. In the first step, systems were heated to 100 K in a *NVT* of 50 ps length. In the second step, the temperature was raised to 310 K in a *NPT*γ (with γ = 10 dyn cm^–1^) simulation of 500 ps length. The Berendsen barostat^79^ was used to adjust the density over the 500 ps simulation at constant pressure (*NPT*γ) (with γ = 10 dyn cm^–1^), with a target pressure of 1 bar and a 2 ps pressure relaxation time. Subsequently, the systems were equilibrated without restraints in a *NPT*γ simulation of 1 ns length with *T* = 310 K and γ = 10 dyn cm^–1^. In the *NPT*γ simulations, semiisotropic pressure scaling to *p* = 1 bar was applied using a pressure relaxation time of 1.0 ps. The temperature of 310 K was used in MD simulations to ensure that the membrane state is above the main phase transition temperature of 298 K for POPC bilayers.^80^

Bonds involving hydrogen atoms were constrained by the SHAKE algorithm,^81^ and a time step of 2 fs was used for the integration of the equations of motion. Long-range electrostatics were calculated using the Particle mesh Ewald summation,^79^ with a 1 Å grid, and short-range nonbonding interactions were truncated at 12 Å with a continuum model long-range correction applied for energy and pressure.

The equilibration phase was followed by production MD simulation for 500 ns for 7 representative ligands (**K18**, **37–39**, **56**, **57**, and **60**) using the same protocol as in the final equilibration step. Snapshots were recorded every 100 ps during the production phase. Within the 500 ns MD simulation time, the total energy and rmsd of the protein backbone Cα atoms reached a plateau, and the systems were considered equilibrated and suitable for statistical analysis (see Figure 1, 4, S5, and S6).

Two MD simulation repeats were performed for each complex using the same starting structure and applying randomized velocities. The visualization of the MD simulation trajectories was performed using VMD.^82^ We used *ptraj* and cpptraj^83^ of AmberTools22,^76^ MDAnalysis,^84,85^ and Matplotlib programs^86^ and ProLIF,^87^ NumPy^88^ libraries to perform analysis of the MD simulations trajectories.

Particle mesh Ewald molecular dynamics (PMEMD) is the primary engine for running MD simulations with AMBER22 software.^76^ All the MD simulations with AMBER22 software^76^ were run on GTX 3070i GPUs in lab workstations. The pmemd.CUDA executable provides the ability to use NVIDIA GPUs to run the MD simulations.

#### Alchemical TI/MD Binding Free Energies Calculated with the MBAR Method

For the TI/MD calculations, the relaxed complex of compound **37** at hA_3_R from the 500 ns MD simulations in a POPC lipid bilayer with the ff19sb^39^ was used as the reference structure for the calculations. Thus, binding poses of ligands aligned with **37**-inactive hA_3_R complexes were used as starting structures for the alchemical calculations with our optimized^40^ multistate AF2 method^41,42^ of hA_3_R generated from GPCRdb^43^ web-tool. These alchemical perturbations are described in Table S1. We found that the final snapshots from the 2 ns TI/MD simulations matched the final snapshots of relevant ligands from the 500 ns MD simulations (compounds **K18**, **37–39**, **56**, **57**, and **60**); the ligand did not change its binding pose during the much longer MD simulations. Thus, the TI/MD binding free energy simulations calculated the binding free energy change of the binding poses between the examined two ligands without any of the ligands changing its conformation inside the receptor. TI/MD calculations were also performed for the ligands in solution.

The calculation of the relative binding free energies ΔΔA_b,0→1_ or ΔΔA_b,0,1_ for two ligands 0 and 1 bound to A_3_R (for the 23 pairs of ligands shown in Table S1) can be performed using the MBAR method^89^ and applying a thermodynamic cycle,^90−92^ i.e., using the Δ*A* values obtained for the alchemical transformations of the ligands in the bound (b) and the solvent (s; water) state Δ*A*_b,0,1_ and Δ*A*_s,0,1_(s), respectively, according to eq 7
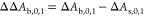
7

The TI estimator computes the free energy change of the transformation 0 → 1 Δ*A*_0→1_ or Δ*A*_0,1_ by integrating the Boltzmann averaged d*U*(λ)/dλ as is described in eqs 8 and 9
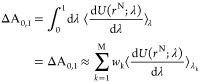
8
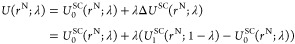
9

MBAR^89^ calculates the free energy difference between neighboring intermediate states Δ*A*_*λ*→λ+1_ using eq 10
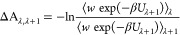
10where *w* is a function of *Α*(λ) and *Α*(λ+1). The equation is solved iteratively to give the free energy change of neighboring states Δ*A*_*λ*→λ+1_ which via combination yield the overall free energy change. The MBAR method has been shown to minimize the variance in the calculated free energies, by making more efficient use of the simulation data.^89,93−95^ Details of the TI/MD theory^17^ and the TI/MD protocol^32^ have been described.

Experimental relative binding free energies were estimated using the experimental binding affinities p*K*_d_ in Table S1 according to eq 11
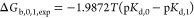
11

### Chemistry

#### General Information

Melting points were determined on a Büchi apparatus and are uncorrected. ^1^H NMR and ^13^C NMR spectra were recorded on a Bruker AVANCE III 600 or a Bruker AVANCE DRX 400 instrument in deuterated solvents and were referenced to TMS (δ scale) (Figure S8). Mass spectra were recorded with a LTQ Orbitrap Discovery instrument, possessing an Ionmax ionization source. Flash chromatography was performed on Merck silica gel 60 (0.040–0.063 mm). Analytical thin-layer chromatography (TLC) was carried out on precoated (0.25 mm) Merck silica gel F-254 plates. The purity of the target derivatives (>95%) was determined on a Thermo Finnigan HPLC System (P4000 Pump, AS3000 Autosampler, UV Spectra System UV6000LP detector, Chromquest 4.1 Software); Phenomenex HYPERSIL C18-BDS (250 mm, 4.0 mm, 5 μm); mobile phase: Method A: 0.2% formic acid in water/acetonitrile; flow rate 0.8 mL/min or Method B: 1% formic acid in water/acetonitrile/methanol (9:1); flow rate 1 mL/min; column temperature 25 °C; injection volume 5 μL (Table S9 and Figure S9).

##### **6-Chloro-9-methyl-9H-purine (15) and 6-Chloro-7-methyl-7H-purine (16)**

Sodium hydride (60% dispersion in mineral oil, 1.1 g, 27.5 mmol) was added in two portions into a suspension of 6-chloropurine (**14**, 3 g, 19.39 mmol) in anhydrous *N*,*N*-dimethylformamide (40 mL) at 0 °C, and this mixture was stirred at r.t. under argon for 1 h. Then, the reaction was cooled at 0 °C, iodomethane (1.8 mL, 28.86 mmol) was added, and this mixture was stirred at r.t. for 20 h. Upon completion of the reaction, the mixture was diluted with water and extracted with dichloromethane (4 × 250 mL), and the combined organic layers were extracted with brine (2 × 400 mL), dried over sodium sulfate, and evaporated. The crude mixture was purified by column chromatography using a mixture of cyclohexane/ethyl acetate as the eluent (from 40/60 up to 10/90, v/v) to provide the pure isomers **15** and **16**.

Data for **15**: Yield 58%. White solid, mp 156–157 °C (CH_2_Cl_2_/*n*-pentane) (reported^96^ 142–143 °C). ^1^H NMR (600 MHz, CDCl_3_): δ 8.68 (s, 1H), 8.07 (s, 1H), 3.89 (s, 3H). ^13^C NMR (151 MHz, CDCl_3_): δ 152.3, 152.1, 151.0, 145.8, 131.6, 30.4.

Data for **16**: Yield 34%. White solid, mp 209 °C (CH_2_Cl_2_/*n*-pentane) (reported^96^ 180–182 °C). ^1^H NMR (600 MHz, CDCl_3_): δ 8.88 (s, 1H), 8.17 (s, 1H), 4.17 (s, 3H). ^13^C NMR (151 MHz, CDCl_3_): δ 162.1, 152.7, 149.6, 143.6, 123.2, 34.5.

#### **9-Methyl-9*H*-purine-6-carbonitrile (17)**

Zinc cyanide (83 mg, 0.71 mmol), zinc dust (8.6 mg, 0.13 mmol), tris(dibenzylideneacetone)dipalladium(0) (Pd_2_(dba)_3_, 22 mg, 0.024 mmol), and 1,1′-ferrocenediyl-bis(diphenylphosphine) (dppf, 26 mg, 0.049 mmol) were added into a solution of the chloroderivative **15** (200 mg, 1.19 mmol) in anhydrous *N*,*N*-dimethylacetamide (1.5 mL), and this mixture was refluxed, under argon, for 3 h. Upon completion of the reaction, the mixture was filtered through a Celite pad and washed with dichloromethane. The filtrate was diluted with water and extracted with dichloromethane (4 × 20 mL), and the combined organic layers were extracted with brine (2 × 50 mL), dried over sodium sulfate, and evaporated. The crude product was purified by column chromatography using a mixture of cyclohexane/ethyl acetate as the eluent (from 70/30 up to 20/80, v/v) to provide the pure nitrile **17** (150 mg, yield 79%) as a brown solid. mp 171–172 °C (CH_2_Cl_2_/*n*-pentane), reported^97^ 153–154 °C. ^1^H NMR (600 MHz, CDCl_3_): δ 9.06 (s, 1H), 8.30 (s, 1H), 3.99 (s, 3H). ^13^C NMR (151 MHz, CDCl_3_): δ 153.5, 152.9, 149.0, 135.1, 131.0, 113.8, 30.6.

### General Procedure for the Preparation of the Arylamidoximes **18–30**

### Method A

An aqueous solution of hydroxylamine (50 wt %, 0.75 mL, 11.4 mmol) was added into a solution of the corresponding arylnitrile (4 mmol) in ethanol (20 mL), and this mixture was refluxed for 2 h. Upon completion of the reaction, the solvent was evaporated at half volume, and the solid was filtered under vacuum, washed with a small amount of ethanol, and air-dried. The solid product was collected and recrystallized to provide the pure amidoxime.

### Method B

Hydroxylamine hydrochloride (417 mg, 6 mmol) and sodium bicarbonate (504 mg, 6 mmol) were added into a solution of the corresponding arylnitrile (4 mmol) in ethanol (20 mL), and this mixture was stirred at room temperature for 90 min and then refluxed for 2 h. Upon completion of the reaction, the solvent was evaporated, water was added into the flask, and the solid was filtered under vacuum, washed with water, and air-dried. The solid product was collected and recrystallized to provide the pure amidoxime.

### (*Z*)-*N*′-Hydroxypicolinimidamide (**18**)

This compound was prepared according to method A, starting from pyridine-2-carbonitrile (**1**). Yield 82%. White solid, mp 128 °C (EtOH) (reported^98^ 119–120 °C). ^1^H NMR (600 MHz, DMSO-*d*_6_): δ 9.88 (br s, 1H, D_2_O exch.), 8.56 (d, *J* = 4.6 Hz, 1H), 7.86 (d, *J* = 8.0 Hz, 1H), 7.82–7.78 (m, 1H), 7.39 (dd, *J* = 6.5, 5.4 Hz, 1H), 5.82 (br s, 2H, D_2_O exch.). ^13^C NMR (151 MHz, DMSO-*d*_6_): δ 150.0, 149.4, 148.2, 136.5, 124.0, 119.3.

### (*Z*)-3,5-Dichloro-*N*′-hydroxypicolinimidamide (**19**)

This compound was prepared according to method B, starting from 3,5-dichloropyridine-2-carbonitrile (**2**). Yield 88%. White solid, mp 151–152 °C (EtOH). ^1^H NMR (400 MHz, DMSO-*d*_6_): δ 9.90 (br s, 1H, D_2_O exch.), 8.64 (d, *J* = 1.9 Hz, 1H), 8.28 (d, *J* = 1.9 Hz, 1H), 5.87 (br s, 2H, D_2_O exch.). ^13^C NMR (151 MHz, DMSO-*d*_6_): δ 148.9, 147.5, 145.7, 137.8, 130.8, 130.0.

### (*Z*)-5-Bromo-*N*′-hydroxypicolinimidamide (**20**)

This compound was prepared according to method A, starting from 5-bromopyridine-2-carbonitrile (**3**). Yield 76%. White solid, mp 181–182 °C (EtOH) (reported^99^ 162–164 °C). ^1^H NMR (600 MHz, DMSO-*d*_6_): δ 10.02 (br s, 1H, D_2_O exch.), 8.68 (dd, *J* = 2.4, 0.7 Hz, 1H), 8.04 (dd, *J* = 8.6, 2.4 Hz, 1H), 7.81 (dd, *J* = 8.6, 0.6 Hz, 1H), 5.83 (br s, 2H, D_2_O exch.). ^13^C NMR (151 MHz, DMSO-*d*_6_): δ 148.89, 148.87, 139.2, 121.1, 120.3.

### (*Z*)-*N*′-Hydroxy-6-methylpicolinimidamide (**21**)

This compound was prepared according to method A, starting from 6-methylpyridine-2-carbonitrile (**4**). Yield 80%. White solid, mp 152–153 °C (EtOH) (reported^100^ 179–180 °C). ^1^H NMR (400 MHz, DMSO-*d*_6_): δ 9.84 (br s, 1H, D_2_O exch.), 7.69–7.65 (m, 2H), 7.25 (dd, *J* = 7.0, 1.2 Hz, 1H), 5.78 (br s, 2H, D_2_O exch.), 2.49 (s, 3H, overlapping with DMSO-*d*_6_). ^13^C NMR (151 MHz, DMSO-*d*_6_): δ 156.6, 149.5, 149.2, 136.8, 123.2, 116.3, 23.9.

### (*Z*)-*N*′-Hydroxypyrimidine-2-carboximidamide (**22**)

This compound was prepared according to method A, starting from pyrimidine-2-carbonitrile (**5**). Yield 93%. White solid, mp 228–229 °C (EtOH) (reported^101^ 211–212 °C). ^1^H NMR (600 MHz, DMSO-*d*_6_): δ 10.15 (br s, 1H, D_2_O exch.), 8.83 (d, *J* = 4.8 Hz, 2H), 7.50 (t, *J* = 4.8 Hz, 1H), 5.81 (br s, 2H, D_2_O exch.). ^13^C NMR (151 MHz, DMSO-*d*_6_): δ 158.3, 157.2, 149.0, 121.1.

### (*Z*)-2-Chloro-*N*′-hydroxynicotinimidamide (**23**)

This compound was prepared according to method A, starting from 2-chloropyridine-3-carbonitrile (**6**). Yield 79%. White solid, mp 165–166 °C (EtOH) (reported^101^ 139–140 °C). ^1^H NMR (600 MHz, DMSO-*d*_6_): δ 9.59 (br s, 1H, D_2_O exch.), 8.43 (dd, *J* = 4.8, 1.9 Hz, 1H), 7.84 (dd, *J* = 7.5, 1.9 Hz, 1H), 7.46–7.43 (m, 1H), 5.93 (br s, 2H, D_2_O exch.). ^13^C NMR (151 MHz, DMSO-*d*_6_): δ 149.7, 149.5, 149.0, 140.2, 130.3, 122.8.

### (*Z*)-*N*′-Hydroxy-6-methylnicotinimidamide (**24**)

This compound was prepared according to method A, starting from 6-methylpyridine-3-carbonitrile (**7**). Yield 86%. White solid, mp 184–185 °C (EtOH) (reported^100^ 172–173 °C). ^1^H NMR (600 MHz, DMSO-*d*_6_): δ 9.71 (br s, 1H, D_2_O exch.), 8.72 (d, *J* = 2.1 Hz, 1H), 7.89 (dd, *J* = 8.1, 2.3 Hz, 1H), 7.25 (d, *J* = 8.1 Hz, 1H), 5.88 (br s, 2H, D_2_O exch.), 2.47 (s, 3H). ^13^C NMR (151 MHz, DMSO-*d*_6_): δ 158.3, 149.0, 145.9, 133.1, 126.2, 122.4, 23.8.

### (*Z*)-*N*′-Hydroxynicotinimidamide (**25**)

This compound was prepared according to method B, starting from pyridine-3-carbonitrile (**8**). Yield 85%. White solid, mp 138 °C (MeOH/*n*-pentane) (reported^102^ 134–136 °C). ^1^H NMR (400 MHz, DMSO-*d*_6_): δ 9.84 (br s, 1H, D_2_O exch.), 8.86 (dd, *J* = 2.2, 0.7 Hz, 1H), 8.56 (dd, *J* = 4.8, 1.6 Hz, 1H), 8.04–7.98 (m, 1H), 7.42–7.38 (m, 1H), 5.97 (br s, 2H, D_2_O exch.). ^13^C NMR (100 MHz, DMSO-*d*_6_): δ 149.8, 149.0, 146.6, 132.9, 129.1, 123.3.

### (*Z*)-*N*′-Hydroxy-3-methylbenzimidamide (**26**)

This compound was prepared according to method A, starting from 3-methylbenzolocarbonitrile (**9**). Upon completion of the reaction, the solvent was evaporated, and the oily residue was purified by column chromatography, using a mixture of dichloromethane/methanol (from 95/5 up to 90/10, v/v) as the eluent, to provide the pure amidoxime **26** as a pale yellow solid. Yield 96%. mp 94–95 °C (EtOAc/*n*-pentane) (reported^103^ 90–91 °C). ^1^H NMR (400 MHz, DMSO-*d*_6_): δ 9.56 (br s, 1H, D_2_O exch.), 7.49 (s, 1H), 7.45 (d, *J* = 7.7 Hz, 1H), 7.25 (t, *J* = 7.6 Hz, 1H), 7.18 (d, *J* = 7.5 Hz, 1H), 5.74 (br s, 2H, D_2_O exch.), 2.31 (s, 3H). ^13^C NMR (100 MHz, DMSO-*d*_6_): δ 151.0, 137.2, 133.3, 129.5, 128.0, 126.0, 122.6, 21.1.

### (*Z*)-*N*′-Hydroxy-2-(pyridin-2-yl)acetimidamide (**27**)

This compound was prepared according to method B, starting from 2-(pyridin-2-yl)acetonitrile (**10**). Yield 71%. Beige solid, mp 114–115 °C (EtOH) (reported^104^ 112–114 °C). ^1^H NMR (600 MHz, DMSO-*d*_6_): δ 8.92 (br s, 1H, D_2_O exch.), 8.47 (d, *J* = 4.8 Hz, 1H), 7.71 (td, *J* = 7.7, 1.8 Hz, 1H), 7.33 (d, *J* = 7.8 Hz, 1H), 7.23 (dd, *J* = 6.8, 5.1 Hz, 1H), 5.40 (br s, 2H, D_2_O exch.), 3.44 (s, 2H). ^13^C NMR (151 MHz, DMSO-*d*_6_): δ 157.6, 150.8, 148.7, 136.4, 122.9, 121.6, 39.4 (overlapping with DMSO-*d*_6_).

### (*Z*)-*N*′-Hydroxy-2-(pyridin-3-yl)acetimidamide (**28**)

This compound was prepared according to method A, starting from 2-(pyridin-3-yl)acetonitrile (**11**). Yield 78%. Pale yellow solid, mp 173 °C (EtOH) (reported^105^ 153 °C). ^1^H NMR (400 MHz, DMSO-*d*_6_): δ 8.97 (br s, 1H, D_2_O exch.), 8.47 (d, *J* = 1.8 Hz, 1H), 8.41 (dd, *J* = 4.8, 1.5 Hz, 1H), 7.69–7.64 (m, 1H), 7.31 (dd, *J* = 7.7, 4.9 Hz, 1H), 5.50 (br s, 2H, D_2_O exch.), 3.29 (s, 2H). ^13^C NMR (151 MHz, DMSO-*d*_6_): δ 151.5, 149.8, 147.5, 136.1, 133.5, 123.2, 34.3.

### (*Z*)-*N*′-Hydroxy-1-methyl-1*H*-indole-5-carboximidamide (**29**)

This compound was prepared according to method A, starting from 1-methyl-1*H*-indole-5-carbonitrile (**13**). Upon completion of the reaction, the solvent was evaporated, and the residue was purified by column chromatography, using a mixture of dichloromethane/methanol (from 95/5 up to 88/12, v/v) as the eluent, to provide the pure amidoxime **29** as a white solid. Yield 72%. mp 175–176 °C (EtOH) (reported^106^ 169–171 °C). ^1^H NMR (600 MHz, DMSO-*d*_6_): δ 9.36 (br s, 1H, D_2_O exch.), 7.86 (s, 1H), 7.52 (dd, *J* = 8.6, 1.2 Hz, 1H), 7.39 (d, *J* = 8.6 Hz, 1H), 7.32 (d, *J* = 2.9 Hz, 1H), 6.44 (d, *J* = 2.6 Hz, 1H), 5.68 (br s, 2H, D_2_O exch.), 3.78 (s, 3H). ^13^C NMR (151 MHz, DMSO-*d*_6_): δ 152.0, 136.7, 130.2, 127.5, 124.4, 119.1, 117.6, 109.2, 100.9, 32.5.

### (*Z*)-*N*′-Hydroxy-9-methyl-9*H*-purine-6-carboximidamide (**30**)

This compound was prepared according to method B, starting from 9-methyl-9*H*-purine-6-carbonitrile (**17**). Yield 67%. Beige solid, mp 245 °C (CH_2_Cl_2_/*n*-pentane). ^1^H NMR (400 MHz, DMSO-*d*_6_): δ 10.53 (br s, 1H, D_2_O exch.), 8.92 (s, 1H), 8.56 (s, 1H), 6.10 (br s, s2H, D_2_O exch.), 3.85 (s, 3H). ^13^C NMR (151 MHz, DMSO-*d*_6_): δ 152.6, 151.0, 148.7, 147.5, 147.2, 129.6, 29.5.

### General Procedure for the Preparation of the Acyl Chlorides **34–36**

A solution of the corresponding carboxylic acid **31**–**33** (1 mmol) in thionyl chloride (3 mL) was refluxed for 3 h under argon. Then, the solvent was evaporated under reduced pressure, and the resulting acyl chlorides **34**–**36** were used immediately to the next step, with no further purification.

### General Procedure for the Preparation of the Target Derivatives **37–55**

The corresponding amidoxime **18**–**30** (1 mmol) and triethylamine (0.15 mL, 1.1 mmol) were added into a solution of the acyl chloride **34**, **35**, or **36** (1 mmol) in anhydrous tetrahydrofuran (5 mL), under argon, and this reaction mixture was stirred at room temperature for 2–16 h. Upon completion of the reaction, the mixture was diluted with ethyl acetate (40 mL) and extracted with water (40 mL). The aqueous layer was extracted two more times with ethyl acetate (40 mL). The combined organic layers were washed with brine (100 mL), dried over sodium sulfate, and concentrated under reduced pressure. The resulting crude products were recrystallized to provide the pure target derivatives **37**–**55**.

### (*Z*)-*N*′-((3-(2,6-Dichlorophenyl)-5-methylisoxazole-4-carbonyl)oxy)picolinimidamide (**37**)

This compound was prepared according to the general procedure described above, upon reaction of the acyl chloride **34** with amidoxime **18**. Reaction time: 4 h. The crude product was recrystallized from ethyl acetate, to provide the pure derivative **37** in 76% yield, as a beige solid. mp 184–185 °C (EtOAc). ^1^H NMR (400 MHz, DMSO-*d*_6_): δ 8.65 (d, *J* = 4.8 Hz, 1H), 7.95–7.86 (m, 2H), 7.70–7.65 (m, 2H), 7.63–7.52 (m, 2H), 6.99 (br s, 1H, D_2_O exch.), 5.95 (br s, 1H, D_2_O exch.), 2.88 (s, 3H). ^13^C NMR (100 MHz, DMSO-*d*_6_): δ 176.1, 158.3, 158.1, 154.9, 148.9, 147.7, 137.4, 134.3, 132.7, 128.5, 127.3, 126.0, 121.1, 108.3, 13.6. HRMS (ESI) *m*/*z*: calcd for C_17_H_13_Cl_2_N_4_O_3_ [Μ + Η]^+^, 391.0360; found, 391.0354. HPLC analysis (Method A): *t*_R_ = 11.35 min, purity 99.75%.

### (*Z*)-3,5-Dichloro-*N*′-((3-(2,6-dichlorophenyl)-5-methylisoxazole-4-carbonyl)oxy)picolinimidamide (**38**)

This compound was prepared according to the general procedure described above, upon reaction of the acyl chloride **34** with amidoxime **19**. Reaction time: 4 h. The crude product was recrystallized from dichloromethane/diethyl ether, to provide the pure derivative **38** in 87% yield, as a pale yellow solid. mp 190 °C (CH_2_Cl_2_/Et_2_O). ^1^H NMR (600 MHz, DMSO-*d*_6_): δ 8.68 (d, *J* = 1.9 Hz, 1H), 8.38 (d, *J* = 1.9 Hz, 1H), 7.69–7.65 (m, 2H), 7.62–7.58 (m, 1H), 6.57 (br s, 2H, D_2_O exch.), 2.88 (s, 3H). ^13^C NMR (151 MHz, DMSO-*d*_6_): δ 175.8, 158.3, 157.7, 154.7, 146.5, 146.2, 137.6, 134.3, 132.44, 132.38, 130.8, 128.3, 127.3, 108.2, 13.5. HRMS (ESI) *m*/*z*: calcd for C_17_H_11_Cl_4_N_4_O_3_ [Μ + Η]^+^, 458.9580; found, 458.9578. HPLC analysis (Method A): *t*_R_ = 11.52 min, purity 99.00%.

### (*Z*)-5-Bromo-*N*′-((3-(2,6-dichlorophenyl)-5-methylisoxazole-4-carbonyl)oxy)picolinimidamide (**39**)

This compound was prepared according to the general procedure described above, upon reaction of the acyl chloride **34** with amidoxime **20**. Reaction time: 2 h. The crude product was recrystallized from ethyl acetate, to provide the pure derivative **39** in 82% yield, as a white solid. mp 249 °C (EtOAc). ^1^H NMR (600 MHz, DMSO-*d*_6_): δ 8.79 (d, *J* = 2.2 Hz, 1H), 8.17 (dd, *J* = 8.5, 2.3 Hz, 1H), 7.83 (d, *J* = 8.5 Hz, 1H), 7.69–7.65 (m, 2H), 7.61–7.58 (m, 1H), 7.05 (br s, 1H, D_2_O exch.), 6.02 (br s, 1H, D_2_O exch.), 2.88 (s, 3H). ^13^C NMR (151 MHz, DMSO-*d*_6_): δ 176.1, 158.3, 157.9, 154.4, 149.6, 146.6, 140.1, 134.3, 132.7, 128.4, 127.3, 122.7, 122.5, 108.2, 13.6. HRMS (ESI) *m*/*z*: calcd for C_17_H_12_BrCl_2_N_4_O_3_ [Μ + Η]^+^, 468.9465; found, 468.9465. HPLC analysis (Method A): *t*_R_ = 11.87 min, purity 97.21%.

### (*Z*)-*N*′-((3-(2,6-Dichlorophenyl)-5-methylisoxazole-4-carbonyl)oxy)-6-methylpicolinimidamide (**40**)

This compound was prepared according to the general procedure described above, upon reaction of the acyl chloride **34** with amidoxime **21**. Reaction time: 4 h. The crude product was recrystallized from ethyl acetate/*n*-pentane, to provide the pure derivative **40** in 74% yield, as a white solid. mp 167–168 °C (EtOAc/*n*-pentane). ^1^H NMR (600 MHz, DMSO-*d*_6_): δ 7.78 (t, *J* = 7.7 Hz, 1H), 7.69–7.66 (m, 3H), 7.63–7.59 (m, 1H), 7.40 (d, *J* = 7.6 Hz, 1H), 6.76 (br s, 1H, D_2_O exch.), 5.80 (br s, 1H, D_2_O exch.), 2.88 (s, 3H), 2.53 (s, 3H). ^13^C NMR (151 MHz, DMSO-*d*_6_): δ 176.1, 158.1, 158.0, 157.5, 154.8, 146.8, 137.5, 134.2, 132.6, 128.4, 127.3, 125.3, 118.0, 108.3, 23.9, 13.5. HRMS (ESI) *m*/*z*: calcd for C_18_H_15_Cl_2_N_4_O_3_ [Μ + Η]^+^, 405.0516; found, 405.0507. HPLC analysis (Method A): *t*_R_ = 11.69 min, purity 99.49%.

### (*Z*)-*N*′-((3-(2-Chlorophenyl)-5-methylisoxazole-4-carbonyl)oxy)-6-methylpicolinimidamide (**41**)

This compound was prepared according to the general procedure described above, upon reaction of the acyl chloride **35** with amidoxime **21**. Reaction time: 4 h. The crude product was recrystallized from dichloromethane/diethyl ether, to provide the pure derivative **41** in 70% yield, as a white solid. mp 141–142 °C (CH_2_Cl_2_/Et_2_O). ^1^H NMR (600 MHz, DMSO-*d*_6_): δ 7.77 (t, *J* = 7.7 Hz, 1H), 7.68 (d, *J* = 7.8 Hz, 1H), 7.65–7.61 (m, 1H), 7.60–7.56 (m, 2H), 7.51 (t, *J* = 7.1 Hz, 1H), 7.38 (d, *J* = 7.6 Hz, 1H), 6.70 (br s, 1H, D_2_O exch.), 5.43 (br s, 1H, D_2_O exch.), 2.81 (s, 3H), 2.51 (s, 3H, overlapping with DMSO-*d*_6_). ^13^C NMR (151 MHz, DMSO-*d*_6_): δ 175.7, 160.1, 158.5, 157.6, 154.7, 146.8, 137.6, 132.7, 131.9, 131.3, 129.6, 128.2, 127.5, 125.3, 118.1, 108.7, 23.9, 13.2. HRMS (ESI) *m*/*z*: calcd for C_18_H_16_ClN_4_O_3_ [Μ + Η]^+^, 371.0906; found, 371.0905. HPLC analysis (Method A): *t*_R_ = 11.45 min, purity 98.53%.

### (*Z*)-*N*′-((3-(2-Chlorophenyl)-5-methylisoxazole-4-carbonyl)oxy)picolinimidamide (**42**)

This compound was prepared according to the general procedure described above, upon reaction of the acyl chloride **35** with amidoxime **18**. Reaction time: 8 h. The crude product was recrystallized from ethyl acetate/*n*-pentane, to provide the pure derivative **42** in 80% yield, as a beige solid. mp 168 °C (EtOAc/*n*-pentane). ^1^H NMR (600 MHz, DMSO-*d*_6_): δ 8.63 (d, *J* = 4.8 Hz, 1H), 7.92–7.88 (m, 2H), 7.63 (d, *J* = 7.9 Hz, 1H), 7.60–7.56 (m, 2H), 7.55–7.53 (m, 1H), 7.52–7.49 (m, 1H), 2.82 (s, 3H). ^13^C NMR (151 MHz, DMSO-*d*_6_): δ 175.5, 160.0, 158.3, 154.6, 148.7, 147.6, 137.3, 132.6, 131.7, 131.2, 129.5, 128.1, 127.3, 125.8, 121.0, 108.6, 13.1. HRMS (ESI) *m*/*z*: calcd for C_17_H_14_ClN_4_O_3_ [Μ + Η]^+^, 357.0749; found, 357.0743. HPLC analysis (Method A): *t*_R_ = 11.03 min, purity 99.35%.

### (*Z*)-6-Methyl-*N*′-((5-methyl-3-phenylisoxazole-4-carbonyl)oxy)picolinimidamide (**43**)

This compound was prepared according to the general procedure described above, upon reaction of the acyl chloride **36** with amidoxime **21**. Reaction time: 3 h. The crude product was recrystallized from ethyl acetate/*n*-pentane, to provide the pure derivative **43** in 73% yield, as a beige solid. mp 148–149 °C (EtOAc/*n*-pentane). ^1^H NMR (600 MHz, DMSO-*d*_6_): δ 7.79 (t, *J* = 7.7 Hz, 1H), 7.72 (d, *J* = 7.8 Hz, 1H), 7.65 (d, *J* = 6.4 Hz, 2H), 7.55–7.49 (m, 3H), 7.40 (d, *J* = 7.6 Hz, 1H), 6.67 (br s, 1H, D_2_O exch.), 6.18 (br s, 1H, D_2_O exch.), 2.78 (s, 3H), 2.53 (s, 3H). ^13^C NMR (151 MHz, DMSO-*d*_6_): δ 175.6, 161.8, 159.1, 157.4, 155.0, 146.9, 137.5, 130.0, 128.9, 128.4, 128.3, 125.2, 118.1, 107.7, 23.9, 13.3. HRMS (ESI) *m*/*z*: calcd for C_18_H_17_N_4_O_3_ [Μ + Η]^+^, 337.1296; found, 337.1294. HPLC analysis (Method A): *t*_R_ = 11.30 min, purity 99.15%.

### (*Z*)-*N*′-((5-methyl-3-phenylisoxazole-4-carbonyl)oxy)picolinimidamide (**44**)

This compound was prepared according to the general procedure described above, upon reaction of the acyl chloride **36** with amidoxime **18**. Reaction time: 4 h. The crude product was recrystallized from ethyl acetate, to provide the pure derivative **44** in 75% yield, as a beige solid. mp 169 °C (EtOAc). ^1^H NMR (400 MHz, DMSO-*d*_6_): δ 8.65 (dt, *J* = 4.8, 1.3 Hz, 1H), 7.96–7.88 (m, 2H), 7.69–7.64 (m, 2H), 7.57–7.48 (m, 4H), 6.84 (br s, 1H, D_2_O exch.), 6.30 (br s, 1H, D_2_O exch.), 2.78 (s, 3H). ^13^C NMR (151 MHz, DMSO-*d*_6_): δ 175.5, 161.8, 159.0, 155.0, 148.7, 147.8, 137.3, 129.9, 128.9, 128.33, 128.26, 125.8, 121.0, 107.7, 13.3. HRMS (ESI) *m*/*z*: calcd for C_17_H_15_N_4_O_3_ [Μ + Η]^+^, 323.1139; found, 323.1156. HPLC analysis (Method A): *t*_R_ = 10.81 min, purity 99.71%.

### (*Z*)-*N*′-((3-(2,6-Dichlorophenyl)-5-methylisoxazole-4-carbonyl)oxy)pyrimidine-2-carboximidamide (**45**)

This compound was prepared according to the general procedure described above, upon reaction of the acyl chloride **34** with amidoxime **22**. Reaction time: 2 h. The crude product was recrystallized from dichloromethane/*n*-pentane, to provide the pure derivative **45** in 73% yield, as a white solid. mp 219–220 °C (CH_2_Cl_2_/*n*-pentane). ^1^H NMR (400 MHz, DMSO-*d*_6_): δ 8.92 (d, *J* = 4.9 Hz, 2H), 7.70–7.64 (m, 3H), 7.63–7.58 (m, 1H), 2.88 (s, 3H). ^13^C NMR (100 MHz, DMSO-*d*_6_): δ 176.2, 158.1, 157.9, 157.7, 157.1, 154.5, 134.3, 132.7, 128.4, 127.3, 122.7, 108.3, 13.5. HRMS (ESI) *m*/*z*: calcd for C_16_H_12_Cl_2_N_5_O_3_ [Μ + Η]^+^, 392.0312; found, 392.0304. HPLC analysis (Method A): *t*_R_ = 10.58 min, purity 99.76%.

### (*Z*)-2-Chloro-*N*′-((3-(2,6-dichlorophenyl)-5-methylisoxazole-4-carbonyl)oxy)nicotinimidamide (**46**)

This compound was prepared according to the general procedure described above, upon reaction of the acyl chloride **34** with amidoxime **23**. Reaction time: 2 h. The crude product was recrystallized from dichloromethane/*n*-pentane, to provide the pure derivative **46** in 80% yield, as a beige solid. mp 191–192 °C (CH_2_Cl_2_/*n*-pentane). ^1^H NMR (400 MHz, DMSO-*d*_6_): δ 8.52 (dd, *J* = 4.8, 1.9 Hz, 1H), 7.93 (dd, *J* = 7.5, 1.9 Hz, 1H), 7.70–7.65 (m, 2H), 7.62–7.57 (m, 1H), 7.50 (dd, *J* = 7.6, 4.8 Hz, 1H), 6.71 (br s, 2H, D_2_O exch.), 2.88 (s, 3H). ^13^C NMR (151 MHz, DMSO-*d*_6_): δ 175.7, 158.4, 157.9, 155.7, 150.9, 148.7, 140.3, 134.3, 132.4, 128.3, 127.9, 127.3, 122.9, 108.3, 13.6. HRMS (ESI) *m*/*z*: calcd for C_17_H_12_Cl_3_N_4_O_3_ [Μ + Η]^+^, 424.9970; found, 424.9969. HPLC analysis (Method A): *t*_R_ = 10.80 min, purity 99.76%.

### (*Z*)-*N*′-((3-(2,6-Dichlorophenyl)-5-methylisoxazole-4-carbonyl)oxy)-6-methylnicotinimidamide (**47**)

This compound was prepared according to the general procedure described above, upon reaction of the acyl chloride **34** with amidoxime **24**. Reaction time: 8 h. The crude product was recrystallized from ethyl acetate, to provide the pure derivative **47** in 77% yield, as a pale yellow solid. mp 195–196 °C (EtOAc). ^1^H NMR (400 MHz, DMSO-*d*_6_): δ 8.71 (d, *J* = 1.9 Hz, 1H), 7.92 (dd, *J* = 8.1, 2.4 Hz, 1H), 7.70–7.66 (m, 2H), 7.63–7.58 (m, 1H), 7.33 (d, *J* = 8.1 Hz, 1H), 6.51 (br s, 2H, D_2_O exch.), 2.87 (s, 3H), 2.51 (s, 3H, overlapping with DMSO-*d*_6_). ^13^C NMR (100 MHz, DMSO-*d*_6_): δ 176.0, 160.5, 158.3, 158.1, 155.7, 147.0, 134.7, 134.3, 132.6, 128.4, 127.4, 124.2, 122.8, 108.3, 24.0, 13.6. HRMS (ESI) *m*/*z*: calcd for C_18_H_15_Cl_2_N_4_O_3_ [Μ + Η]^+^, 405.0516; found 405.0516. HPLC analysis (Method B): *t*_R_ = 8.64 min, purity 99.29%.

### (*Z*)-*N*′-((3-(2,6-Dichlorophenyl)-5-methylisoxazole-4-carbonyl)oxy)nicotinimidamide (**48**)

This compound was prepared according to the general procedure described above, upon reaction of the acyl chloride **34** with amidoxime **25**. Reaction time: 4 h. The crude product was recrystallized from dichloromethane/*n*-pentane, to provide the pure derivative **48** in 83% yield, as a beige solid. mp 183 °C (CH_2_Cl_2_/*n*-pentane). ^1^H NMR (400 MHz, DMSO-*d*_6_): δ 8.84 (dd, *J* = 2.2, 0.7 Hz, 1H), 8.69 (dd, *J* = 4.8, 1.6 Hz, 1H), 8.06–8.02 (m, 1H), 7.70–7.66 (m, 2H), 7.63–7.58 (m, 1H), 7.51–7.46 (m, 1H), 6.61 (br s, 2H, D_2_O exch.), 2.88 (s, 3H). ^13^C NMR (151 MHz, DMSO-*d*_6_): δ 176.0, 158.3, 158.1, 155.7, 151.6, 147.6, 134.6, 134.3, 132.6, 128.4, 127.4, 127.0, 123.5, 108.3, 13.6. HRMS (ESI) *m*/*z*: calcd for C_17_H_13_Cl_2_N_4_O_3_ [Μ + Η]^+^, 391.0360; found, 391.0359. HPLC analysis (Method A): *t*_R_ = 10.85 min, purity 99.70%.

### (*Z*)-*N*′-((3-(2,6-Dichlorophenyl)-5-methylisoxazole-4-carbonyl)oxy)-3-methylbenzimidamide (**49**)

This compound was prepared according to the general procedure described above, upon reaction of the acyl chloride **34** with amidoxime **26**. Reaction time: 2 h. The crude product was recrystallized from ethyl acetate, to provide the pure derivative **49** in 90% yield, as a white solid. mp 197–198 °C (EtOAc). ^1^H NMR (600 MHz, DMSO-*d*_6_): δ 7.69–7.66 (m, 2H), 7.62–7.59 (m, 1H), 7.49 (s, 1H), 7.46–7.44 (m, 1H), 7.34–7.30 (m, 2H), 6.23 (br s, 2H, D_2_O exch.), 2.87 (s, 3H), 2.33 (s, 3H). ^13^C NMR (151 MHz, DMSO-*d*_6_): δ 175.9, 158.1, 157.3, 137.7, 134.3, 132.6, 131.3, 130.9, 128.4, 128.3, 127.5, 127.3, 123.9, 108.4, 20.9, 13.5. HRMS (ESI) *m*/*z*: calcd for C_19_H_16_Cl_2_N_3_O_3_ [Μ + Η]^+^, 404.0564; found, 404.0558. HPLC analysis (Method A): *t*_R_ = 11.60 min, purity 98.45%.

### (*Z*)-3-Methyl-*N*′-((5-methyl-3-phenylisoxazole-4-carbonyl)oxy)benzimidamide (**50**)

This compound was prepared according to the general procedure described above, upon reaction of the acyl chloride **36** with amidoxime **26**. Reaction time: 2 h. The crude product was recrystallized from ethyl acetate, to provide the pure derivative **50** in 81% yield, as a white solid. mp 163–164 °C (EtOAc). ^1^H NMR (600 MHz, DMSO-*d*_6_): δ 7.67–7.63 (m, 2H), 7.56–7.50 (m, 4H), 7.47 (d, *J* = 6.4 Hz, 1H), 7.34–7.30 (m, 2H), 6.32 (br s, 2H, D_2_O exch.), 2.76 (s, 3H), 2.34 (s, 3H). ^13^C NMR (151 MHz, DMSO-*d*_6_): δ 175.5, 161.8, 159.2, 157.5, 137.6, 131.2, 131.1, 129.9, 128.9, 128.42, 128.38, 128.26, 127.3, 123.9, 107.9, 20.9, 13.3. HRMS (ESI) *m*/*z*: calcd for C_19_H_18_N_3_O_3_ [Μ + Η]^+^, 336.1343; found, 336.1347. HPLC analysis (Method A): *t*_R_ = 11.21 min, purity 98.94%.

### (*Z*)-*N*′-((3-(2-Chlorophenyl)-5-methylisoxazole-4-carbonyl)oxy)-3-methylbenzimidamide (**51**)

This compound was prepared according to the general procedure described above, upon reaction of the acyl chloride **35** with amidoxime **26**. Reaction time: 3 h. The crude product was recrystallized from ethyl acetate, to provide the pure derivative **51** in 86% yield, as a white solid. mp 176–177 °C (EtOAc). ^1^H NMR (400 MHz, DMSO-*d*_6_): δ 7.67–7.63 (m, 1H), 7.61–7.56 (m, 2H), 7.54–7.50 (m, 1H), 7.48 (s, 1H), 7.46–7.42 (m, 1H), 7.34–7.28 (m, 2H), 6.05 (br s, 2H, D_2_O exch.), 2.81 (s, 3H), 2.32 (s, 3H). ^13^C NMR (100 MHz, DMSO-*d*_6_): δ 175.5, 160.0, 158.5, 157.2, 137.7, 132.7, 131.7, 131.30, 131.25, 130.9, 129.5, 128.29, 128.26, 127.34, 127.26, 123.9, 108.8, 20.9, 13.2. HRMS (ESI) *m*/*z*: calcd for C_19_H_17_ClN_3_O_3_ [Μ + Η]^+^, 370.0953; found, 370.0957. HPLC analysis (Method A): *t*_R_ = 11.35 min, purity 99.82%.

### (*Z*)-*N*′-((3-(2,6-Dichlorophenyl)-5-methylisoxazole-4-carbonyl)oxy)-1-methyl-1*H*-indole-5-carboximidamide (**52**)

This compound was prepared according to the general procedure described above, upon reaction of the acyl chloride **34** with amidoxime **29**. Reaction time: 16 h. The crude product was recrystallized from dichloromethane/diethyl ether, to provide the pure derivative **52** in 82% yield, as a white solid. mp 188 °C (CH_2_Cl_2_/Et_2_O). ^1^H NMR (600 MHz, DMSO-*d*_6_): δ 7.91 (s, 1H), 7.70–7.66 (m, 2H), 7.63–7.60 (m, 1H), 7.49–7.45 (m, 2H), 7.38 (d, *J* = 3.0 Hz, 1H), 6.49 (d, *J* = 3.0 Hz, 1H), 6.13 (br s, 2H, D_2_O exch.), 3.80 (s, 3H), 2.88 (s, 3H). ^13^C NMR (100 MHz, DMSO-*d*_6_): δ 176.0, 158.29, 158.28, 158.15, 137.5, 134.3, 132.6, 130.9, 128.5, 127.6, 127.5, 121.5, 119.8, 119.4, 109.6, 108.6, 101.2, 32.6, 13.5. HRMS (ESI) *m*/*z*: calcd for C_21_H_17_Cl_2_N_4_O_3_ [Μ + Η]^+^, 443.0673; found, 443.0666. HPLC analysis (Method A): *t*_R_ = 11.50 min, purity 97.63%.

### (*Z*)-*N*′-((3-(2,6-Dichlorophenyl)-5-methylisoxazole-4-carbonyl)oxy)-9-methyl-9*H*-purine-6-carboximidamide (**53**)

This compound was prepared according to the general procedure described above, upon reaction of the acyl chloride **34** with amidoxime **30**. Reaction time: 16 h. The crude product was recrystallized from dichloromethane/diethyl ether, to provide the pure derivative **53** in 74% yield, as a white solid. mp 222–223 °C (CH_2_Cl_2_/Et_2_O). ^1^H NMR (600 MHz, DMSO-*d*_6_): δ 9.01 (s, 1H), 8.65 (s, 1H), 7.69–7.67 (m, 2H), 7.63–7.59 (m, 1H), 6.64 (br s, 2H, D_2_O exch.), 3.87 (s, 3H), 2.91 (s, 3H). ^13^C NMR (151 MHz, DMSO-*d*_6_): δ 176.0, 158.3, 157.7, 154.2, 153.1, 151.1, 148.6, 145.4, 134.3, 132.5, 130.6, 128.4, 127.3, 108.2, 29.7, 13.5. HRMS (ESI) *m*/*z*: calcd for C_18_H_14_Cl_2_N_7_O_3_ [Μ + Η]^+^, 446.0530; found, 446.0539. HPLC analysis (Method A): *t*_R_ = 10.58 min, purity 98.43%.

### (*Z*)-*N*′-((3-(2,6-Dichlorophenyl)-5-methylisoxazole-4-carbonyl)oxy)-2-(pyridin-2-yl)acetimidamide (**54**)

This compound was prepared according to the general procedure described above, upon reaction of the acyl chloride **34** with amidoxime **27**. Reaction time: 2 h. The crude product was recrystallized from ethyl acetate, to provide the pure derivative **54** in 76% yield, as a beige solid. mp 156–157 °C (EtOAc). ^1^H NMR (400 MHz, DMSO-*d*_6_): δ 8.49 (d, *J* = 4.1 Hz, 1H), 7.74 (td, *J* = 7.7, 1.8 Hz, 1H), 7.68–7.62 (m, 2H), 7.61–7.56 (m, 1H), 7.35 (d, *J* = 7.8 Hz, 1H), 7.26 (dd, *J* = 7.0, 5.3 Hz, 1H), 6.05 (br s, 2H, D_2_O exch.), 3.54 (s, 2H), 2.81 (s, 3H). ^13^C NMR (151 MHz, DMSO-*d*_6_): δ 175.6, 158.1, 158.0, 157.5, 156.0, 148.9, 136.7, 134.2, 132.4, 128.3, 127.4, 123.0, 122.0, 108.4, 38.9 (overlapping with DMSO-*d*_6_), 13.4. HRMS (ESI) *m*/*z*: calcd for C_18_H_14_Cl_2_N_4_NaO_3_ [Μ + Na]^+^, 427.0336; found, 427.0331. HPLC analysis (Method B): *t*_R_ = 8.46 min, purity 98.10%.

### (*Z*)-*N*′-((3-(2,6-Dichlorophenyl)-5-methylisoxazole-4-carbonyl)oxy)-2-(pyridin-3-yl)acetimidamide (**55**)

This compound was prepared according to the general procedure described above, upon reaction of the acyl chloride **34** with amidoxime **28**. Reaction time: 16 h. The crude product was recrystallized from ethyl acetate, to provide the pure derivative **55** in 71% yield, as a pale yellow solid. mp 175–176 °C (EtOAc). ^1^H NMR (400 MHz, DMSO-*d*_6_): δ 8.50 (s, 1H), 8.44 (d, *J* = 3.9 Hz, 1H), 7.72–7.62 (m, 3H), 7.60–7.55 (m, 1H), 7.34 (dd, *J* = 7.6, 4.9 Hz, 1H), 3.40 (s, 2H), 2.80 (s, 3H). ^13^C NMR (100 MHz, DMSO-*d*_6_): δ 175.7, 158.3, 158.2, 158.1, 149.8, 147.9, 136.1, 134.3, 132.5, 132.1, 128.4, 127.4, 123.5, 108.4, 33.7, 13.5. HRMS (ESI) *m*/*z*: calcd for C_18_H_15_Cl_2_N_4_O_3_ [Μ + Η]^+^, 405.0516; found, 405.0505. HPLC analysis (Method B): *t*_R_ = 8.17 min, purity 97.87%.

### General Procedure for the Preparation of the Target Derivatives **56–61**

Potassium hydroxide (14 mg, 0.25 mmol) was added into a solution of the corresponding derivative **37**, **39**, **45**, **47**, **48**, or **55** (0.25 mmol) in anhydrous DMSO (0.4 mL), and each reaction mixture was stirred at room temperature for 30–45 min. Upon completion of the reaction, the mixture was diluted with water (30 mL) and extracted with dichloromethane (3 × 30 mL). The combined organic layers were washed with brine (2 × 20 mL), dried over sodium sulfate, and evaporated. The crude products were purified with column chromatography to provide the pure target derivatives **56**–**61**.

### 5-(3-(2,6-Dichlorophenyl)-5-methylisoxazol-4-yl)-3-(pyridin-2-yl)-1,2,4-oxadiazole (**56**)

This compound was prepared according to the general procedure described above, starting from derivative **37**. Reaction time: 30 min. Purification was carried out by using a mixture of cyclohexane/ethyl acetate as the eluent (from 90/10 up to 70/30, v/v) to provide the pure compound **56** in 91% yield, as a white solid. mp 189–190 °C (EtOAc/Et_2_O). ^1^H NMR (400 MHz, CDCl_3_): δ 8.79 (d, *J* = 4.2 Hz, 1H), 7.96 (d, *J* = 7.9 Hz, 1H), 7.83 (t, *J* = 7.7 Hz, 1H), 7.49–7.40 (m, 4H), 3.03 (s, 3H). ^13^C NMR (151 MHz, CDCl_3_): δ 174.5, 169.6, 168.1, 157.8, 150.4, 146.1, 137.7, 136.02, 131.95, 128.3, 127.2, 126.0, 123.7, 104.4, 13.8. HRMS (ESI) *m*/*z*: calcd for C_17_H_11_Cl_2_N_4_O_2_ [Μ + Η]^+^, 373.0254; found, 373.0253. HPLC analysis (Method A): *t*_R_ = 11.74 min, purity 99.59%.

### 3-(5-Bromopyridin-2-yl)-5-(3-(2,6-dichlorophenyl)-5-methylisoxazol-4-yl)-1,2,4-oxadiazole (**57**)

This compound was prepared according to the general procedure described above, starting from derivative **39**. Reaction time: 45 min. Purification was carried out by using a mixture of cyclohexane/ethyl acetate as the eluent (from 90/10 up to 80/20, v/v) to provide the pure compound **57** in 81% yield, as a white solid. mp 209–210 °C (EtOAc/Et_2_O). ^1^H NMR (600 MHz, CDCl_3_): δ 8.82 (d, *J* = 2.0 Hz, 1H), 7.96 (dd, *J* = 8.3, 2.3 Hz, 1H), 7.83 (d, *J* = 8.3 Hz, 1H), 7.49–7.39 (m, 3H), 3.02 (s, 3H). ^13^C NMR (151 MHz, CDCl_3_): δ 174.4, 169.6, 167.7, 157.7, 151.8, 144.7, 139.9, 135.9, 131.9, 128.3, 127.0, 124.5, 123.3, 104.2, 13.7. HRMS (ESI) *m*/*z*: calcd for C_17_H_10_BrCl_2_N_4_O_2_ [Μ + Η]^+^, 450.9359; found, 450.9369. HPLC analysis (Method A): *t*_R_ = 12.42 min, purity 99.91%.

### 5-(3-(2,6-Dichlorophenyl)-5-methylisoxazol-4-yl)-3-(pyrimidin-2-yl)-1,2,4-oxadiazole (**58**)

This compound was prepared according to the general procedure described above, starting from derivative **45**. Reaction time: 30 min. Purification was carried out by using a mixture of cyclohexane/ethyl acetate as the eluent (from 90/10 up to 60/40, v/v) to provide the pure compound **58** in 89% yield as a pale yellow solid. mp 213 °C (CH_2_Cl_2_/*n*-pentane). ^1^H NMR (400 MHz, CDCl_3_): δ 8.90 (d, *J* = 4.9 Hz, 2H), 7.44–7.34 (m, 4H), 3.00 (s, 3H). ^13^C NMR (100 MHz, CDCl_3_): δ 174.5, 170.2, 167.9, 158.1, 157.5, 156.1, 135.7, 132.0, 128.3, 126.7, 122.3, 104.2, 13.7. HRMS (ESI) *m*/*z*: calcd for C_16_H_10_Cl_2_N_5_O_2_ [Μ + Η]^+^, 374.0207; found, 374.0210. HPLC analysis (Method A): *t*_R_ = 11.05 min, purity 99.42%.

### 5-(3-(2,6-Dichlorophenyl)-5-methylisoxazol-4-yl)-3-(pyridin-3-yl)-1,2,4-oxadiazole (**59**)

This compound was prepared according to the general procedure described above, starting from derivative **48**. Reaction time: 30 min. Purification was carried out by using a mixture of cyclohexane/ethyl acetate as the eluent (from 90/10 up to 70/30, v/v) to provide the pure compound **59** in 93% yield as a white solid. mp 183–184 °C (CH_2_Cl_2_/*n*-pentane). ^1^H NMR (400 MHz, CDCl_3_): δ 9.19 (br s, 1H), 8.73 (br s, 1H), 8.27 (dt, *J* = 8.0, 1.8 Hz, 1H), 7.50–7.38 (m, 4H), 3.01 (s, 3H). ^13^C NMR (100 MHz, CDCl_3_): δ 174.4, 169.2, 166.5, 157.8, 152.1, 148.7, 135.9, 135.1, 132.0, 128.3, 127.1, 124.0, 123.1, 104.2, 13.7. HRMS (ESI) *m*/*z*: calcd for C_17_H_11_Cl_2_N_4_O_2_ [Μ + Η]^+^, 373.0254; found, 373.0255. HPLC analysis (Method A): *t*_R_ = 12.31 min, purity 99.54%.

### 5-(3-(2,6-Dichlorophenyl)-5-methylisoxazol-4-yl)-3-(6-methylpyridin-3-yl)-1,2,4-oxadiazole (**60**)

This compound was prepared according to the general procedure described above, starting from derivative **47**. Reaction time: 30 min. Purification was carried out by using a mixture of cyclohexane/ethyl acetate as the eluent (from 90/10 up to 80/20, v/v) to provide the pure compound **60** in 92% yield as a white solid. mp 185–186 °C (CH_2_Cl_2_/*n*-pentane). ^1^H NMR (400 MHz, CDCl_3_): δ 9.04 (d, *J* = 1.7 Hz, 1H), 8.15 (dd, *J* = 8.1, 2.1 Hz, 1H), 7.49–7.40 (m, 3H), 7.26 (d, *J* = 8.0 Hz, 1H, overlapping with CDCl_3_), 3.01 (s, 3H), 2.63 (s, 3H). ^13^C NMR (151 MHz, CDCl_3_): δ 174.3, 169.1, 166.6, 161.6, 157.8, 148.0, 136.0, 135.4, 132.0, 128.3, 127.2, 123.7, 120.4, 104.3, 24.6, 13.7. HRMS (ESI) *m*/*z*: calcd for C_18_H_13_Cl_2_N_4_O_2_ [Μ + Η]^+^, 387.0411; found, 387.0410. HPLC analysis (Method A): *t*_R_ = 14.32 min, purity 99.73%.

### 5-(3-(2,6-Dichlorophenyl)-5-methylisoxazol-4-yl)-3-(pyridin-3-ylmethyl)-1,2,4-oxadiazole (**61**)

This compound was prepared according to the general procedure described above, starting from derivative **55**. Reaction time: 45 min. Purification was carried out by using a mixture of cyclohexane/ethyl acetate as the eluent (from 90/10 up to 65/35, v/v) to provide the pure compound **61** in 84% yield as a pale yellow solid. mp 109–110 °C (CH_2_Cl_2_/*n*-pentane). ^1^H NMR (600 MHz, CDCl_3_): δ 8.53 (s, 1H), 8.51 (d, *J* = 4.1 Hz, 1H), 7.59 (d, *J* = 7.8 Hz, 1H), 7.44–7.36 (m, 3H), 7.23 (dd, *J* = 7.8, 4.9 Hz, 1H), 4.02 (s, 2H), 2.89 (s, 3H). ^13^C NMR (151 MHz, CDCl_3_): δ 174.1, 169.0, 168.8, 157.7, 150.0, 148.4, 137.1, 135.9, 131.9, 131.3, 128.2, 127.1, 123.7, 104.3, 29.7, 13.5. HRMS (ESI) *m*/*z*: calcd for C_18_H_13_Cl_2_N_4_O_2_ [Μ + Η]^+^, 387.0411; found, 387.0411. HPLC analysis (Method A): *t*_R_ = 17.34 min, purity 99.47%.

### General Procedure for the Preparation of the Carboxylic Acids **70** and **71**

Sodium hydroxide (160 mg, 4 mmol) was dissolved in a mixture of methanol (13 mL) and water (13 mL), and then the corresponding methyl ester **68** or **69** (3.4 mmol) was added. This reaction mixture was heated under stirring at 65 °C for 3 h. Upon completion of the reaction, the mixture was poured into cold water (50 mL) and acidified with 1 N HCl until pH 3. The solid was filtered under vacuum and air-dried. The crude product was then recrystallized from methanol to provide the pure derivative **70** or **71**.

### 3-(2,6-Dibromophenyl)-5-methylisoxazole-4-carboxylic Acid (**70**)

This compound was prepared according to the general procedure described above, starting from derivative **68**, in 83% yield, as a white solid. mp 233–235 °C (MeOH). ^1^H NMR (600 MHz, DMSO-*d*_6_): δ 13.06 (br s, 1H, D_2_O exch.), 7.78 (d, *J* = 8.1 Hz, 2H), 7.37 (t, *J* = 8.1 Hz, 1H), 2.75 (s, 3H). ^13^C NMR (151 MHz, DMSO-*d*_6_): δ 175.5, 161.8, 161.6, 132.6, 131.6, 131.3, 123.8, 109.4, 12.9. HRMS (ESI) *m*/*z*: calcd for C_11_H_8_Br_2_NO_3_ [Μ + Η]^+^, 361.8845; found, 361.8892.

### 3-(2,6-Dimethylphenyl)-5-methylisoxazole-4-carboxylic Acid (**71**)

This compound was prepared according to the general procedure described above, starting from derivative **69**, in 77% yield, as a white solid. mp 177–178 °C (MeOH). ^1^H NMR (600 MHz, DMSO-*d*_6_): δ 12.81 (br s, 1H, D_2_O exch.), 7.24 (t, *J* = 7.5 Hz, 1H), 7.11 (d, *J* = 7.5 Hz, 2H), 2.73 (s, 3H), 2.01 (s, 6H). ^13^C NMR (151 MHz, DMSO-*d*_6_): δ 175.6, 162.4, 161.3, 136.5, 128.73, 128.66, 127.0, 109.3, 19.6, 13.1. HRMS (ESI) *m*/*z*: calcd for C_13_H_14_NO_3_ [Μ + Η]^+^, 232.0969; found, 232.0999.

### 3-(2,6-Dibromophenyl)-5-methylisoxazole-4-carbonyl Chloride (**72**) and 3-(2,6-Dimethylphenyl)-5-methylisoxazole-4-carbonyl Chloride (**73**)

These compounds were synthesized according to the general method described for the preparation of derivatives **34**–**36** and were used immediately to the next step, with no further purification.

### (*Z*)-*N*′-((3-(2,6-Dibromophenyl)-5-methylisoxazole-4-carbonyl)oxy)picolinimidamide (**74**)

This compound was prepared according to the general procedure described for the synthesis of the target derivatives **37**–**55**, upon reaction of the acyl chloride **72** with amidoxime **18**. Reaction time: 2 h. The crude product was recrystallized from ethyl acetate, to provide the pure derivative **74** in 97% yield, as a white solid. mp 198–199 °C (EtOAc). ^1^H NMR (400 MHz, DMSO-*d*_6_): δ 8.67–8.63 (m, 1H), 7.95–7.88 (m, 2H), 7.86 (d, *J* = 8.1 Hz, 2H), 7.58–7.53 (m, 1H), 7.44 (t, *J* = 8.1 Hz, 1H), 6.98 (br s, 1H, D_2_O exch.), 5.67 (br s, 1H, D_2_O exch.), 2.89 (s, 3H). ^13^C NMR (100 MHz, DMSO-*d*_6_): δ 176.1, 161.2, 157.9, 154.7, 148.8, 147.6, 137.4, 133.2, 132.0, 131.0, 126.0, 123.7, 121.1, 107.9, 13.5. HRMS (ESI) *m*/*z*: calcd for C_17_H_13_Br_2_N_4_O_3_ [Μ + Η]^+^, 480.9329; found, 480.9389. HPLC analysis (Method B): *t*_R_ = 9.14 min, purity 95.34%.

### (*Z*)-5-Bromo-*N*′-((3-(2,6-dibromophenyl)-5-methylisoxazole-4-carbonyl)oxy)picolinimidamide (**75**)

This compound was prepared according to the general procedure described for the synthesis of the target derivatives **37**–**55**, upon reaction of the acyl chloride **72** with amidoxime **20**. Reaction time: 2 h. The crude product was recrystallized from ethyl acetate, to provide the pure derivative **75** in 91% yield, as a white solid. mp 229–230 °C (EtOAc). ^1^H NMR (400 MHz, DMSO-*d*_6_): δ 8.78 (d, *J* = 1.9 Hz, 1H), 8.17 (dd, *J* = 8.5 Hz, 2.3 Hz, 1H), 7.88–7.82 (m, 3H), 7.43 (t, *J* = 8.1 Hz, 1H), 6.91 (br s, 1H, D_2_O exch.), 5.71 (br s, 1H, D_2_O exch.), 2.88 (s, 3H). ^13^C NMR (100 MHz, DMSO-*d*_6_): δ 176.1, 161.2, 157.8, 154.1, 149.6, 146.5, 140.1, 133.2, 132.0, 130.9, 123.7, 122.7, 122.5, 107.8, 13.5. HRMS (ESI) *m*/*z*: calcd for C_17_H_12_Br_3_N_4_O_3_ [Μ + Η]^+^, 558.8434; found, 558.8474. HPLC analysis (Method B): *t*_R_ = 9.87 min, purity 97.35%.

### (*Z*)-*N*′-((3-(2,6-Dimethylphenyl)-5-methylisoxazole-4-carbonyl)oxy)picolinimidamide (**76**)

This compound was prepared according to the general procedure described for the synthesis of the target derivatives **37**–**55**, upon reaction of the acyl chloride **73** with amidoxime **18**. Reaction time: 2 h. The crude product was recrystallized from ethyl acetate/*n*-pentane, to provide the pure derivative **76** in 90% yield, as a pale gray solid. mp 159–161 °C (EtOAc/*n*-pentane). ^1^H NMR (400 MHz, DMSO-*d*_6_): δ 8.65–8.59 (m, 1H), 7.94–7.83 (m, 2H), 7.58–7.49 (m, 1H), 7.33 (t, *J* = 7.8 Hz, 1H), 7.22 (t, *J* = 7.8 Hz, 2H), 6.76 (br s, 1H, D_2_O exch.), 4.82 (br s, 1H, D_2_O exch.), 2.84 (s, 3H) 2.08 (s, 6H). ^13^C NMR (100 MHz, DMSO-*d*_6_): δ 176.7, 160.5, 158.4, 154.3, 148.8, 147.6, 137.4, 136.6, 129.5, 128.6, 127.5, 125.9, 121.0, 107.9, 19.6, 13.4. HRMS (ESI) *m*/*z*: calcd for C_19_H_19_N_4_O_3_ [Μ + Η]^+^, 351.1452; found, 351.1495. HPLC analysis (Method B): *t*_R_ = 9.14 min, purity 97.20%.

### (*Z*)-5-Bromo-*N*′-((3-(2,6-dimethylphenyl)-5-methylisoxazole-4-carbonyl)oxy)picolinimidamide (**77**)

This compound was prepared according to the general procedure described for the synthesis of the target derivatives **37**–**55**, upon reaction of the acyl chloride **73** with amidoxime **20**. Reaction time: 2 h. The crude product was recrystallized from ethyl acetate, to provide the pure derivative **77** in 93% yield, as a white solid. mp 234–235 °C (EtOAc). ^1^H NMR (400 MHz, DMSO-*d*_6_): δ 8.75 (d, *J* = 1.9 Hz, 1H), 8.15 (dd, *J* = 8.5 Hz, 2.2 Hz, 1H), 7.80 (d, *J* = 8.5 Hz, 1H), 7.32 (t, *J* = 7.6 Hz, 1H), 7.20 (d, *J* = 7.6 Hz, 2H), 6.81 (br s, 1H, D_2_O exch.), 4.83 (br s, 1H, D_2_O exch.), 2.83 (s, 3H), 2.06 (s, 6H). ^13^C NMR (100 MHz, DMSO-*d*_6_): δ 176.8, 160.5, 158.3, 153.8, 149.6, 146.6, 140.1, 136.6, 129.6, 128.6, 127.5, 122.6, 122.4, 107.8, 19.6, 13.4. HRMS (ESI) *m*/*z*: calcd for C_19_H_18_BrN_4_O_3_ [Μ + Η]^+^, 429.0557; found, 429.0606. HPLC analysis (Method B): *t*_R_ = 9.95 min, purity 97.03%.

## Data Availability

The following link is provided to access the starting structures (docking poses) and output frames of the complexes between our revised model of the inactive hA_3_R generated using the multistate AF2 method and ligands K18, **37–39**, **56**, **57**, and **60** from the MD simulations: https://github.com/annachor/inactive_A3R_AF2-carbonyloxycarboximidamides_MDs.

## Supporting Information Available

Molecular formula strings (CSV)
Information on the pharmacological, computational, and chemical characterization of the tested compounds (PDF)

## Abbreviations

ΔΔ*G*_b,TI/MD_: relative binding free energy
A_2A_R: adenosine A_2A_ receptor
A_2B_R: adenosine A_2B_ receptor
AF2: Alphafold 2
ARs: adenosine receptors
cAMP: 3′,5′-cyclic adenosine monophosphate
CHO-K1: Chinese hamster ovary K1
CCK-8: cell counting kit-8
CL_int_: intrinsic clearance
CPA: N^6^-cyclopentyl-adenosine
dppf: 1,1′-ferrocenediyl-bis(diphenylphosphine)
DMA: dimethylacetamide
DMSO: dimethyl sulfoxide
FACS: fluorescence-activated cell sorting
FEP/MD: free energy perturbation coupled with molecular dynamics simulations
Ff14sb: amber14sb force field
Ff19sb: amber19sb force field
GPCRs: G protein-coupled receptors
hA_1_R: human adenosine A_1_ receptor
hA_3_R: human adenosine A_3_ receptor
HEK293T: human embryonic kidney 293T
IB-MECA: 1-deoxy-1-[6-[[(3-iodophenyl)methyl]amino]-9*H*-purin-9-yl]-*N*-methyl-β-d-ribofuranuronamide
MAPK: mitogen-activated protein kinase
MD: molecular dynamics
MM/PBSA: Poisson–Boltzmann surface area continuum solvation
MM/GBSA: generalized Born surface area continuum solvation
MRS1220: (*N*-[9-chloro-2-(2-furanyl)-1,2,4-triazolo[1,5-*c*]quinazolin-5-yl]benzeneacetamide)
PMEMD: particle mesh Ewald molecular dynamics
*P*_app_: permeability assay
NECA: 5′-*N*-ethylcarboxamidoadenosine
NanoBRET: Nanoluciferease-based bioluminescence resonance energy transfer
Nluc: Nanoluciferease
RT: residence time
rmsd: root-mean-square deviation
SAR: structure–activity relationships
SBDD: structure-based drug design
SEM: standard error of the mean
TI/MD: thermodynamic integration coupled with molecular dynamics simulations method
TM: transmembrane
VS: virtual screening
WT: wild type
