# Supplementary material for: Hit-to-Lead Optimization of Heterocyclic Carbonyloxycarboximidamides as Selective Antagonists at Human Adenosine A3 Receptor
Source: J Med Chem. 2024 Jul 29;67(15):13117–46. doi: 10.1021/acs.jmedchem.4c01092 (PMC11320584; doi:10.1021/acs.jmedchem.4c01092)
Supplement: Supplementary file 2 — jm4c01092_si_002.pdf [file jm4c01092_si_002.pdf]

Hit-to-Lead Optimization of Heterocyclic Carbonyloxycarboximidamides as Selective Antagonists at Human Adenosine A_3 Receptor

Xianglin Huang, [‡,x] Anna Chorianopoulou, [†,x] Panagoula Kalkounou,[†,x] Maria Georgiou, [†] Athanasios Pousias, [†] Amy Davies, [‡] Abigail Pearce, [‡] Matthew Harris, [‡] George Lambrinidis,[†] Panagiotis Marakos, [†] Nicole Pouli,[†] Antonios Kolocouris, [†,y,*] Nikolaos Lougiakis, [†,y,*] and Graham Ladds, [‡,y,*]

[‡] Department of Pharmacology, University of Cambridge, Tennis Court Road, Cambridge, CB2 1PD, UK.

[†] Laboratory of Medicinal Chemistry, Section of Pharmaceutical Chemistry, Department of Pharmacy, School of Health Sciences, National and Kapodistrian University of Athens, Panepistimiopolis-Zografou, 15771 Athens, Greece

Keywords: adenosine A_3 receptor, antagonist, BRET, kinetics of binding, molecular dynamics, mutagenesis, residence time, thermodynamic integration

[x] These authors contribute equally
[y] These authors contribute equally

Corresponding authors
Prof Graham Ladds, Department of Pharmacology, University of Cambridge, Tennis Court Road, Cambridge, CB2 1PD Tel; +44 (0) 1223 334020. Email: grl30@cam.ac.uk

Dr Nikolaos Lougiakis, Laboratory of Medicinal Chemistry, Section of Pharmaceutical Chemistry, Department of Pharmacy, National and Kapodistrian University of Athens, Panepistimiopolis Zografou, Athens, 15771. Tel: 210-727-4759, Email: nlougiak@pharm.uoa.gr

Prof Antonios Kolocouris, Laboratory of Medicinal Chemistry, Section of Pharmaceutical Chemistry, Department of Pharmacy, National and Kapodistrian University of Athens, Panepistimiopolis Zografou, Athens, 15771. Tel: 210-727-4834, Email: ankol@pharm.uoa.gr

Table S1. Calculated relative binding free energies ($\Delta\Delta G_{b,TI/MD}$) determined by the TI/MD method [1,2] with ff19sb [3] and a thermodynamic cycle for alchemical transformations of heterocyclic carbonyloxycarboximidamides in complex with inactive hA_3R embedded in phospholipid bilayers, experimental relative binding free energies ($\Delta\Delta G_{b,exp}$) and absolute deviation of calculated binding free energies from experimental binding free energies ($|\Delta\Delta G_{b,TI/MD} - \Delta\Delta G_{b,exp}|$) (free energies in kcal mol^{-1}). For inactive hA_3R we used our revised model [4] generated using the multi-state AF2 method. [5,6]

No	ligA	Chemical structure	pK_i	ligB	Chemical structure	pK_i	$\Delta\Delta G_{b,exp}$[a]	error	$\Delta\Delta G_{b,TI/MD}$	error	$\|\Delta\Delta G_{b,TI/MD} - \Delta\Delta G_{b,exp}\|$
1	**K18**	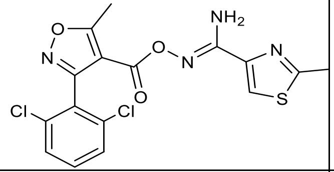	6.92±0.10	**37**	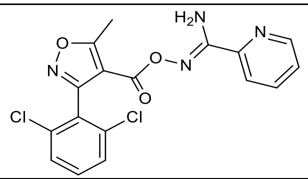	7.33±0.16	**-0.77**	0.08	**-1.51**	0.08	**0.74**
2	**37**	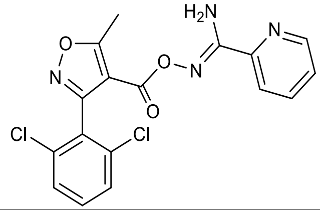	7.33± 0.16	**40**	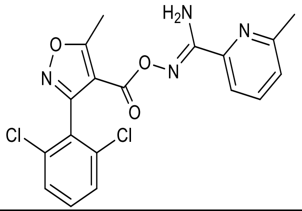	6.89±0.06	**0.81**	0.06	**0.57**	0.04	**0.24**
3	**37**	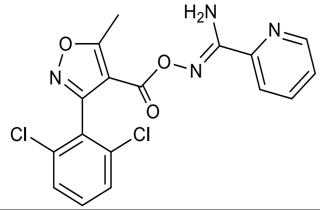	7.33±0.16	**48**	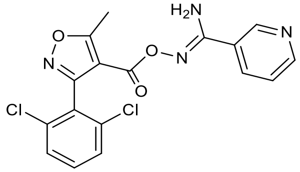	6.99±0.06	**0.67**	0.05	**2.14**	0.08	**1.47**
4	**37**	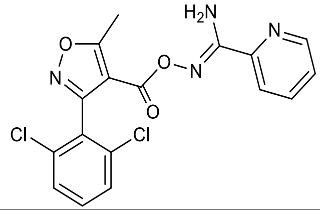	7.33±0.16	**45**	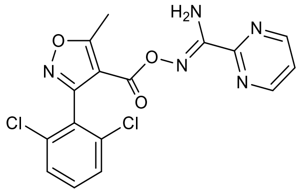	6.27±0.06	**1.69**	0.05	**0.50**	0.04	**1.19**
5	**37**	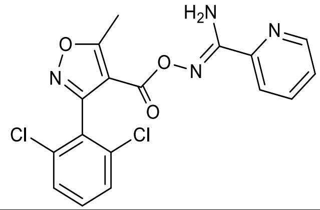	7.33±0.16	**39**	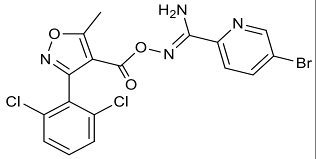	7.92±0.06	**-0.67**	0.07	**-1.01**	0.04	**0.34**

6	**37**	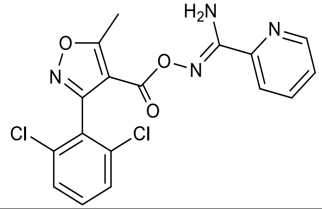	7.33±0.16	**38**	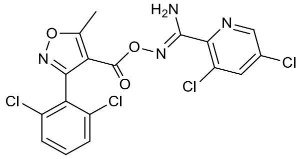	5.97±0.03	**2.11**	0.04	**0.58**	0.04	**1.53**
7	**37**	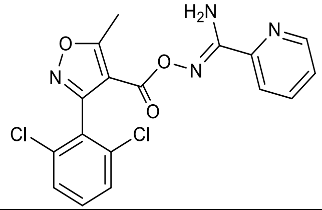	7.33±0.16	**54**	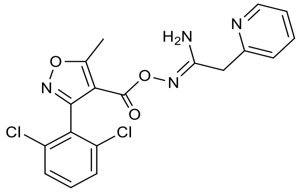	6.50±0.02	**1.36**	0.04	**2.55**	0.06	**1.19**
8	**37**	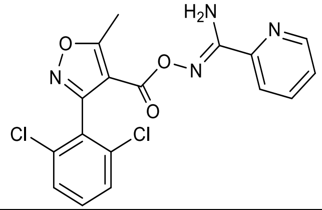	7.33±0.16	**74**	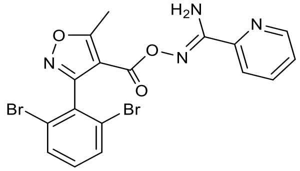	7.56±0.04	**-0.14**	0.05	**-0.53**	0.04	**0.39**
9	**37**	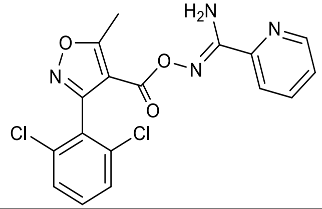	7.33±0.16	**76**	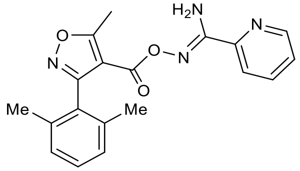	6.90±0.03	**0.79**	0.05	**-0.64**	**0.05**	**1.43**
10	**39**	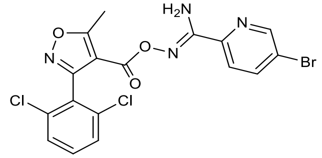	7.92±0.06	**75**	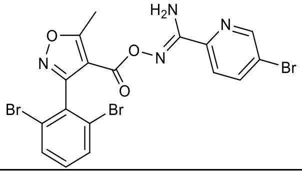	7.53±0.06	**0.57**	0.07	**-0.45**	0.04	**1.02**
11	**39**	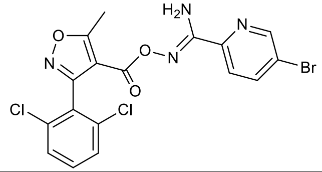	7.92±0.06	**77**	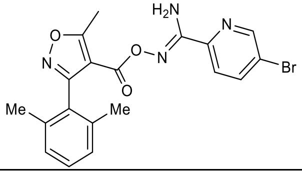	7.36±0.07	**0.81**	0.08	**-0.46**	0.05	**1.27**

12	**37**	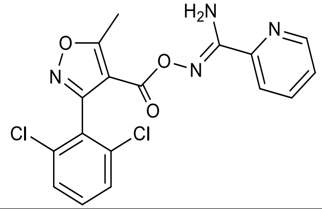	7.46±0.05	**53**	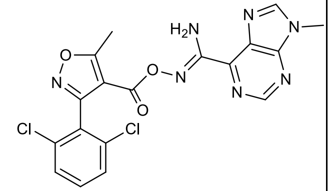	6.37±0.06	**1.55**	0.06	**2.10**	0.11	**0.55**
13	**37**	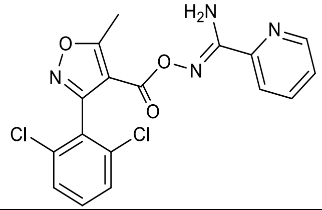	7.33±0.16	**52**	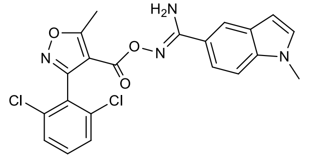	5.33±0.02	**3.02**	0.04	**1.44**	0.12	**1.58**
14	**40**	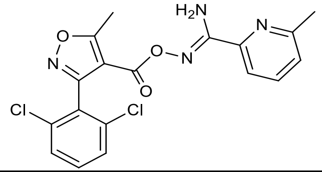	6.89±0.06	**49**	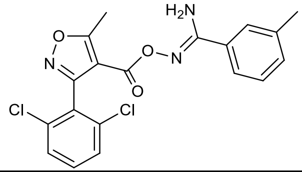	6.68±0.10	**0.30**	0.08	**1.60**	0.09	**1.30**
15	**55**	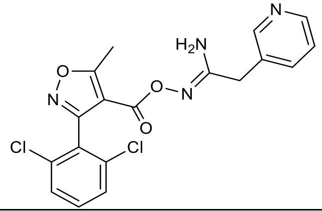	6.05±0.04	**48**	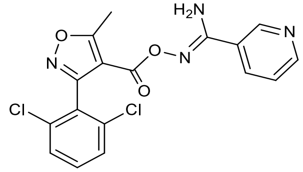	6.99±0.05	**-1.33**	0.05	**-2.64**	0.11	**1.31**
16	**48**	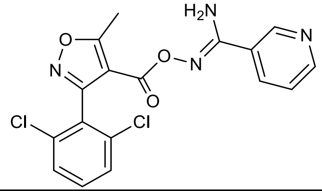	6.99±0.05	**46**	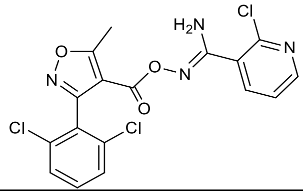	5.68±0.15	**1.86**	0.10	**0.57**	0.05	**1.92**
17	**37**	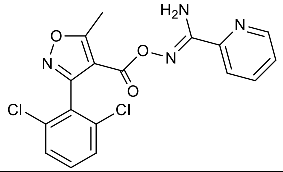	7.33±0.16	**44**	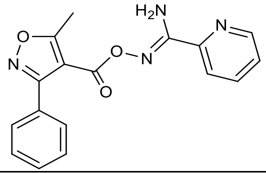	6.17±0.03	**1.83**	0.06	**0.64**	0.04	**1.19**

18	**42**	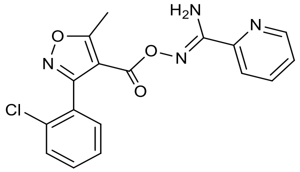	6.70±0.03	**44**	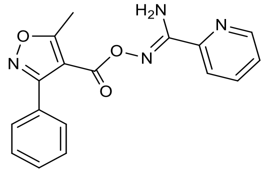	6.17±0.06	**0.75**	0.05	**0.98**	0.04	**0.23**
19	**42**	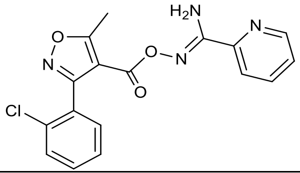	6.70±0.03	**37**	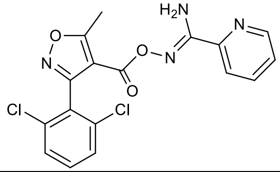	7.33±0.16	**-1.08**	0.04	**-1.33**	0.03	**0.25**
20	**41**	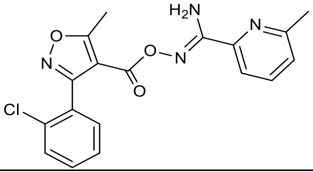	6.18±0.06	**40**	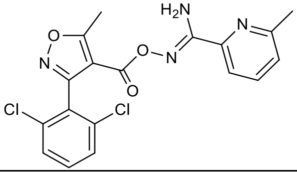	6.89±0.06	**-1.01**	0.06	**-0.47**	0.05	**0.54**
21	**56**	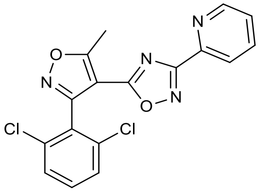	6.72±0.10	**59**	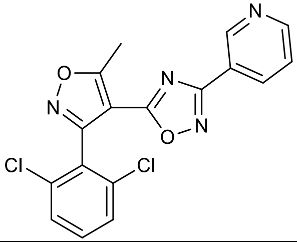	7.04±0.08	**-0.45**	0.08	**0.26**	0.08	**0.71**
22	**56**	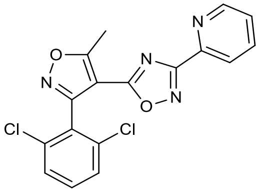	6.72±0.10	**57**	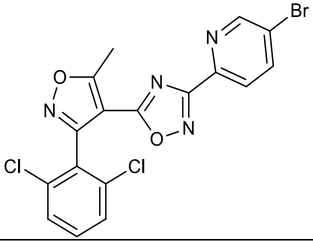	6.32±0.14	**0.57**	0.14	**-0.48**	0.05	**1.05**

23	**59**	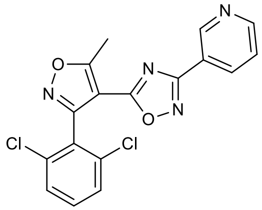	7.04±0.08	**60**	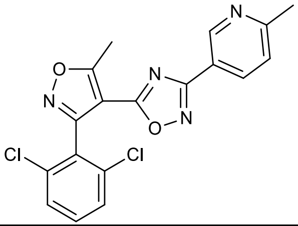	7.23±0.06	**-0.27**	0.07	**0.07**	0.05	**0.34**
										MUE=0.89

[a]Experimental relative binding free energies ($\Delta\Delta G_{b,exp}$) are estimated using experimental binding affinities (pK_i) according to eq. (11) in Methods Section.

Table S2. Chemical structures of the amidoximes **18-30**, and their corresponding precursor aryl or aralkyl nitriles **1-11**, **13** and **17**. Method of preparation of amidoximes **18-30**, yield of each reaction and solvent used for their recrystallization.

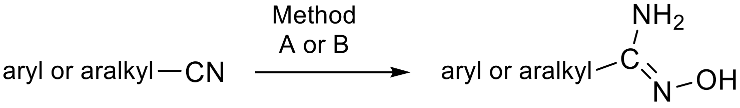

1-11, 13, 17 **18 - 30**

Nitrile	Amidoxime	Method of preparation	Yield	Recrystallization solvent
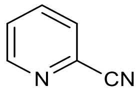 **1**	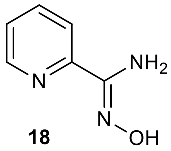 **18**	A	82%	EtOH
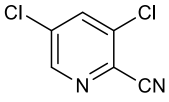 **2**	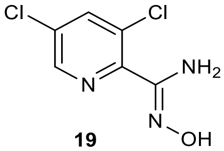 **19**	B	88%	EtOH
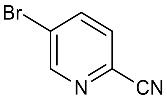 **3**	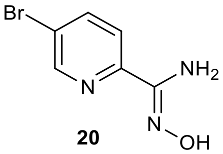 **20**	A	76%	EtOH
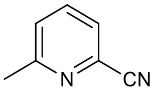 **4**	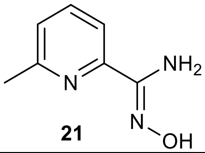 **21**	A	80%	EtOH
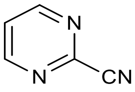 **5**	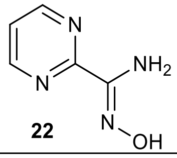 **22**	A	93%	EtOH
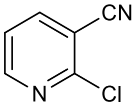 **6**	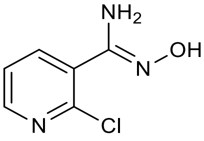 **23**	A	79%	EtOH
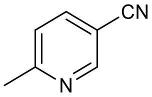 **7**	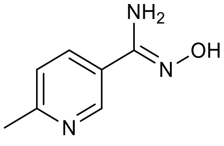 **24**	A	86%	EtOH

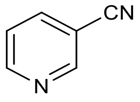 **8**	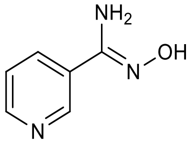 **25**	B	85%	MeOH/*n*-pentane
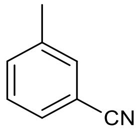 **9**	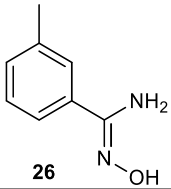 **26**	A	96%	EtOAc/*n*-pentane
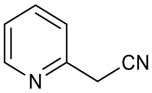 **10**	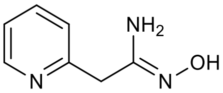 **27**	B	71%	EtOH
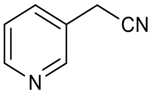 **11**	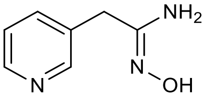 **28**	A	78%	EtOH
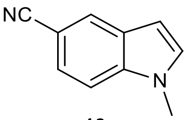 **13**	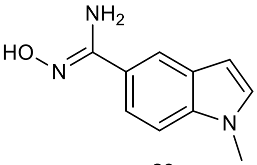 **29**	A	72%	EtOH
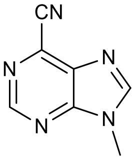 **17**	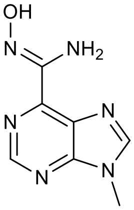 **30**	B	67%	CH_2Cl_2/*n*-pentane

Method A: $HONH_2$ (50% wt solution in water), EtOH, reflux, 2 h
Method B: $HONH_2 \cdot HCl$, $NaHCO_3$, EtOH, reflux, 2 h

Table S3. Chemical structure and the binding affinity (pK_i [a]) determined in NanoBRET ligand binding assay of the 18 compounds that have lower affinity than **K18**.

Group A

Compound	Structure	pK_i ± SEM	n
**38**	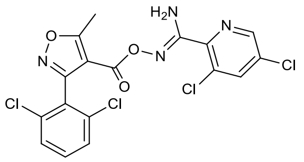	5.97 ± 0.03* (*p* < *0.0001*)	3
**45**	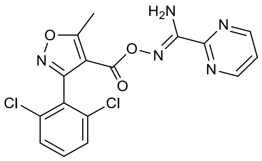	6.27 ± 0.06* (*p* < *0.0001*)	3
**46**	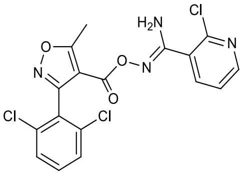	5.68 ± 0.15* (*p* < *0.0001*)	3
**49**	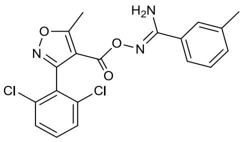	6.68 ± 0.10	4
**52**	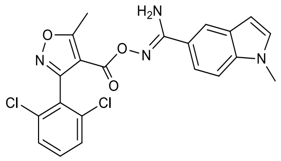	5.33 ± 0.02* (*p* < *0.0001*)	3
**53**	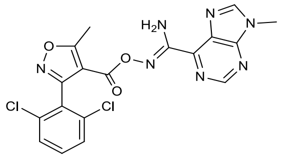	6.37 ± 0.06* (*p* = *0.0010*)	3
**54**	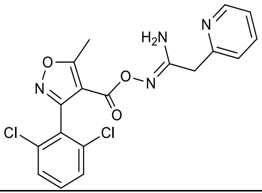	6.50 ± 0.02* (*p* = 0.0124)	4
**55**	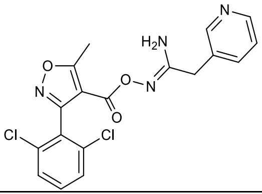	6.05 ± 0.04* (*p* < *0.0001*)	4

Group B

Compound	Structure	pK_i ± SEM	n
**41**	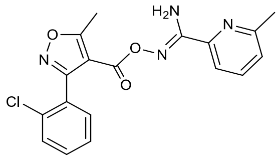	6.18 ± 0.06* ($p < 0.0001$)	4
**42**	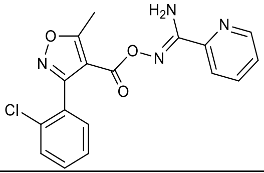	6.70 ± 0.03	3
**51**	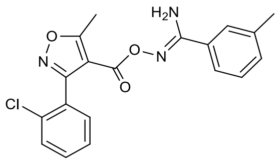	6.26 ± 0.03* ($p < 0.0001$)	3

Group C

Compound	Structure	pK_i ± SEM	n
**43**	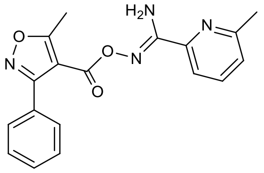	6.15 ± 0.12* ($p< 0.0001$)	4
**44**	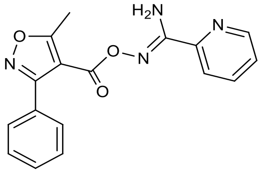	6.17 ± 0.06* ($p < 0.0001$)	3
**50**	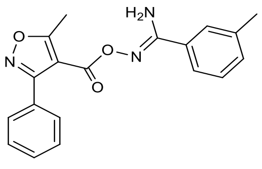	6.17 ± 0.14* ($p < 0.0001$)	3

Group D

Compound	Structure	pK_i ± SEM	n
**56**	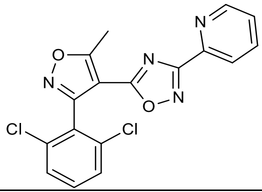	6.72 ± 0.10	5
**57**	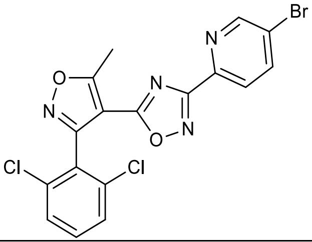	6.32 ± 0.14* (*p = 0.0002*)	3
**58**	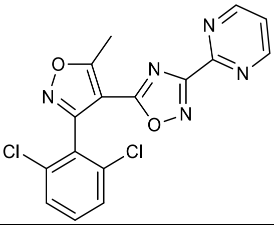	6.21 ± 0.07* (*p = 0.0008*)	3
**61**	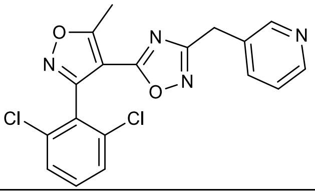	5.91 ± 0.05* (*p < 0.0001*)	3

[a] All the equilibrium binding affinities (pK_i) were determined with NanoBRET binding assay and represented as mean ± standard error of the mean (SEM) of at least 3 independent repeats with experiment conducted in duplicates. One-way ANOVA with Dunnett's post-test was used to determine the statistical significance (*$p < 0.05$) compared to the pK_i of **K18**.

Table S4. Potency of **NECA** as stimulated cAMP inhibition (in hA_1R/hA_3R) or accumulation (in $A_{2A}R/A_{2B}R$) in the presence of selected compounds.[a]

	pEC_{50}			
	**hA_1R**	**$hA_{2A}R$**	**$hA_{2B}R$**	**hA_3R**
**DMSO**	9.745 ± 0.31	7.822 ± 0.08	6.525 ± 0.16	7.802 ± 0.21
**37**	9.593 ± 0.17	6.848 ± 0.39	6.102 ± 0.26	5.309 ± 0.22* ($p < 0.0001$)
**39**	9.699 ± 0.24	7.768 ± 0.09	6.110 ± 0.21	5.672 ± 0.48* ($p = 0.0003$)
**40**	9.186 ± 0.07	7.563 ± 0.42	6.544 ± 0.11	5.748 ± 0.14* ($p < 0.0001$)
**47**	9.252 ± 0.07	7.992 ± 0.21	6.105 ± 0.26	5.806 ± 0.33* ($p = 0.0001$)
**48**	9.487 ± 0.10	7.692 ± 0.25	6.423 ± 0.12	5.706 ± 0.24* ($p < 0.0001$)
**59**	8.803 ± 0.08* ($p = 0.0159$)	6.960 ± 0.16* ($p = 0.0200$)	5.523 ± 0.19* ($p = 0.0136$)	5.840 ± 0.17* ($p = 0.0002$)
**60**	9.257 ± 0.29	6.610 ± 0.25* ($p = 0.0015$)	5.578 ± 0.15* ($p = 0.0228$)	5.904 ± 0.15* ($p = 0.0002$)

[a] CHO-K1 cells stably expressing $hA_1R/hA_{2A}R/hA_{2B}R/hA_3R$ were stimulated with 1 µM **forskolin** (in hA_1R/hA_3R) or DMSO (in $hA_{2A}R/hA_{2B}R$) as well as 10 µM tested compound and increasing concentrations of **NECA**. Data are expressed as mean ± SEM obtained in n = 3 independent experimental repeats, conduct in duplicates. One-way ANOVA with Dunnett's post-test was used to determine the statistical significance (*$p < 0.05$) compared to 'DMSO'.

Table S5. Potency of **NECA**-stimulated cAMP inhibition (in A_1R/A_3R) or accumulation (in $A_{2A}R/A_{2B}R$) in the presence of the compounds **74-77**. [a]

	pEC$_{50}$			
	**A_1R**	**$A_{2A}R$**	**$A_{2B}R$**	**A_3R**
DMSO	9.543 ± 0.12	8.000 ± 0.09	6.792 ± 0.15	8.268 ± 0.023
**74**	9.235 ± 0.14	7.603 ± 0.220	6.282 ± 0.227	7.710 ± 0.184* ($p < 0.0001$)
**75**	9.284 ± 0.08	7.724 ± 0.244	6.364 ± 0.276	5.721 ± 0.120* ($p < 0.0001$)
**76**	9.552 ± 0.07	7.863 ± 0.029	6.690 ± 0.264	5.918 ± 0.141* ($p < 0.0001$)
**77**	9.502 ± 0.15	7.732 ± 0.081	6.677 ± 0.204	6.645 ± 0.202* ($p < 0.0001$)

[a] CHO-K1 cells stably expressing $A_1R/A_{2A}R/A_{2B}R/A_3R$ were stimulated with 1 µM **forskolin** (in A_1R/A_3R) or DMSO (in $A_{2A}R/A_{2B}R$) as well as 10 µM tested compound and increasing concentrations of **NECA**. Data are expressed as mean ± SEM obtained in n = 3 independent experimental repeats, conduct in duplicates. One-way ANOVA with Dunnett's post-test was used to determine the statistical significance (*$p < 0.05$) compared to '**NECA** only'.

Table S6. The binding affinities (K_i) of **CA200645** and the changes in affinity(ΔpK_i) of compounds **37**, **39**, **CA200645** and **NECA** at WT and mutant Nluc-hA_3R.

Mutation	Residue's region in the binding area	Cell-surface expression[a]	K_i of CA200645[b]			
				**37**	**39**	**NECA**
WT		100 ± <0.1	23.8 ± 2.2	-	-	-
Y15$^{1.35}$A	Top	26 ± 2* (p = 0.0291)	61.6 ± 5.8* (p = 0.0063)	-0.56 ± 0.20* (p = 0.0019)	-0.94 ± 0.23* (p < 0.0001)	-1.25 ± 0.36* (p < 0.0001)
V65$^{2.57}$A	Top	48 ± 2	36.3 ± 2.1	1.31 ± 0.33* (p<0.0001)	1.11 ± 0.29* (p < 0.0001)	-0.28 ± 0.15
A69$^{2.61}$G	Top	85± 7	27.7 ± 4.2	-0.43 ± 0.13	-0.57 ± 0.10* (p = 0.0013)	-0.83 ± 0.07* (p = 0.0190)
V72$^{2.64}$A	Top	134 ± 22	48.6 ± 3.3	-0.03 ± 0.23	-0.02 ± 0.08	-0.99 ± 0.24* (p = 0.0022)
T87$^{3.29}$A	Bottom	46 ± 11	42.1 ± 3.0	-0.11 ± 0.11	-0.14 ± 0.07	-0.48 ± 0.27
L90$^{3.32}$A	Bottom	43 ± 2	23.3 ± 1.2	0.96 ± 0.13* (p<0.0001)	0.89 ± 0.13* (p < 0.0001)	-0.46 ± 0.22
L91$^{3.33}$A	Bottom	96 ± 10	153.1 ± 6.7* (p < 0.0001)	-1.72 ± 0.14* (p<0.0001)	-1.95 ± 0.13* (p < 0.0001)	-1.61 ± 0.17* (p < 0.0001)
T94$^{3.36}$A	Bottom	40 ± 3	27.8 ± 2.0	0.07 ± 0.19	-0.42 ± 0.28	-0.84 ± 0.16* (p = 0.0065)
F168$^{5.29}$A	Middle	133 ± 7	n.b.	N.D.	N.D.	N.D.
V169$^{5.30}$A	Top	84 ± 3	32.6 ± 2.0	-0.20 ± 0.04	0.13 ± 0.01	-0.10 ± 0.09
M172$^{5.33}$A	Top	95 ± 6	252.3 ± 6.5* (p < 0.0001)	-0.72 ± 0.07* (p=0.0029)	-0.99 ± 0.07* (p < 0.0001)	-0.74 ± 0.11
M177$^{5.38}$A	Middle	98 ± 8	129.9 ± 11.2* (p < 0.0001)	-0.91 ± 0.07* (p<0.0001)	-1.36 ± 0.05* (p < 0.0001)	-1.63 ± 0.03* (p < 0.0001)
V178$^{5.39}$A	Middle	117 ± 4	60.1 ± 7.9* (p = 0.0096)	-1.58 ± 0.05 * (p<0.0001)	-1.25 ± 0.16* (p < 0.0001)	-1.29 ± 0.20* (p < 0.0001)
F182$^{5.43}$A	Bottom	133 ± 9	41.6 ± 6.8	-0.65 ± 0.16* (p=0.0099)	-0.57 ± 0.10* (p = 0.0015)	-0.18 ± 0.20
I186$^{5.47}$A	Bottom	81 ± 4	34.3 ± 3.8	-0.39 ± 0.20	-0.50 ± 0.14* (p = 0.0054)	-1.74 ± 0.23* (p < 0.0001)
W243$^{6.48}$A	Bottom	19 ± 1* (p = 0.0063)	15.1 ± 0.8	-0.42 ± 0.23	-0.61 ± 0.04* (p = 0.006)	-0.18 ± 0.29
L246$^{6.51}$A	Middle	32 ± 4	258.4 ± 18.4* (p < 0.0001)	-1.26 ± 0.20* (p < 0.0001)	-2.04 ± 0.10* (p < 0.0001)	-2.10 ± 0.10* (p < 0.0001)
I249$^{6.54}$A	Middle	62 ± 3	18.4 ± 5.3	-0.01 ± 0.14	-0.26 ± 0.24	0.74 ± 0.14* (p = 0.0379)
N250$^{6.55}$A	Middle	82 ± 8	n.b.	N.D.	N.D.	N.D.
I253$^{6.58}$A	Top	51 ± 4	17.0 ± 0.7	-0.72 ± 0.10* (p = 0.0030)	-0.15 ± 0.14	-0.47 ± 0.11
L264$^{7.35}$A	Middle	42 ± 3	34.7 ± 11.3	-0.21 ± 0.19	0.33 ± 0.31	0.07 ± 0.17
Y265$^{7.36}$A	Middle	63 ± 1	33.2 ± 1.9	-0.21 ± 0.13	-0.67 ± 0.10* (p = 0.0038)	-0.82 ± 0.11* (p = 0.0048)
I268$^{7.39}$A	Bottom	28 ± 2 (p = 0.0417)	121.9 ± 12.0* (p < 0.0001)	-1.11 ± 0.13* (p < 0.0001)	-1.14 ± 0.07* (p < 0.0001)	-1.78 ± 0.10* (p < 0.0001)
H272$^{7.43}$A	Bottom	22 ± 1 (p = 0.0093)	n.b.	N.D.	N.D.	N.D.

[a] Cell surface expression level of FLAG-Nluc-hA_3R WT and mutants measures as median fluorescence intensity of PE (% of WT response) determined in flow cytometry. [b] The equilibrium dissociation constant (K_D) of **CA200645** in nM determined in NanoBRET saturation binding assay. [c] The change of compound affinity when compared with WT (ΔpK_i) determined in NanoBRET competition assay. Data is represented as mean±SEM of n=3 independent repeats conducted in duplicates. n.b. indicates no binding of **CA200645** and the pK_i of compounds at the corresponding mutants are therefore not determined (N.D.). Statistical significance (*$p < 0.05$) compared with WT was determined using Kruskal-Wallis test for the normalised cell surface expression level or one-way ANOVA with Dunnett's post-test for the pK_i.

Table S7 Potency of **CPA**- or **IB-MECA**-stimulated inhibition of forskolin-mediated cAMP accumulation NCI-H1792 cells in the absence or presence of 10μM **37** or **39**, with associated pK_B values. [a]

	**IB-MECA**		**CPA**	
	**pEC_{50}**	**pK_B**	**pEC_{50}**	**pK_B**
**DMSO**	8.50 ± 0.32	-	6.51 ± 0.63	-
**37**	6.69 ± 0.57 *	7.17 ± 0.13	6.83 ± 0.52	N.D.
**39**	6.24 ± 0.42 *	7.46 ± 0.20	7.13 ± 0.60	N.D.

[a] NCI-H1792 cells were treated with 1μM **forskolin** and 10μM **37** or **39** (or DMSO), and stimulated with **IB-MECA** or **CPA**. Data sets are expressed as mean ± SEM of n individual experiments, where n = 5 (**IB-MECA**) or 4 (**CPA**). Significance was determined using a One-Way ANOVA with Dunnett's post-hoc test for multiple comparisons. * $p < 0.05$, compared to DMSO treated cells.

Table S8 Potency and Emax values for the inhibition of LK-2 and NCI-H1792 cell proliferation mediated by **37** and **39**. [a]

	**37**		**39**	
	**pEC_{50}**	**E_{max}**	**pEC_{50}**	**E_{max}**
**LK-2**	4.64 ± 0.13	99.09 ± 0.92	4.85 ± 0.12	40.03 ± 9.85
**NCI-H1792**	4.56 ± 0.22	100.00 ± 0.00	5.44 ± 0.12 *	61.51 ± 3.32 *

[a] LK-2 or NCI-H1792 cells were treated with different concentrations of **37** or **39** (or DMSO as a vehicle control) and cell count measured after 72 hours. E_{max} values are expressed as inhibition of cell proliferation. Values are expressed as mean ± SEM of n individual experiments, where n = 3 (LK-2) or 4 (NCI-H1792). Significance between cell lines was determined using an unpaired Student's t-test. * $p < 0.05$.

Table S9. HPLC analysis methods for purity determination
HPLC method A:

Mobile phase: 0.2% Formic acid (FA) in Water (H_2O)/ Acetonitrile (ACN).

Flowrate: 0.8 mL/min; column temperature: 25 °C; injection volume: 5 μL.

Eluent:

TIME (min)	H_2O+0.2%FA %	ACN%	FLOW (mL/min)
0	80	20	0.8
6	50	50	0.8
8	0	100	0.8
14	70	30	0.8
20	70	30	0.8

HPLC method B:

Mobile phase: 1% Formic acid (FA) in Water (H_2O)/ Acetonitrile (ACN): Methanol (MeOH) 9:1.

Flowrate: 1 mL/min; column temperature: 25 °C; injection volume: 5 μL.

Eluent:

TIME (min)	H_2O+1%FA %	ACN/MeOH(9:1)%	FLOW (mL/min)
0	80	20	1
3	60	40	1
8	0	100	1
15	70	30	1
20	70	30	1

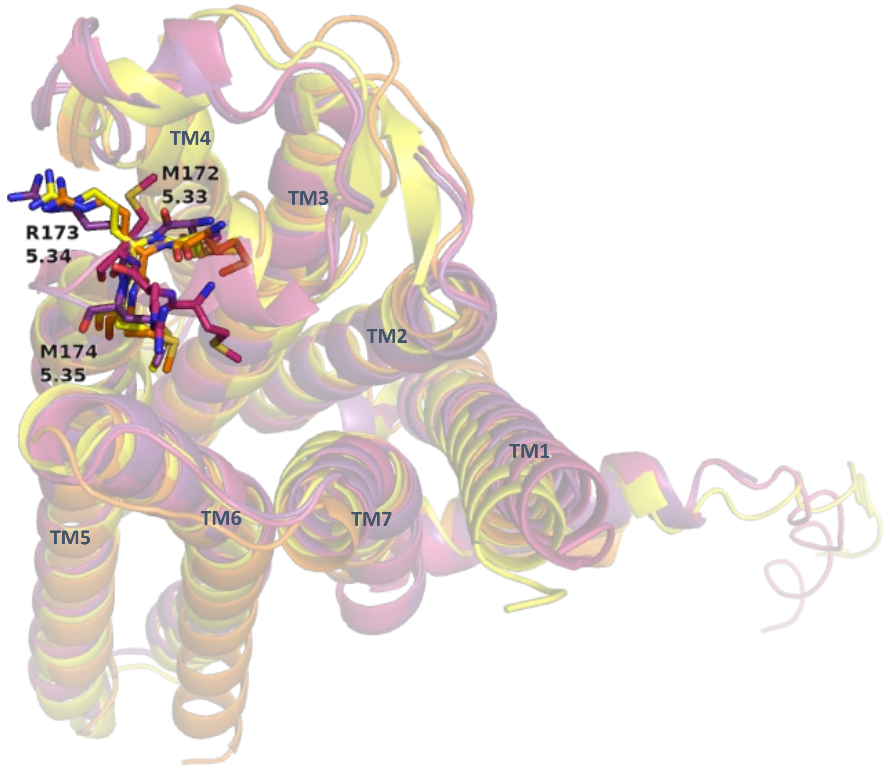

Figure S1. Superposition of M172$^{5.33}$, R173$^{5.34}$ M174$^{5.34}$ motif (shown in sticks) between the experimental structures of the active hA_3R (PDB IDs 8X16 [7]) and in our revised [8] multi AF2-based model for the inactive hA_3R (**Figure S1**), that we used for all the MD simulations in the present work; with orange ribbons for the TMs and orange carbons for the M172$^{5.33}$, R173$^{5.34}$ M174$^{5.34}$ motif is depicted the cryo-EM structure (PDB IDs 8X16 [7]); with yellow ribbons for the TMs and yellow carbons for the M172$^{5.33}$, R173$^{5.34}$ M174$^{5.34}$ motif is depicted the multi AF2-based model for the active hA_3R; with magenta ribbons for the TMs and magenta orange carbons for the M172$^{5.33}$, R173$^{5.34}$ M174$^{5.34}$ motif is depicted our revised [8] multi AF2-based model for the inactive hA_3R; with red ribbons for the TMs and red carbons for the M172$^{5.33}$, R173$^{5.34}$ M174$^{5.34}$ motif is depicted the multi AF2-based model for the inactive hA_3R.

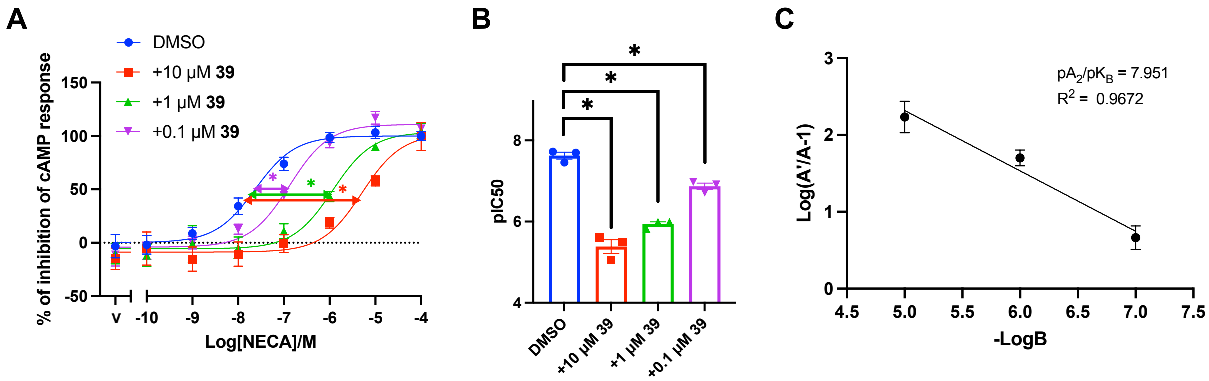

Figure S2. Schild analysis of compound 39 at hA_3R.(A) Dose-response curves of **NECA**-induced cAMP inhibition was measured using 10 M, 1 μM and 0.1 μM **39**, normalized against the response induced by 100 μM **forskolin**. (B)pIC_{50} values for individual repeats. In both (A) and (B), One-way ANOVA analysis was performed to compare the changes in potency between **DMSO** only and the presence of different concentrations of **39** (*, $p<0.05$) (C) Using the pEC_{50} values (**Table S4**), Schild regression analysis was conducted to calculate pA_2/pK_B values of **39**.

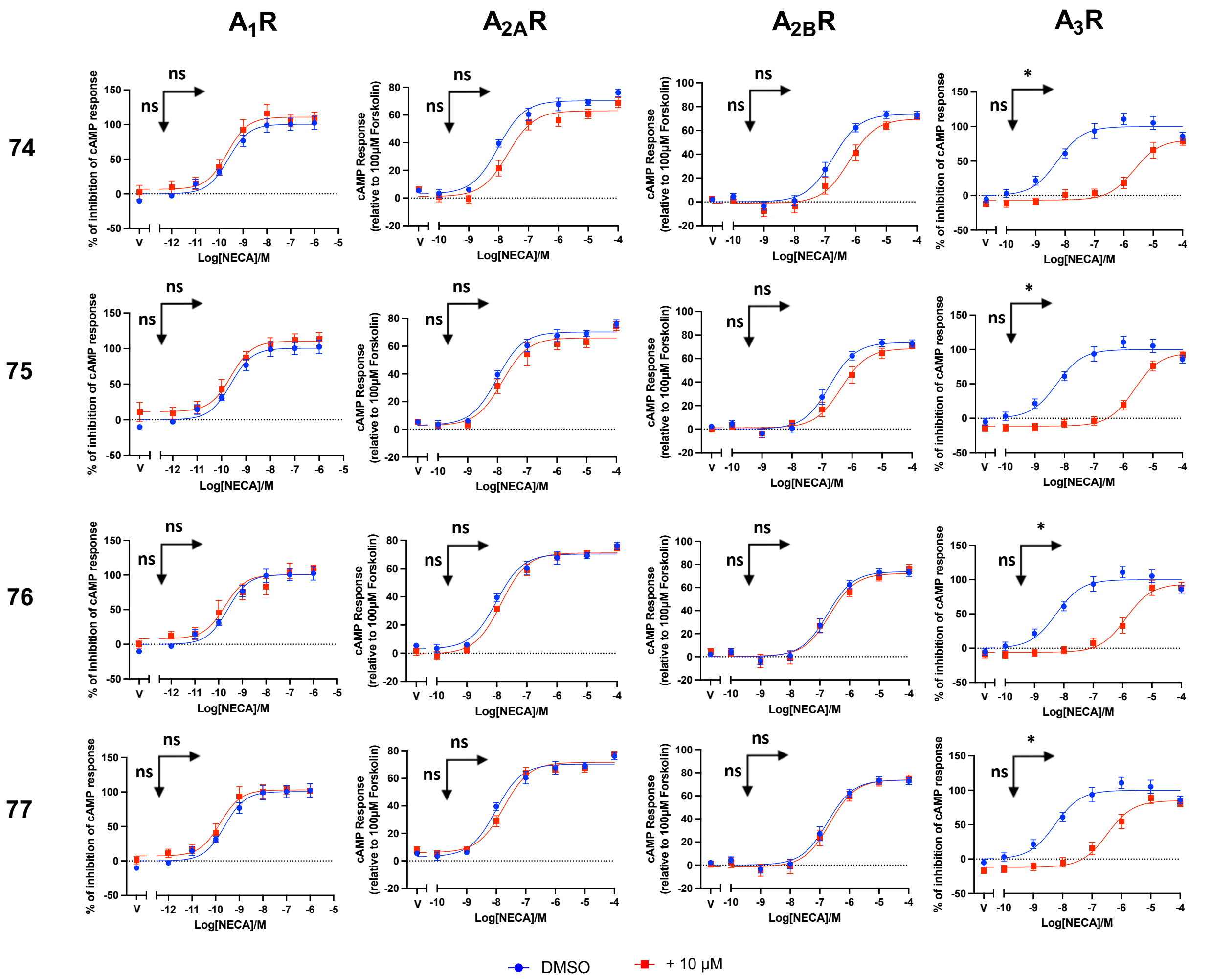

Figure S3. Characterization of the four new compounds at all human AR subtypes in cAMP accumulation assay. CHO-K1 cells stably expressing individual AR subtypes were treated with different concentrations of **NECA** or vehicle (V) and 1 μM **forskolin** in the case of $G_{i/o}$-coupled hA_1R and hA_3R or DMSO control in the case of G_s-coupled $hA_{2A}R$ and $hA_{2B}R$, as well as 10 μM test compound (red) or DMSO control (blue) for 30 minutes. In $hA_{2A}R$ and $hA_{2B}R$, cAMP response was normalized against the response induced by 100 μM **forskolin**, whereas in hA_1R and hA_3R, responses were represented as the percentage of the inhibition of cAMP response generated by 100μM forskolin. Vertical arrow and horizontal arrow denote the significance of the change in efficacy and potency respectively. One-way ANOVA was performed to compare the changes between DMSO only and the presence of tested compound (*, $p < 0.05$). All values are represented as mean ± SEM, obtained in n = 3 independent experimental repeats, conduct in duplicates.

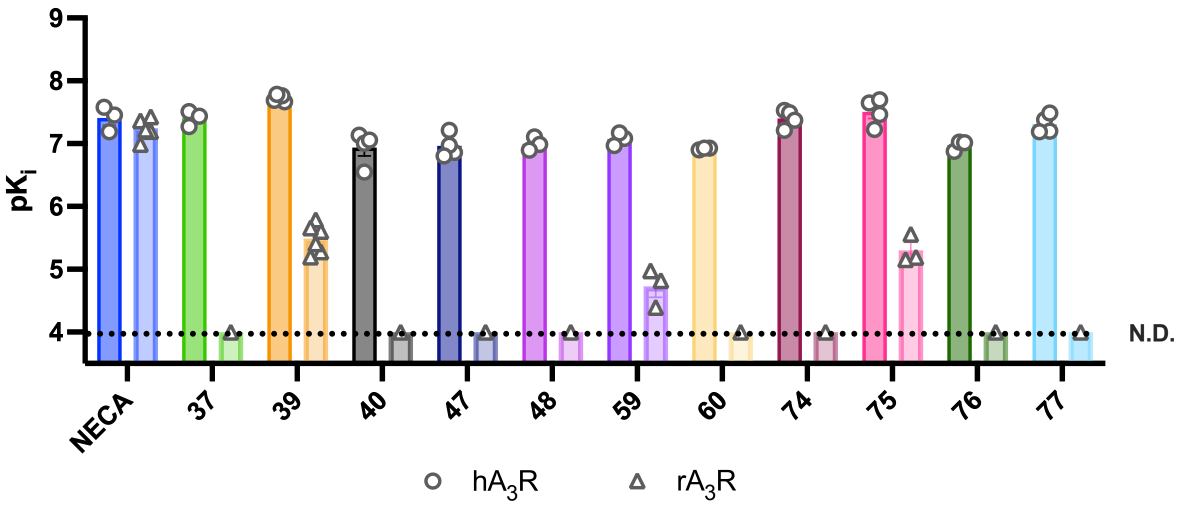

Figure S4. Establishing species selectivity at rA_3R for hA_3R lead compounds. The affinity of the eleven compounds **37, 39, 40, 47, 48, 59, 60, 74, 75, 76 and 77** at rA_3R was determined using the NanoBRET ligand binding assay in HEK293 cells stably expressing Nluc-rA_3R. 70 nM (Nluc-hA_3R) or 100 nM (Nluc-rA_3R) **CA200623** (a fluorescent derivative of **NECA**) was added to HEK293 cells stably expressing either Nluc-hA_3R or Nluc-rA_3R. Note **CA200623** was used since **CA200645** does not bind to the rA_3R. The BRET ratio values were baseline-corrected with the response induced by high concentration (1 μM) A_3R antagonist. The baseline-corrected BRET ratio values at 10 min poststimulation were used to plot the binding curves. The curves were fitted with Cheng-Prusoff equation built into GraphPad Prism 9.3 to determine the binding affinity (p*K*i) of the compounds. Each data point represents the mean ± SEM of at least three experiments performed in duplicates. N.D. presented the non-determined pK_i because the compounds do not fully displace **CA200623**.

Supplementary results on simulations

MD simulations

A Compound **38**

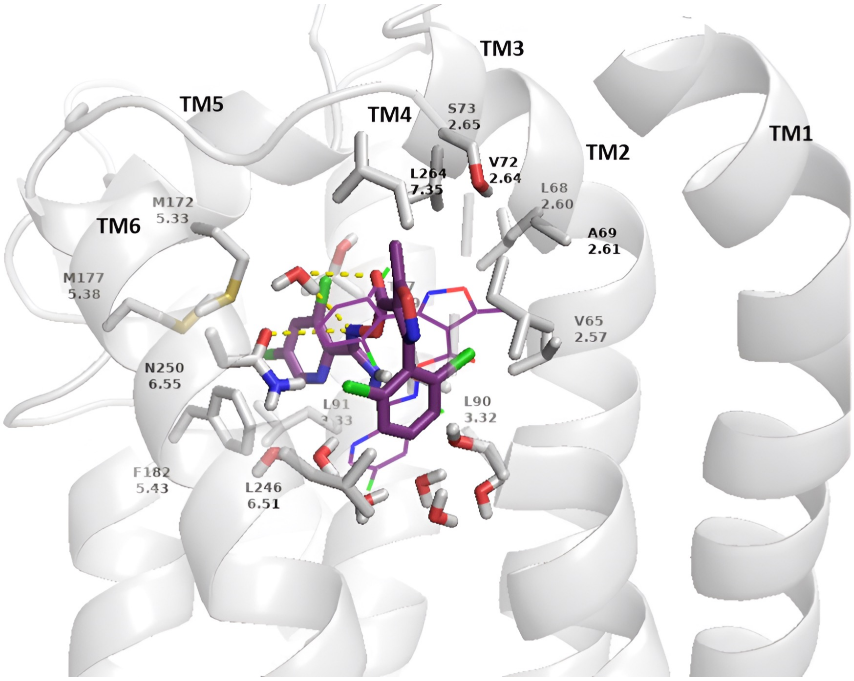

B

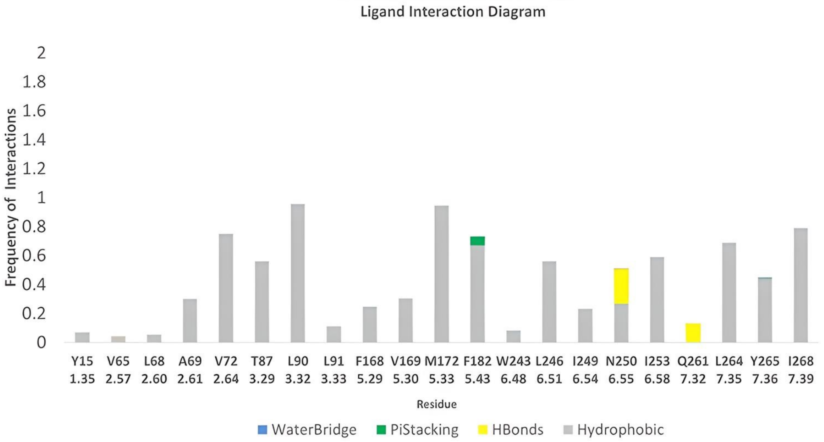

Figure S5. Continued

C

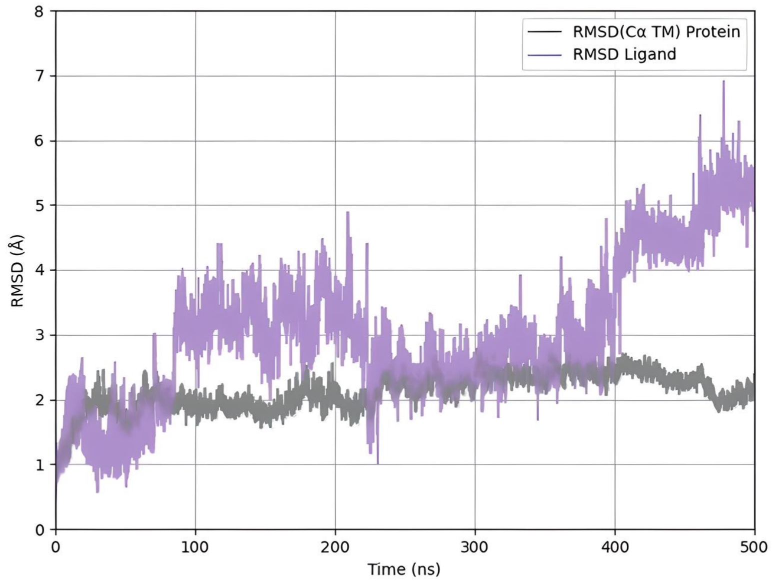

Figure S5. 500ns-MD simulations for the complex of compound **38** and the WT hA_3R using the amber ff19sb.[9](A) Representative frame of **38** inside the orthosteric binding area, (B)receptor-ligand interaction frequency histograms (C)RMSD plots. In (B), bars are plotted only for residues with interaction frequencies ≥ 0.2. Color figure in frames or bar plots: ligand is shown with pink sticks and ligand's starting position with an orange wire, receptor is shown with a white cartoon and sticks, hydrogen bonding interactions are shown with yellow dashes or bars, π-π interactions are shown with green dashes or bars; hydrophobic interactions are shown with grey bars; water bridges are shown with blue bars. In (C) the RMSD plots of Ca carbons of the protein (blue line) and of heavy atoms of the ligand (orange line) are shown. For MD simulations we used a revised model of the inactive form of hA_3R we have recently published, [8] generated using the multi-state AF2 method [5,6] of hA_3R generated from GPCRdb [10] web-tool; the complexes of the starting structure (docking pose) and final snapshot from the MD simulations are available as pdb files (see Ancillary Information).

Oxadiazolyl derivatives

We further expanded this analysis to include compounds **56**, **57, 60** from the subset consisting of compounds **56-61**. In compounds **56-61** we included the 1,2,4-oxadiazole ring between oxazolyl and pyridinyl groups instead of the acyloxyimidamide moiety aiming at lowering the entropic penalty by reducing the ligand's flexibility while maintaining hydrogen bond acceptor atoms for the interaction with N250$^{6.55}$. This rigidification produced an obtuse angle between the (oxadiazolyl) C5-C4 (oxazolyl) and (oxadiazolyl) C3-C1 (pyridinyl) bond vectors which in combination with 5-methyl oxazolyl substitution restricts the hydrogen bonding ability of 4-oxazolyl nitrogen. The 500ns-MD simulations showed that this rigidification caused the rotation of the isoxazole-oxadiazole rotor by 180° degrees re-orientating the dichlorophenyl group towards TM2. As is shown in **Figure S6,A-C** and **Figure S6,D-F** which describe the binding interactions of compounds **56** and **57** the 4-oxadiazolyl nitrogen can form a hydrogen bond with amide side chain of N250$^{6.55}$. Comparison with acyloxyimidamide derivatives **37** and **39** (**Figure 4**), and the corresponding oxadiazole analogs **56** and **57** the van der Waals interactions with V169$^{5.30}$, M172$^{5.33}$, M177$^{5.38}$ can't be formed since the dichlorophenyl group in the later compounds adopts an opposite orientation favoring hydrophobic contacts with more TM2 residues, i.e., V61$^{2.53}$, L68$^{2.60}$, A69$^{2.61}$, V72$^{2.64}$. The combination of 3-pyridinyl group and 4-methyl increased the affinity in **60** (Ki = 58.9 nM, RT = 10.7 min) providing an additional lead for further improvement; the 4-methyl is oriented towards M243$^{6.48}$ favoring hydrophobic interactions.

Among compounds **56-61,** the 3-pyridinyl derivative **59** is a stronger binder by 2-fold compared to 2-pyridinyl derivative **56**, while the opposite is true for the acyloxyimidamide derivatives with **37** having a 2-fold stronger affinity compared to **48**. The methyl substituent at 4-position of the 3-pyridinyl group in **60** (*K*i = 58.9 nM) increases the affinity by 1.5-fold compared to **59** (*K*i = 91.2 nM). A comparison of the MD dynamics simulations between compounds **56** and **60** is described. Starting from a similar docking pose with the pyridinyl nitrogen close to the hydrogen bonding donor amide side chain of N250$^{6.55}$, the 500 ns-MD simulations showed that while 2-pyridinyl group in **56** didn't change considerably its position (**Figure S6,A**) in the complex of compound **60** the 3-pyridinyl group rotates (around the rotor between the oxadiazolyl and pyridinyl groups) and the nitrogen moves away from unfavorable repulsions with isopropyl side chain of L246$^{6.51}$ and faces the center of the binding area (**Figure S6,G**. This allows stronger hydrogen bonding interactions between 4-oxadiazolyl nitrogen and amide side chain of N250$^{6.55}$ and between oxazolyl nitrogen and amide side chain of Q261$^{7.32}$ (**Figure S6,E,F**). The 4-methyl substituent in 3-pyridinyl group is oriented at the bottom of the binding site in the hydrophobic area formed by L91$^{3.32}$, W243$^{6.48}$, I186$^{5.47}$ (**Figure S6,E,F**). Further derivative **57** (*K*i = 479 nM) has ~ 50-fold smaller affinity than it chemical precursor **39** since the bromo substituent in **57** causes a reduction in its hydrogen bonding ability compared to **39** (see **Figure 5D,E** and **Figure S6,D,E**).

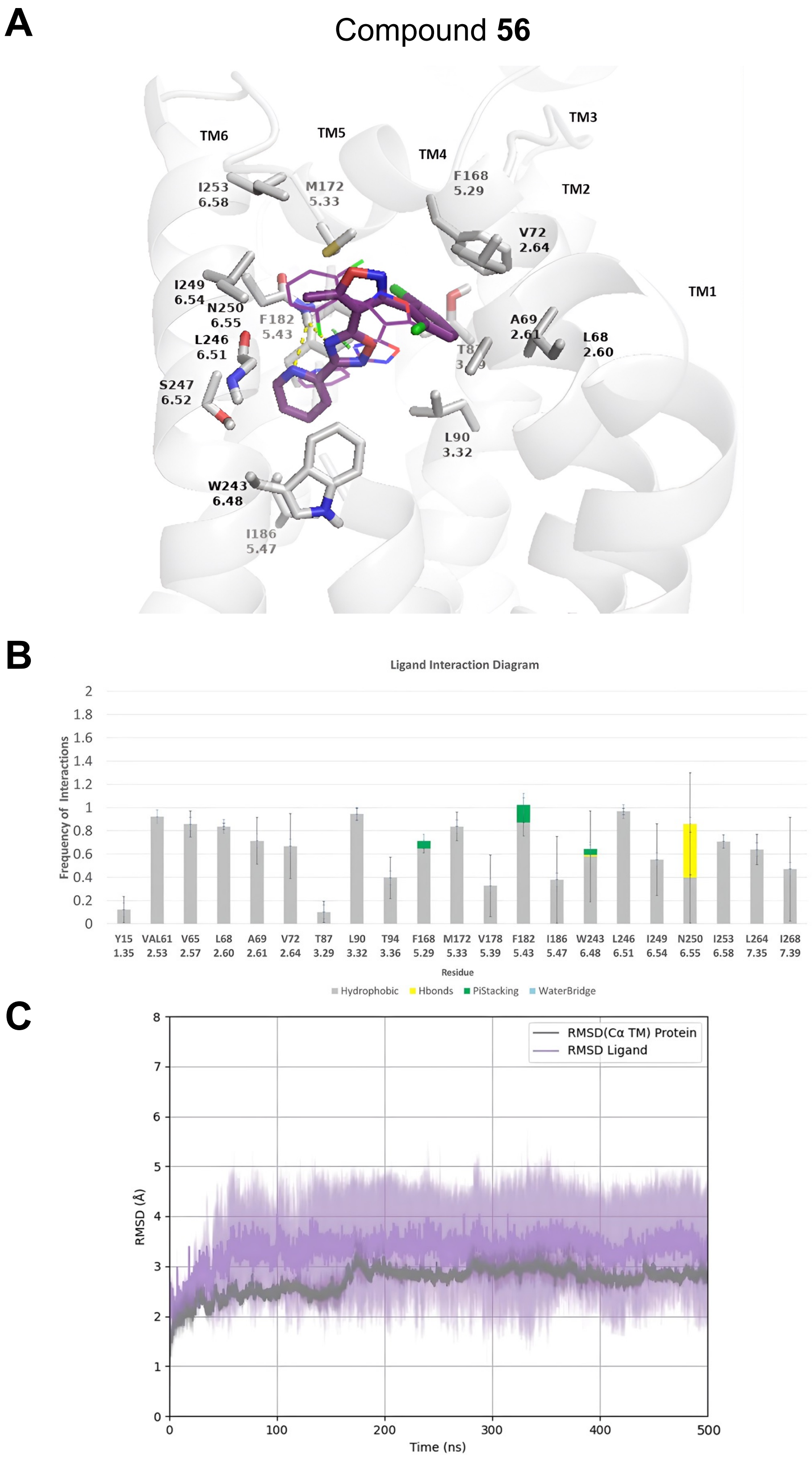

Figure S6. Continued

D

Compound **57**

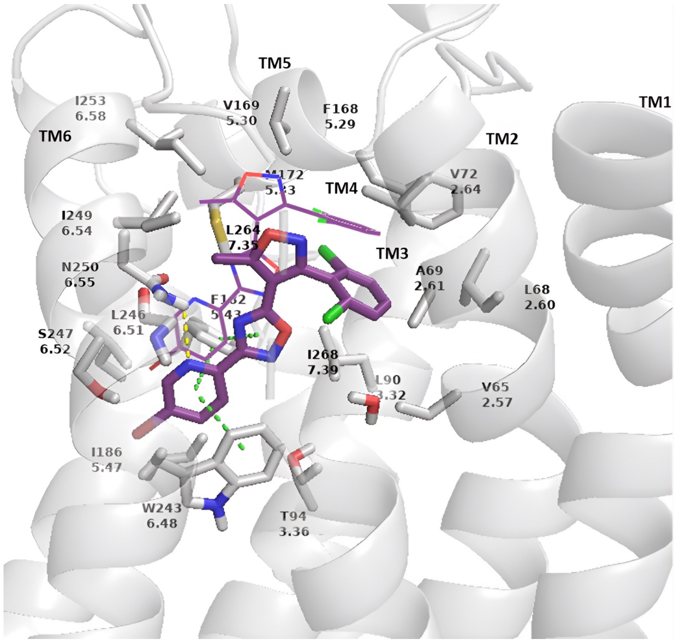

E

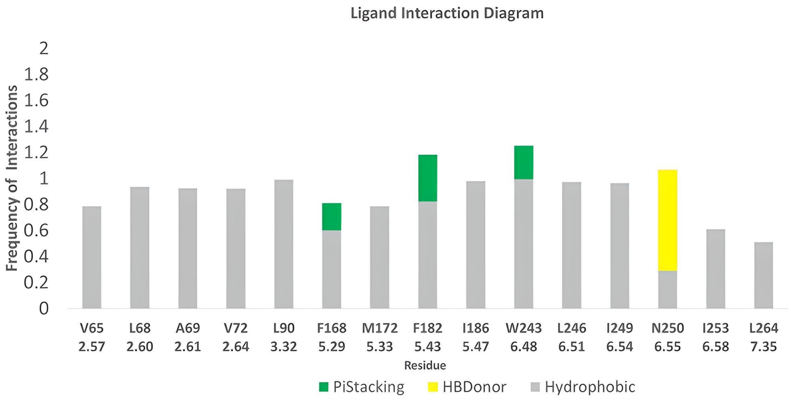

F

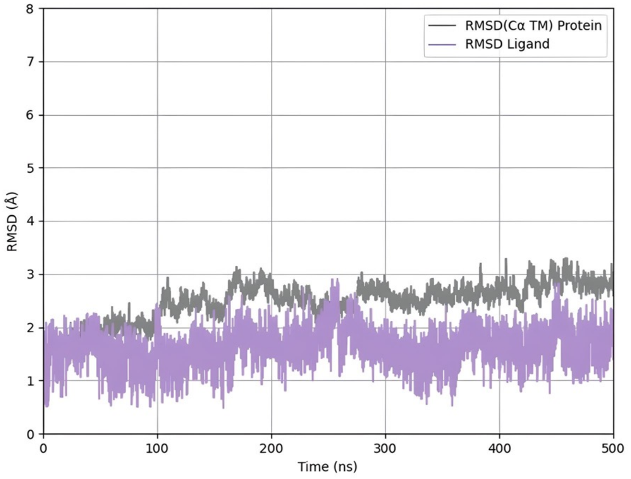

Figure S6. Continued

G Compound **60**

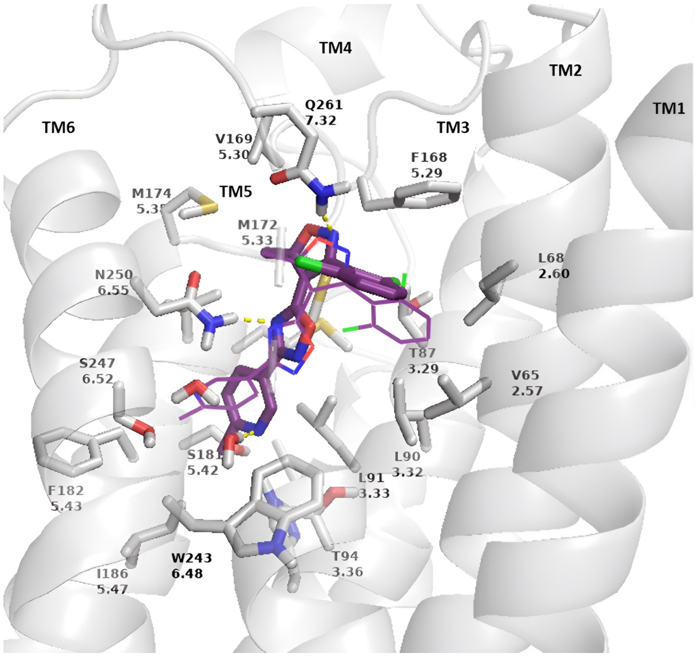

H

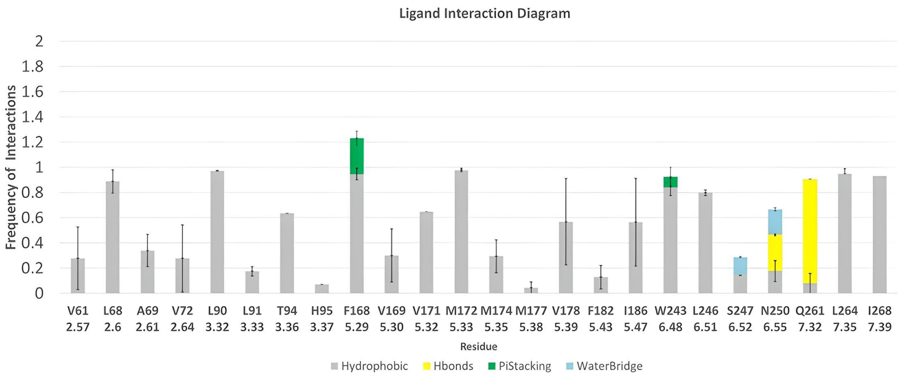

I

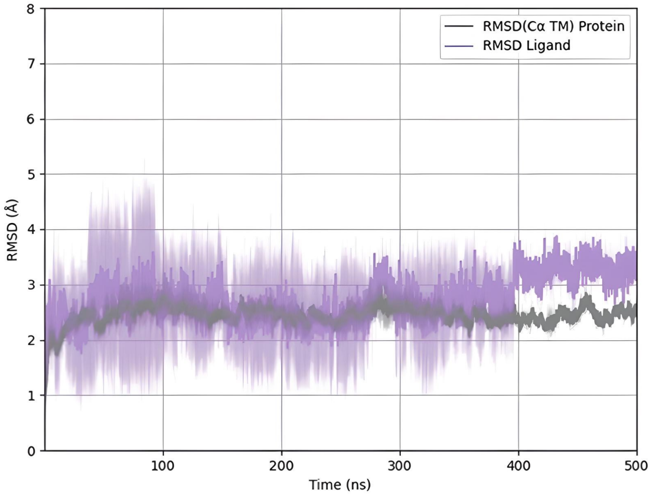

Figure S6. 500ns-MD simulations for the complex of compounds **56**, **57** and **60** with the WT hA_3R using the amber ff19sb.[9](A), (D), (G) Representative frame of the ligandinside the orthosteric binding area. (B), (E), (H) Receptor-ligand interaction frequency histograms; bars are plotted only for residues with interaction frequencies ≥ 0.2. Color figure in frames or bar plots: ligand is shown with pink sticks and ligand's starting position with an orange wire, receptor is shown with a white cartoon and sticks, hydrogen bonding interactions are shown with yellow dashes or bars, π-π interactions are shown with green dashes or bars; hydrophobic interactions are shown with grey bars; water bridges are shown with blue bars. In (C), (F), (I) are shown the RMSD plots of Ca carbons of the protein (blue line) and of heavy atoms of the ligand (orange line) are shown. For MD simulations we used a revised model of the inactive form of hA_3R we have recently published, [8] generated using the multi-state AF2 method [5,6] of hA_3R generated from GPCRdb [10] web-tool; the complexes of the starting structure (docking pose) and final snapshot from the MD simulations are available as pdb files (see Ancillary Information).

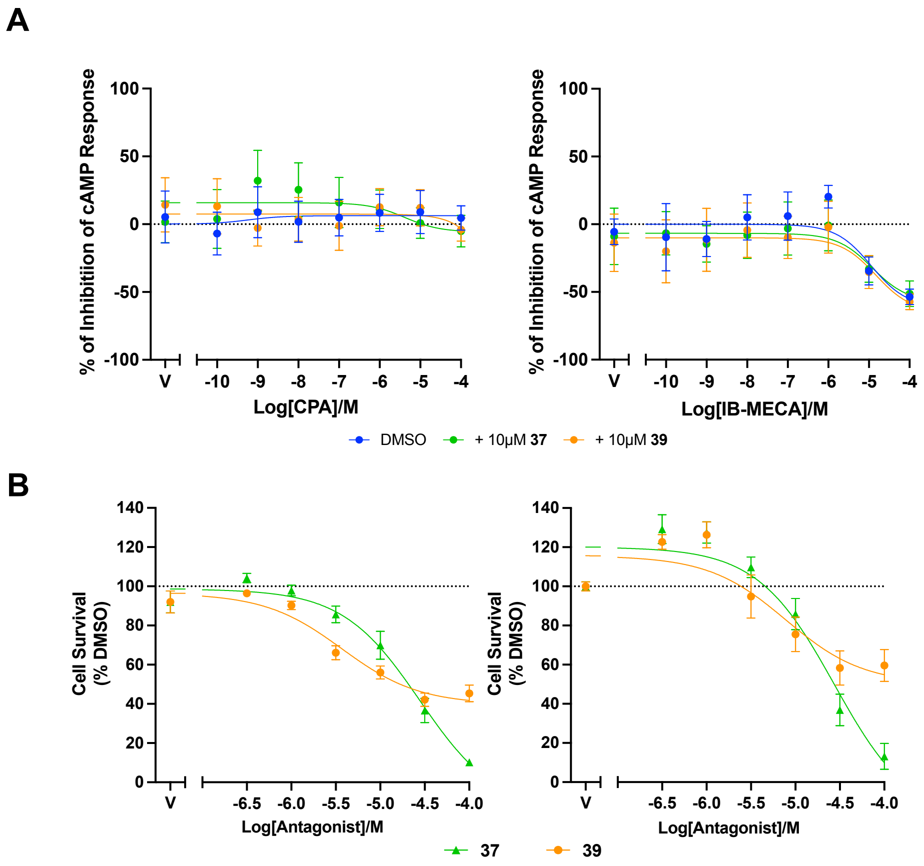

Figure S7. (A) Inhibition of forskolin-mediated cAMP accumulation in LK-2 cells by **CPA** or **IB-MECA**, co-treated with DMSO, or 10μM **37** or **39**. Cells were treated with 1μM **forskolin** and 10μM compound (or DMSO) and stimulated with **CPA** or **IB-MECA**. Data are expressed as percent inhibition. (B) Inhibition of NCI-H1792 (left) or LK-2 (right) cell proliferation by **37** or **39**. Cells were treated for 72-hours, before counting using the cell-counting kit-8. Data are expressed as percentage survival, relative to cells treated with DMSO alone.

TI/MD calculations

In the oxadiazole series the affinity of 3-pyridinyl derivative **59** (*K*i = 91.2 nM) is higher compared to 2-pyridinyl derivative **56** (*K*i = 192 nM) due to the stronger hydrogen bonding interactions in **59** between 4-oxadiazolyl nitrogen and amide side chain of N250$^{6.55}$ and because an additional hydrogen bond between oxazolyl nitrogen and amide side chain of Q261$^{7.32}$ can be formed in **59**. The calculated relative binding free energy for **56** → **59** $\Delta\Delta G_{b,exp}$ = - 0.45±0.08 kcal mol^{-1} with $\Delta\Delta G_{b,TI/MD}$ = 0.26±0.08 kcal mol^{-1} and deviation 0.71 kcal mol^{-1}. When a 4-bromine was added at 2-pyridinyl group in **56** resulting in compound **57** the binding free energy changes for **56** → **57** are $\Delta\Delta G_{b,exp}$ = 0.57±0.14 kcal mol^{-1} and $\Delta\Delta G_{b,TI/MD}$ = - 0.48±0.05 kcal mol^{-1} with deviation 1.05 kcal mol^{-1}. The addition of a 4-methyl substituent at 3-pyridinyl group in compound **59** results in compound **60** results and the binding free energy changes are $\Delta\Delta G_{b,exp}$ = - 0.27±0.07 kcal mol^{-1} with $\Delta\Delta G_{b,TI/MD}$ = 0.07±0.05 kcal mol^{-1} and deviation 0.34 kcal mol^{-1}.

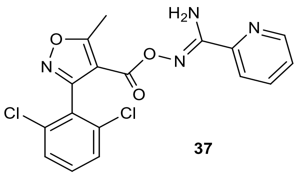

37

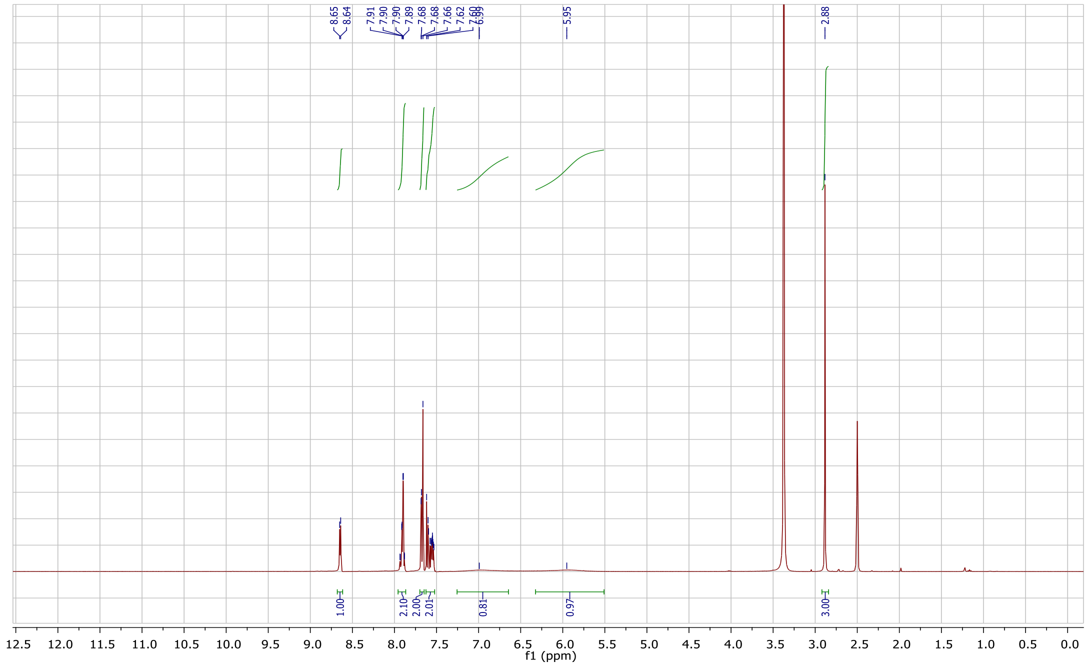

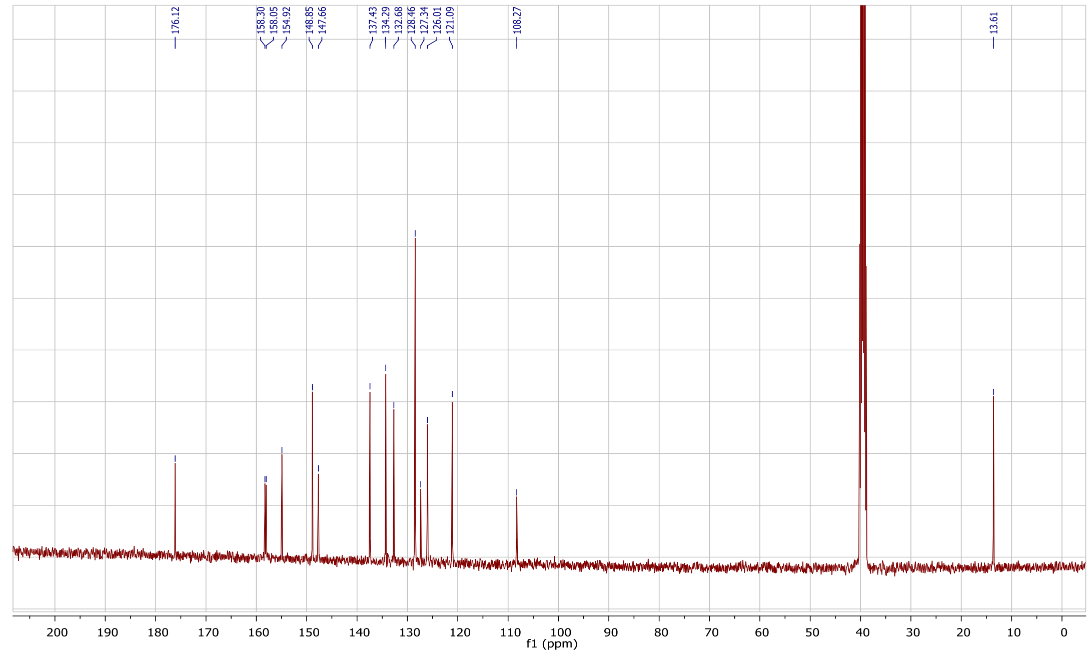

^{1}H and ^{13}C NMR spectra of compound **37** in DMSO-d_6.

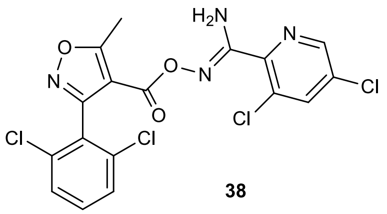

38

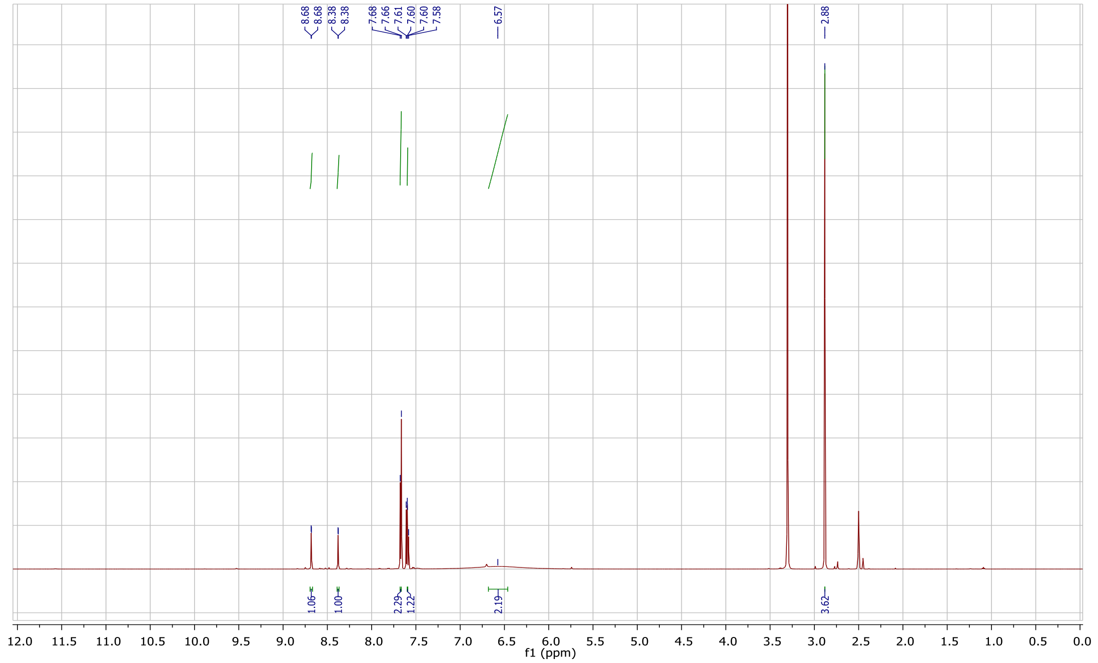

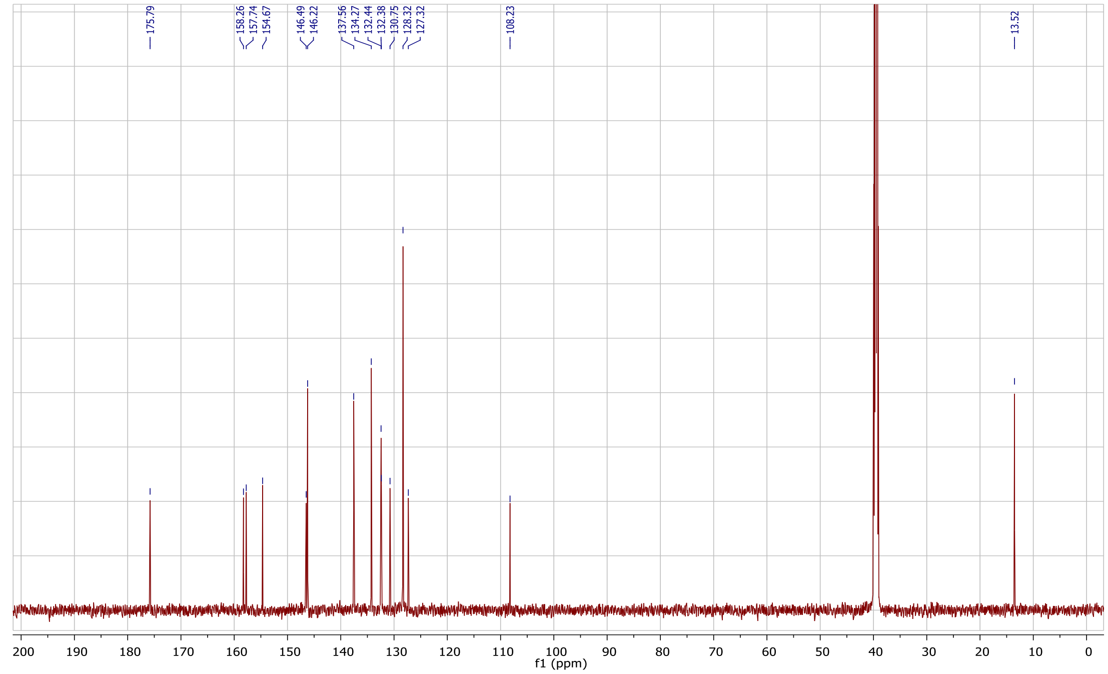

^{1}H and ^{13}C NMR spectra of compound **38** in DMSO-d_6.

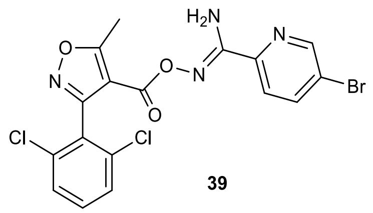

39

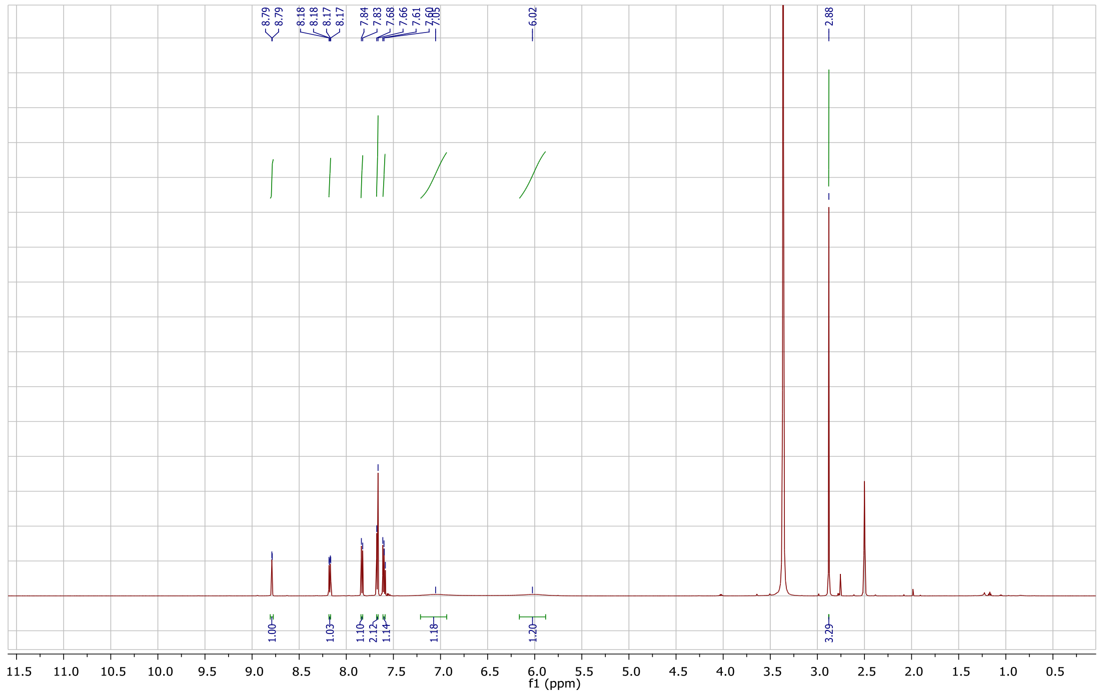

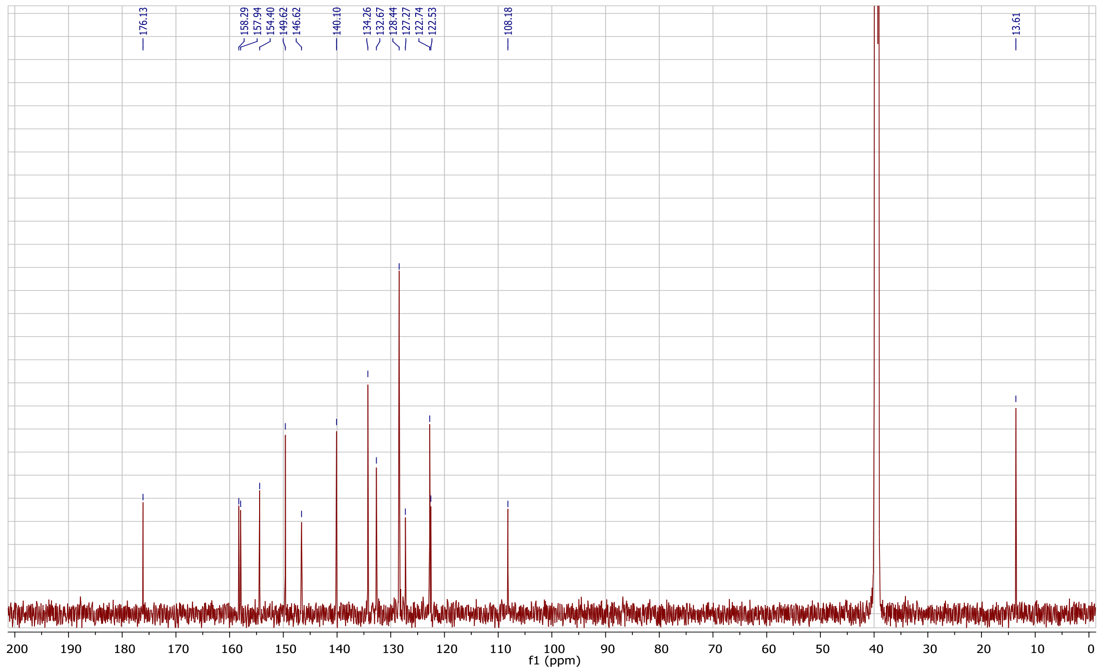

^{1}H and ^{13}C NMR spectra of compound **39** in DMSO-d_6.

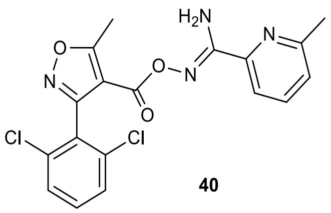

40

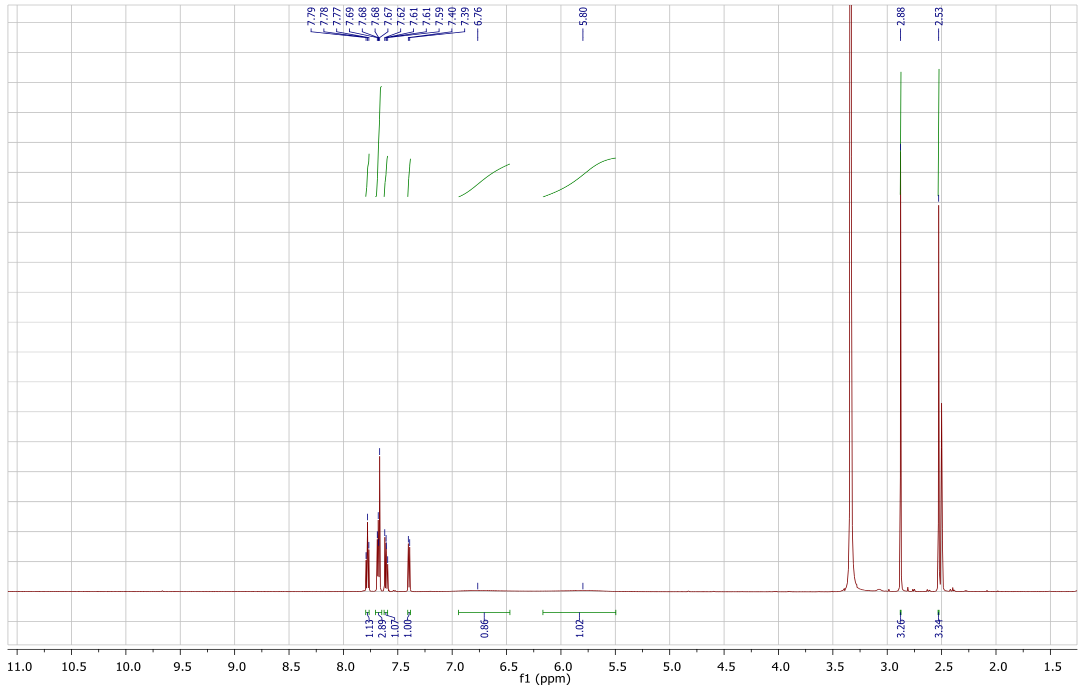

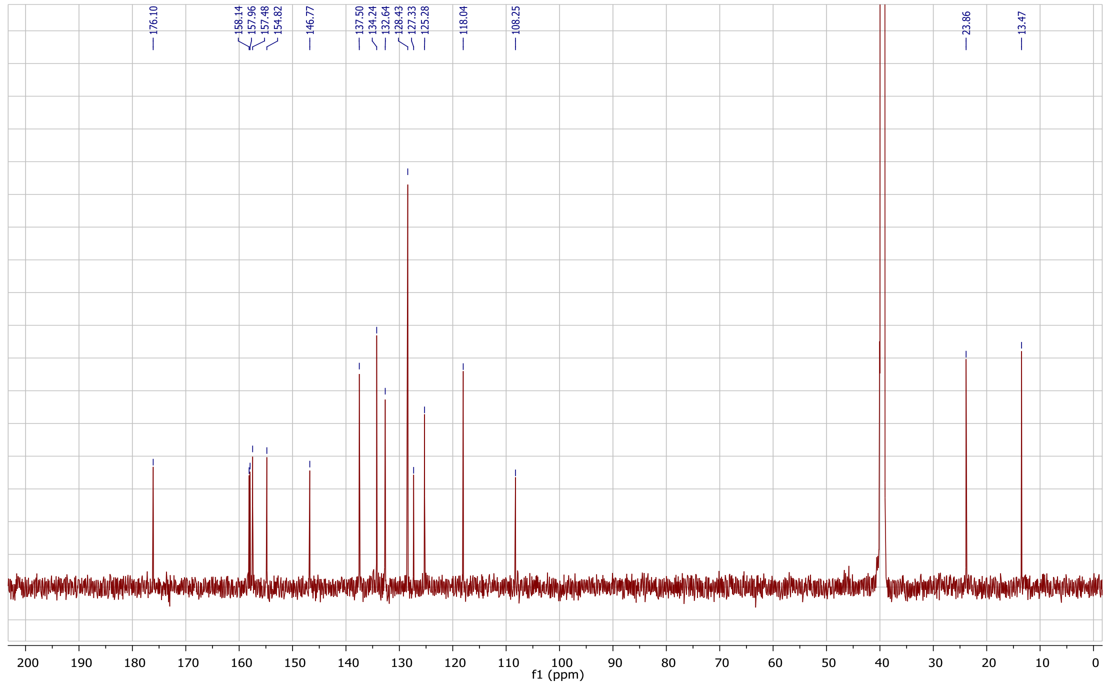

^{1}H and ^{13}C NMR spectra of compound **40** in DMSO-d_6.

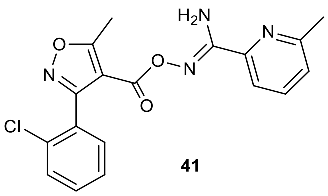

41

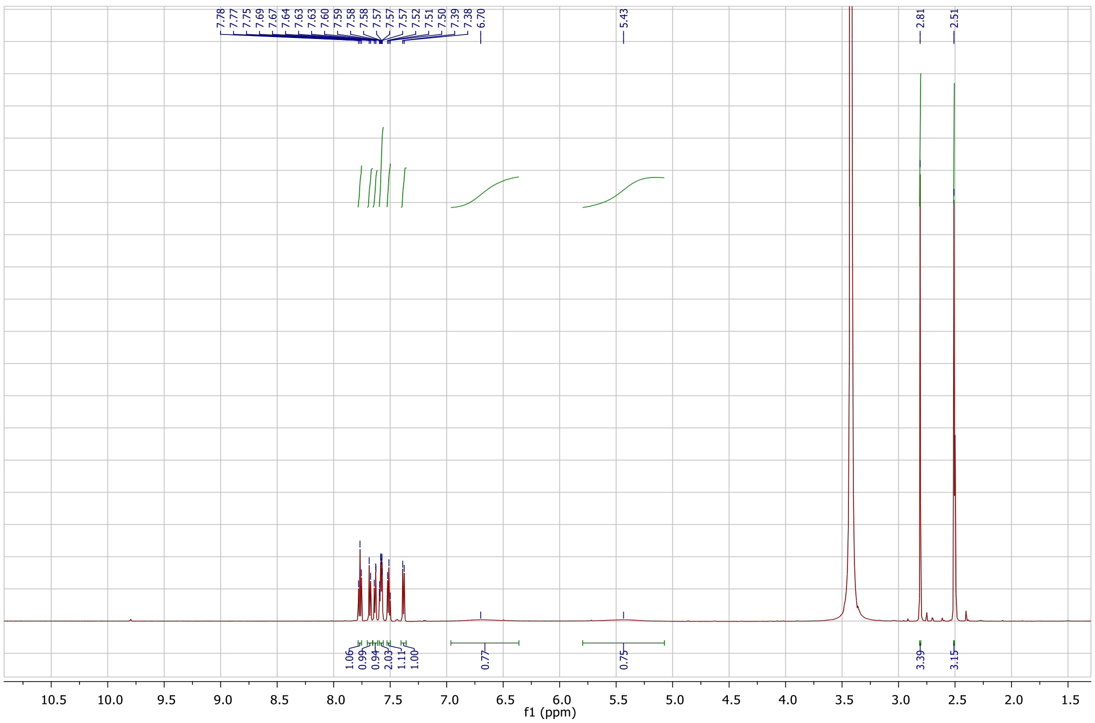

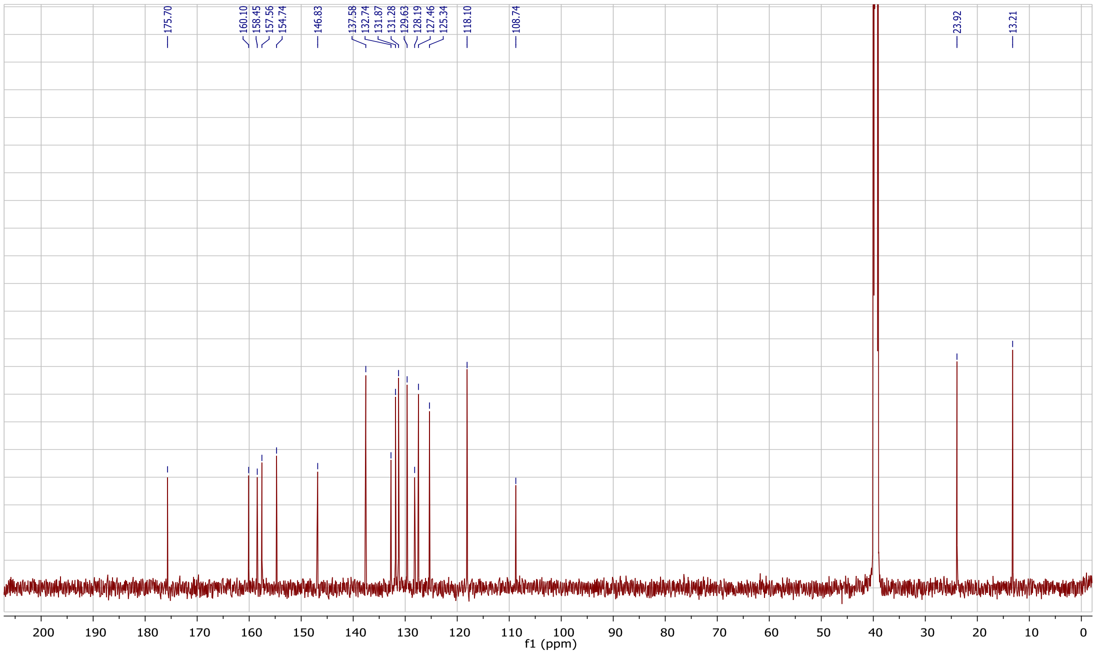

^{1}H and ^{13}C NMR spectra of compound **41** in DMSO-d_6.

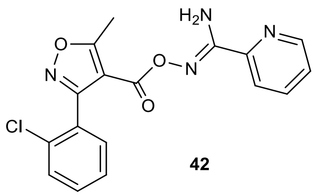

42

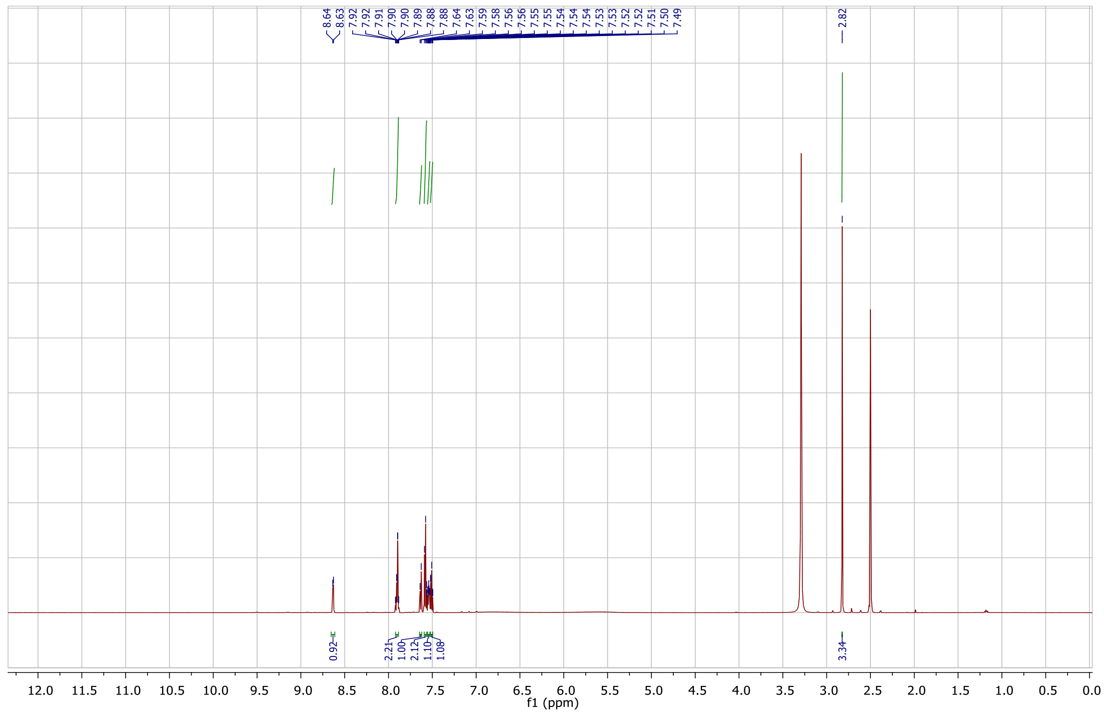

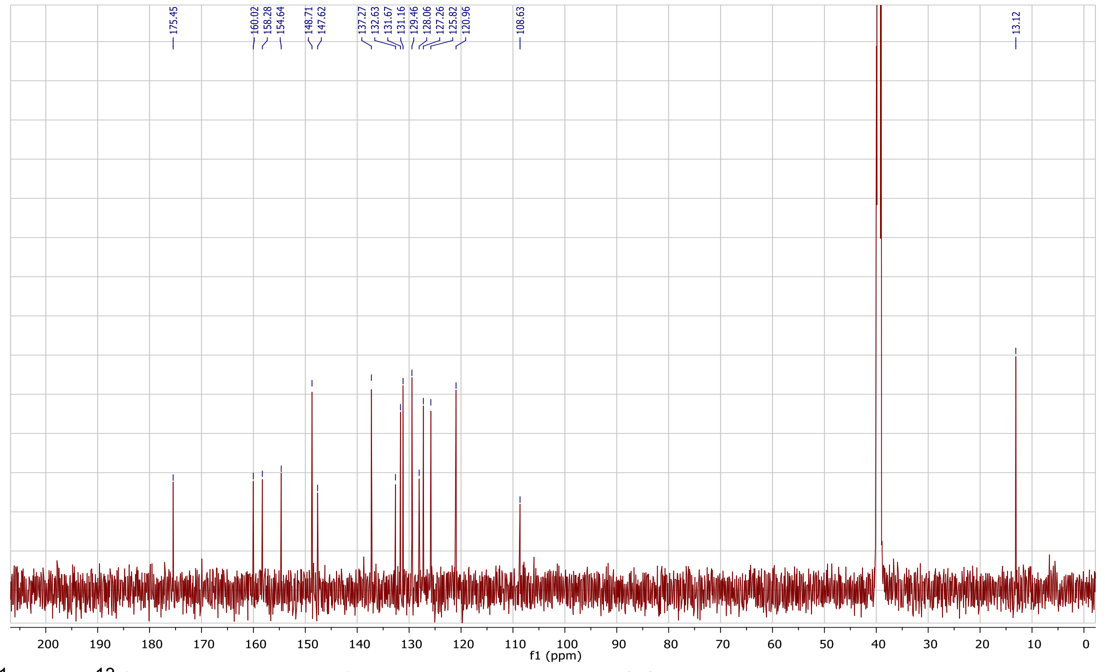

^{1}H and ^{13}C NMR spectra of compound **42** in DMSO-d_6.

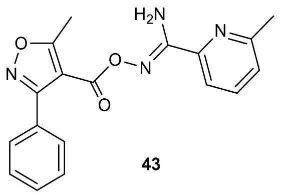

43

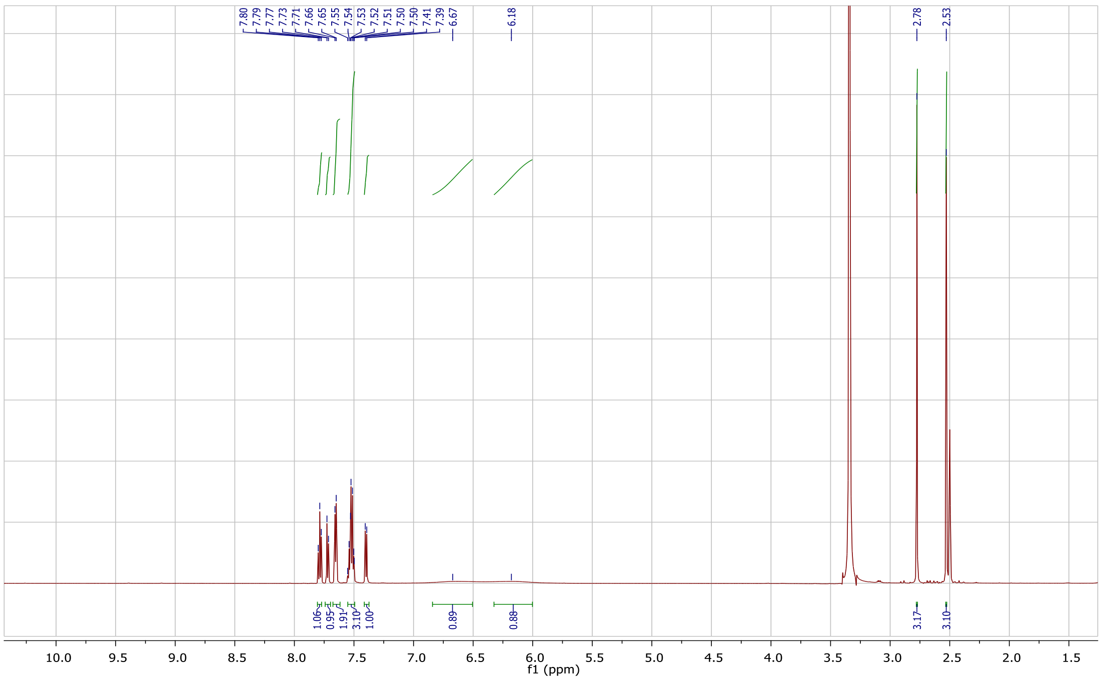

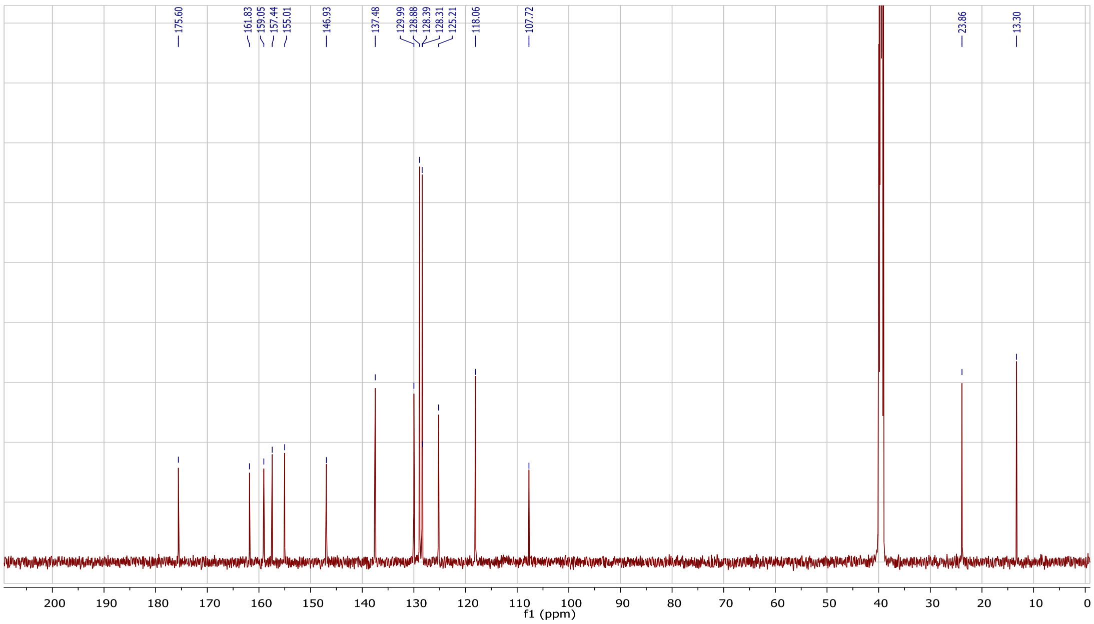

^{1}H and ^{13}C NMR spectra of compound **43** in DMSO-d_6.

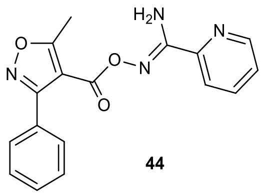

44

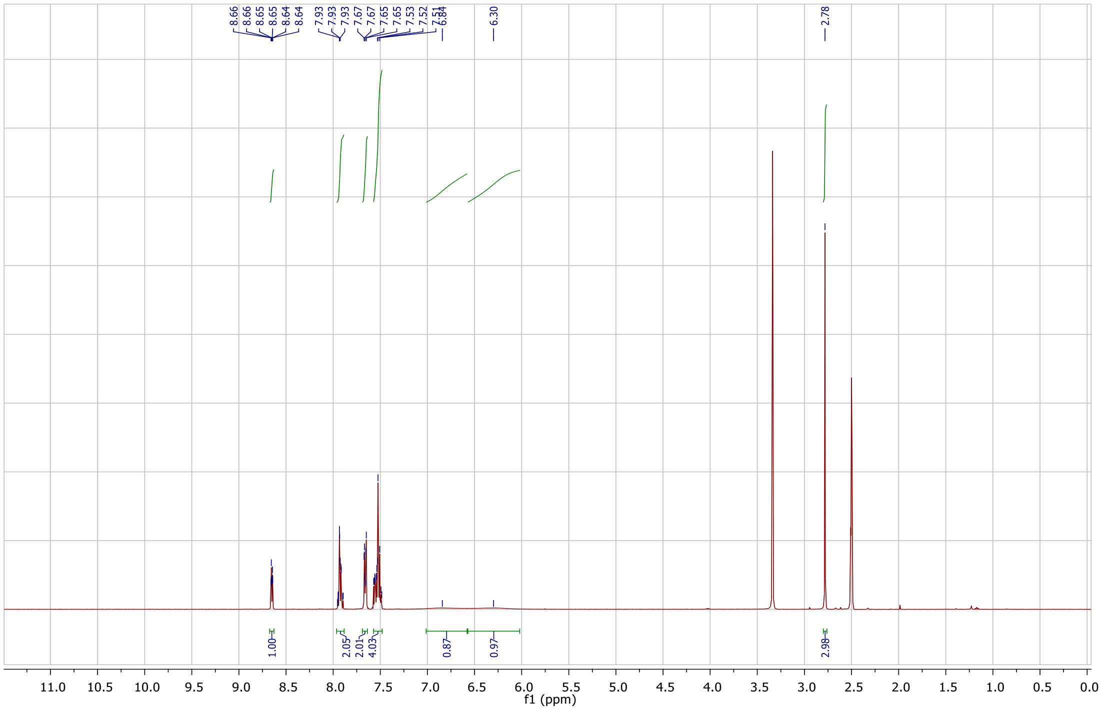

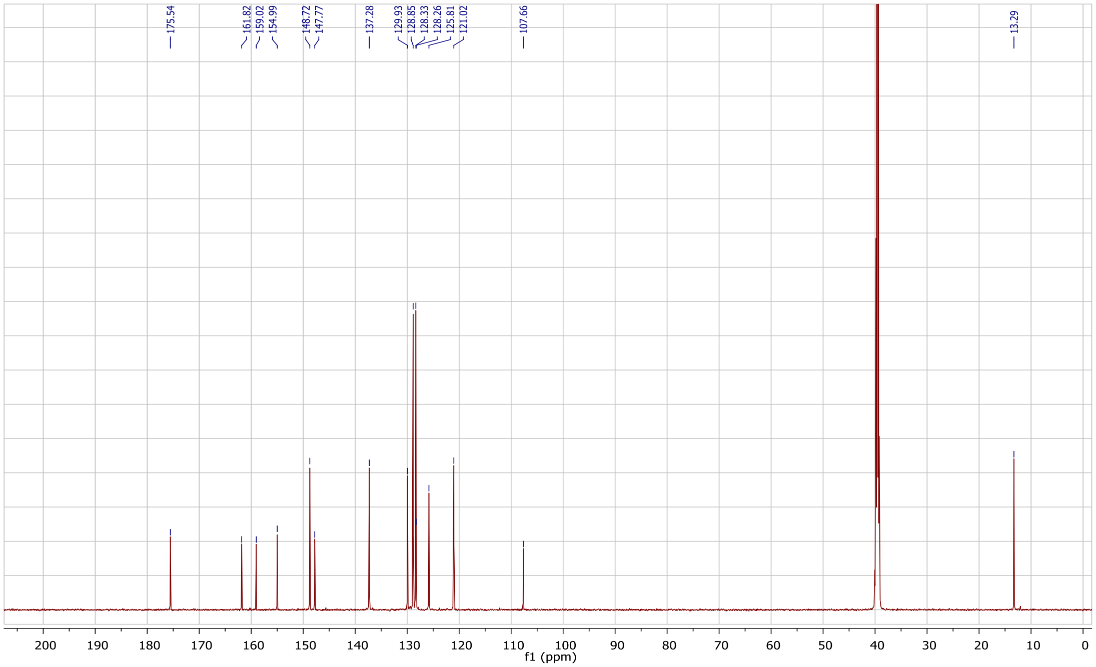

^{1}H and ^{13}C NMR spectra of compound **44** in DMSO-d_6.

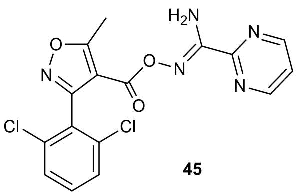

45

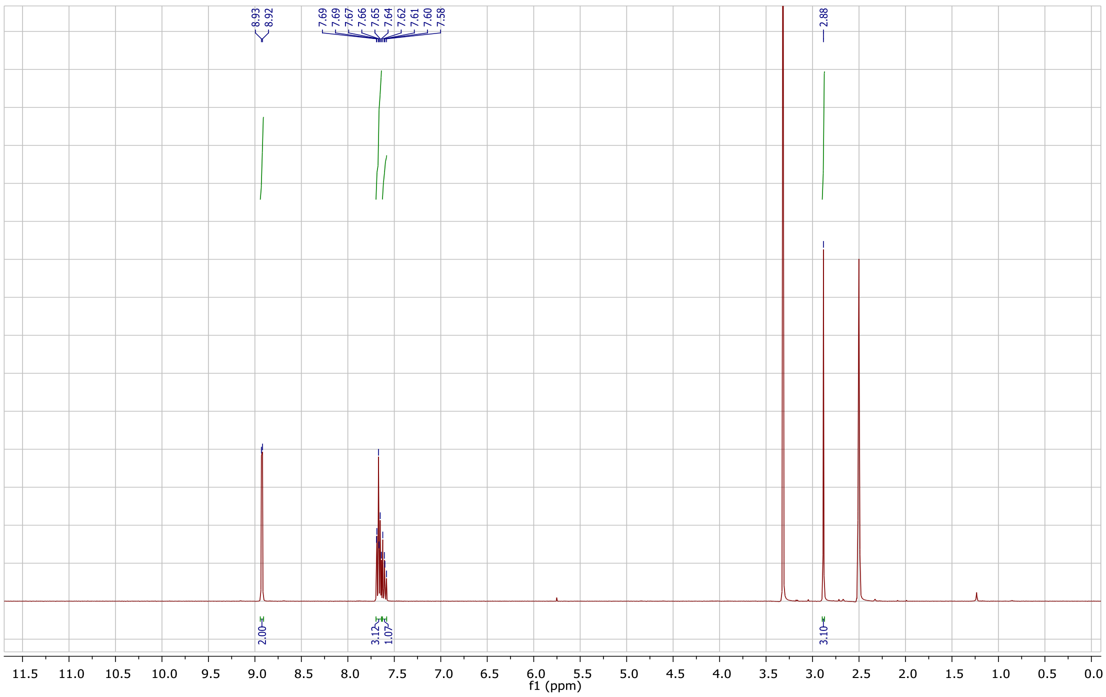

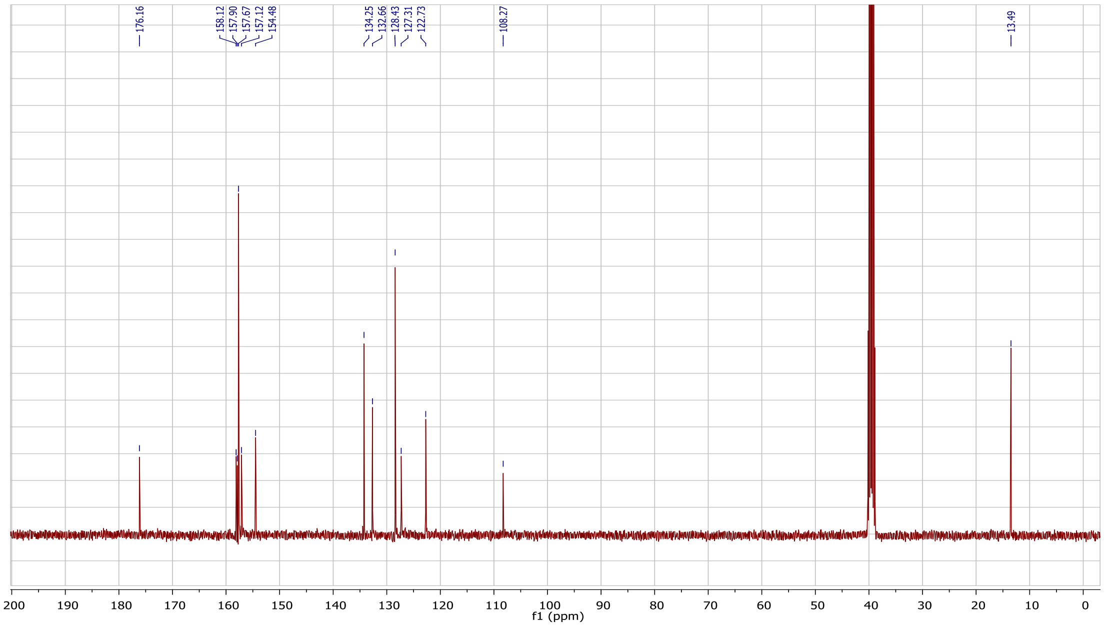

^{1}H and ^{13}C NMR spectra of compound **45** in DMSO-d_6.

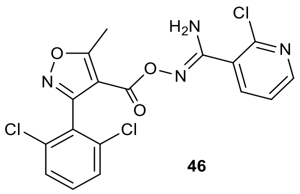

46

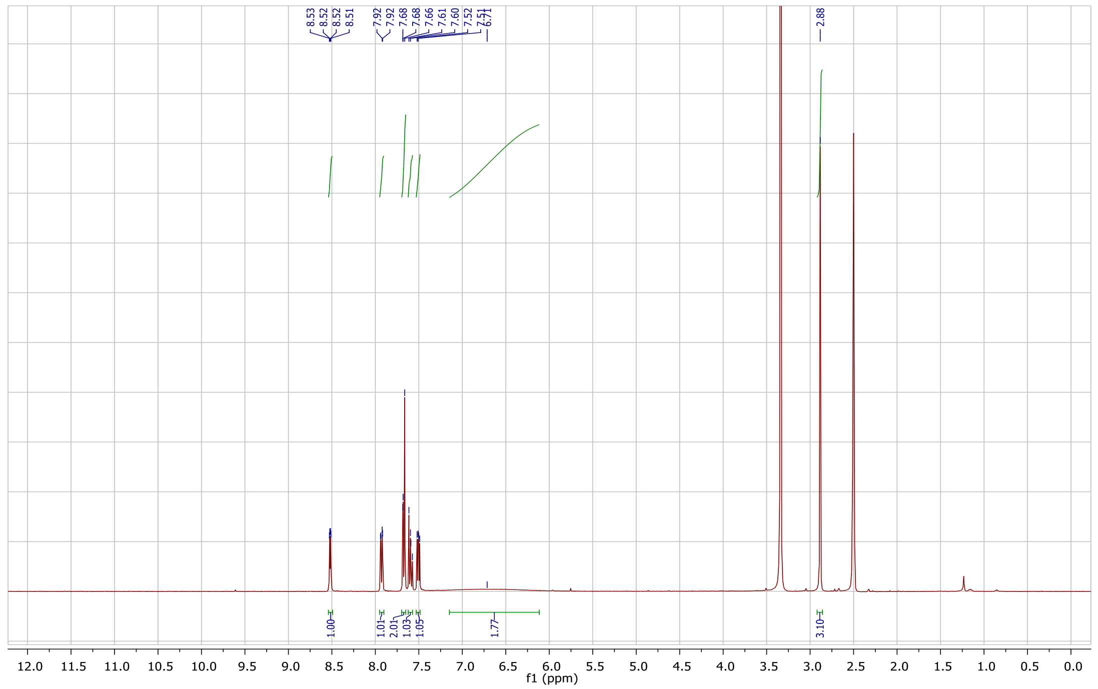

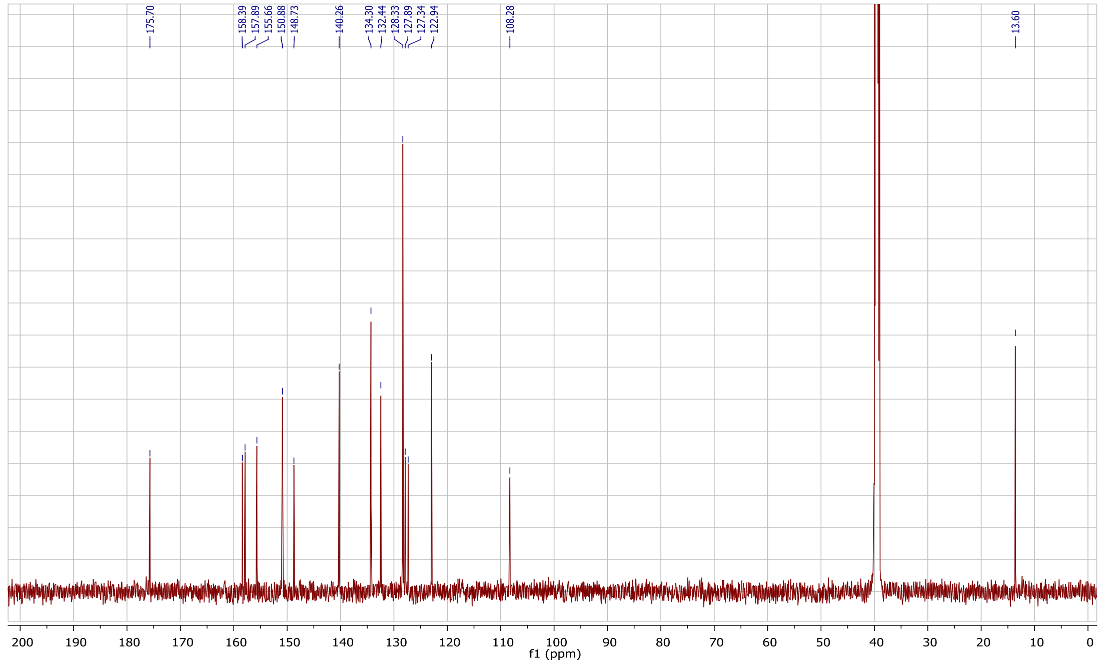

^{1}H and ^{13}C NMR spectra of compound **46** in DMSO-d_6.

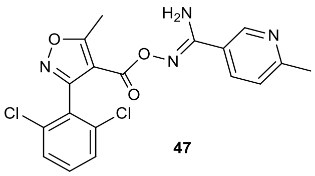

47

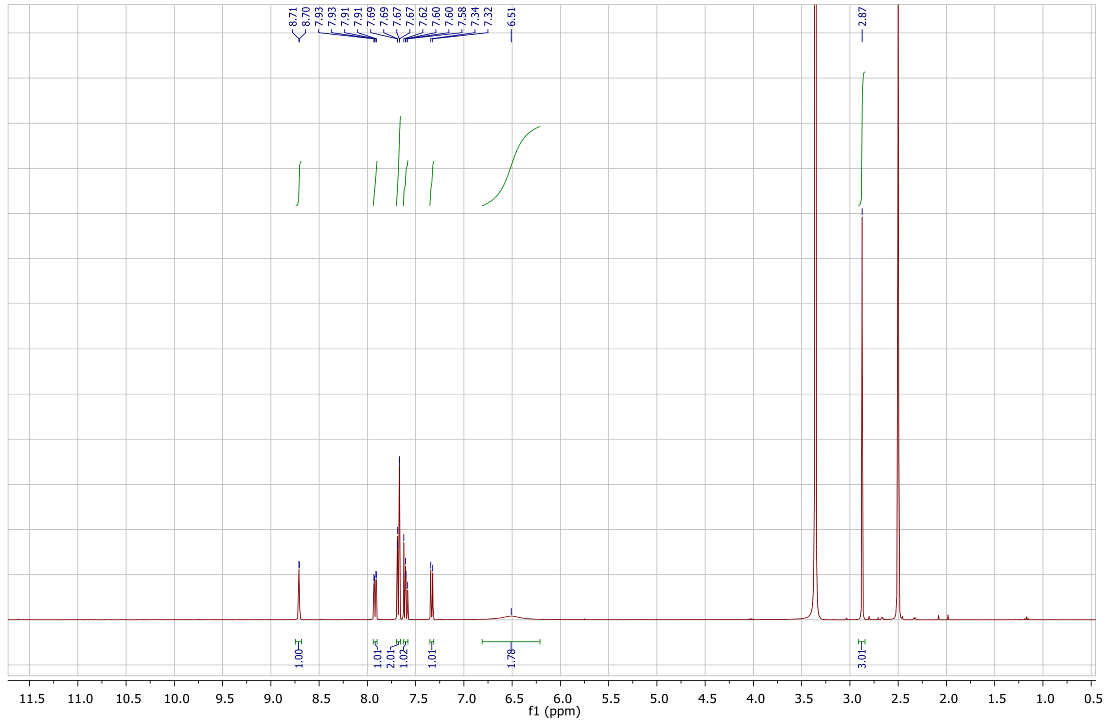

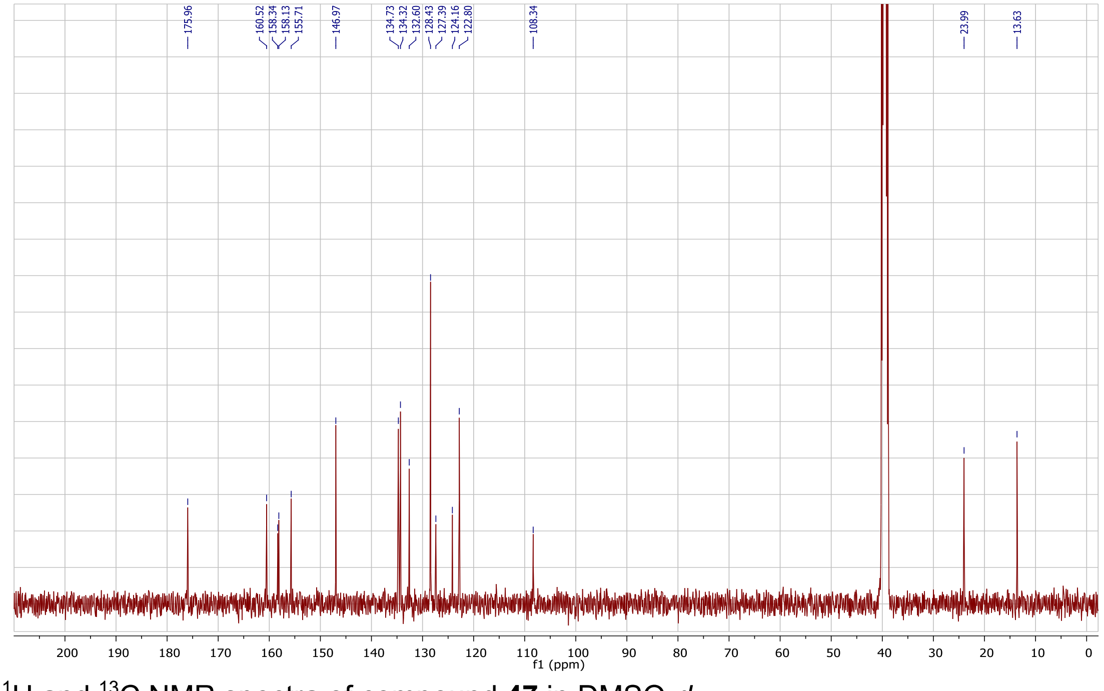

^{1}H and ^{13}C NMR spectra of compound **47** in DMSO-d_6.

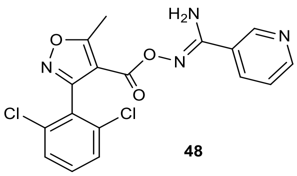

48

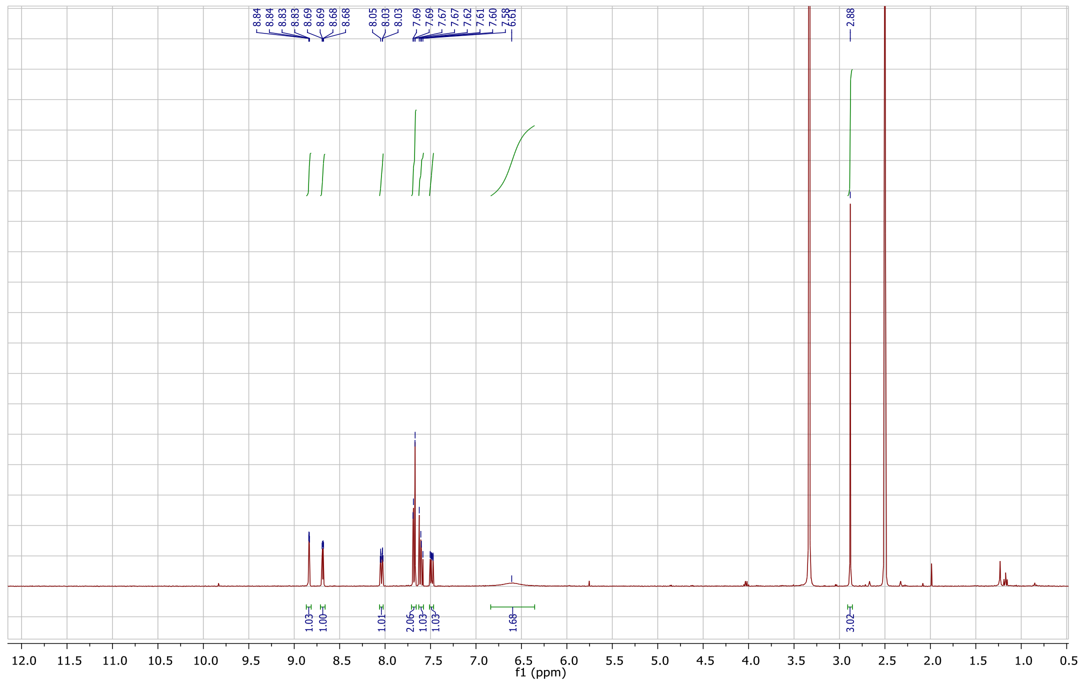

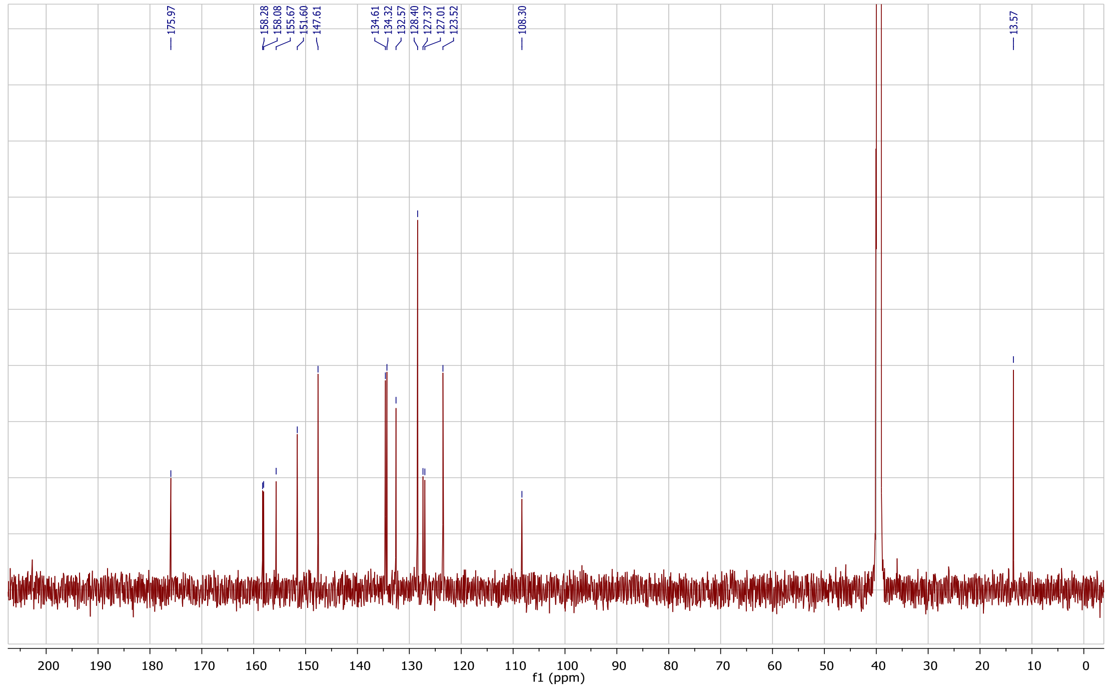

^{1}H and ^{13}C NMR spectra of compound **48** in DMSO-d_6.

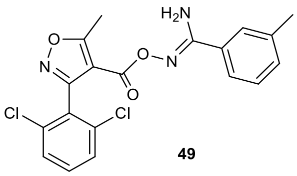

49

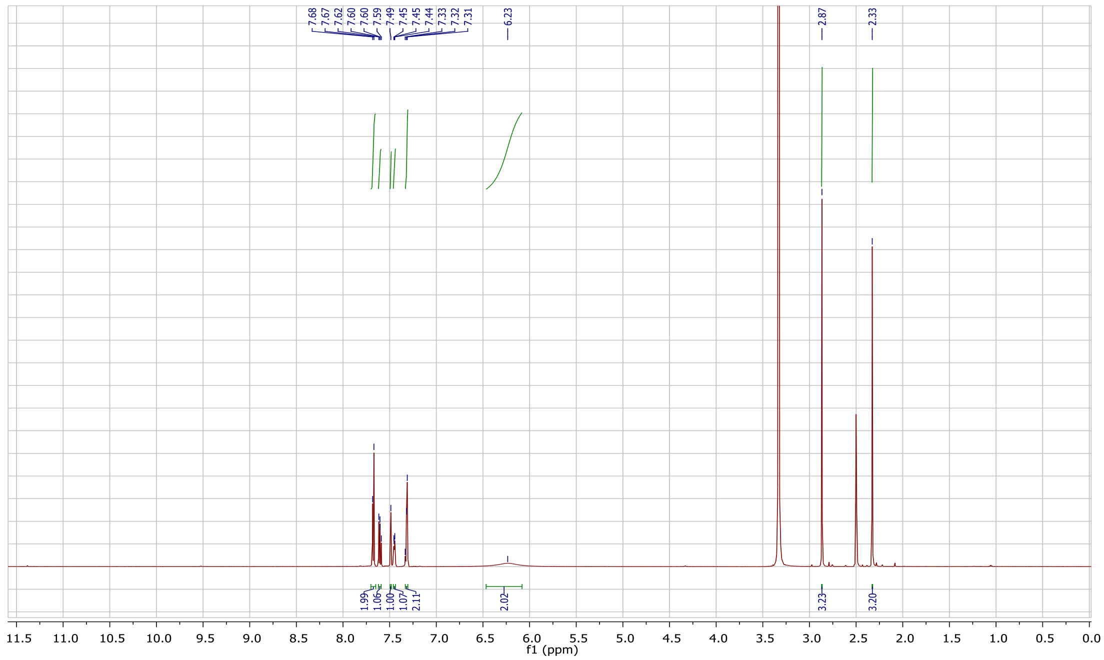

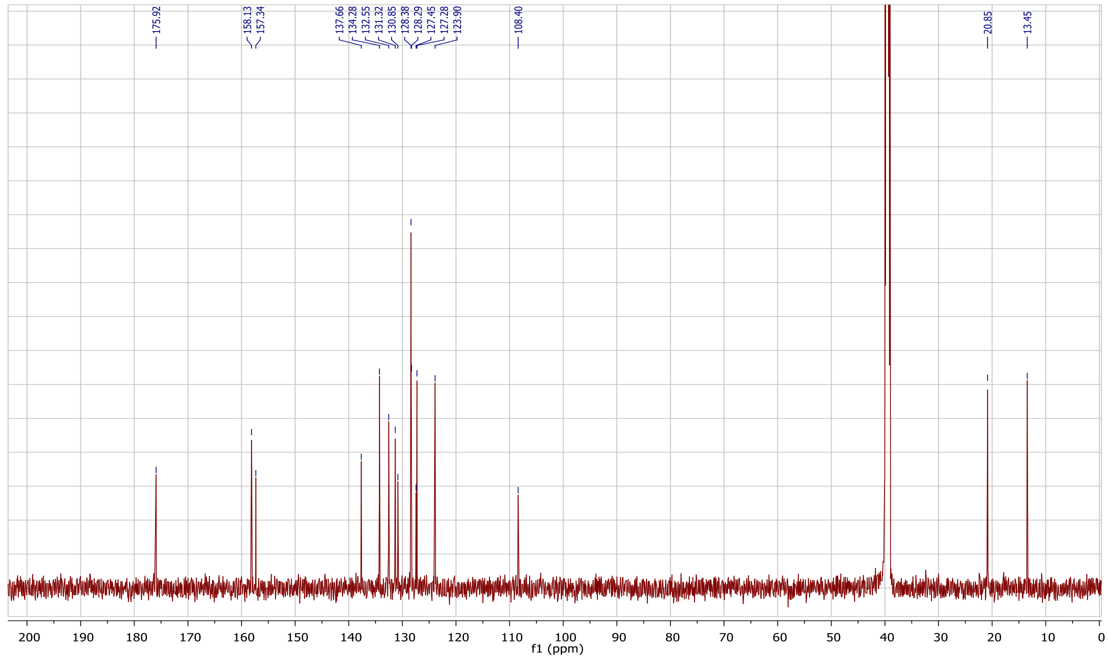

^{1}H and ^{13}C NMR spectra of compound **49** in DMSO-d_6.

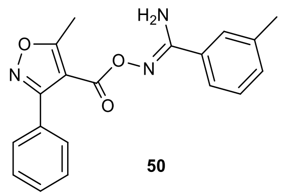

50

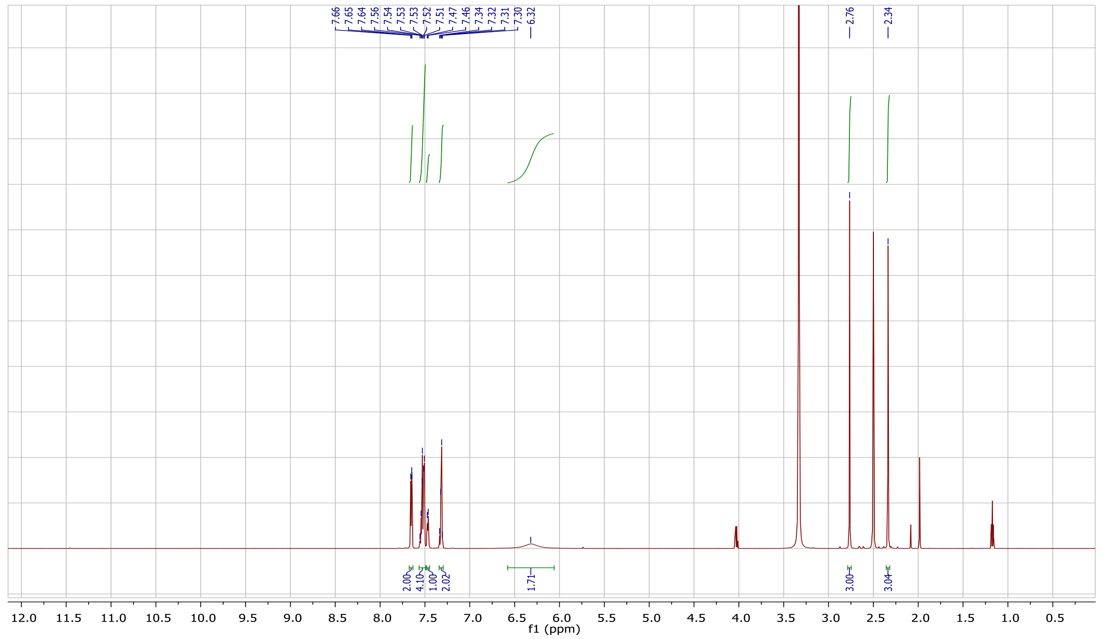

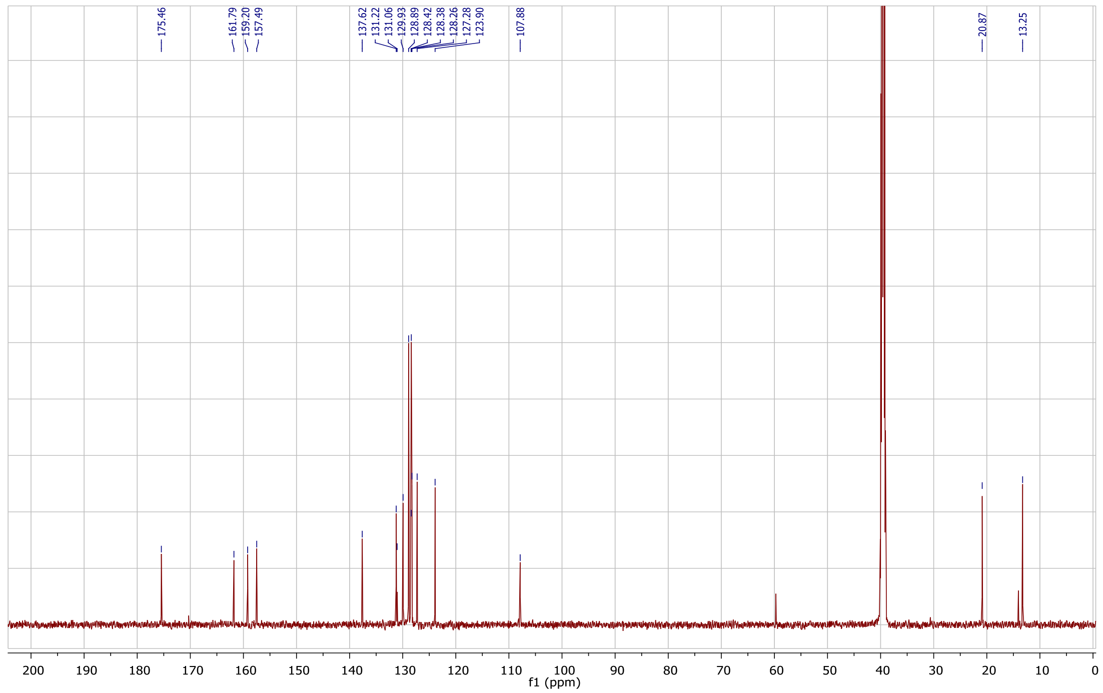

^{1}H and ^{13}C NMR spectra of compound **50** in DMSO-d_6.

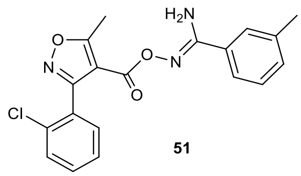

51

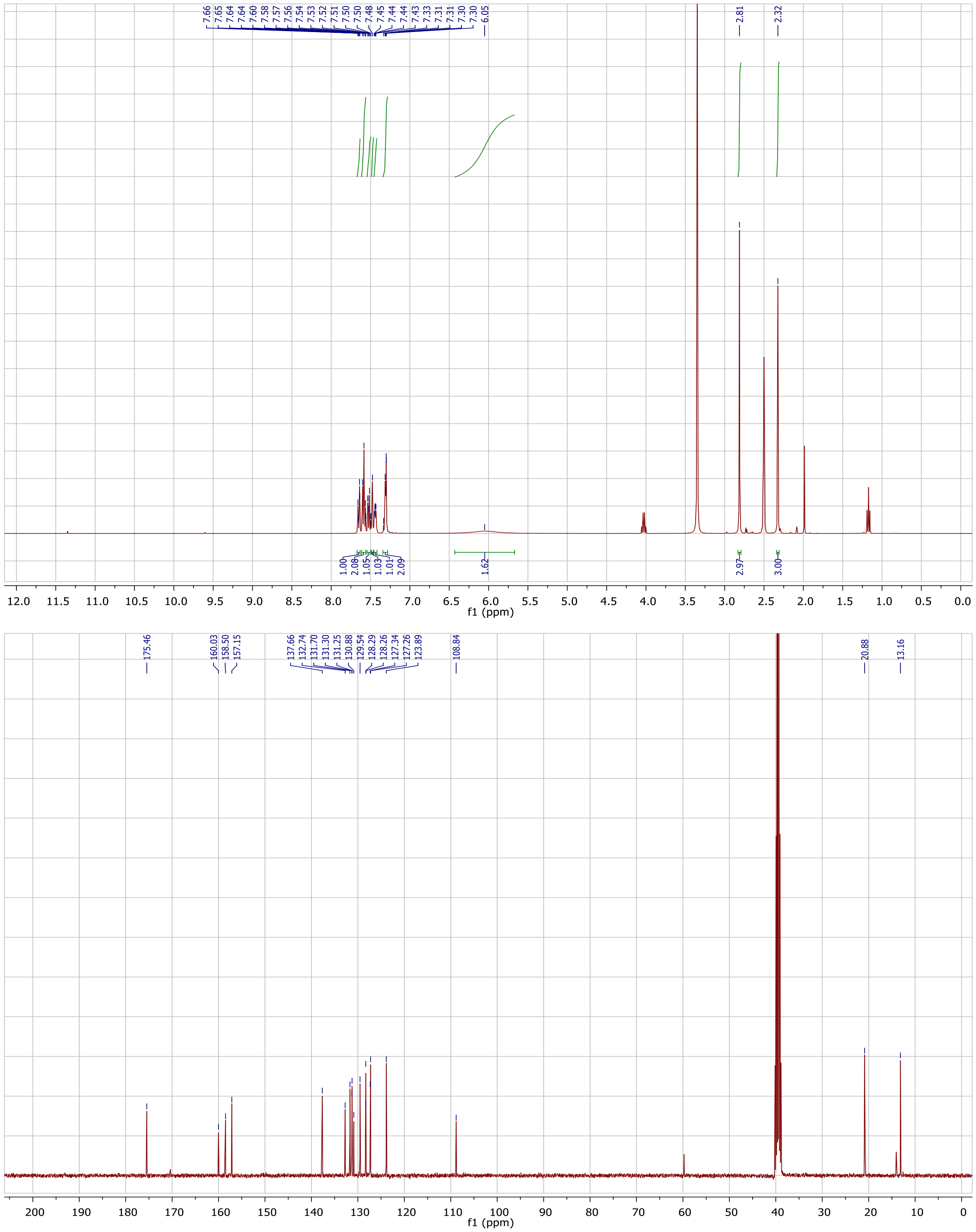

^{1}H and ^{13}C NMR spectra of compound **51** in DMSO-d_6.

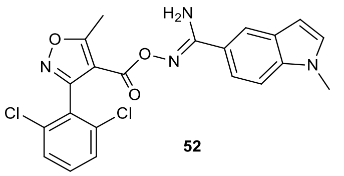

52

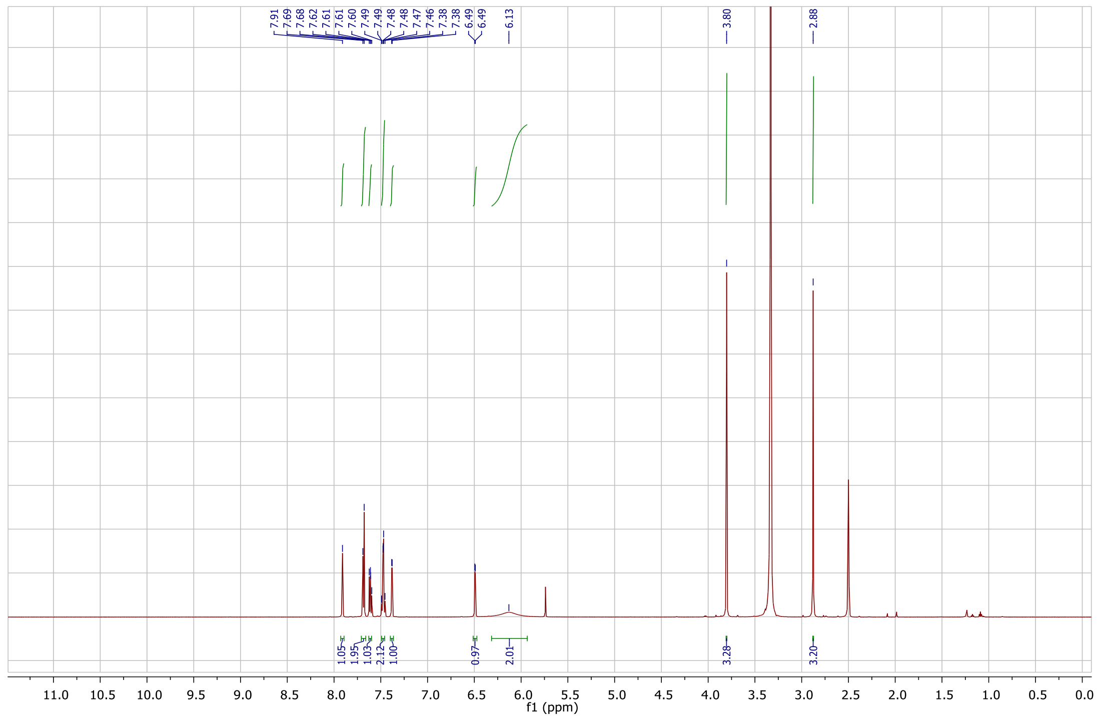

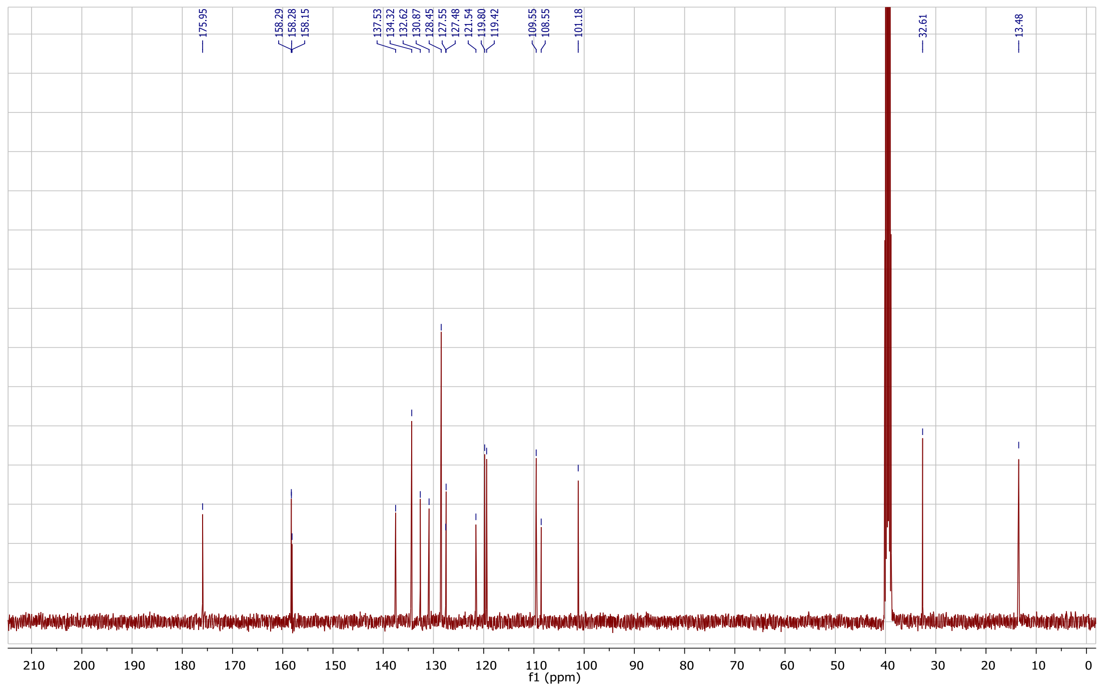

^{1}H and ^{13}C NMR spectra of compound **52** in DMSO-d_6.

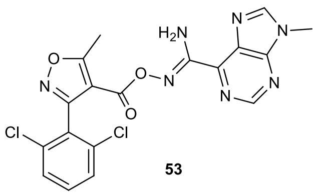

53

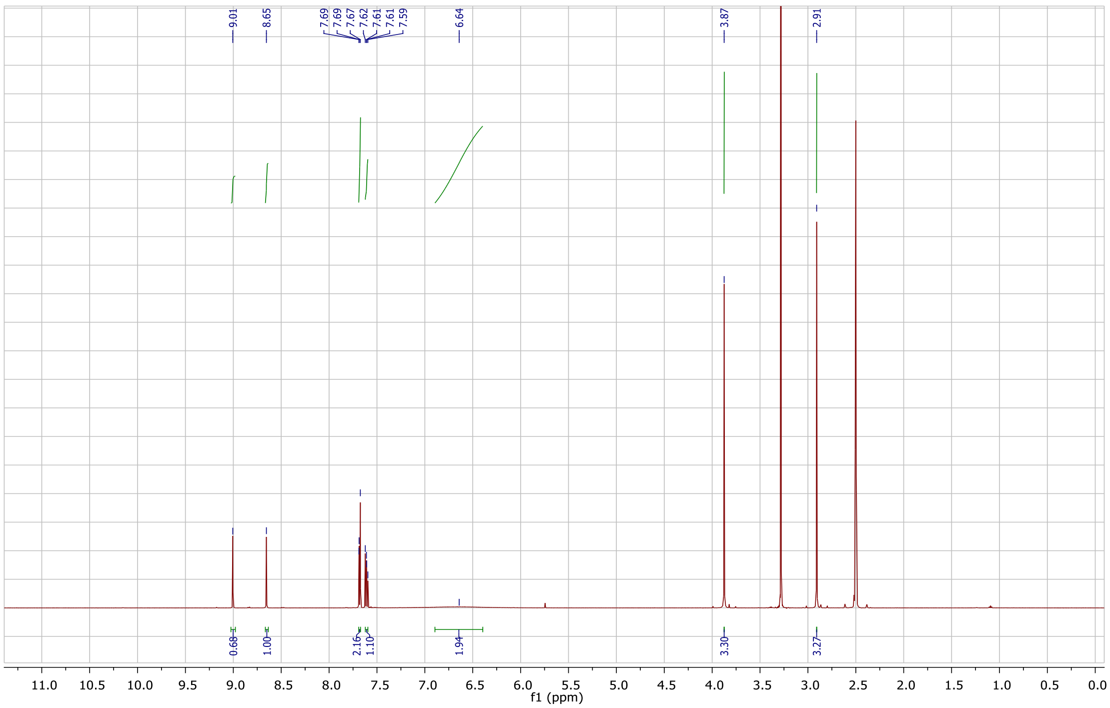

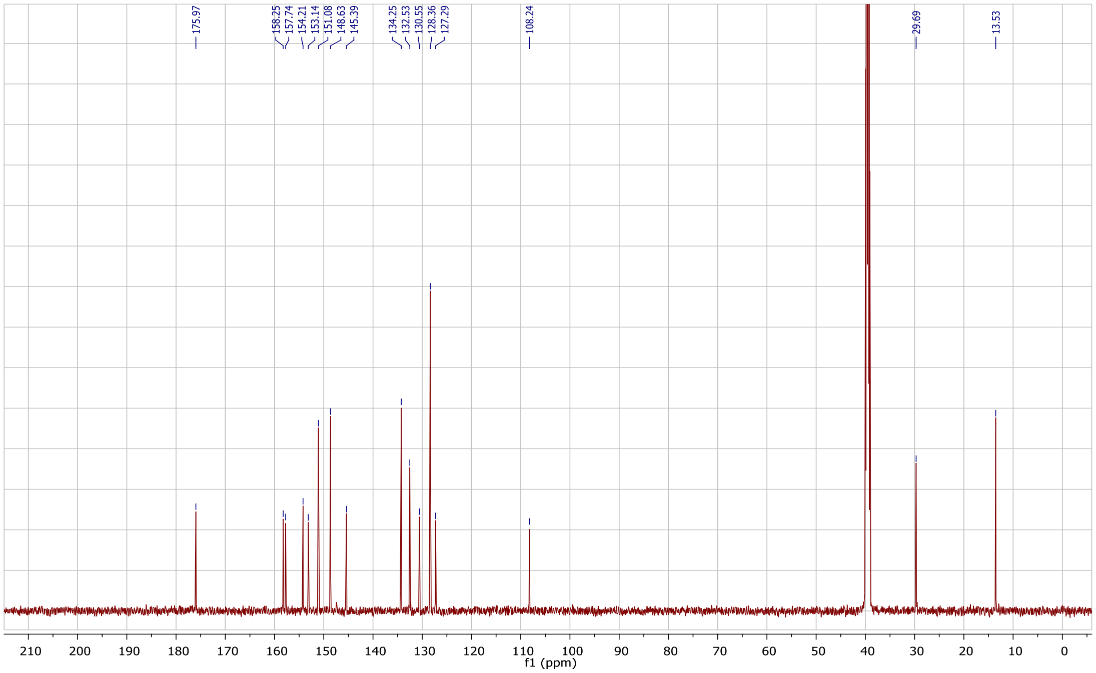

^{1}H and ^{13}C NMR spectra of compound **53** in DMSO-d_6.

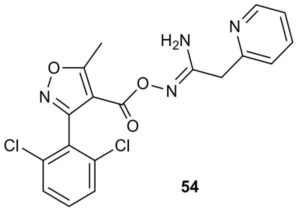

54

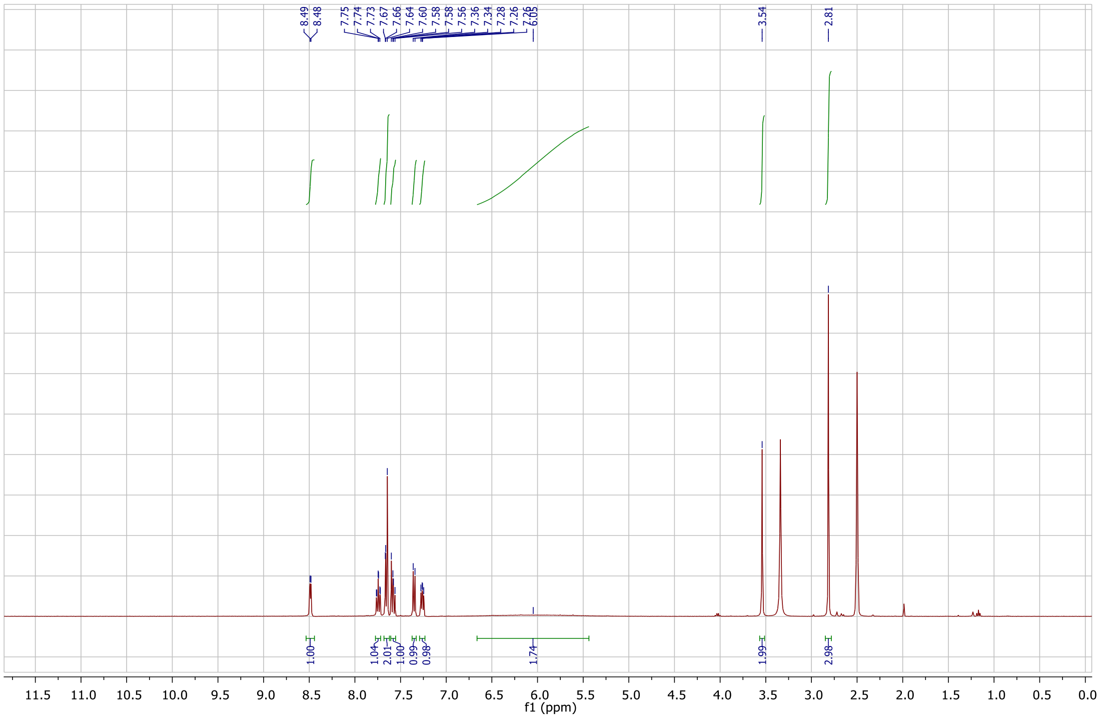

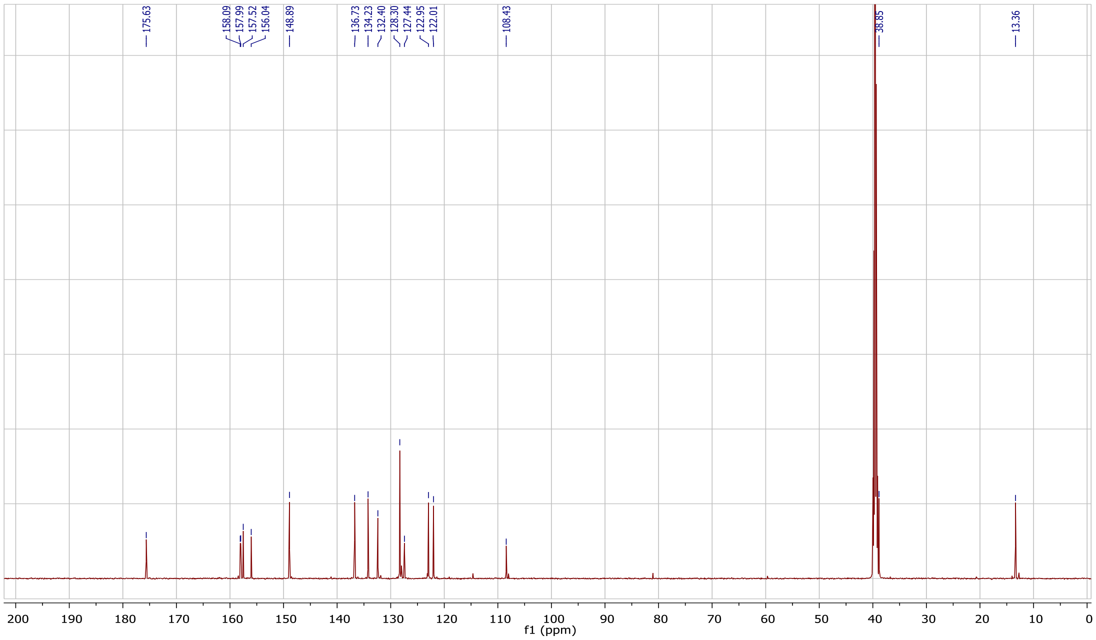

^{1}H and ^{13}C NMR spectra of compound **54** in DMSO-d_6.

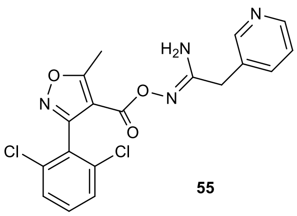

55

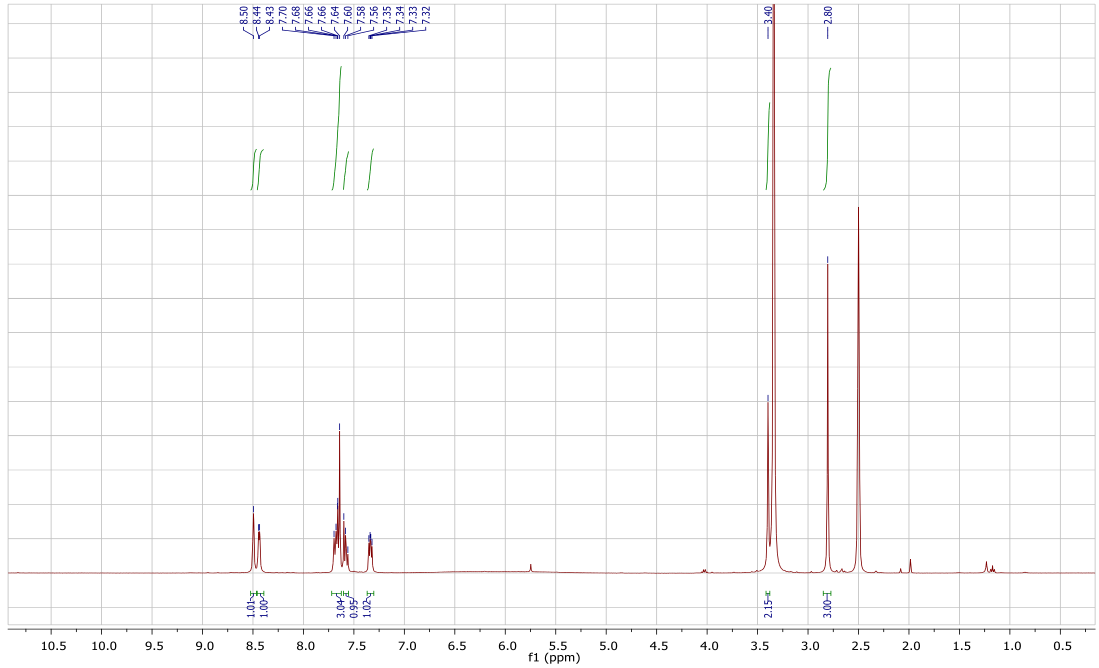

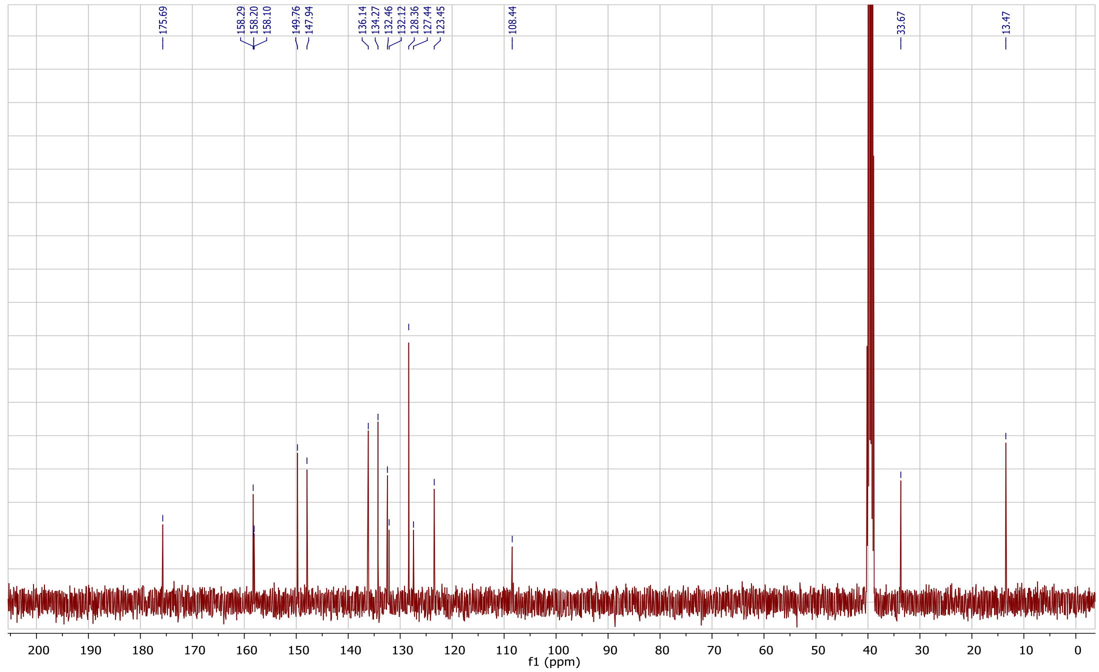

^{1}H and ^{13}C NMR spectra of compound **55** in DMSO-d_6.

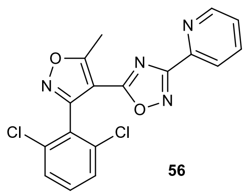

56

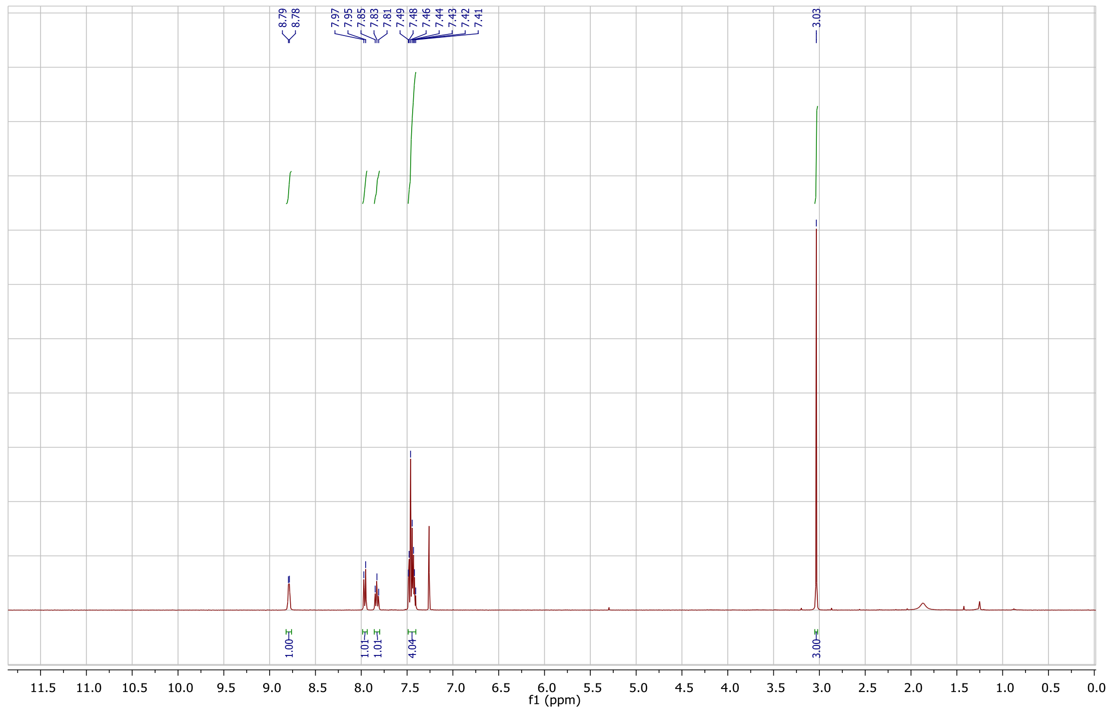

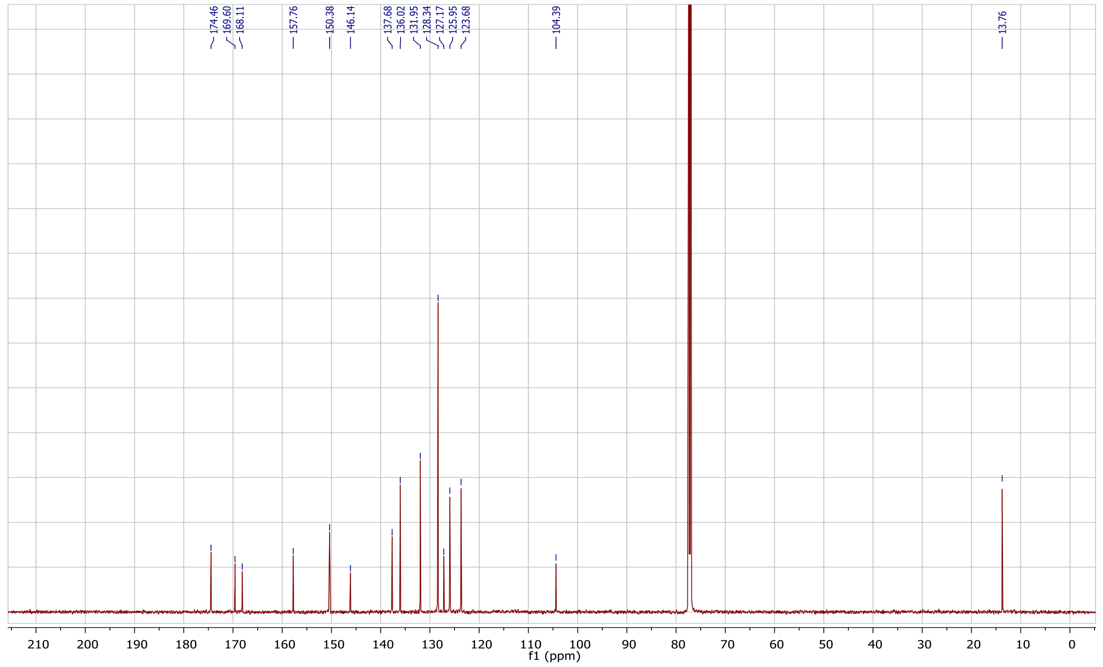

^{1}H and ^{13}C NMR spectra of compound **56** in $CDCl_3$.

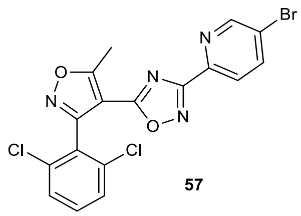

57

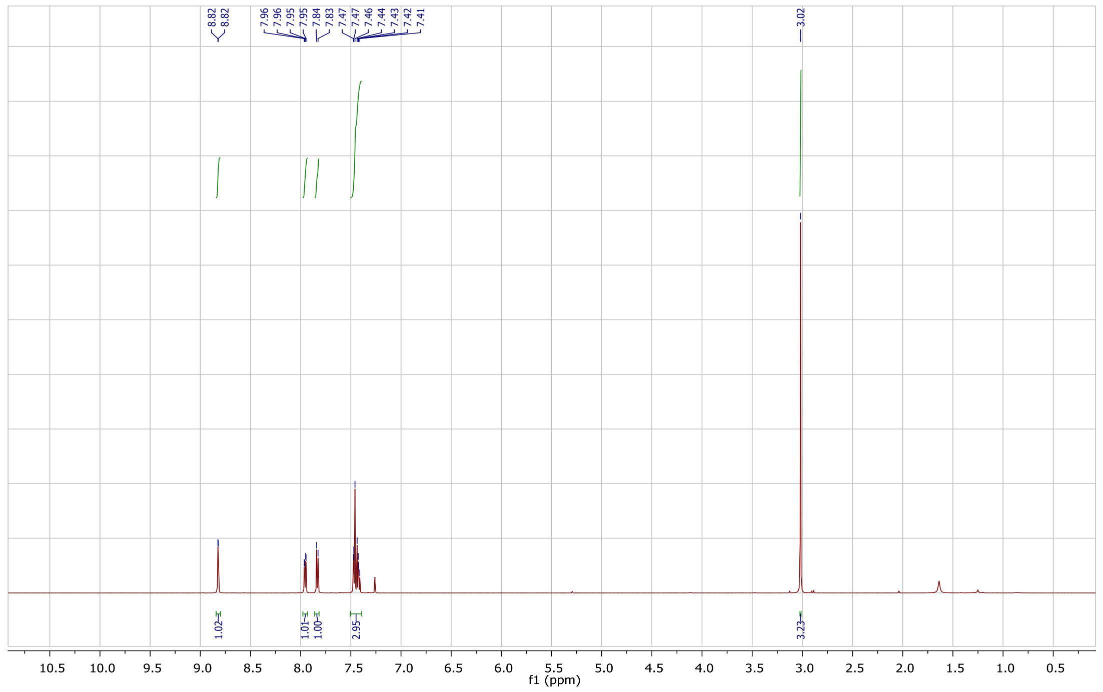

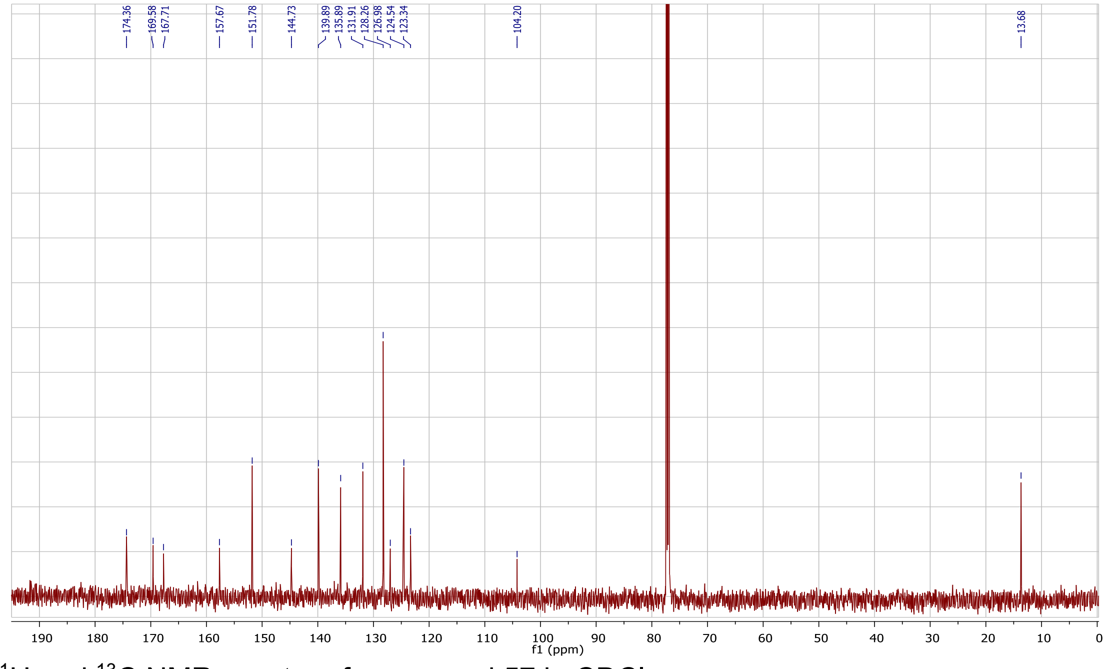

^{1}H and ^{13}C NMR spectra of compound **57** in $CDCl_3$.

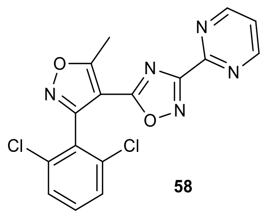

58

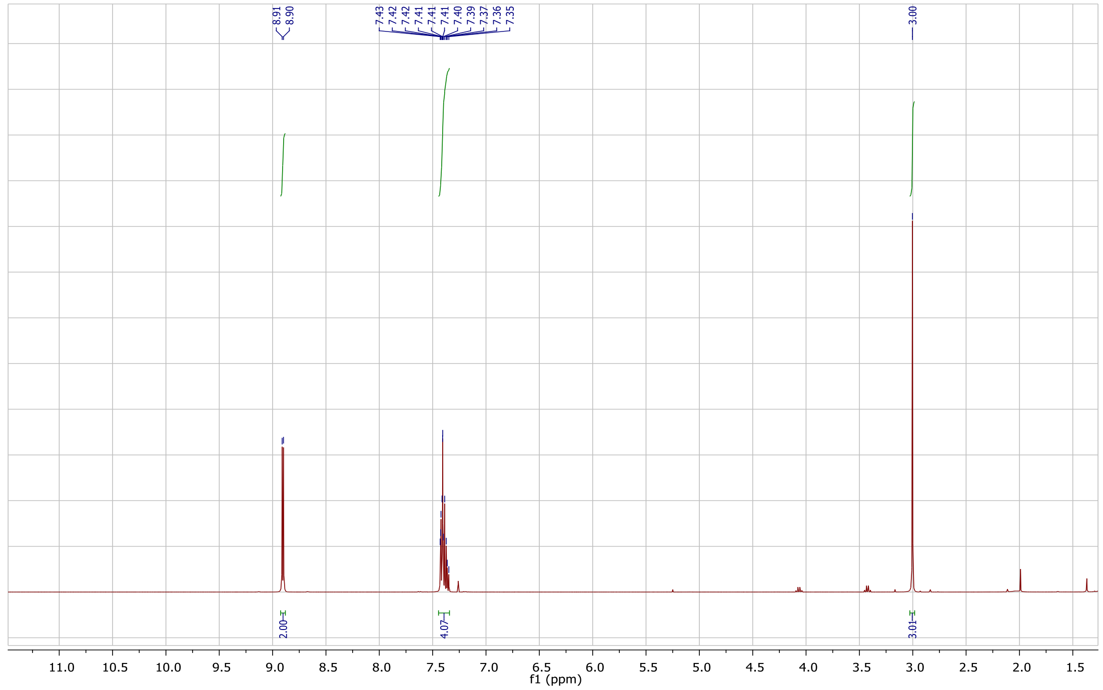

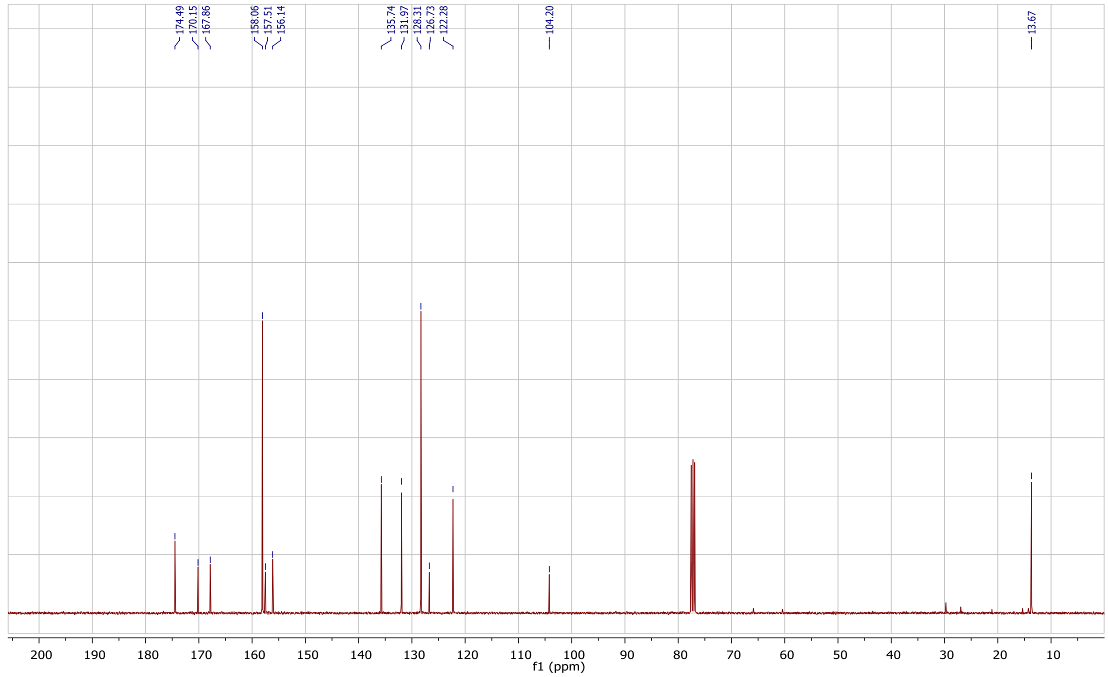

^{1}H and ^{13}C NMR spectra of compound **58** in $CDCl_3$.

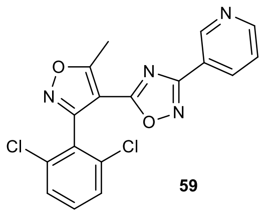

59

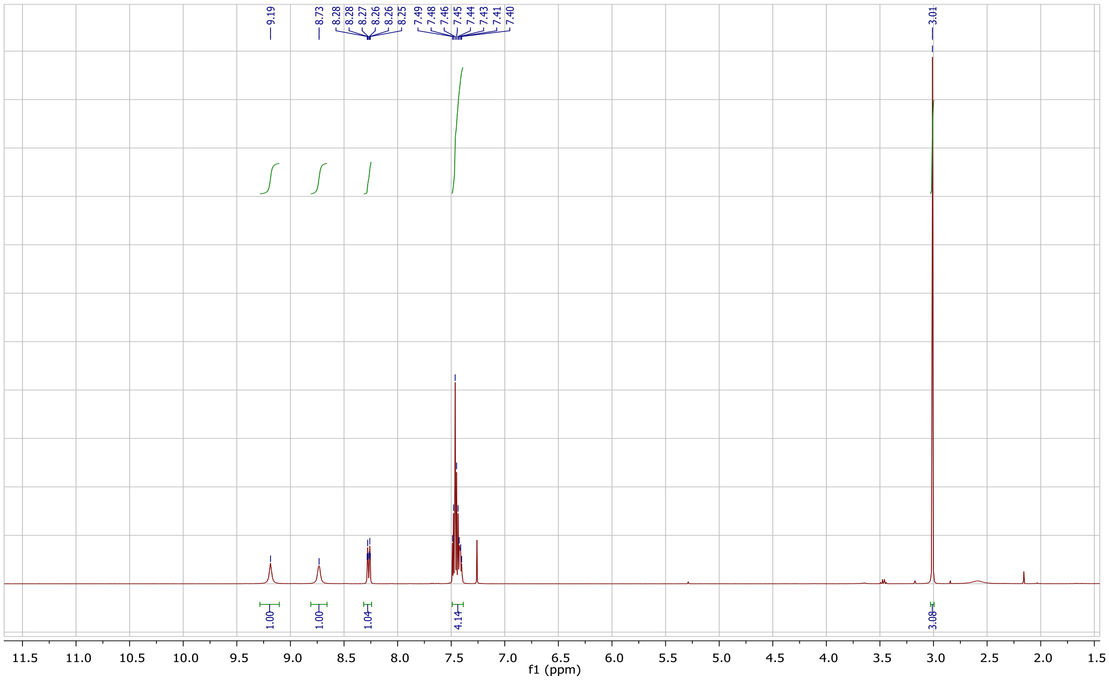

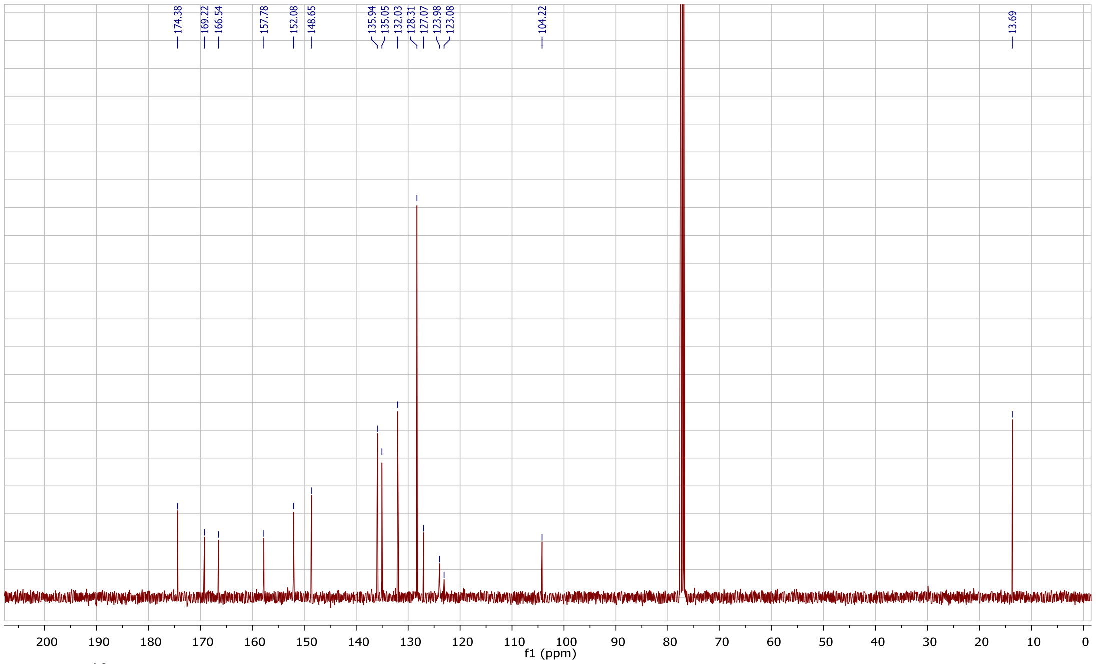

^{1}H and ^{13}C NMR spectra of compound **59** in $CDCl_3$.

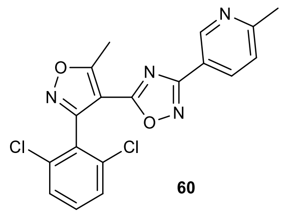

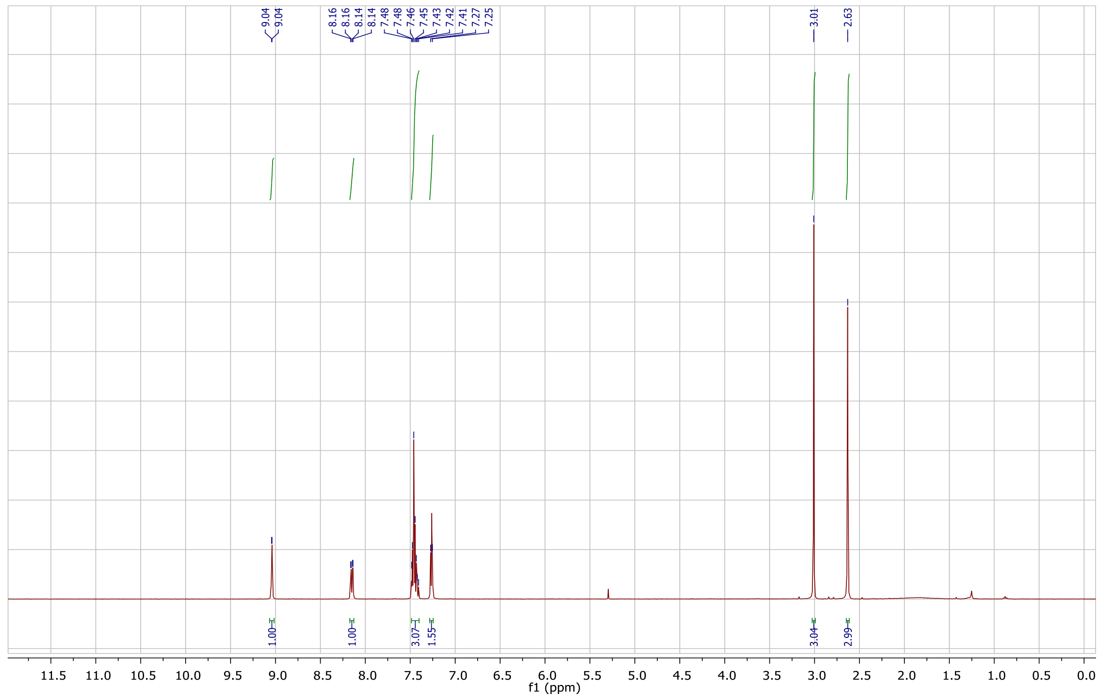

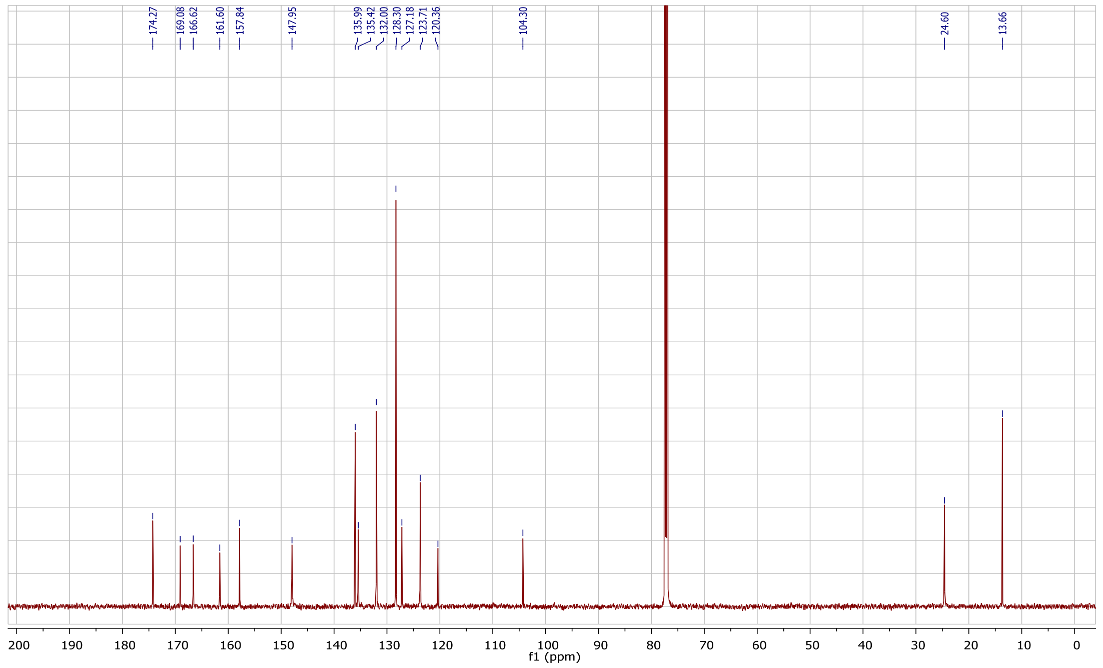

^{1}H and ^{13}C NMR spectra of compound **60** in $CDCl_3$.

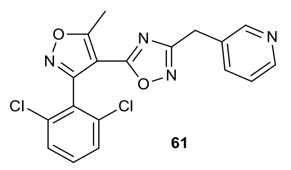

61

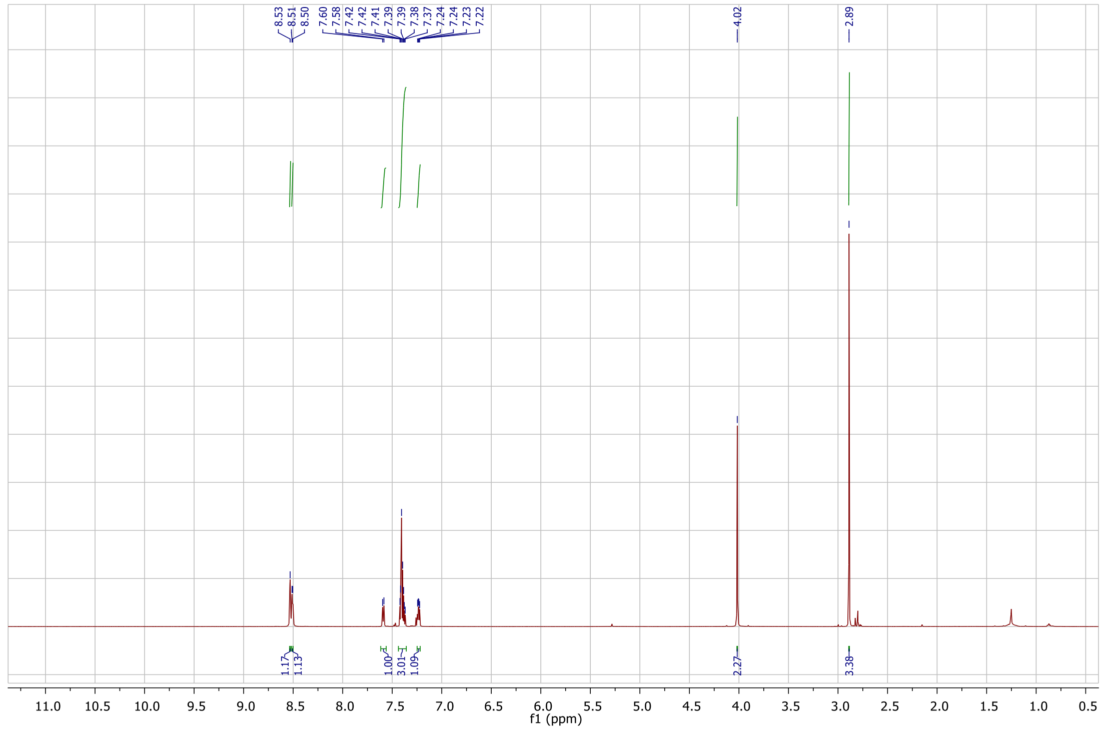

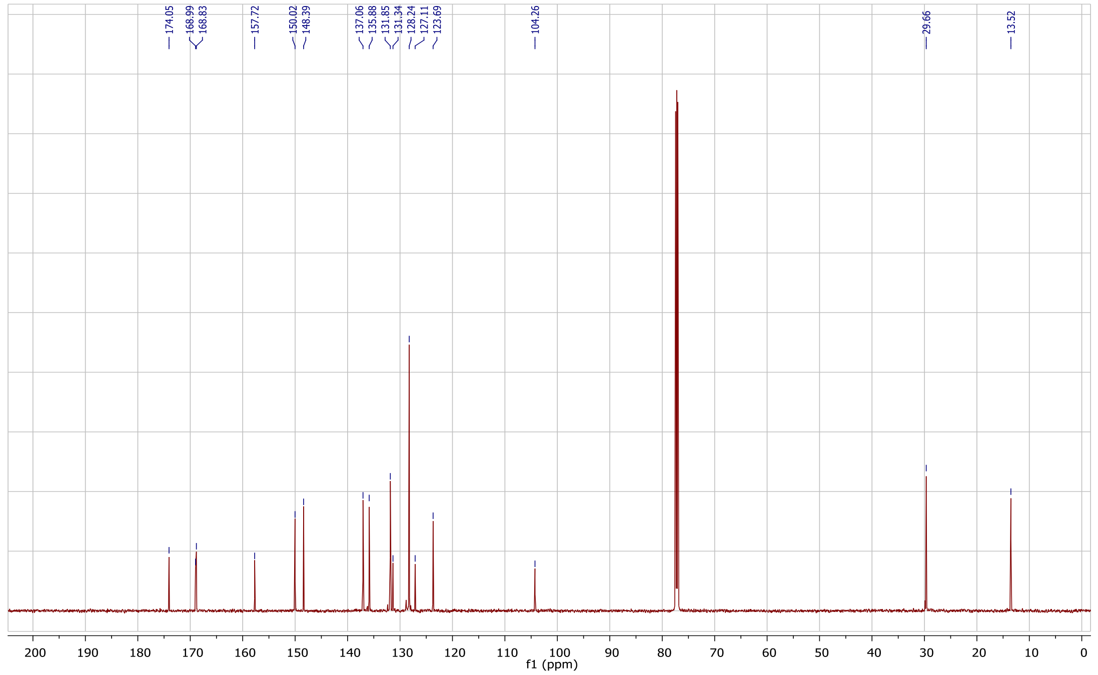

^{1}H and ^{13}C NMR spectra of compound **61** in $CDCl_3$.

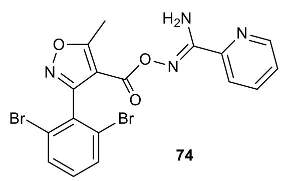

74

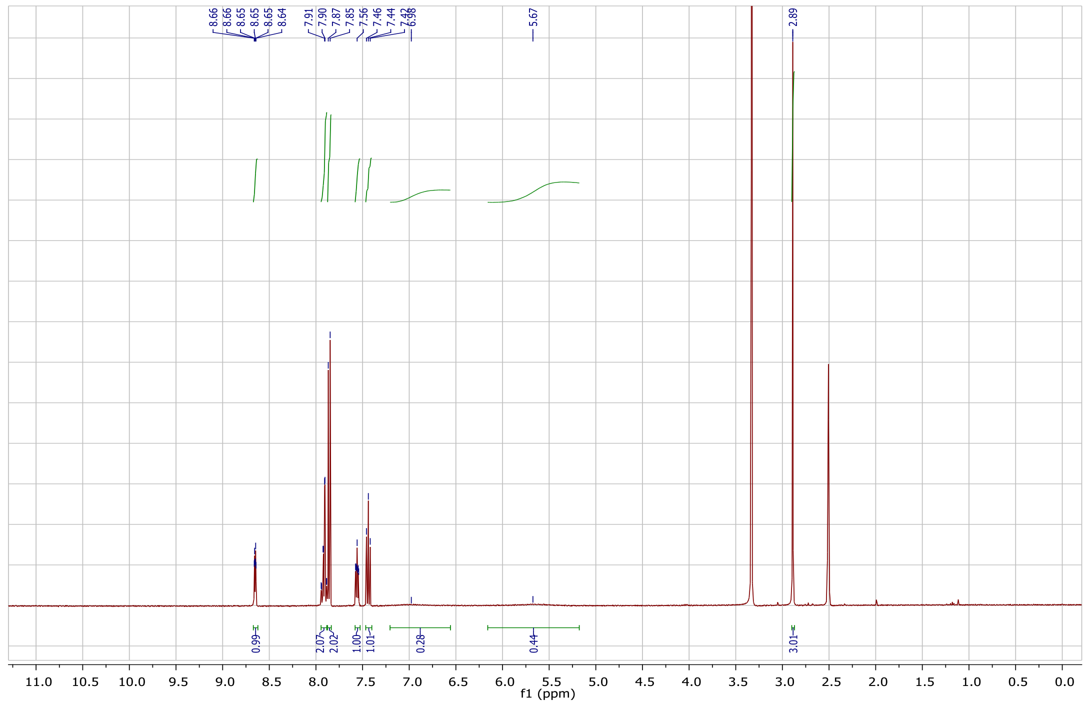

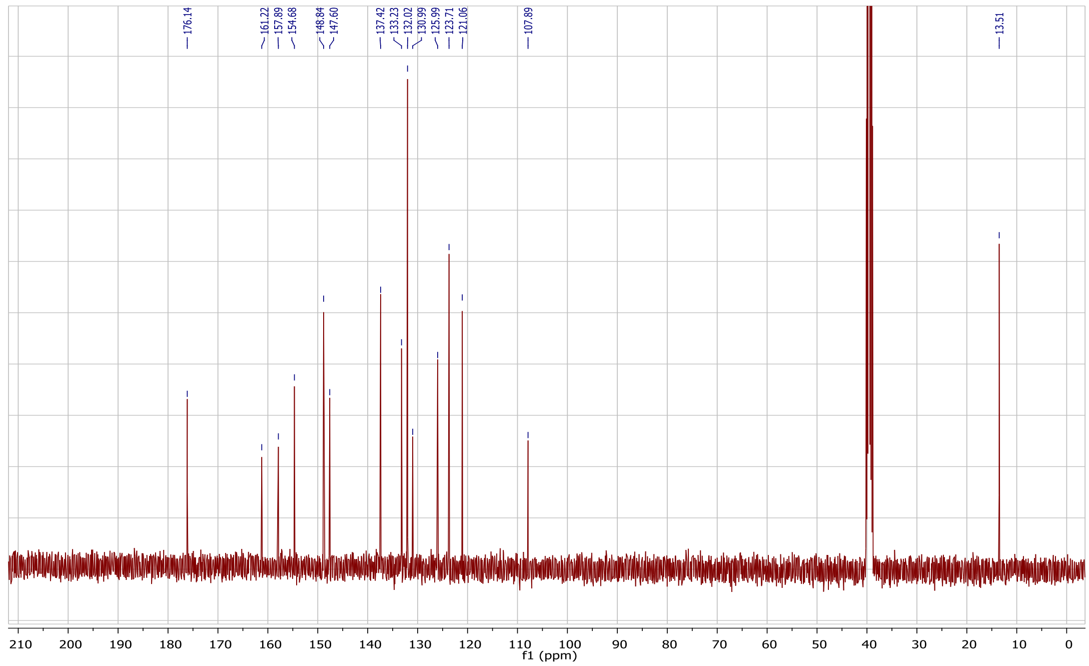

^{1}H and ^{13}C NMR spectra of compound **74** in DMSO-d_6.

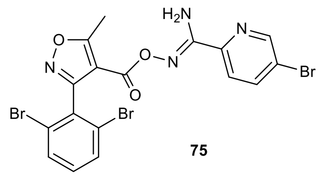

75

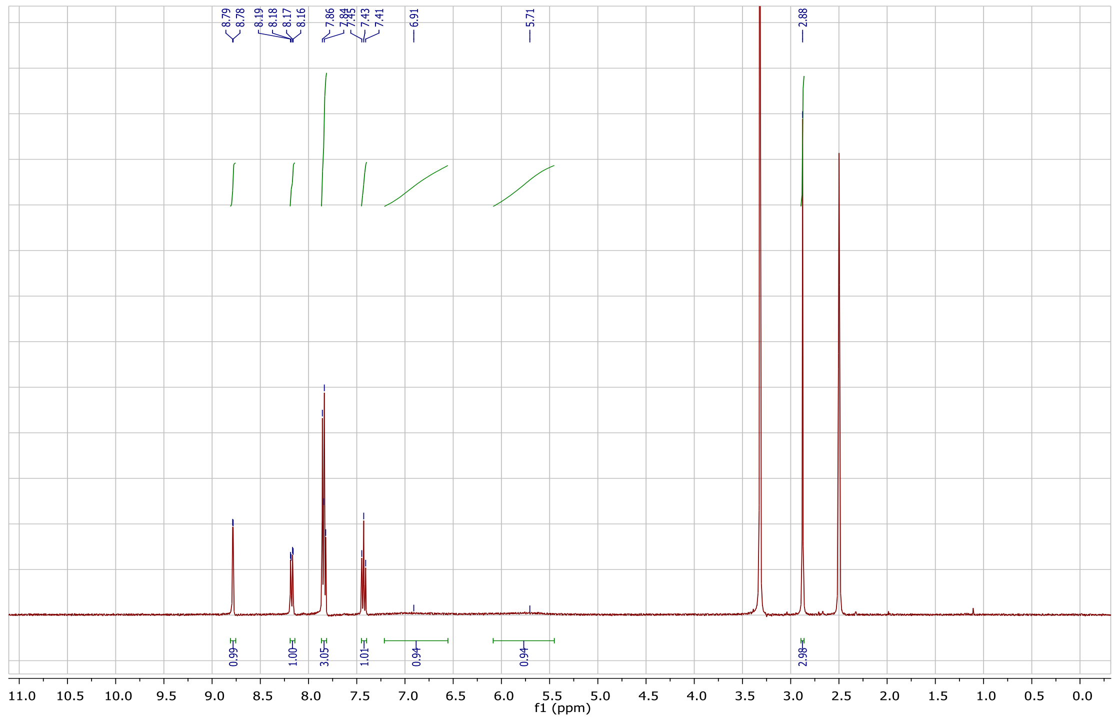

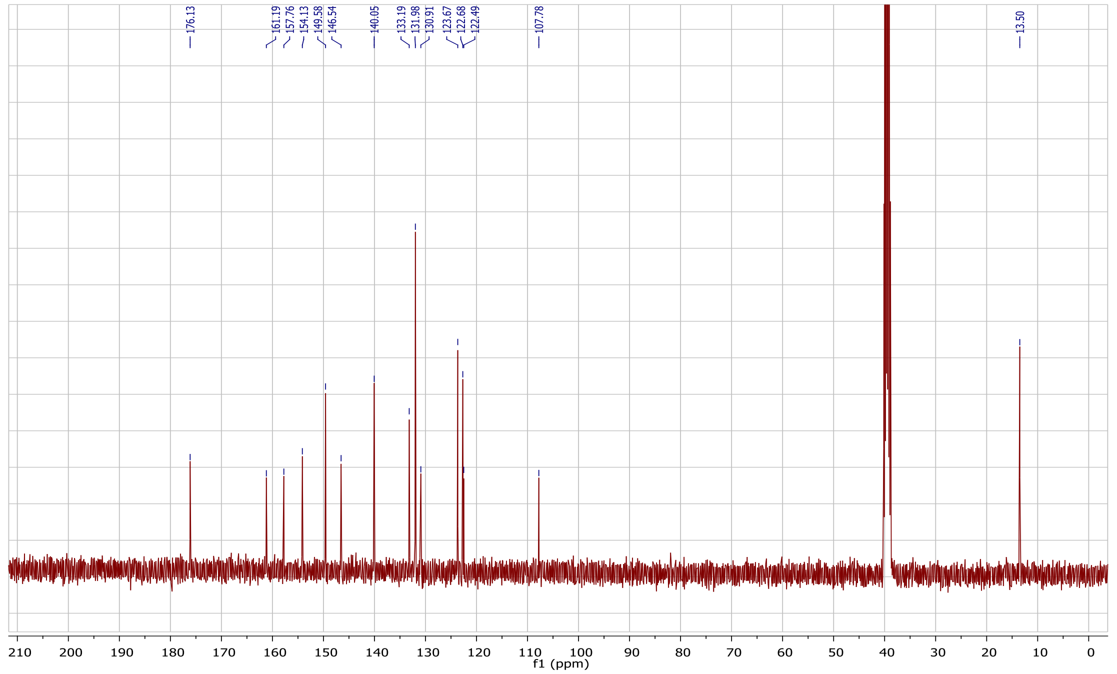

^{1}H and ^{13}C NMR spectra of compound **75** in DMSO-d_6.

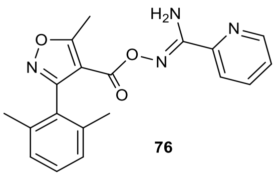

76

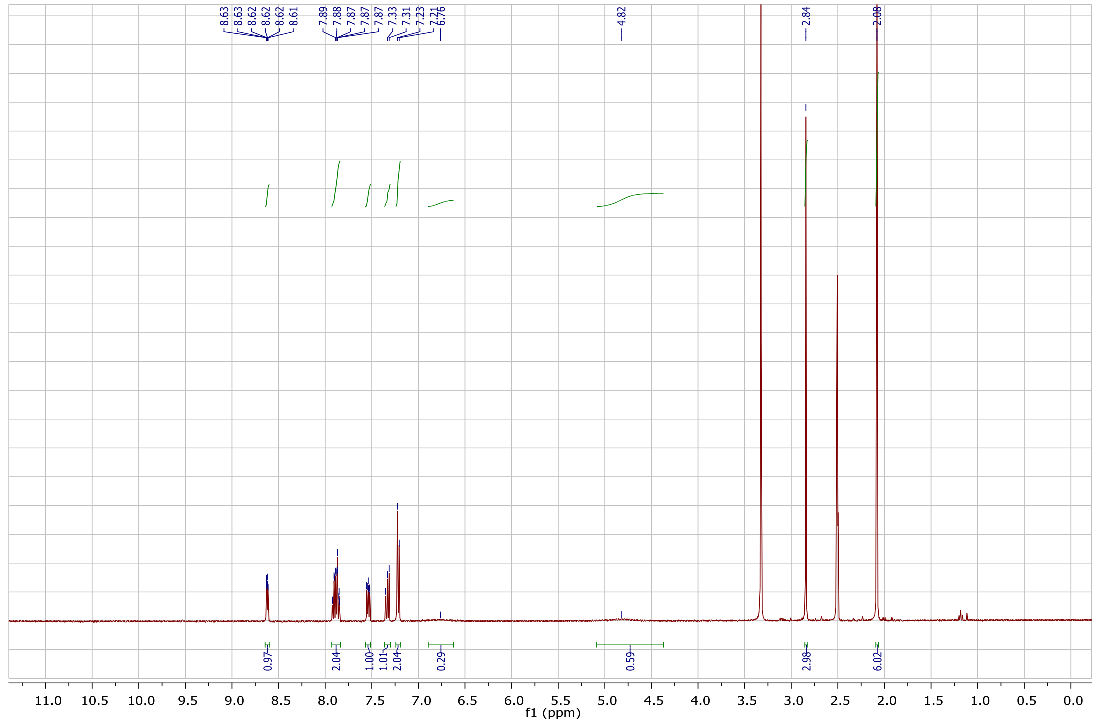

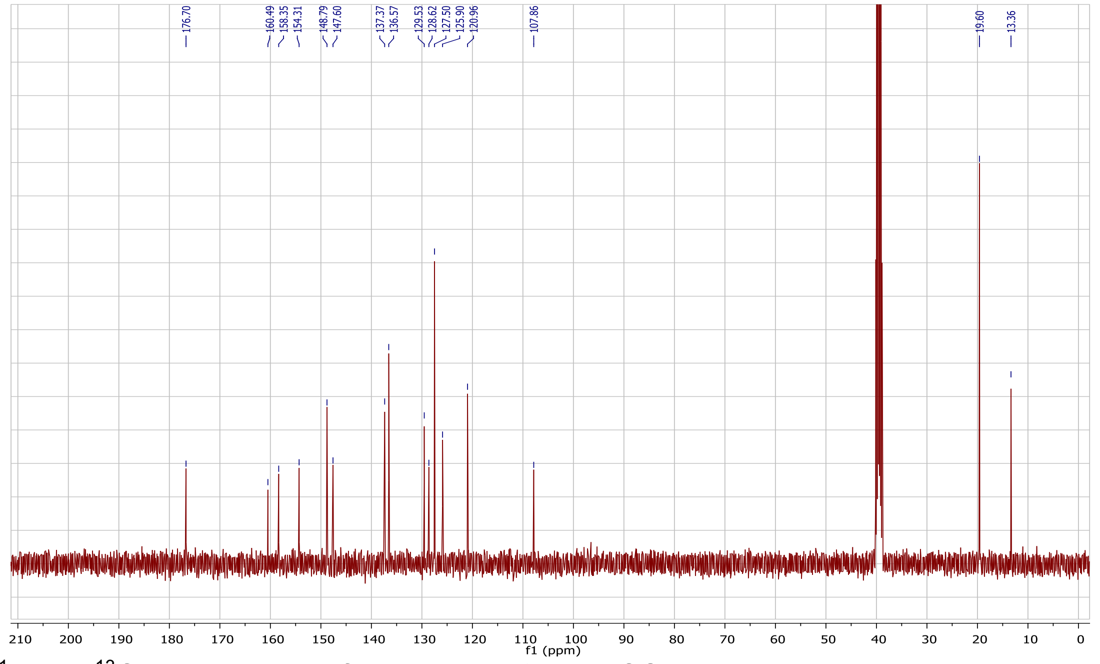

^{1}H and ^{13}C NMR spectra of compound **76** in DMSO-d_6.

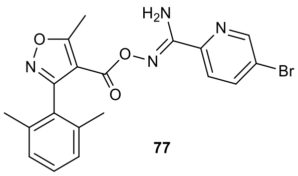

77

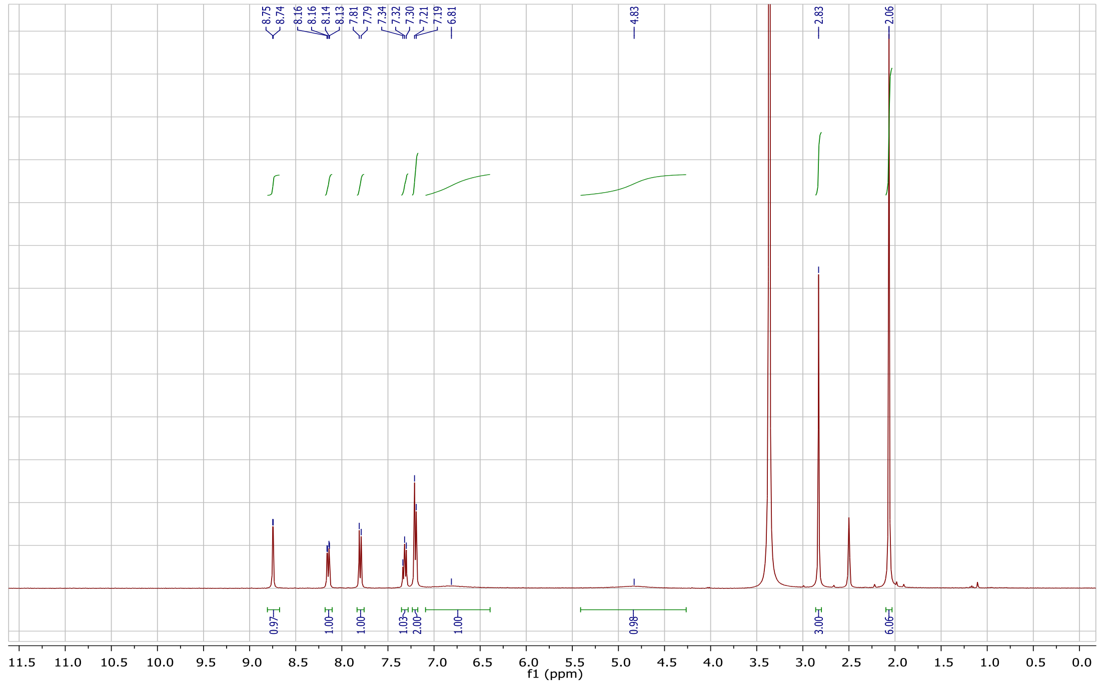

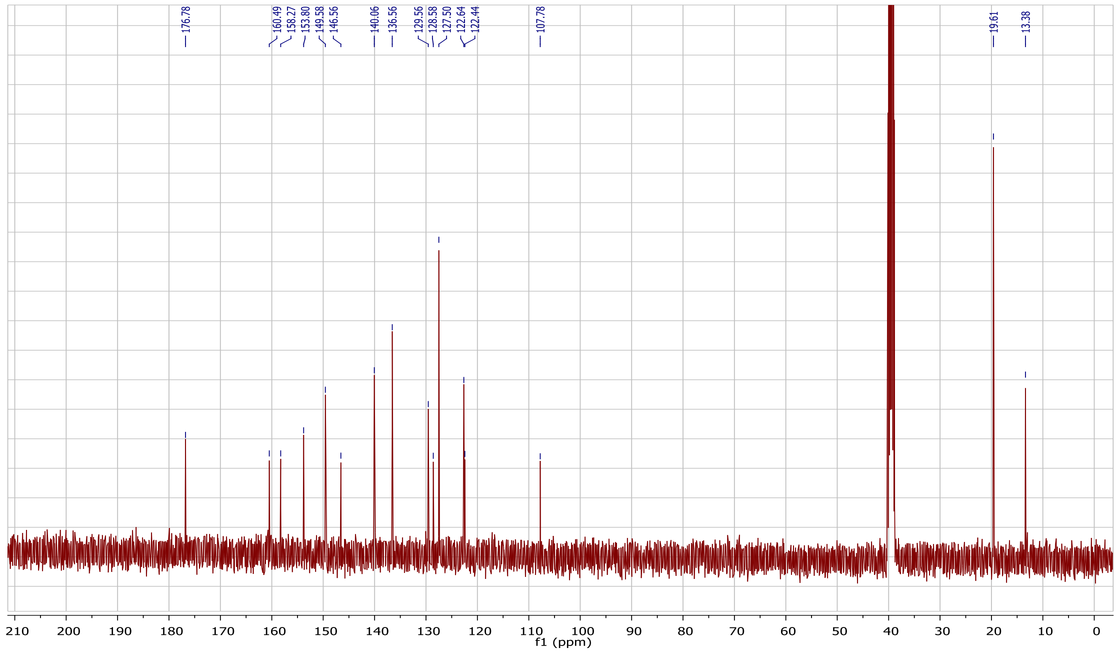

^{1}H and ^{13}C NMR spectra of compound **77** in DMSO-d_6.

Figure S8. Copies of ^{1}H and ^{13}C NMR spectra of the target derivatives **37-61** and **74-77.**

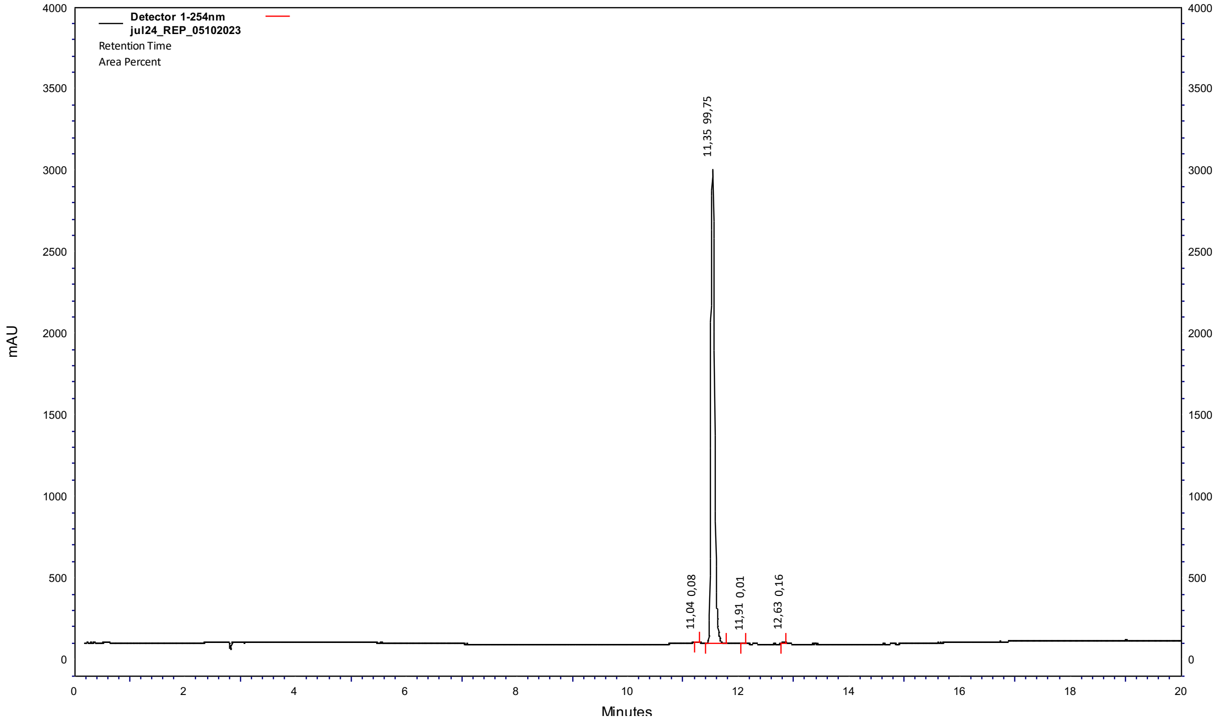

Detector 1- 254nm

Peak	Retention Time	Height	Area	Area %
1	11.04	2380	10974	0.08
2	11.35	2906950	13682603	99.75
3	11.91	617	1371	0.01
4	12.63	4505	21947	0.16
Total			13716895	100.00

HPLC chromatogram of compound **37**, acquired by applying Method A.

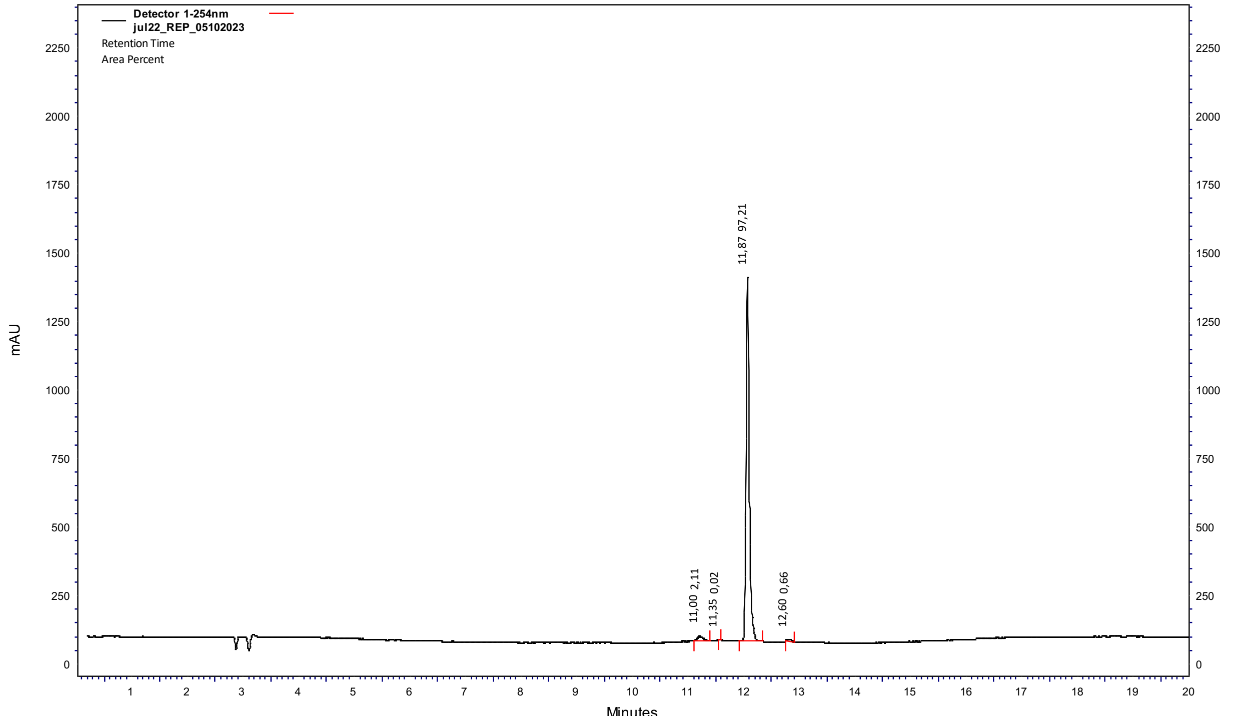

Detector 1- 254nm

Peak	Retention Time	Height	Area	Area %
1	11.00	15471	112202	2.11
2	11.35	961	1262	0.02
3	11.87	1329039	5171698	97.21
4	12.60	7299	34915	0.66
Total			5320077	100.00

HPLC chromatogram of compound **39**, acquired by applying Method A.

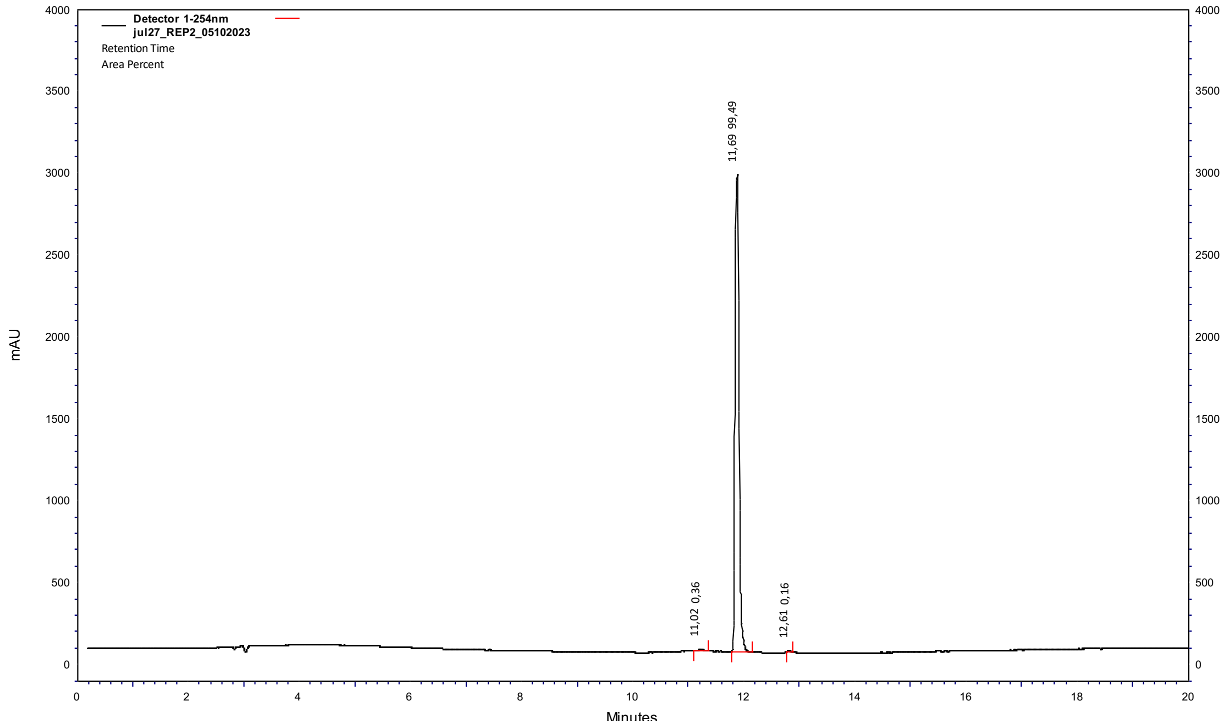

Detector 1- 254nm

Peak	Retention Time	Height	Area	Area %
1	11.02	8448	51913	0.36
2	11.69	2913561	14545664	99.49
3	12.61	5703	22903	0.15
Total			14620480	100.00

HPLC chromatogram of compound **40**, acquired by applying Method A.

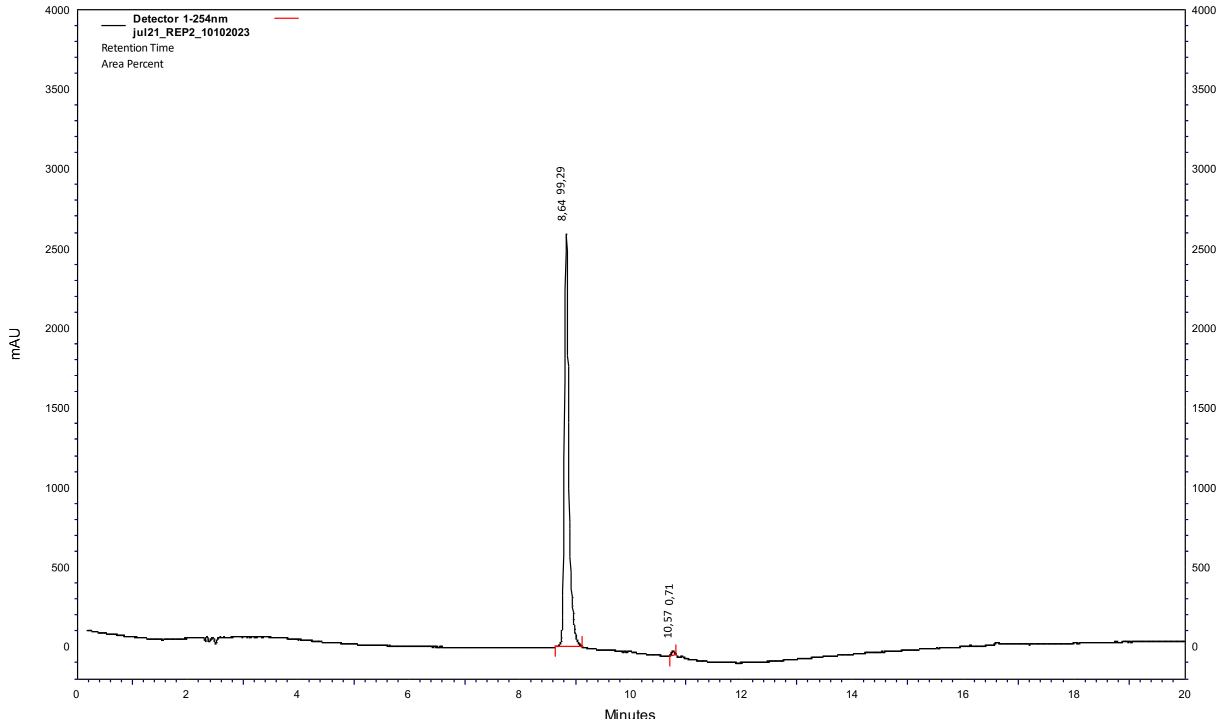

Detector 1- 254nm

Peak	Retention Time	Height	Area	Area %
1	8.64	2583856	14422569	99.29
2	10.57	27717	103450	0.71
Total			14526019	100.00

HPLC chromatogram of compound **47**, acquired by applying Method B.

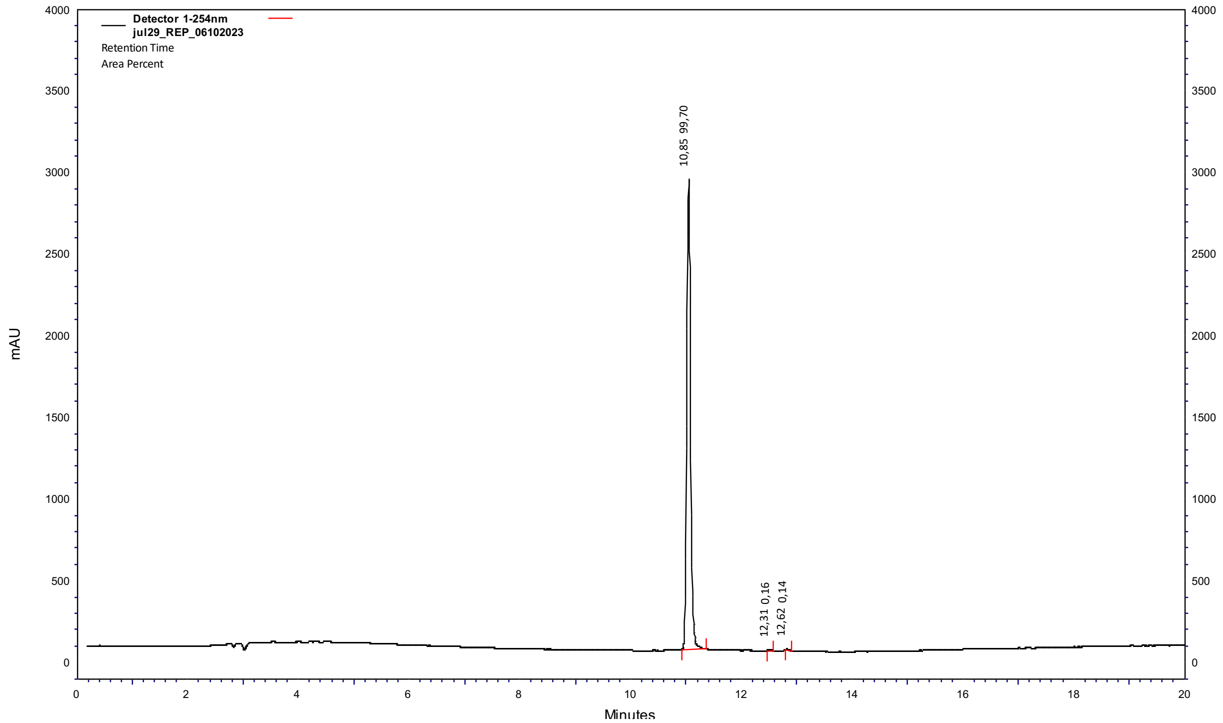

Detector 1- 254nm

Peak	Retention Time	Height	Area	Area %
1	10.85	2882095	13950100	99.70
2	12.31	6031	22888	0.16
3	12.62	5027	19127	0.14
Total			13992115	100.00

HPLC chromatogram of compound **48**, acquired by applying Method A.

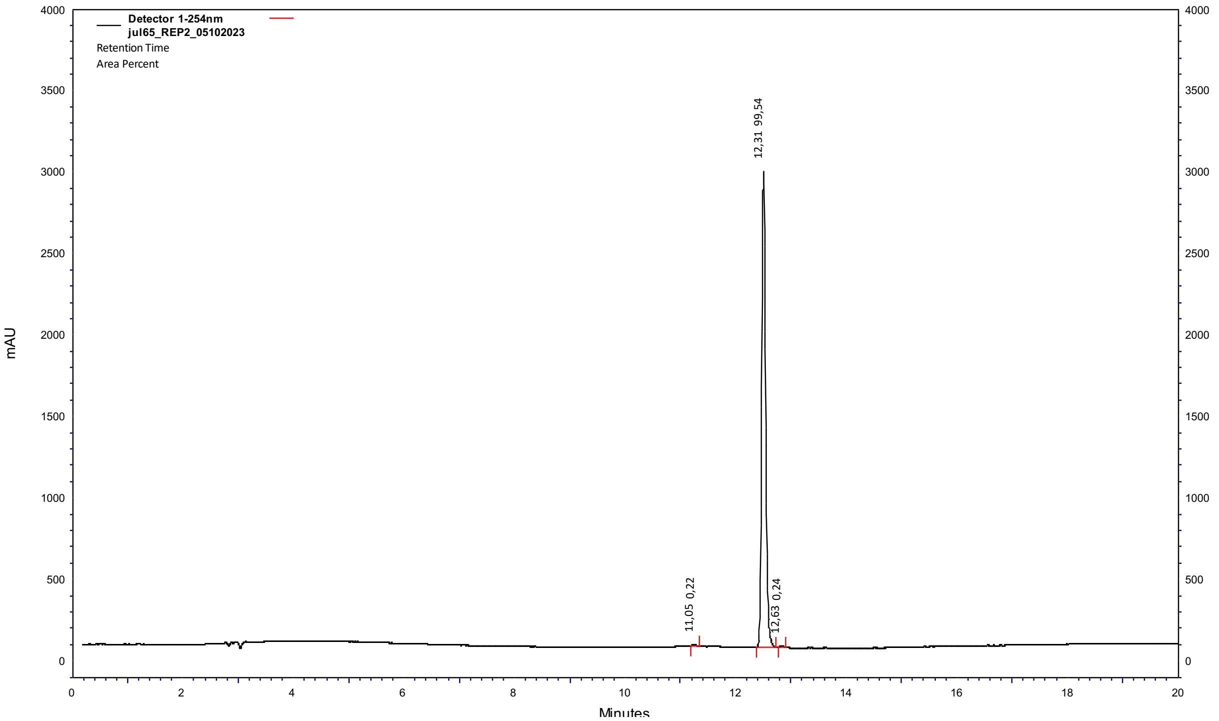

Detector 1- 254nm

Peak	Retention Time	Height	Area	Area %
1	11.05	6712	32369	0.22
2	12.31	2924539	14888188	99.54
3	12.63	8150	36291	0.24
Total			14956848	100.00

HPLC chromatogram of compound **59**, acquired by applying Method A.

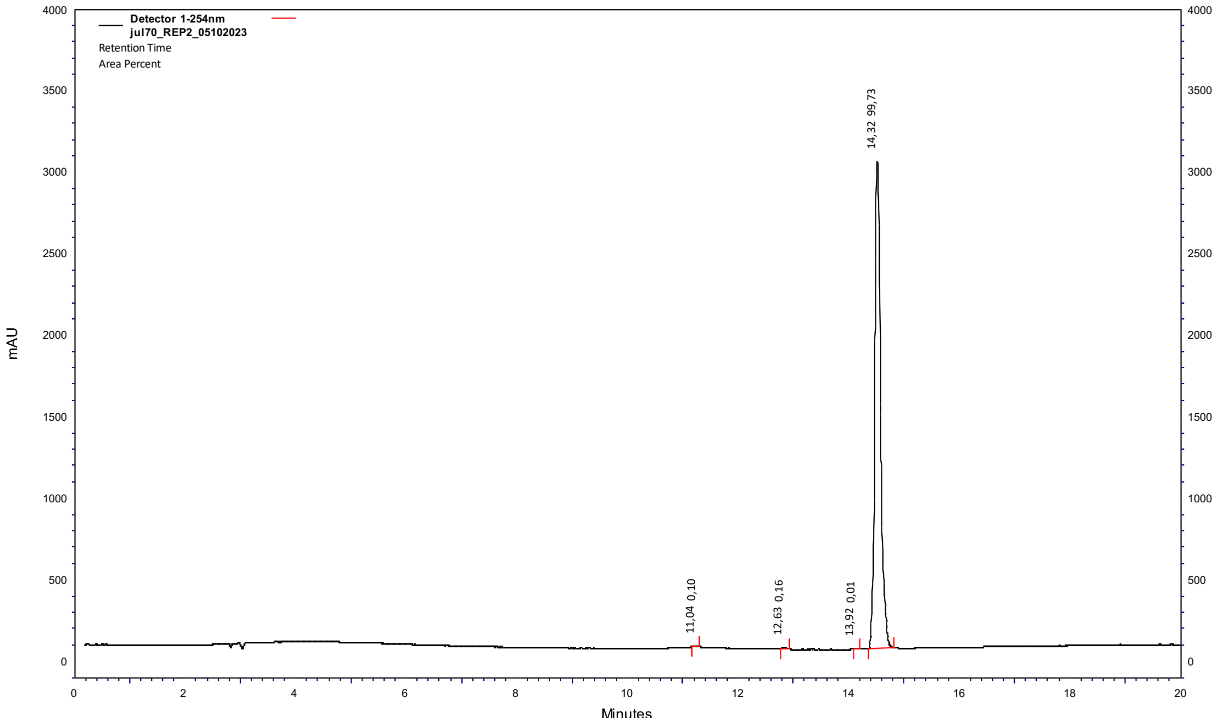

Detector 1- 254nm

Peak	Retention Time	Height	Area	Area %
1	11.04	4561	22350	0.10
2	12.63	7079	35498	0.16
3	13.92	887	2339	0.01
4	14.32	2988347	22079643	99.73
Total			22139830	100.00

HPLC chromatogram of compound **60**, acquired by applying Method A.

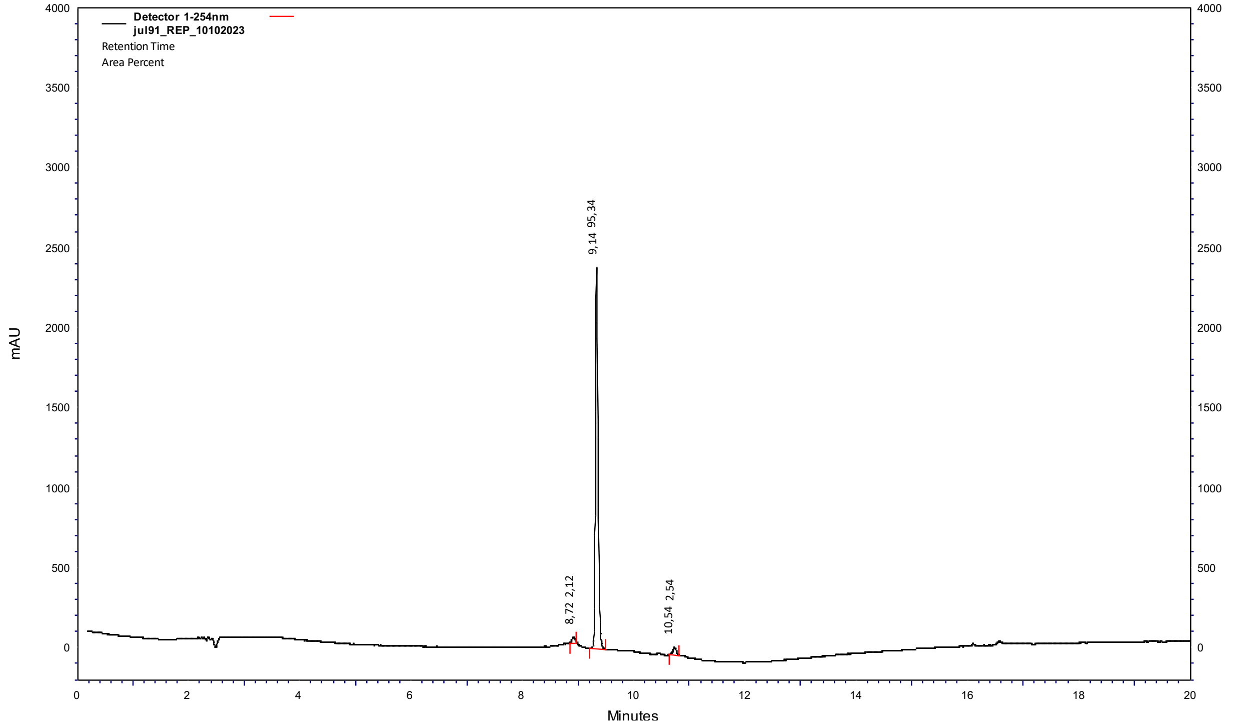

Detector 1- 254nm

Peak	Retention Time	Height	Area	Area %
1	8.72	38022	168330	2.12
2	9.14	2383603	7586104	95.34
3	10.54	45826	202069	2.54
Total			7956503	100.00

HPLC chromatogram of compound **74**, acquired by applying Method B.

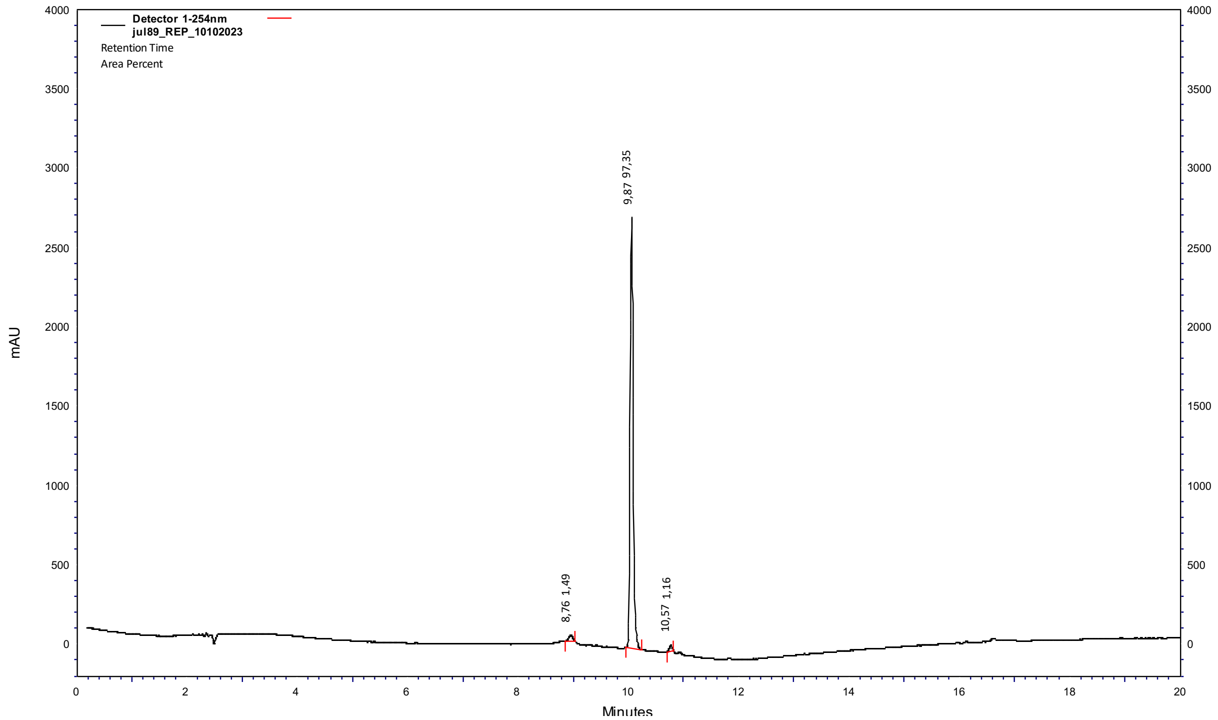

Detector 1- 254nm

Peak	Retention Time	Height	Area	Area %
1	8.76	37221	164981	1.49
2	9.87	2716378	10788980	97.35
3	10.57	36642	128163	1.16
Total			11082124	100.00

HPLC chromatogram of compound **75**, acquired by applying Method B.

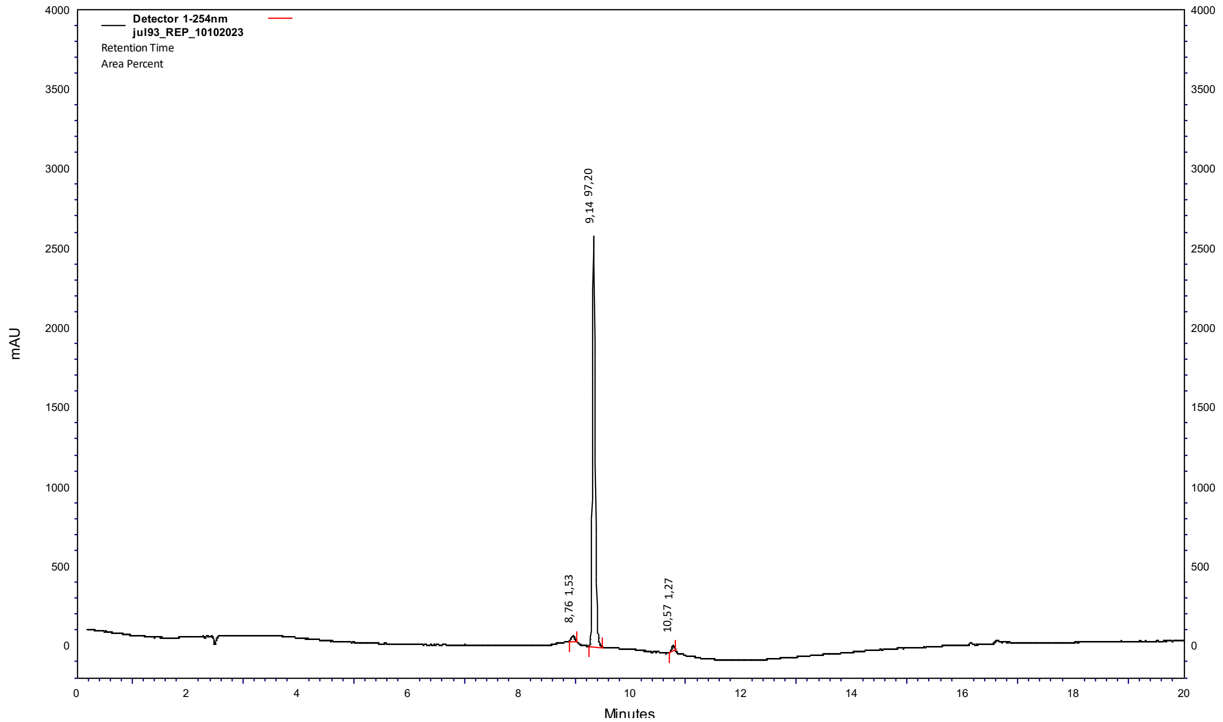

Detector 1- 254nm

Peak	Retention Time	Height	Area	Area %
1	8.76	32137	140761	1.53
2	9.14	2574451	8962587	97.20
3	10.57	34224	117043	1.27
Total			9220391	100.00

HPLC chromatogram of compound **76**, acquired by applying Method B.

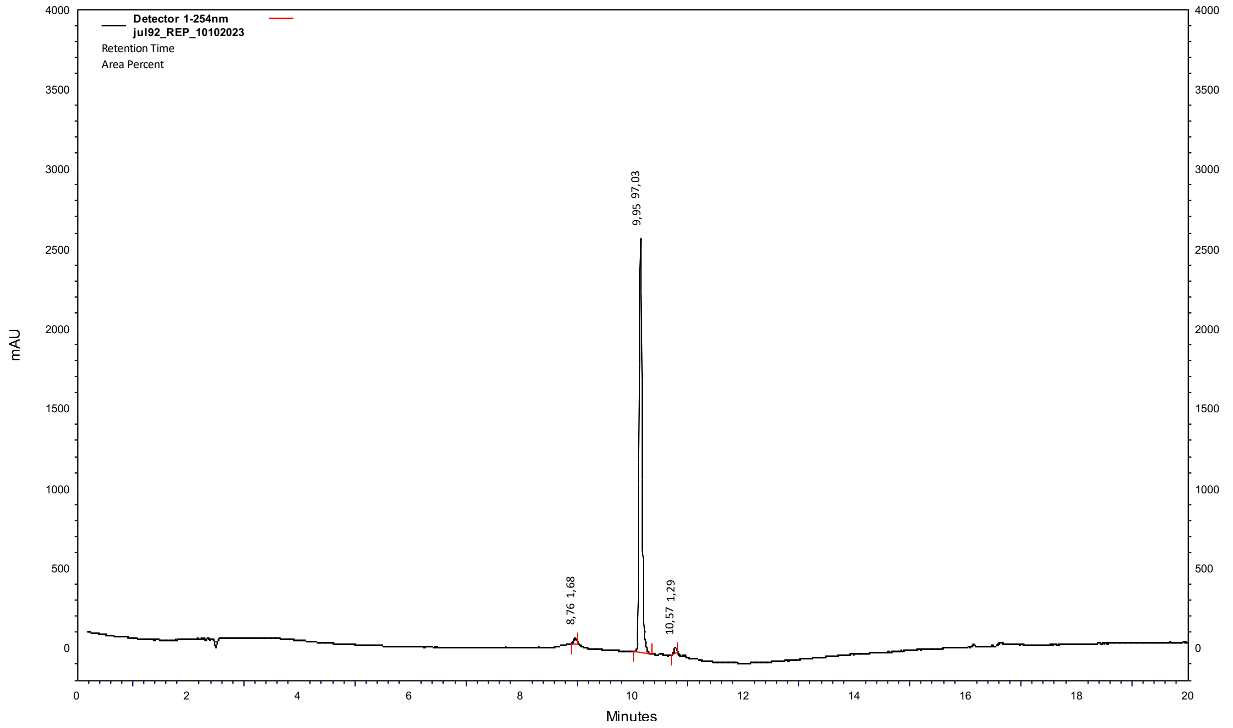

Detector 1- 254nm

Peak	Retention Time	Height	Area	Area %
1	8.76	36271	156251	1.68
2	9.95	2591195	9032736	97.03
3	10.57	35532	120049	1.29
Total			9309036	100.00

HPLC chromatogram of compound **77**, acquired by applying Method B.

Figure S9. Copies of HPLC chromatograms of the most active target compounds.

Supporting Information References

(1) He, X.; Liu, S.; Lee, T.-S.; Ji, B.; Man, V. H.; York, D. M.; Wang, J. Fast, Accurate, and Reliable Protocols for Routine Calculations of Protein–Ligand Binding Affinities in Drug Design Projects Using AMBER GPU-TI with Ff14SB/GAFF. *ACS Omega* **2020**, *5* (9), 4611–4619. https://doi.org/10.1021/acsomega.9b04233.

(2) Song, L. F.; Lee, T. S.; Zhu, C.; York, D. M.; Merz, K. M. Using AMBER18 for Relative Free Energy Calculations. *J Chem Inf Model* **2019**, *59* (7), 3128–3135. https://doi.org/10.1021/acs.jcim.9b00105.

(3) Maier, J. A.; Martinez, C.; Kasavajhala, K.; Wickstrom, L.; Hauser, K. E.; Simmerling, C. Ff14SB: Improving the Accuracy of Protein Side Chain and Backbone Parameters from Ff99SB. *J Chem Theory Comput* **2015**, *11* (8), 3696–3713. https://doi.org/10.1021/acs.jctc.5b00255.

(4) Stampelou, M.; Ladds, G.; Kolocouris, A. *Drug Design Using Kinetic and Thermodynamic Binding Calculations: A Case Study for the Unresolved Inactive Human Adenosine A3 Receptor*; 2023. https://doi.org/10.26434/chemrxiv-2023-llhdq.

(5) Heo, L.; Feig, M. Multi-state Modeling of G-protein Coupled Receptors at Experimental Accuracy. *Proteins: Structure, Function, and Bioinformatics* **2022**, *90* (11), 1873–1885. https://doi.org/10.1002/prot.26382.

(6) Sala, D.; Hildebrand, P. W.; Meiler, J. Biasing AlphaFold2 to Predict GPCRs and Kinases with User-Defined Functional or Structural Properties. *Front Mol Biosci* **2023**, *10*. https://doi.org/10.3389/fmolb.2023.1121962.

(7) Cai, H.; Guo, S.; Xu, Y.; Sun, J.; Li, J.; Xia, Z.; Jiang, Y.; Xie, X.; Xu, H. E. Cryo-EM Structures of Adenosine Receptor A3AR Bound to Selective Agonists. *Nat Commun* **2024**, *15* (1), 3252. https://doi.org/10.1038/s41467-024-47207-6.

(8) Stampelou, M.; Ladds, G.; Kolocouris, A. Computational Workflow for Refining AlphaFold Models in Drug Design Using Kinetic and Thermodynamic Binding Calculations: A Case Study for the Unresolved Inactive Human Adenosine A3 Receptor. *J Phys Chem B* **2024**. https://doi.org/10.1021/acs.jpcb.3c05986.

(9) Tian, C.; Kasavajhala, K.; Belfon, K. A. A.; Raguette, L.; Huang, H.; Migues, A. N.; Bickel, J.; Wang, Y.; Pincay, J.; Wu, Q.; Simmerling, C. Ff19SB: Amino-Acid-Specific Protein Backbone Parameters Trained against Quantum Mechanics Energy Surfaces in Solution. *J Chem Theory Comput* **2020**, *16* (1), 528–552. https://doi.org/10.1021/acs.jctc.9b00591.

(10) Pándy-Szekeres, G.; Munk, C.; Tsonkov, T. M.; Mordalski, S.; Harpsøe, K.; Hauser, A. S.; Bojarski, A. J.; Gloriam, D. E. GPCRdb in 2018: Adding GPCR Structure Models and Ligands. *Nucleic Acids Res* **2018**, *46* (D1), D440–D446. https://doi.org/10.1093/NAR/GKX1109.
